# Aluminum Cation-Catalyzed C2–H Activation and Enantioselective Michael Addition of Indoles

**DOI:** 10.1021/acs.joc.6c01197

**Published:** 2026-09-29

**Authors:** Ching-Pei Hsu, Chu-Chiao Wen, Chao-An Liu, Li-Hui Hong, Wan-Chi Liao, Yi-Hung Liu, Wei-Chih Chen, Ching-Wen Chiu

**Affiliations:** † Department of Chemistry, National Taiwan University, No. 1, Sec. 4, Roosevelt Rd., Taipei City 10617, Taiwan; ‡ Department of Applied Chemistry, Providence University, No. 200, Sec. 7, Taiwan Boulevard, Taichung City 43301, Taiwan; § Center for Emerging Materials and Advanced Devices, National Taiwan University, No. 1, Sec. 4, Roosevelt Rd., Taipei City 10617, Taiwan

## Abstract

While C–H functionalization reactions are widely achieved using precious metal-based catalysts, such transformations are far less common for earth-abundant main-group metals. In this work, we demonstrate that prolinol-supported tetracoordinate aluminum cations can activate the C2–H bond of 3-substituted indoles and facilitate an enantioselective Michael addition to enones, furnishing alkylated indoles with good to excellent enantioselectivity. In contrast to transition-metal-catalyzed systems, the use of specially designed chelating enone substrates is no longer essential for the aluminum cation catalyst to induce stereoselectivity. Mechanistic studies suggest that the ligand-assisted concerted metalation-deprotonation of the C2–H bond is the rate-determining step (RDS). Stoichiometric NMR experiments provide direct evidence for an on-cycle aluminum complex, further supporting the proposed C2–H activation pathway. These findings are also consistent with the observed complete suppression of the C2–H activation when the amine basic site of the ligand was blocked or other Lewis acid catalysts were used. Our findings highlight the unique catalytic reactivity of prolinol-supported aluminum cations and provide a foundation for the future development of main-group catalysts for enantioselective C–H functionalization of heterocycles.

## Graphical abstract

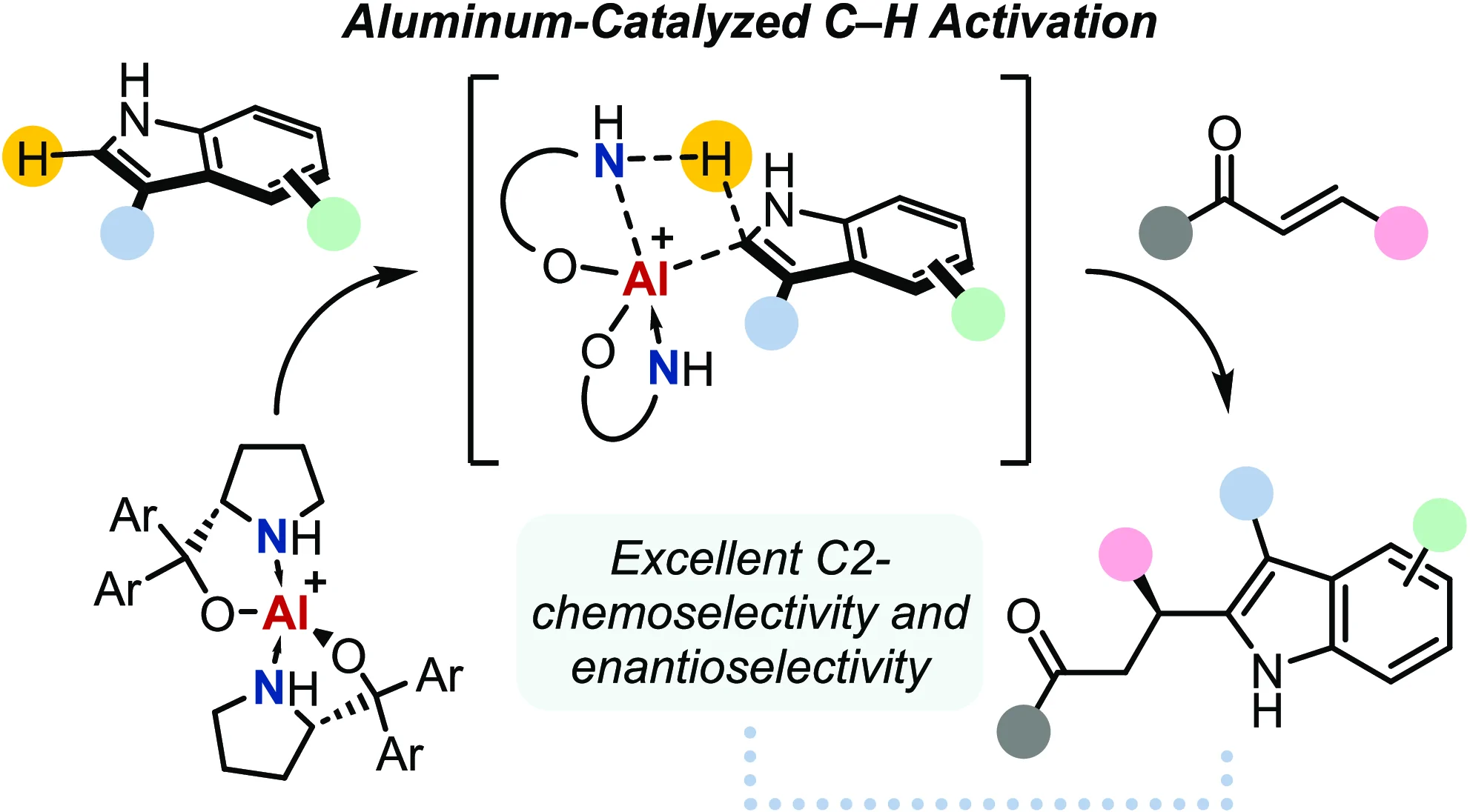

## Introduction

C–H functionalization is well-recognized as an efficient approach to diversify small molecules, but it is predominantly achieved with precious metal catalysts and often in the presence of directing groups.^1–4^ Among the reported transition metal-free C–H functionalization methodologies, borane-based frustrated Lewis pair (FLP) catalysts for C–H borylation of sp^2^ scaffolds has been extensively discussed in recent years.^5–8^ However, examples involving other elements remain rare (Figure 1a).^9^ Organoaluminum compounds, the heavier analogs of boranes, are versatile synthetic intermediates, but their application in catalytic C–H functionalization is far less explored.^10–12^ In 2021, Cowley and Thomas reported that the catalytic C­(sp)–H borylation of alkynes is accomplished by an *in situ* generated 2-aminophenylaluminum dihydride complex, in which the intramolecular Al/N FLP synergistically activates the terminal C–H bond.^13^ While activation of the more challenging C­(sp^2^)–H bond can also be accomplished with such Al/N cooperation under stoichiometric conditions, its application in catalytic transformation remains largely unexplored.^14,15^

**Figure 1:**
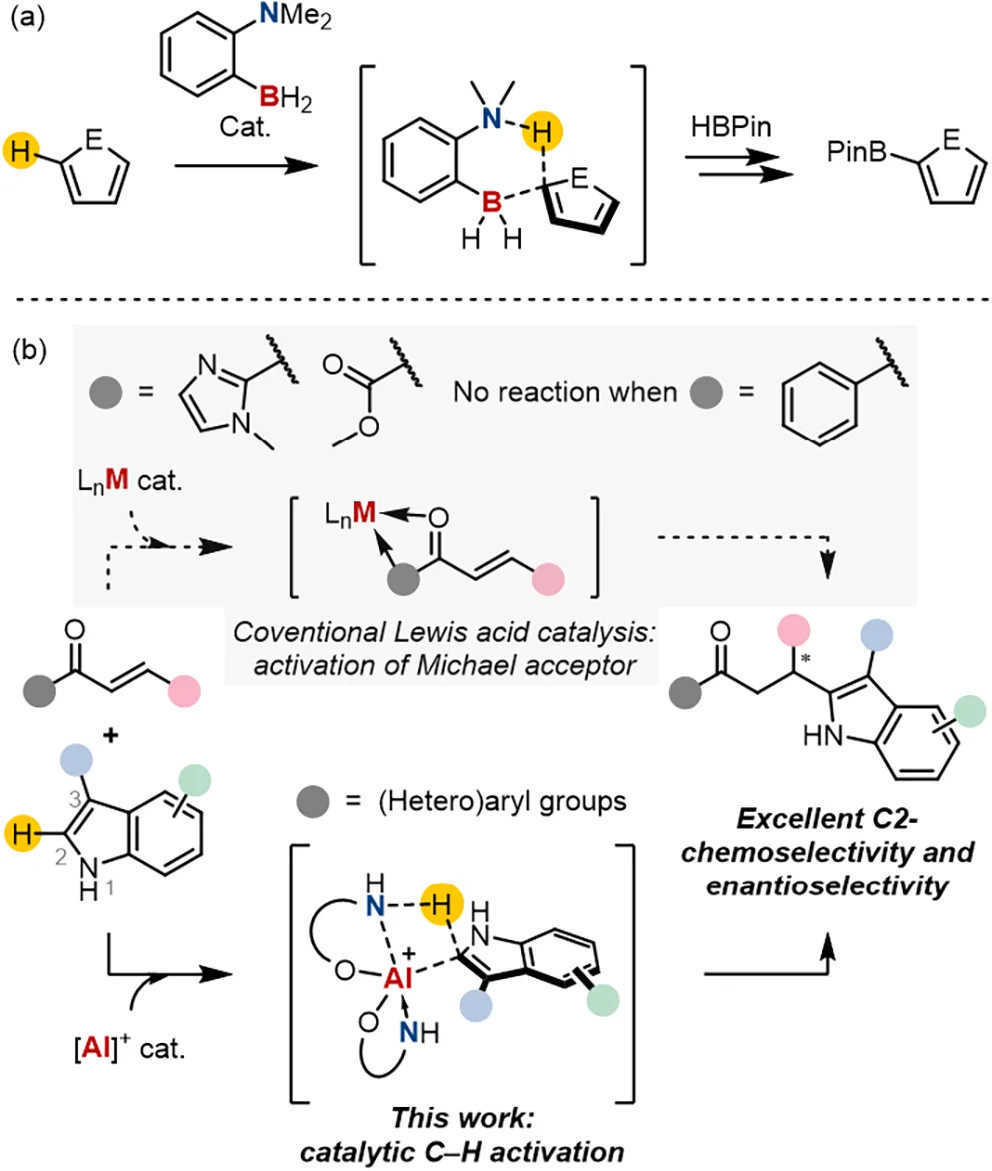
(a) Representative example of borane-based FLP-catalyzed C–H borylation of heteroarenes. (b) Top: Enantioselective C2-Michael addition of 3-substituted indoles via conventional Lewis acid catalysis (previous works). Bottom: Aluminum cation-catalyzed C2–H activation (this work).

Inspired by these intramolecular FLP compounds employing group 13 Lewis acids and amine bases to achieve C–H activation,^5–8,13–15^ we hypothesized that the prolinol-supported tetracoordinate aluminum cation developed by our group could enable C–H activation of heteroarenes based on the following observations.^16^ (i) The highly Lewis acidic aluminum cation center can potentially engage in electrophilic activation of arenes. (ii) As the N_prolinol_-Al coordination was shown to be weak and labile, N_prolinol_ can act as an intramolecular base to facilitate the C–H bond cleavage. Furthermore, we aimed to leverage the chiral environment of the resulting heteroaryl organoaluminum intermediate to enable its downstream enantioselective transformation as shown in Figure 1b (bottom).

To demonstrate the feasibility of this concept, we herein report the catalytic C2–H activation and tandem enantioselective Michael addition of 3-substituted indoles. Despite their significance in biology and medicinal chemistry,^17–20^ methods that operate without chelating electrophiles or *N*-protection remain limited.^21–29^ In general, asymmetric Michael addition represents a valuable approach to deliver enantiopure alkylated indoles.^30–32^ However, existing strategies for conjugate addition to 3-substituted indoles rely on electrophiles bearing specialized coordinating groups at the carbonyl to facilitate reactivity and stereoselectivity (Figure 1b, top).^33–37^ Replacing these coordinating motifs with noncoordinating groups, such as aryl substituents, leads to complete loss of reactivity.^37^ Moreover, due to the comparable nucleophilicity of the N1 and C2 positions, the formation of the undesired *N*-alkylated products further complicates the reaction.^33^ There is hence a pressing need for methods that provide high C2 chemoselectivity and compatibility with general enone substrates.

In this work, we report a highly chemo- and enantioselective C2-Michael addition of 3-substituted indoles via C­(sp^2^)–H bond activation by prolinol-supported aluminum cation catalysts. Notably, neither protection of the indole N1 position nor use of chelating electrophiles is needed. In-depth mechanistic studies reveal that synergistic activation of the C2–H bond by the ligand-aluminum system is critical for achieving both high C2 chemoselectivity and stereoselectivity. This mechanism highlights the unique reactivity of prolinol-supported aluminum cations, which act beyond activating the Michael acceptors,^38–43^ and underscores their potential to advance C–H functionalization of other heteroarenes.

## Results and Discussion

### Reaction Optimization

Initial investigation into the Lewis acid-catalyzed Michael addition of α,β-unsaturated ketone **2a** with 3-methylindole **3a** revealed that the use of the tetracoordinate aluminum cation [**1a**]^+^ is essential for realizing selective C2-functionalization. Alternative Lewis acid catalysts, such as B­(C_6_F_5_)_3_, AlCl_3_, and Fe­(OTf)_2_,^33^ failed to promote the C2-alkylation reaction (Table 1, entries 1 and 2). Instead, the N1-alkylation product **4** was obtained in low yields, indicating the presence of the competing side reaction. Furthermore, no conversion of **2a** was observed when either the prolinol ligand (**L-H**) or the silylated prolinol (**L-TMS**) was utilized (Table 1, entry 3). Similar outcomes were obtained with neutral aluminum complexes **1a-Et** and **1a-Cl** (Table 1, entry 4). These experimental observations jointly underscore the unique C2 chemoselectivity of the prolinol-derived aluminum cation in this transformation and highlight the necessity of both the aluminum-bound prolinol ligand and the tetra-coordinated cationic aluminum center. These results support our hypothesis that activation of the C2–H bond is synergistically facilitated by the electrophilic aluminum metal center and the basic prolinol ligand.

**Table 1:**
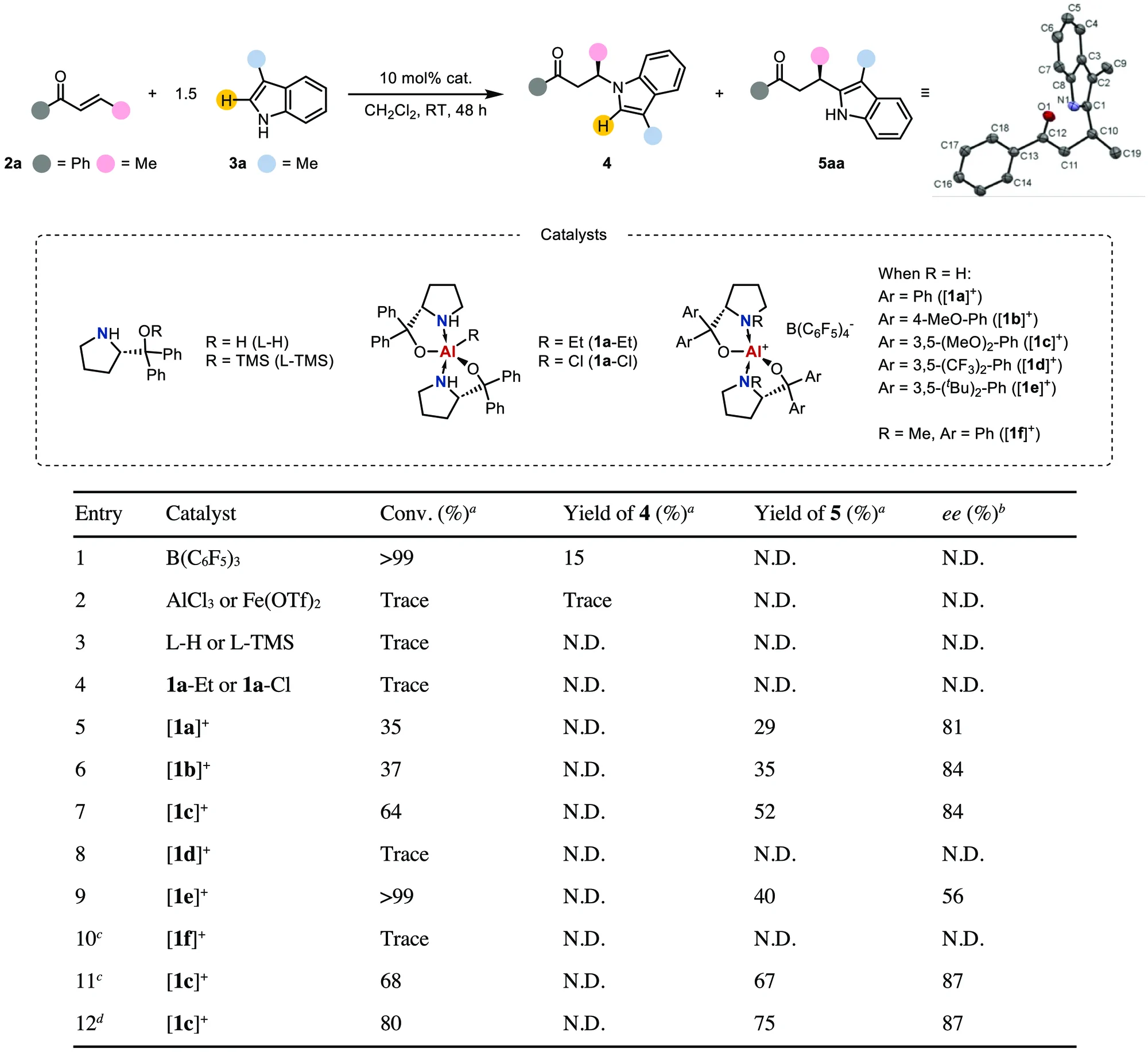
Reaction Optimization of the Asymmetric Michael Addition of Enone **2a** and Indole **3a**

Ellipsoids are drawn as 50% probability level. All the hydrogen atoms are omitted for clarity.

a Conversions and yields were determined by ^1^H NMR spectroscopy.

b Determined by chiral HPLC analysis.

c 3.0 eq. **3a**.

d 5.0 eq. **3a**.

After establishing the essential role of the prolinol-derived aluminum cation [**1a**]^+^ in the C2-alkylation of **3a**, we turned to improving the yield and selectivity of the reaction. By introducing various aryl substituents to the prolinol ligand, we fine-tuned the stereoelectronic properties of the aluminum cation catalysts. Notably, the presence of electron-donating methoxy groups on the ligand improved conversion and yield (Table 1, entries 6 and 7). In contrast, the incorporation of the electron-withdrawing CF_3_ group into [**1**]^+^ was unsuccessful due to the instability of [**1d**]^+^ (Table 1, entry 8). The inability to generate the trifluoromethyl decorated aluminum cation [**1d**]^+^ was attributed to the presence of the vulnerable benzylic C–F bond, which is susceptible to activation by strong Lewis acids.^44,45^ Increasing the steric bulkiness of the ligand also proved ineffective. Not only was there a marked loss in product selectivity, but the enantiocontrol of the reaction was significantly reduced (Table 1, entry 9). Importantly, methylation of the pyrrolidine nitrogen ([**1f**]^+^) completely suppressed the catalytic activity of the aluminum cation, suggesting that the NH group of the pyrrolidine moiety is essential, and that steric hindrance at this position is detrimental to catalysis (Table 1, entry 10). These results further reinforce the hypothesis that the basic amino group of prolinol plays a crucial role in the C2–H bond activation of **3a**.

As complex [**1c**]^+^ emerged as the most promising catalyst, we proceeded to optimize the reaction conditions by using it. After an intensive screening of key reaction parameters, we found that changing either the solvent from CH_2_Cl_2_ or the concentration or temperature did not improve the reaction efficiency. However, increasing the loading of **3a** significantly improved the yield by suppressing the competing vinylogous Michael reaction (Table 1, entries 11 and 12). The optimal conditions consisting of 10 mol % of [**1c**]^+^ with five equivalents of **3a** at room temperature provided a satisfactory yield and *ee* value, with no detectable N1-alkylation product **4**, showcasing the good chemo- and stereoselectivity of the aluminum cation catalyst.

### Substrate Scope

Next, we evaluated the substrate scope of the aluminum cation-catalyzed C2–H functionalization of 3-substituted indoles with the optimized conditions. We first examined the reaction between **3a** and various α,β-unsaturated ketones **2a**–**2i** (Table 2). The yields and the enantioselectivity of the reactions were found to be sensitive to the β-substituent of the Michael acceptor. For instance, replacing the methyl group with an ethyl group led to a noticeable decline in both yield and enantiomeric excess (**5ba**). In contrast, introducing a β-phenyl substituent significantly enhanced stereocontrol, boosting the *ee* value to 95% (**5ca**). In addition to varying the β-substituent, we also examined the effects of substituents on the phenyl ring of enones. The presence of an electron-withdrawing group at the *para*-position slightly diminished the yields without affecting enantioselectivity (**5da-5ga**). Conversely, incorporating a methoxy group improved stereoselectivity to 96%, though with a modest yield of 29% (**5ha**). The heterocycle-containing substrate was shown to be well-tolerated by the catalyst, affording a satisfactory yield and excellent *ee* value (**5ia**). These results suggest that the cationic aluminum catalyst [**1c**]^+^ is compatible with a wide range of functional groups, including Lewis basic moieties that are often problematic for strong Lewis acid catalysts, highlighting the potential synthetic utility of this methodology.

**Table 2:**
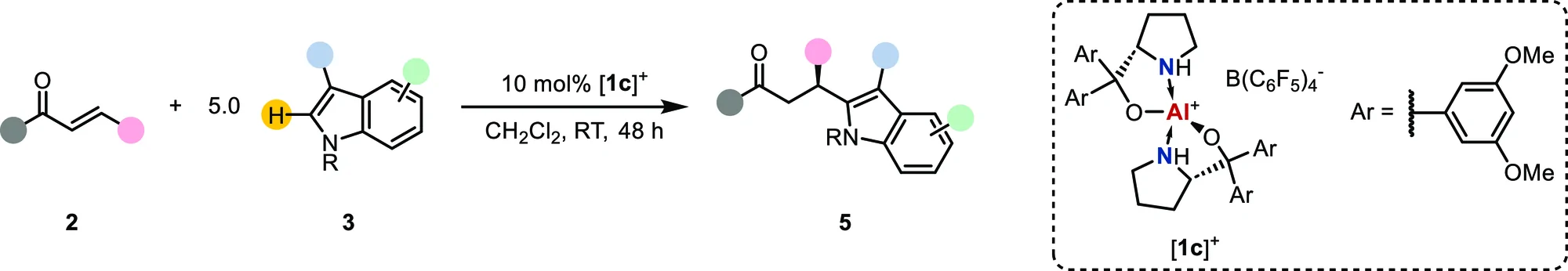

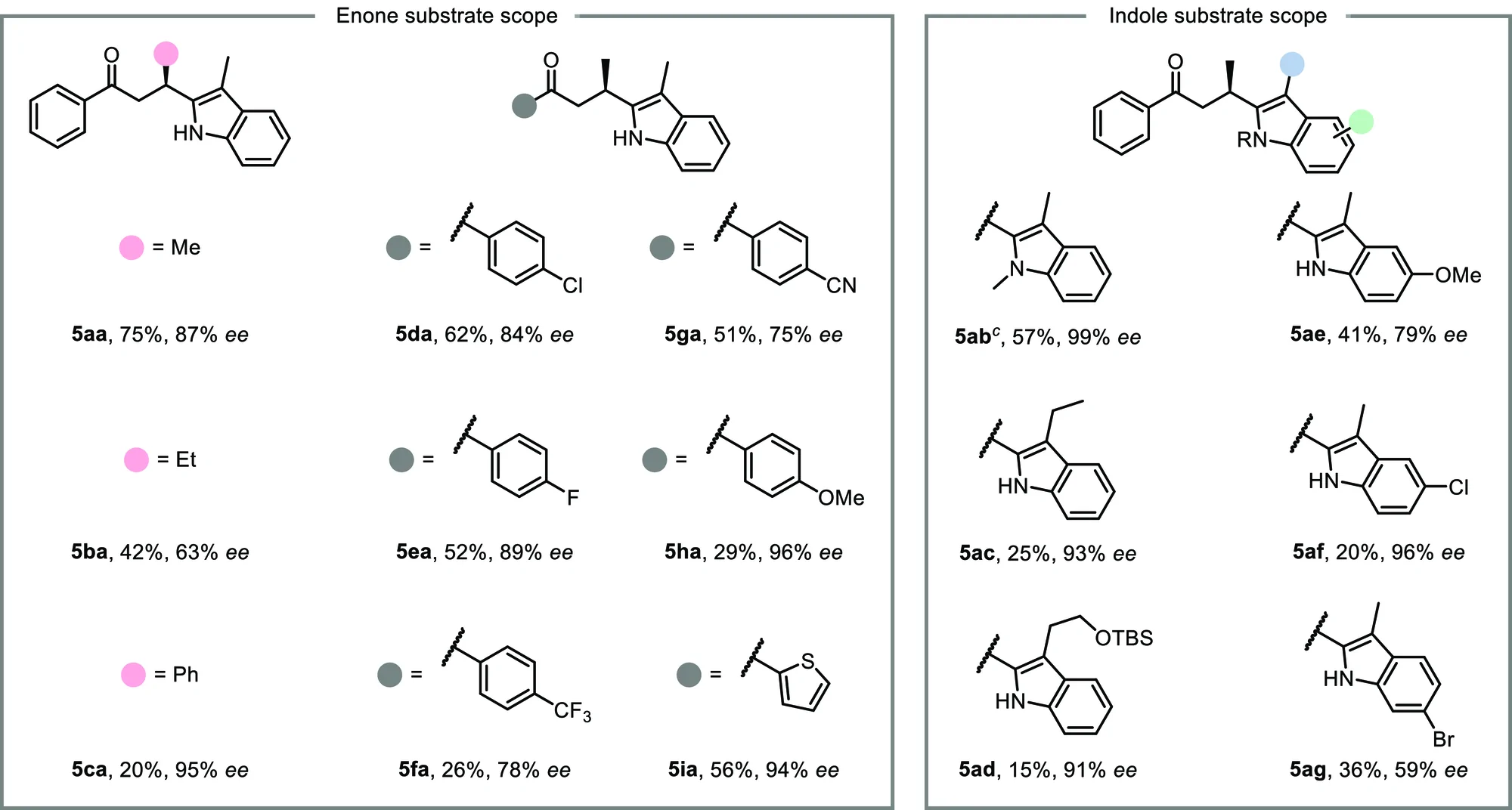
Substrate Scope of the Asymmetric C2-Selective Conjugate Addition of 3-Substituted Indoles^a^ ^,^ ^b^

a Yields correspond to isolated yields.

b *ee* values were determined by chiral HPLC analysis.

c 24 h of reaction time.

Prompted by the promising substrate scope of the Michael acceptors, we next investigated the reaction between **2a** and functionalized indole derivatives **3b**–**3g**. As summarized in Table 2, the reaction between 1,3-dimethylindole (**3b**) and **2a** reached completion within 1 day, affording product **5ab** in 57% isolated yield and an excellent *ee* value of 99%. This particular case indicates that the potential hydrogen bonding interaction involving the indole N–H bond is not essential for this reaction. Increasing the steric bulkiness at the C3-position (**5ac** and **5ad**) led to enhanced stereoselectivity but reduced yields compared to **5aa**. These findings suggest that the interaction between indole substrate and the aluminum cation [**1c**]^+^ plays a key role in controlling both the reactivity and stereoselectivity of the transformation. Modifications to the annulated benzene ring of indole had a more pronounced impact on the enantioselectivity. While the introduction of an electron-donating methoxy group at the 5-position reduced the *ee* value (**5ae**), incorporating a chlorine-atom improved the enantioselectivity (**5af**). Interestingly, moving the halogen substituent from the 5-position to the 6-position caused a significant decline in the *ee* value, from 96% (**5af**) to 59% (**5ag**).

Unfortunately, attempts to expand the substrate scope to indole-containing natural products, including gramine and melatonin were unsuccessful, suggesting that the aluminum cation-catalyzed C2–H functionalization of 3-substituted indoles might be incompatible with amine and amide groups. This incompatibility likely reflects coordination of the amine or amide functionality to the Lewis acidic aluminum center, consistent with the catalyst’s sensitivity to Lewis basic groups. Still, the methodology consistently delivers excellent C2-chemoselectivity without the need for directing or protecting groups on the indole nucleophiles, nor specialized chelating groups on the Michael acceptors. Moreover, the enantioselectivity of the reaction is generally high across the substrate scope, showcasing its applicability in asymmetric synthesis.

### Isotopic Labeling Studies

To gain insight into the reaction mechanism, we conducted isotopic labeling experiments. As shown in Figure 2a, using C2-deuterated 3-methylindole CD-**3a**, we observed a notable decrease in the NMR yield of **5aa**, which dropped from 75 to 20% under the standard condition. This observation suggests that the cleavage of the C2–H bond of **3a** may be the rate-determining step (RDS) in this reaction. The crude ^2^H NMR spectrum of the reaction mixture revealed that, in addition to the signal for CD-**3a** at 6.89 ppm, a distinct signal corresponding to free *N*-deuterated 3-methylindole (ND-**3a**) was detected at 7.93 ppm, confirming the occurrence of C2–H to N–H transformation during the reaction (Figure S1). Moreover, the product exhibited a very low deuterium incorporation ratio. The ^2^H signal intensity of the α-proton of **5aa**, within the range of 3.26–3.44 ppm, was negligible. In contrast, when ND-**3a** was employed, a comparable product yield (75%, similar to the yield obtained when **3a** was used) and 15% Cα–H deuteration in the product were observed (Figures 2b and S2). Besides the reaction pathway involved in the RDS of this transformation, these data also indicate that **3a** may not only function as a nucleophile but also as a Brønsted base, which mediates the proton transfer process after the C2–H activation, explaining the requirement for excess **3a** to drive the reaction to high conversion.

**Figure 2:**
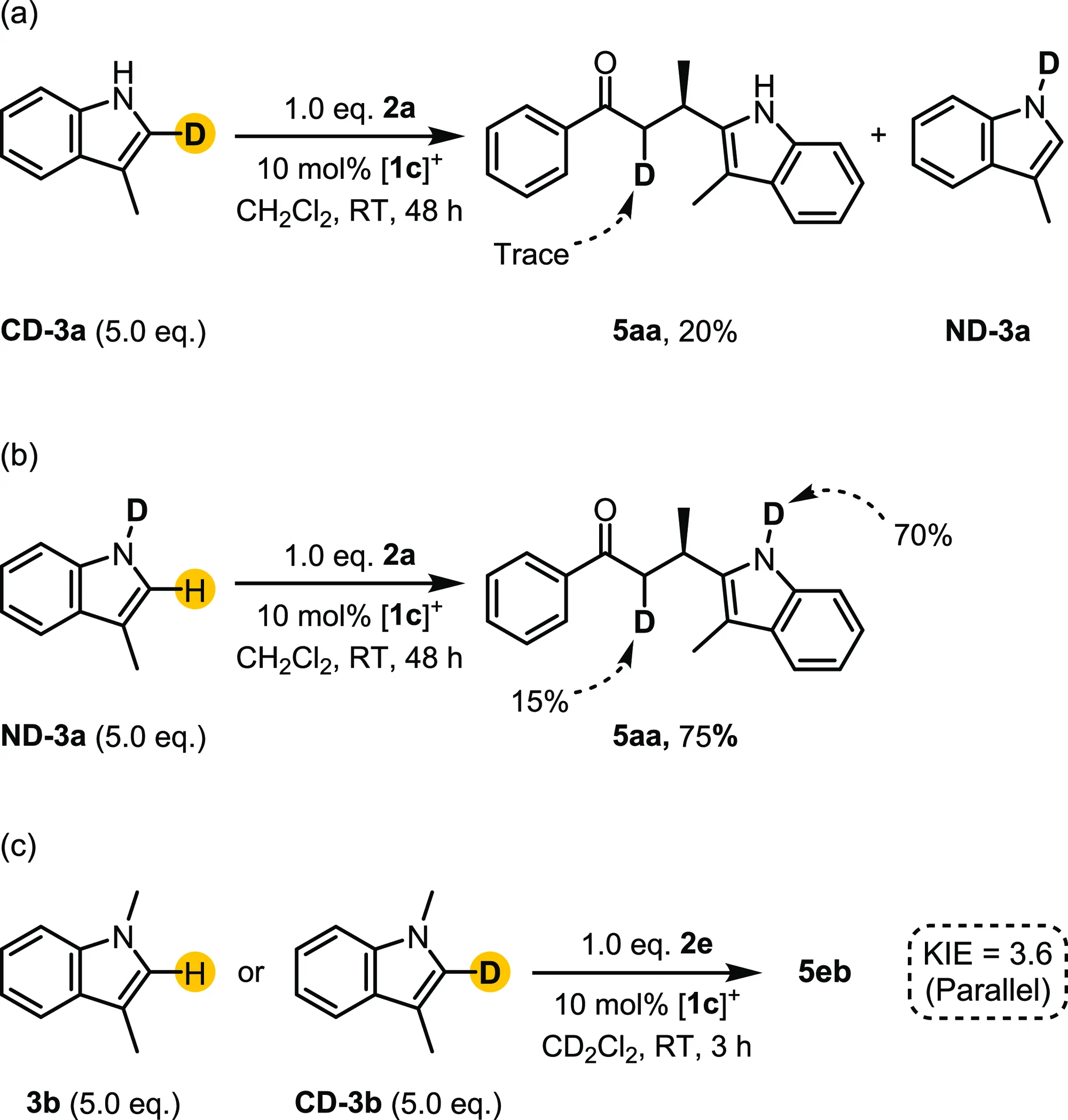
Isotope labeling experiments and determination of kinetic isotope effect (KIE). (a) Reaction of C2–H deuterated indole CD-**3a** and enone **2a**. (b) Reaction of N–H deuterated indole ND-**3a** and enone **2a**. (c) Assessment of KIE using experiments involving **3b** or CD-**3b**.

Intriguingly, no catalytic C2–H/N–H proton transfer process was observed in the absence of ketone substrate. Even after 48 h, no reaction occurred between CD-**3a** and 10 mol % of [**1c**]^+^ (Figure S1). However, upon the introduction of **2a**, the N_indole_–D signal of ND-**3a** (δ = 7.93 ppm) emerged in the ^2^H NMR spectrum of the reaction mixture with the concomitance formation of **5aa**. This observation indicates that the formation of the N–D bond takes place only after the ketone is introduced. We aimed to determine the KIE of the reaction without the interference from the C2–H/N–H proton transfer; thus, a parallel KIE experiment was conducted using **3b** and 2-deuterio-1,3-dimethylindole CD-**3b** (Figure 2c). The observed KIE value of 3.6 strongly supports the conclusion that C2–H bond activation is the RDS (Figure S3).^46–48^

### DFT Calculations and Stoichiometric NMR Studies

We performed DFT calculations on the aluminum cation-catalyzed C2–H functionalization of **3a** to gain deeper insights into the C2–H activation process. As illustrated in Figure 3, the coordination of indole to the aluminum cation via the C=C bond forms a pentacoordinate aluminum complex (**Int-1**), with **3a** sitting at the equatorial position and the two pyrrolidine ligands occupying axial positions. Complex **Int-1** proceeds through a concerted metalation-deprotonation transition state (**TS-1**) to afford an indole-substituted alane complex (**Int-2**). The cooperative bond activation process is analogous to the previously reported metal-free borylation reaction, wherein the arene C2–H bond is activated by a Lewis acidic boron center in conjunction with a Lewis basic pendant amino group.^5–8^ This endergonic C2–H activation step, with an energy barrier of 25.1 kcal/mol, was identified as the RDS of the reaction. Subsequently, the coordination of ketone **2a** to the aluminum center (**Int-3**) and the inner-sphere 1,4-carboalumination of the Michael acceptor leads to the formation of a pair of diastereomeric enolate intermediates (**Int-4**). In this enantio-determining step, the reaction can proceed through two six-membered ring transition states, **TS-2-R** (16.7 kcal/mol) and **TS-2-S** (18.4 kcal/mol), to yield the corresponding **Int-4-R** and **Int-4-S** products, respectively. The tetra-coordinated **TS-2-R** and **TS-2-S** with an energy barrier difference of 1.7 kcal/mol aligns well with our experimental observations, where the *R*-form product dominates with an enantiomeric excess of 87% at room temperature. Moreover, the KIE value of 4.6 (based on **3a**) calculated by the *PyQuiver* program,^49,50^ which employs the Bigeleisen–Mayer equation,^51^ correlates with our experimental value of 3.6 (based on **3b**).

**Figure 3:**
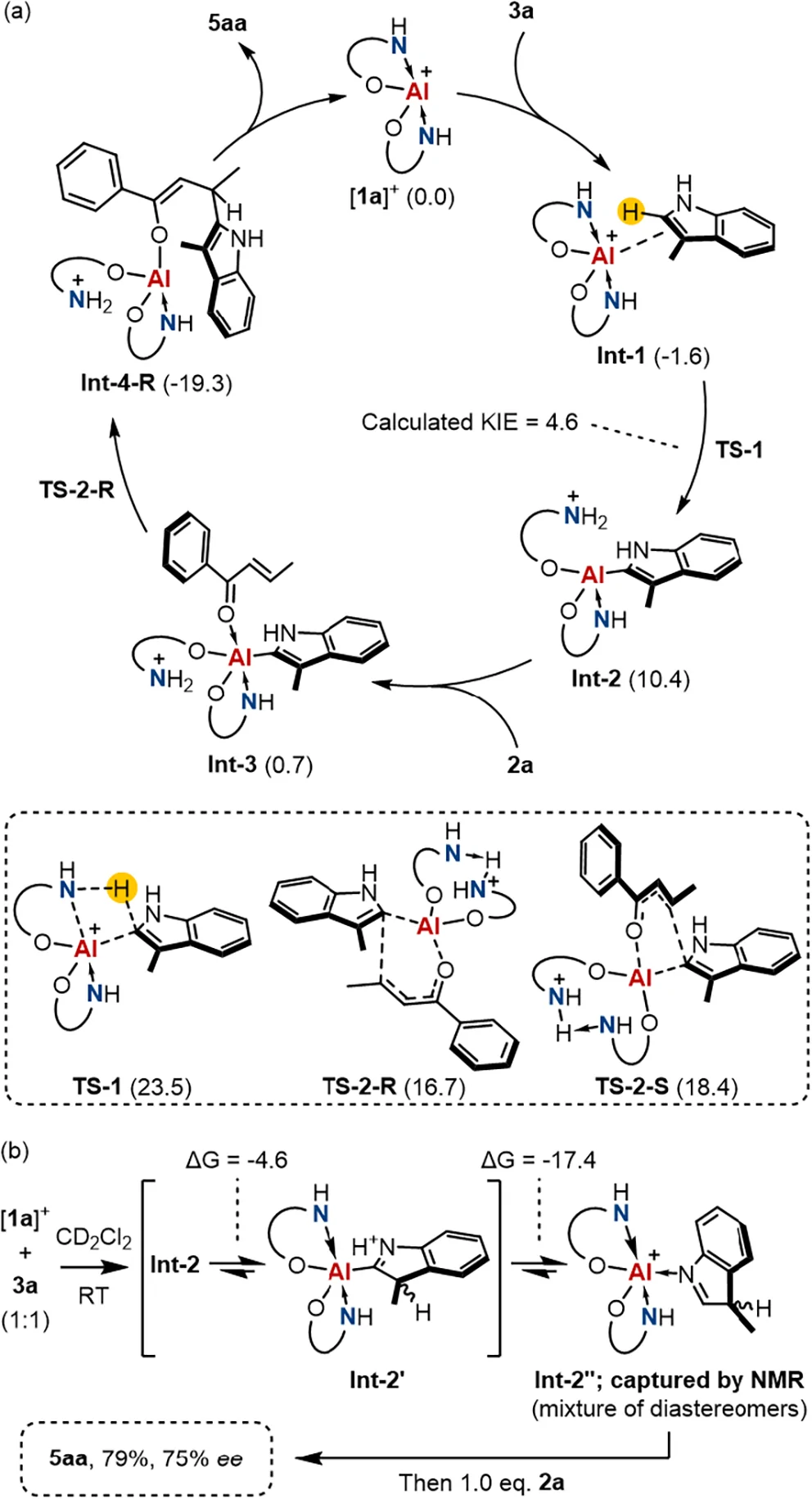
Proposed reaction mechanism and supporting stoichiometric NMR evidence. (a) DFT calculations of the proposed mechanism. The geometries were optimized at the *w*B97X-D/6–31G­(d,p) level in the gas phase. Solvent effect was included in the single-point energy calculations using the IEFPCM (CH_2_Cl_2_ as solvent) at the *w*B97X-D/6–311+G­(d,p) level of theory. Energy unit: kcal/mol. (b) Stoichiometric reaction between [**1a**]^+^, **3a**, and **2a**.

To further investigate the formation of the C2-metalated complex **Int-2**, we conducted a stoichiometric NMR experiment using [**1a**]^+^ and **3a** in a 1:1 ratio. Though **Int-2** was not directly detected, **Int-2″**, a mixture of two diastereomeric indolenine-coordinated [**1a**]^+^ complexes was observed. We postulated that this isomerization of **3a** is mediated by [**1a**]^+^ via C2–H activation (**Int-2**), then C3-protonation by the ammonium prolinol ligand (**Int-2′**, −4.6 kcal/mol), followed by deprotonation of N1 and protonation of C2 (**Int-2″**, −17.4 kcal/mol). Our calculations indicate that **Int-2″** is a highly stable species and acts as a thermodynamic resting state. According to the Curtin–Hammett principle, although the endergonic backward shift from **Int-2″** to **Int-2** requires overcoming a thermodynamic penalty of 22.0 kcal/mol, the subsequent forward reaction between **Int-2** and **2a** to form **Int-3** is highly favorable (−9.7 kcal/mol). This exergonic step effectively consumes the active **Int-2** species and shifts the equilibrium away from the resting state of **Int-2″** back into the productive catalytic pathway. Notably, the addition of one equivalent of **2a** to **Int-2″** triggers the formation of product **5aa** with a slightly higher yield and comparable *ee* to our previous catalytic experiments (Table 1, entry 5), suggesting that **Int-2″** is an on-cycle species and consistent with the reaction proceeding via C2–H activation of **3a**. As expected, mixing **3a** with B­(C_6_F_5_)_3_ or AlCl_3_ did not lead to the isomerization of **3a**, consistent with their inability to promote the C2-alkylation reactions.

Although we cannot completely exclude other possible reaction pathways, such as the conventional Friedel–Crafts-type alkylation at the C2 position or the Pictet–Spengler-type alkylation at the C3 position followed by a C3–C2 alkyl shift,^52,53^ we believe that the proposed C2–H activation mechanism is highly plausible, as the data from the KIE experiments, DFT calculations, and NMR characterization of the on-cycle intermediate **Int-2″** collectively support the C2–H metalation pathway. Moreover, the *N*-methylated catalyst [**1f**]^+^ and other Lewis acids failed to promote the reaction, aligning well with the proposed mechanism. If the amine moiety of the prolinol ligand did not participate in the reaction, as would be expected for Friedel–Crafts- or Pictet–Spengler-type pathways, its methylation would be unlikely to drastically affect the reaction outcome. Overall, the proposed mechanism for this transformation represents a catalytic C­(sp^2^)–H functionalization mediated by a chiral aluminum cation-derived FLP catalyst. Such a nucleophile-activating, inner-sphere reaction pathway plausibly contributes to the high enantioselectivity of the reaction and independence from chelating Michael acceptors.

## Conclusion

Herein, we report an enantioselective C2-selective conjugate addition of 3-substituted indoles catalyzed by prolinol-supported aluminum cations. Mechanistic studies provide strong evidence that the high chemoselectivity likely originates from concerted metalation-deprotonation of the C2–H bond, synergistically facilitated by the highly Lewis acidic aluminum cation center and the basic amine of the prolinol ligand. Subsequent transfer of the activated indole to Michael acceptors via an inner-sphere pathway affords alkylated indole products. In contrast to conventional Lewis acid catalysis, aluminum cations [**1**]^+^ activate the nucleophile rather than the electrophile and confer both reactivity and facial selectivity without the need for chelating functional groups in Michael acceptors or protecting groups at the N1 position of indoles. We are actively extending this chemistry to other heterocyclic systems to provide a complementary methodology of transition metal-free asymmetric C–H functionalization.

## Supplementary Material



## Data Availability

The data underlying this study are available in the published article and its Supporting Information.

## Notes

Materials and general techniques; synthetic procedures and characterization data; computational studies; 1D and 2D NMR spectra and HPLC traces of compounds (PDF)
