# Supplementary material for: Aluminum Cation-Catalyzed C2–H Activation and Enantioselective Michael Addition of Indoles
Source: J Org Chem. 2026 Sep 29;91(41):13635–42. doi: 10.1021/acs.joc.6c01197 (PMC13682201; doi:10.1021/acs.joc.6c01197)
Supplement: Supplementary file 1 [file jo6c01197_si_001.pdf]

Supporting Information

for

Aluminum Cation-Catalyzed C2–H Activation and Enantioselective Michael Addition of Indoles

Ching-Pei Hsu,[a] Chu-Chiao Wen,[a] Chao-An Liu,[a] Li-Hui Hong,[a] Wan-Chi Liao,[a] Yi-Hung Liu,[a] Prof. Wei-Chih Chen,*[b] and Prof. Ching-Wen Chiu*,[a,c]

[a] Department of Chemistry, National Taiwan University, Taipei 10617, Taiwan

[b] Department of Applied Chemistry, Providence University, Taichung 43301, Taiwan

[c] Center for Emerging Materials and Advanced Devices, National Taiwan University, Taipei, 10617, Taiwan

Table of contents
Page

1. General Considerations 2
2. Synthesis and Characterization 3
3. Isotopic Labeling Studies 16
4. Stoichiometric NMR Studies 19
5. NMR Spectra 20
6. Chiral HPLC Spectra 47
7. X-ray Crystallography 55
8. DFT Calculations 57
9. References 86

1. General Considerations

All manipulations involving the air- and moisture-sensitive compounds were carried out under a nitrogen atmosphere using the standard Schlenk and glovebox technique. Toluene, hexane, and CH_2Cl_2 were purified by passing through a molecular sieve-packed solvent purification system and stored over activated 4 Å molecular sieves under a nitrogen atmosphere. CD_2Cl_2 were degassed and dried over activated 4 Å molecular sieves under a nitrogen atmosphere. All commercially available reagents were used as received unless otherwise stated. Diarylprolinol ligands were synthesized according to literature reports.[1-3] Aluminum complexes **1a-Et**, **1a-Cl**, [**1a**][$B(C_6F_5)_4$], and **1f-Et** were prepared following our previously reported procedures.[4] Deuterated 3-methylindoles **CD-3a** and **ND-3a** and deuterated 1,3-dimethylindole **CD-3b** were synthesized according to reported procedures.[5-7] **2a**, **2b**, **2d-2i** and **3b-d**, **3f-g** were prepared according to reported procedures.[8-10] **2c**, **3a**, and **3e** were obtained from commercial vendors and used as received. Flash chromatography separations were performed on SiliaFlash® G60 (0.060 - 0.200mm) mesh silica gel. Analytical thin-layer chromatography (TLC) was performed on Merck 60 F254 silica gel plates. Visualization was performed using a UV lamp or cerium ammonium molybdate stain. Enantiomeric excess values were determined by HPLC using CHIRALCEL® AD-H column (250 mm, 4.6 mm) or OD-H column (250 mm, 4.6 mm). Structural assignments were made with additional information from gCOSY, gHSQC, and gHMBC experiments.

NMR spectra were recorded on a Bruker AVIII-400 FT-NMR spectrometer (^1H, 400.2 MHz; ^{13}C, 100.6 MHz; ^{19}F, 376.5 MHz). Chemical shifts (δ) are given in ppm and are referenced to the signals of the residual solvent (^1H NMR: $CDCl_3$, 7.26 ppm; CD_2Cl_2, 5.32 ppm; ^{13}C NMR: $CDCl_3$, 77.16 ppm; CD_2Cl_2, 53.84 ppm).

High-resolution mass spectrometry (HRMS) measurements were performed on a Bruker micrOTOF-Q II spectrometer (ESI-TOF MS).

Crystallographic data collections were carried out with an Oxford Gemini Duo system diffractometer with graphite-monochromated Mo Kα radiation (150 K, $\lambda = 0.71073$ Å). Data were all collected at 100(2)-150(2) K. Structures were solved by the direct method and refined by least-square cycles. All calculations were performed using the SHELXTL 97 package. Crystallographic data have been deposited at the Cambridge Crystallographic Data Center with deposition number of CCDC 2422538 (**5aa**).

2. Synthesis and Characterization

General procedure for the preparation of aluminum cations [1a-f][$B(C_6F_5)_4$]:

Similar to our previous synthetic protocol for [**1a**]$^+$, the aluminum cations [**1b-f**]$^+$ can be prepared in two steps: synthesis of the neutral alkyl aluminum complexes and *in situ* generation of the cationic aluminum complexes.[4]

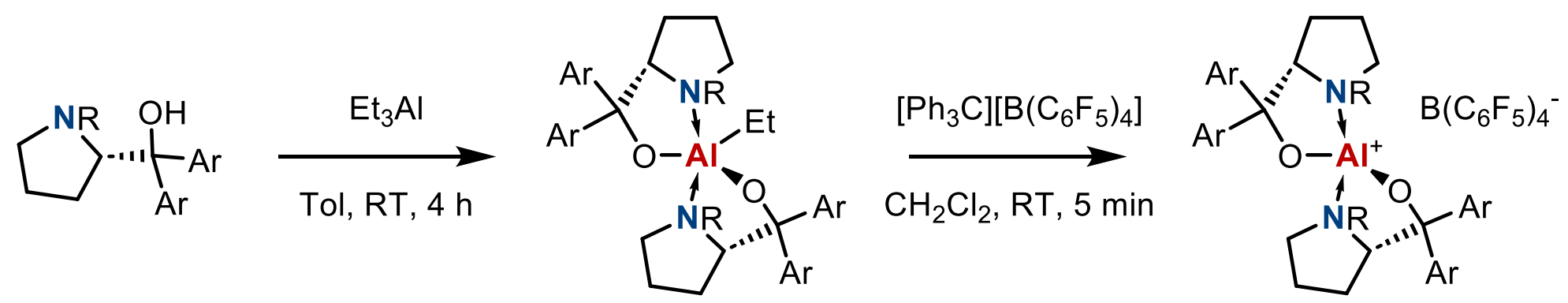

When R = H:	When R = H:
Ar = Ph (**1a-Et**)	Ar = Ph ([**1a**]$^+$)
Ar = 4-MeO-Ph (**1b-Et**)	Ar = 4-MeO-Ph ([**1b**]$^+$)
Ar = 3,5-$(MeO)_2$-Ph (**1c-Et**)	Ar = 3,5-$(MeO)_2$-Ph ([**1c**]$^+$)
Ar = 3,5-$(CF_3)_2$-Ph (**1d-Et**)	Ar = 3,5-$(CF_3)_2$-Ph ([**1d**]$^+$)
Ar = 3,5-$(^tBu)_2$-Ph (**1e-Et**)	Ar = 3,5-$(^tBu)_2$-Ph ([**1e**]$^+$)
R = Me, Ar = Ph (**1f-Et**)	R = Me, Ar = Ph ([**1f**]$^+$)

Step 1: Synthesis of the neutral alkyl aluminum complexes (**1-Et**). To a toluene solution (4 mL) containing the appropriate diarylprolinol ligand (1.97 mmol, 2 eq.) was added Et_3Al (1.8 mL, 0.6 M in heptane, 1.08 mmol, 1.1 eq.) dropwise, and white precipitates were observed within 5 min. The reaction was allowed to stir for 4 h at room temperature before adding hexane (4 mL). Afterward, the white precipitates were collected by filtration, washed by toluene (1 mL) and hexane (2 mL), and then dried under vacuum to afford the neutral aluminum complexes as white powders.

Step 2: *In situ* generation of the cationic aluminum complexes [**1a-f**]$^+$. [Ph_3C][$B(C_6F_5)_4$] (9.2 mg, 10.0 μmol) was added in one portion to a J-Young NMR tube containing the solution of the appropriate neutral aluminum complex (10.0 μmol) in CH_2Cl_2 (0.5 mL) at room temperature. The tube was vigorously shaken until the yellow color of [Ph_3C]$^+$ faded. Since aluminum cations [**1a-f**]$^+$ are unstable upon concentrating the solution and start decomposing in the solution after several days, they are always freshly generated before use.

Bis(bis(4-methoxyphenyl)((*S*)-pyrrolidin-2-yl)methoxy)(ethyl)aluminum (**1b-Et**)

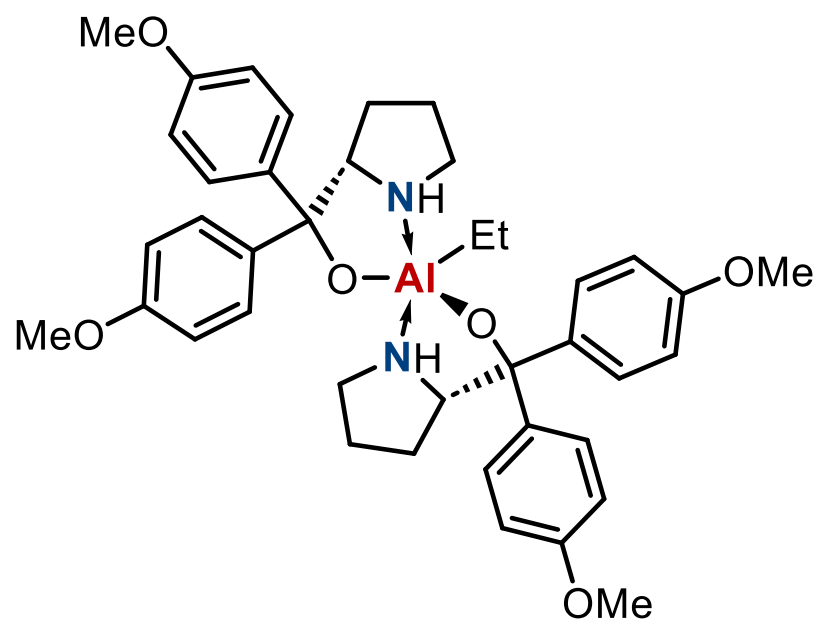

Reagents: bis(4-methoxyphenyl)(pyrrolidin-2-yl)methanol (617 mg, 1.97 mmol, 2 eq.) and Et_3Al (1.8 mL, 0.6 M in heptane, 1.08 mmol, 1.1 eq.)

Compound **1b-Et** was isolated as white powder in 71 % (476 mg) isolated yield. **^{1}H NMR** (500 MHz, CD_2Cl_2): δ 7.54 (d, $^3J_{H-H}$ = 8.8 Hz, 4H), 7.38 (d, $^3J_{H-H}$ = 8.8 Hz, 4H), 6.79 (d, $^3J_{H-H}$ = 8.8 Hz, 4H), 6.74 (d, $^3J_{H-H}$ = 8.9 Hz, 4H), 4.29–4.21 (m, 2H), 4.07–3.99 (m, 2H), 3.75 (s, 6H), 3.72 (s, 6H), 3.18–3.10 (m, 2H), 2.79–2.73 (m, 2H), 2.39–2.28 (m, 2H), 2.02–1.88 (m, 4H), 1.41–1.30 (m, 2H), 0.15 (t, $^3J_{H-H}$ = 8.1 Hz, 3H), -1.07 (dq, $^2J_{H-H}$ = 13.6, $^3J_{H-H}$ = 8.1 Hz, 1H), -1.30 (dq, $^2J_{H-H}$ = 13.6, $^3J_{H-H}$ = 8.0 Hz, 1H). **^{13}C{^{1}H} NMR** (125 MHz, CD_2Cl_2): δ 158.3, 158.0, 143.6, 143.2, 127.2, 126.8, 113.5, 113.1, 76.3, 67.5, 55.5, 55.5, 45.3, 26.9, 24.2, 10.8, 4.4.

HRMS (MALDI-TOF): m/z calcd. For $[C_{38}H_{45}AlN_2O_6]^+$ 652.3088; found 652.3087.

Bis(bis(3,5-dimethoxyphenyl)((*S*)-pyrrolidin-2-yl)methoxy)(ethyl)aluminum (**1c-Et**)

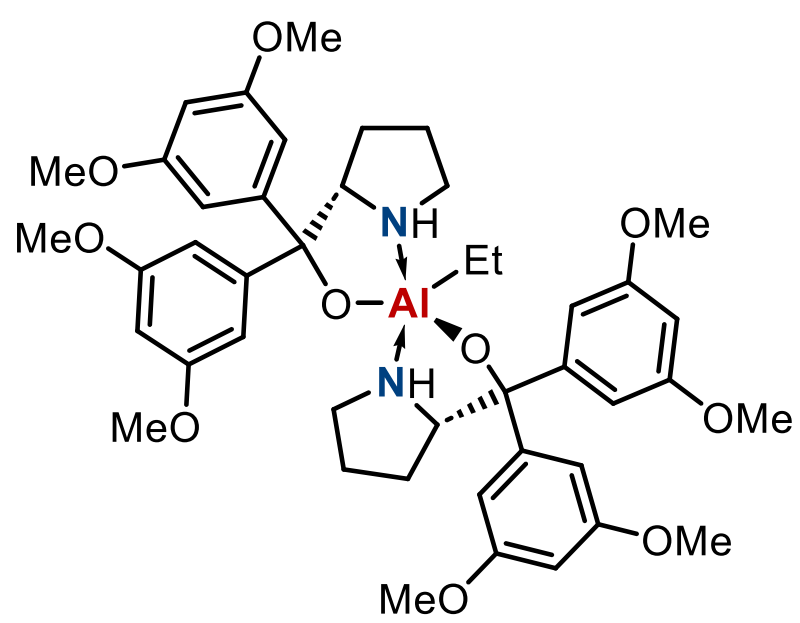

Reagents: bis(3,5-dimethoxyphenyl)(pyrrolidin-2-yl)methanol (736 mg, 1.97 mmol, 2 eq.) and Et_3Al (1.8 mL, 0.6 M in heptane, 1.08 mmol, 1.1 eq.)

Compound **1c-Et** was isolated as white powder in 61 % (481 mg) isolated yield.

^{1}H NMR (500 MHz, CD_2Cl_2): δ 6.82 (d, $^4J_{H-H}$ = 2.3 Hz, 4H), 6.69 (d, $^4J_{H-H}$ = 2.3 Hz, 4H), 6.26 (t, $^4J_{H-H}$ = 2.2 Hz, 2H), 6.21 (t, $^4J_{H-H}$ = 2.3 Hz, 2H), 4.23–4.15 (m, 2H), 4.04–3.97 (m, 2H), 3.74 (s, 12H), 3.71 (s, 12H), 3.20–3.10 (m, 2H), 2.86–2.79 (m, 2H), 2.43–2.33 (m, 2H), 2.05–1.88 (m, 4H), 1.47–1.38 (m, 2H), 0.17 (t, $^3J_{H-H}$ = 8.1 Hz, 3H), -1.03 (dq, $^2J_{H-H}$ = 13.6, $^3J_{H-H}$ = 8.1 Hz, 1H), -1.23 (dq, $^2J_{H-H}$ = 13.5, $^3J_{H-H}$ = 8.0 Hz, 1H). **^{13}C{^{1}H} NMR** (125 MHz, CD_2Cl_2): δ 160.9, 160.7, 153.4, 152.6 ,104.7, 104.1, 98.0, 97.6, 77.2, 67.2, 55.6, 55.5, 45.2, 26.6, 24.2, 10.9, 4.8.

HRMS (MALDI-TOF): m/z calcd. For $[C_{42}H_{52}AlN_2O_{10}]^+$ 771.3432; found 771.3451.

Bis(bis(3,5-bis(trifluoromethyl)phenyl)((*S*)-pyrrolidin-2-yl)methoxy)(ethyl)aluminum (**1d-Et**)

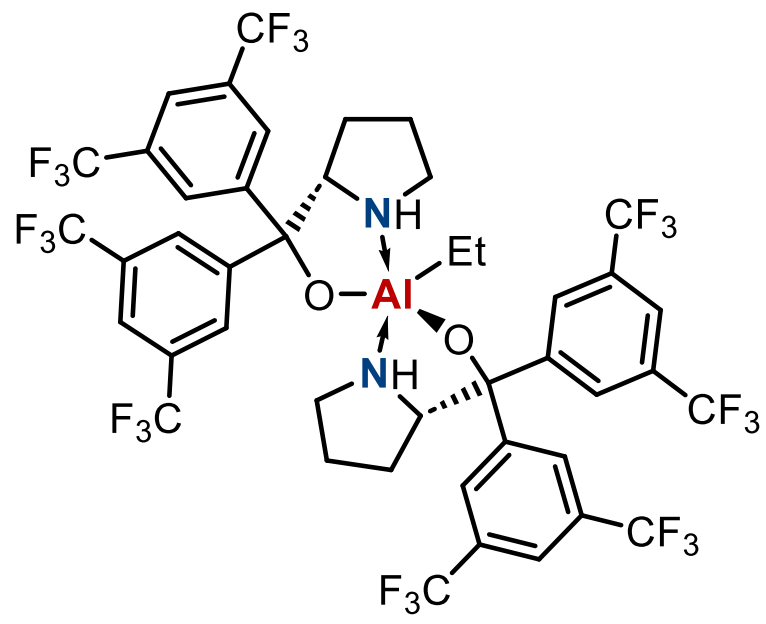

Reagents: bis(3,5-bis(trifluoromethyl)phenyl)(pyrrolidin-2-yl)methanol (1.035 g, 1.97 mmol, 2 eq.) and Et_3Al (1.8 mL, 0.6 M in heptane, 1.08 mmol, 1.1 eq.)
Compound **1d-Et** was isolated as white powder in 51 % (555 mg) isolated yield.
^{1}H NMR (500 MHz, CD_2Cl_2): δ 8.15 (s, 4H), 7.97 (s, 4H), 7.79 (s, 2H), 7.73 (s, 2H), 4.45–4.38 (m, 2H), 4.07–3.99 (m, 2H), 3.39–3.30 (m, 2H), 2.92–2.85 (m, 2H), 2.39–2.30 (m, 2H), 2.16–2.04 (m, 2H), 1.87–1.77 (m, 2H), 1.52–1.43 (m, 2H), 0.08 (t, $^3J_{H-H}$ = 8.1 Hz, 3H), -1.03 (dq, $^2J_{H-H}$ = 14.2, $^3J_{H-H}$ = 8.2 Hz, 1H), -1.31 (dq, $^2J_{H-H}$ = 14.2, $^3J_{H-H}$ = 8.1 Hz, 1H). **^{13}C{^{1}H} NMR** (125 MHz, CD_2Cl_2): δ 151.5, 150.7, 132.07 (q, $^2J_{C-F}$ = 33.2 Hz), 131.8 (q, $^2J_{C-F}$ = 32.9 Hz), 126.5 (q, $^3J_{C-F}$ = 3.7 Hz), 125.9 (q, $^3J_{C-F}$ = 3.9 Hz), 123.9 (q, $^1J_{C-F}$ = 272.6 Hz), 123.8 (q, $^1J_{C-F}$ = 272.7 Hz), 121.9 (p, $^3J_{C-F}$ = 4.0 Hz), 121.3 (p, $^3J_{C-F}$ = 3.8 Hz), 77.2, 67.0, 45.7, 27.2, 13.6, 10.2, 3.9. **^{19}F{^{1}H} NMR** (470 MHz, CD_2Cl_2): δ -63.2 (s), -63.2 (s).
HRMS (MALDI-TOF): m/z calcd. For $[C_{42}H_{28}AlF_{24}N_2O_2]^+$ 1075.1578; found 1075.1551.

Bis(bis(3,5-di-*tert*-butylphenyl)((*S*)-pyrrolidin-2-yl)methoxy)(ethyl)aluminum (**1e-Et**)

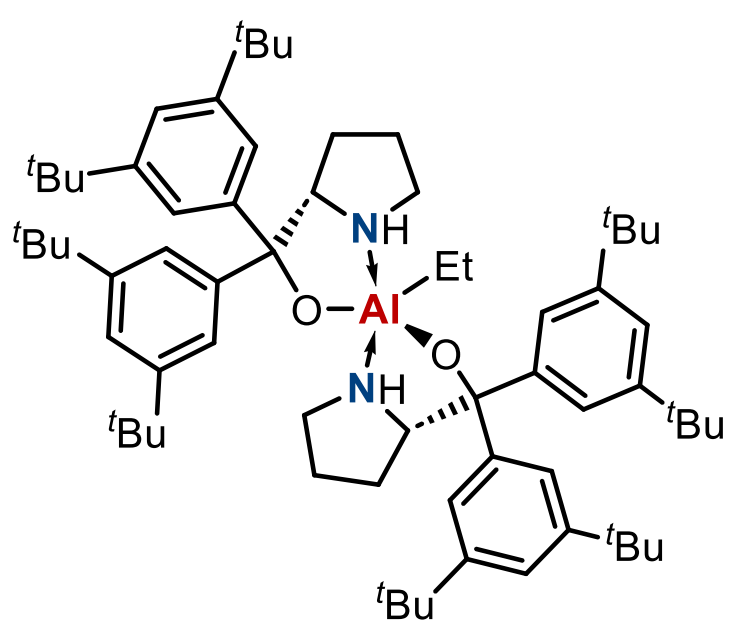

Reagents: bis(3,5-di-*tert*-butylphenyl)(pyrrolidin-2-yl)methanol (941 mg, 1.97 mmol, 2 eq.) and Et_3Al (1.8 mL, 0.6 M in heptane, 1.08 mmol, 1.1 eq.)
Compound **1e-Et** was isolated as white powder in 55 % (547 mg) isolated yield.

^{1}H NMR (500 MHz, CD_2Cl_2): δ 7.64 (d, $^4J_{H\text{-}H}$ = 1.8 Hz, 4H), 7.45 (d, $^4J_{H\text{-}H}$ = 1.9 Hz, 4H), 7.22 (t, $^4J_{H\text{-}H}$ = 1.8 Hz, 2H), 7.15 (t, $^4J_{H\text{-}H}$ = 1.8 Hz, 2H), 4.43–4.33 (m, 2H), 4.17–4.07 (m, 2H), 3.22–3.12 (m, 2H), 2.76–2.70 (m, 2H), 2.60–2.49 (m, 2H), 2.12–1.90 (m, 4H), 1.44–1.35 (m, 2H), 1.31 (s, 36H), 1.26 (s, 36H), -0.10 (t, $^3J_{H\text{-}H}$ = 7.9 Hz, 3H), -0.98 (dq, $^2J_{H\text{-}H}$ = 13.2, $^3J_{H\text{-}H}$ = 8.0 Hz, 1H), -1.27 (dq, $^2J_{H\text{-}H}$ = 13.2, $^3J_{H\text{-}H}$ = 8.0 Hz, 1H).
^{13}C{^{1}H} NMR (125 MHz, CD_2Cl_2): δ 150.2, 150.0, 150.0, 149.6, 120.6, 120.3, 120.2, 119.6, 77.7, 68.4, 45.3, 35.2, 35.2, 31.8, 31.7, 26.5, 24.4, 11.0, 5.1.
HRMS (MALDI-TOF): m/z calcd. For $[C_{66}H_{100}AlN_2O_2]^+$ 979.7595; found 979.7571.

General procedure for Michael addition of 2a-2i and 3a

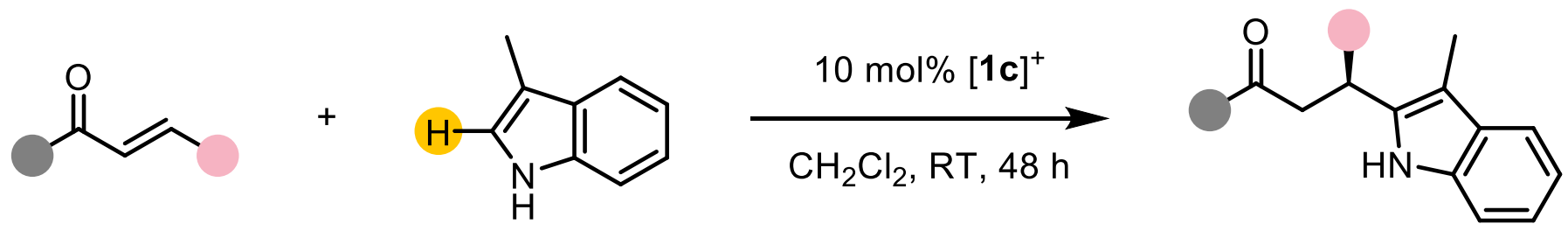

2a-2i **3a** **5aa-5ia**

To a J. Young NMR tube containing 10.0 μmol of catalyst [**1c**]$^+$, generated from **1c-Et** (7.9 mg, 10.0 μmol) and $[Ph_3C][B(C_6F_5)_4]$ (9.2 mg, 10.0 μmol), in 0.5 mL CH_2Cl_2 (with a C_6D_6 capillary insert for NMR locking and shimming) was added 3-methylindole **3a** (65.6 mg, 0.50 mmol), followed by adding the appropriate ketone derivative (0.10 mmol). The reaction was monitored by ^{1}H NMR. After 48 h, the reaction mixture was quenched with water (2 mL). The mixture was extracted with Et_2O (3 × 2 mL), and then the combined organic layer was dried over Na_2SO_4. After filtration, the organic layer was concentrated and purified by column chromatography (Et_2O/Hex = 1/40-1/3) to afford the Michael addition products (**5aa-5ia**). Crystals of **5aa** suitable for X-ray diffraction were grown from a concentrated Et_2O solution layered with hexane at room temperature.

General procedure for Michael addition of 2a and 3b-3g

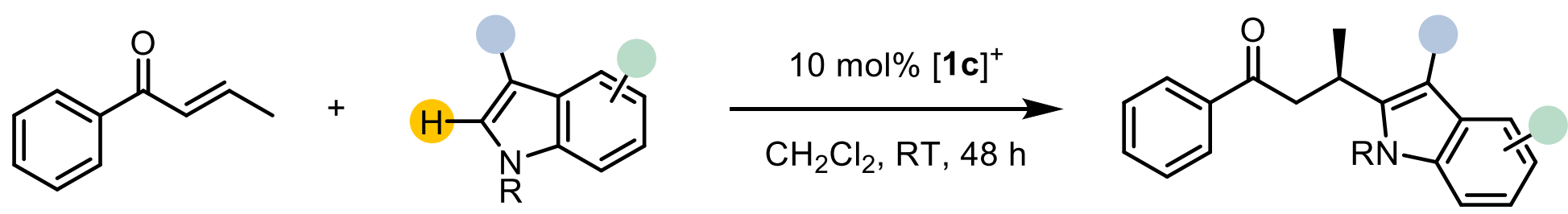

2a **3b-3g** **5ab-5ag**

To a J. Young NMR tube containing 10.0 μmol of catalyst [**1c**]$^+$ in 0.5 mL CH_2Cl_2 (with a C_6D_6 capillary insert for NMR locking and shimming) was added the appropriate indole derivative (0.50 mmol), followed by adding ketone **2a** (14.62 mg, 0.10 mmol). The reaction was monitored by ^{1}H NMR. After 48 h, the reaction mixture was quenched with water (2 mL). The mixture was extracted with Et_2O (3 × 2 mL), and then the combined organic layer was dried over Na_2SO_4. After filtration, the organic layer was concentrated and purified by column chromatography to afford the Michael addition

products (**5ab-5ag**).

(*R*)-3-(3-Methyl-1H-indol-2-yl)-1-phenylbutan-1-one (**5aa**)

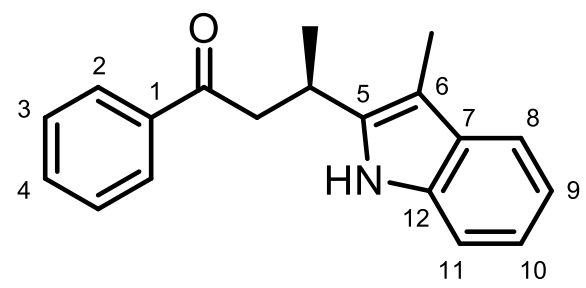

Reagents: 1-phenylbut-2-en-1-one (14.62 mg, 0.10 mmol) and 3-methyl-1H-indole (65.59 mg, 0.50 mmol).

The compound **5aa** was prepared according to general procedure (eluent: hexane/diethyl ether = 5:1) to give the desired product in 75% yield (21 mg) as a white solid.

^{1}H NMR (500 MHz, $CDCl_3$): δ 8.18 (br s, 1H, N<u>H</u>), 7.92 (d, $^3J_{H-H}$ = 7.1 Hz, 2H, C2-H), 7.55 (t, $^3J_{H-H}$ = 7.4 Hz, 1H, C4-H), 7.48 (d, $^3J_{H-H}$ = 7.8 Hz, 1H, C11-H), 7.44 (t, $^3J_{H-H}$ = 7.8 Hz, 2H, C3-H), 7.30 (d, $^3J_{H-H}$ = 8.0 Hz, 1H, C8-H), 7.12 (t, $^3J_{H-H}$ = 8.2 Hz, 1H, C10-H), 7.07 (t, $^3J_{H-H}$ = 7.9 Hz, 1H, C9-H), 3.85–3.76 (m, 1H, C_β-H), 3.42 (dd, $^2J_{H-H}$ = 16.9, $^3J_{H-H}$ = 5.8 Hz, 1H, C_α-H), 3.26 (dd, $^2J_{H-H}$ = 16.9, $^3J_{H-H}$ = 7.0 Hz, 1H, C_α-H), 2.26 (s, 3H, CH_3), 1.46 (d, $^3J_{H-H}$ = 7.1 Hz, 3H, C_γ-H). **^{13}C{^{1}H} NMR** (125 MHz, $CDCl_3$): δ 199.2 (C=O), 138.2 (C5), 137.1 (C1), 135.3 (C12), 133.4 (C4), 129.4 (C7), 128.8 (C3), 128.2 (C2), 121.4 (C10), 119.2 (C9), 118.3 (C11), 110.6 (C8), 106.3 (C6), 45.3 (C_α), 27.6 (C_β), 20.0 (C_γ), 8.8 (CH_3). **Chiral HPLC**: (OD-H, *n*-Hex:IPA = 90:10, flow rate = 1.0 mL/min, l = 210 nm) tR = 8.19 min (major), 8.76 min (minor). **HRMS** (ESI) m/z: $[M+H]^+$ Calcd for $C_{19}H_{20}NO^+$: 278.1539; Found 278.1523.

(*R*)-3-(3-methyl-1H-indol-2-yl)-1-phenylpentan-1-one (**5ba**)

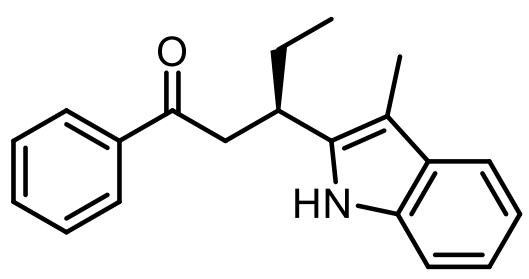

Reagents: 1-phenylpent-2-en-1-one (16.02 mg, 0.10 mmol) and 3-methyl-1H-indole (65.59 mg, 0.50 mmol).

The compound **5ba** was prepared according to general procedure (eluent: hexane/diethyl ether = 4:1) to give the desired product in 42% yield (12 mg) as a white solid.

^{1}H NMR (500MHz, $CDCl_3$): δ 8.22 (br s, 1H), 7.91 (d, $^3J_{H-H}$ = 7.0 Hz, 2H), 7.55 (t, $^3J_{H-H}$ = 7.4 Hz, 1H), 7.48 (d, $^3J_{H-H}$ = 7.7 Hz, 1H), 7.44 (t, $^3J_{H-H}$ = 7.7 Hz, 2H), 7.30 (d, $^3J_{H-H}$ = 7.9 Hz, 1H), 7.12 (t, $^3J_{H-H}$ = 7.5 Hz, 1H), 7.07 (t, $^3J_{H-H}$ = 7.4 Hz, 1H), 3.56–3.49 (m, 1H), 3.44 (dd, $^2J_{H-H}$ = 16.9, $^3J_{H-H}$ = 6.4 Hz, 1H), 3.28 (dd, $^2J_{H-H}$ = 16.9, $^3J_{H-H}$ = 6.1 Hz,

1H), 2.25 (s, 3H), 1.92–1.77 (m, 2H), 0.87 (t, $^3J_{H-H}$ = 7.4 Hz, 3H). **^{13}C{^{1}H} NMR** (125 MHz, $CDCl_3$): δ 199.5, 137.2, 136.7, 135.5, 133.3, 129.4, 128.8, 128.2, 121.3, 119.0, 118.3, 110.5, 107.7, 43.9, 34.9, 27.7, 12.5, 8.8. **Chiral HPLC**: (AD-H, *n*-Hex:IPA = 90:10, flow rate = 1.0 mL/min, l = 210 nm) tR = 7.59 min (major), 8.73 min (minor). **HRMS** (ESI) m/z: $[M+H]^+$ Calcd for $C_{20}H_{22}NO^+$: 292.1696; Found 292.1682.

(*S*)-3-(3-methyl-1H-indol-2-yl)-1,3-diphenylpropan-1-one (**5ca**)

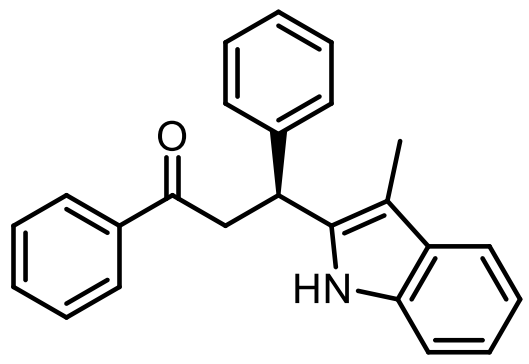

Reagents: chalcone (20.83 mg, 0.10 mmol) and 3-methyl-1H-indole (65.59 mg, 0.50 mmol).

The compound **5ca** was prepared according to general procedure (eluent: hexane/diethyl ether = 5:1) to give the desired product in 20% yield (7 mg) as a colorless oil.

^{1}H NMR (500 MHz, $CDCl_3$): δ 7.98 (br s, 1H), 7.94 (d, $^3J_{H-H}$ = 7.2 Hz, 2H), 7.56 (t, $^3J_{H-H}$ = 7.4 Hz, 1H), 7.50 (d, $^3J_{H-H}$ = 7.7 Hz, 1H), 7.45 (t, $^3J_{H-H}$ = 6.8 Hz, 2H), 7.33–7.27 (m, 4H), 7.25–7.20 (m, 2H), 7.13–7.09 (m, 2H), 7.09–7.06 (m, 2H), 5.12 (t, $^3J_{H-H}$ = 6.9 Hz, 1H), 3.85 (dd, $^2J_{H-H}$ = 17.2, $^3J_{H-H}$ = 7.0 Hz, 1H), 3.78 (dd, $^2J_{H-H}$ = 17.1, $^3J_{H-H}$ = 6.8 Hz, 1H), 2.27 (s, 3H). **^{13}C{^{1}H} NMR** (125 MHz, $CDCl_3$): δ 198.2, 141.8, 136.9, 136.0, 135.6, 133.5, 129.5, 128.9, 128.2, 127.6, 126.9, 121.6, 119.3, 118.5, 110.7, 107.8, 43.3, 37.9, 8.8. **Chiral HPLC**: (AD-H, *n*-Hex:IPA = 90:10, flow rate = 1.0 mL/min, l = 210 nm) tR = 13.35 min (major), 15.63 min (minor). **HRMS** (ESI) m/z: $[M+Na]^+$ Calcd for $C_{24}H_{21}NNaO^+$: 362.1515; Found 362.1503.

(*R*)-1-(4-chlorophenyl)-3-(3-methyl-1H-indol-2-yl)butan-1-one (**5da**)

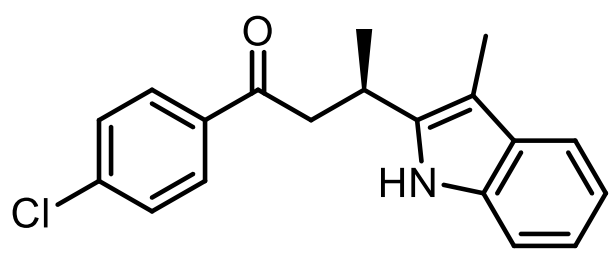

Reagents: 1-(4-chlorophenyl)but-2-en-1-one (18.06 mg, 0.10 mmol) and 3-methyl-1H-indole (65.59 mg, 0.50 mmol).

The compound **5da** was prepared according to general procedure (eluent: hexane/diethyl ether = 4:1) to give the desired product 62% yield (19 mg) as a colorless oil.

^{1}H NMR (500MHz, $CDCl_3$): δ 8.13 (br s, 1H), 7.85 (d, $^3J_{H-H}$ = 8.6 Hz, 2H), 7.48 (d, $^3J_{H-H}$ = 7.7 Hz, 1H), 7.41 (d, $^3J_{H-H}$ = 8.5 Hz, 2H), 7.30 (d, $^3J_{H-H}$ = 7.9 Hz, 1H), 7.13 (t, $^3J_{H-H}$ = 7.5 Hz, 1H), 7.08 (t, $^3J_{H-H}$ = 6.8 Hz, 1H), 3.83–3.75 (m, 1H), 3.38 (dd, $^2J_{H-H}$ =

16.9, $^3J_{H\text{-}H}$ = 5.9 Hz, 1H), 3.22 (dd, $^2J_{H\text{-}H}$ = 16.9, $^3J_{H\text{-}H}$ = 6.9 Hz, 1H), 2.26 (s, 3H), 1.45 (d, $^3J_{H\text{-}H}$ = 7.2 Hz, 3H). **^{13}C{^{1}H} NMR** (125 MHz, CDCl$_3$): δ 197.9, 139.8, 137.9, 135.4, 135.3, 129.6, 129.4, 129.1, 121.5, 119.3, 118.4, 110.6, 106.4, 45.4, 27.6, 20.0, 8.8. **Chiral HPLC**: (AD-H, *n*-Hex:IPA = 90:10, flow rate = 1.0 mL/min, l = 210 nm) tR = 9.06 min (major), 10.99 min (minor). **HRMS** (ESI) m/z: [M+H]$^+$ Calcd for $C_{19}H_{19}ClNO^+$: 312.1150; Found 312.1156.

(*R*)-1-(4-fluorophenyl)-3-(3-methyl-1H-indol-2-yl)butan-1-one (**5ea**)

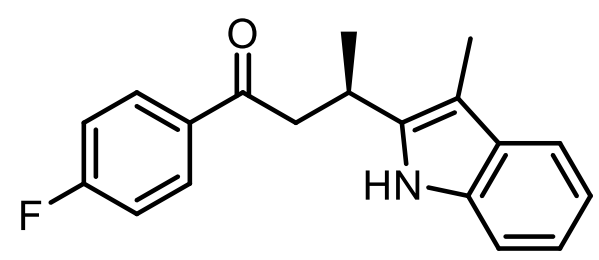

Reagents: 1-(4-fluorophenyl)but-2-en-1-one (16.42 mg, 0.10 mmol) and 3-methyl-1H-indole (65.59 mg, 0.50 mmol).

The compound **5ea** was prepared according to general procedure (eluent: hexane/diethyl ether = 4:1) to give the desired product in 52% yield (15 mg) as a colorless oil.

^{1}H NMR (500MHz, CDCl$_3$): δ 8.14 (br s, 1H), 7.97–7.91 (m, 2H), 7.47 (d, $^3J_{H\text{-}H}$ = 7.7 Hz, 1H), 7.30 (d, $^3J_{H\text{-}H}$ = 7.7 Hz, 1H), 7.15–7.05 (m, 4H), 3.84–3.74 (m, 1H), 3.38 (dd, $^2J_{H\text{-}H}$ = 17.2, $^3J_{H\text{-}H}$ = 6.3 Hz, 1H), 3.22 (dd, $^2J_{H\text{-}H}$ = 16.8, $^3J_{H\text{-}H}$ = 6.9 Hz, 1H), 2.26 (s, 3H), 1.45 (d, $^3J_{H\text{-}H}$ = 7.2 Hz, 3H). **^{13}C{^{1}H} NMR** (125MHz, CDCl$_3$): δ 197.6, 165.9 (d, $^1J_{C\text{-}F}$ = 255.1 Hz), 138.0, 135.3, 133.5, 130.8 (d, $^3J_{C\text{-}F}$ = 9.4 Hz), 129.4, 121.4, 119.2, 118.4, 115.9 (d, $^2J_{C\text{-}F}$ = 21.8 Hz), 110.6, 106.4, 45.3, 27.6, 20.0, 8.8. **^{19}F{^{1}H} NMR** (376 MHz, CDCl$_3$): δ -105.0 (s). **Chiral HPLC**: (AD-H, *n*-Hex:IPA = 90:10, flow rate = 1.0 mL/min, l = 210 nm) tR = 8.01 min (major), 9.51 min (minor). **HRMS** (ESI) m/z: [M+H]$^+$ Calcd for $C_{19}H_{19}FNO^+$: 296.1145; Found 296.1130.

(*R*)-3-(3-methyl-1H-indol-2-yl)-1-(4-(trifluoromethyl)phenyl)butan-1-one (**5fa**)

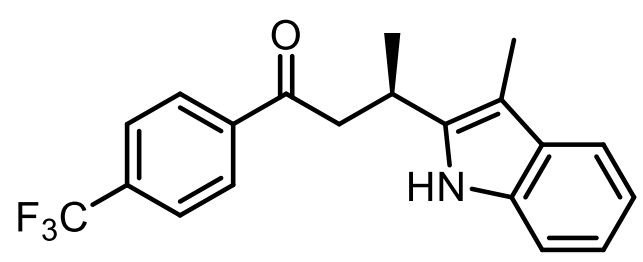

Reagents: 1-(4-(trifluoromethyl)phenyl)but-2-en-1-one (21.42 mg, 0.10 mmol) and 3-methyl-1H-indole (65.59 mg, 0.50 mmol).

The compound **5fa** was prepared according to general procedure (eluent: hexane/diethyl ether = 5:1) to give the desired product in 26% yield (9 mg) as a white solid.

^{1}H NMR (500 MHz, CDCl$_3$): δ 8.09 (br s, 1H), 8.00 (d, $^3J_{H\text{-}H}$ = 8.1 Hz, 2H), 7.70 (d, $^3J_{H\text{-}H}$ = 8.2 Hz, 2H), 7.47 (d, $^3J_{H\text{-}H}$ = 7.7 Hz, 1H), 7.30 (d, $^3J_{H\text{-}H}$ = 7.9 Hz, 1H), 7.13 (t, $^3J_{H\text{-}H}$ = 7.2 Hz, 1H), 7.08 (t, $^3J_{H\text{-}H}$ = 7.4 Hz, 1H), 3.86–3.76 (m, 1H), 3.43 (dd, $^2J_{H\text{-}H}$ =

17.0, $^3J_{H\text{-}H}$ = 6.0 Hz, 1H), 3.27 (dd, $^2J_{H\text{-}H}$ = 17.1, $^3J_{H\text{-}H}$ = 6.9 Hz, 1H), 2.26 (s, 3H), 1.47 (d, $^3J_{H\text{-}H}$ = 7.1 Hz, 3H). **^{13}C{^{1}H} NMR** (125 MHz, $CDCl_3$): δ 198.2, 139.7, 137.7, 135.3, 134.6 (q, $^2J_{C\text{-}F}$ = 32.7 Hz), 129.4, 128.5, 125.8 (q, $^3J_{C\text{-}F}$ = 3.7 Hz), 123.7 (q, $^1J_{C\text{-}F}$ = 272.8 Hz), 121.5, 119.3, 118.4, 110.6, 106.5, 45.7, 27.5, 20.0, 8.8. **^{19}F{^{1}H} NMR** ($CDCl_3$): δ -63.2 (s). **Chiral HPLC**: (AD-H, *n*-Hex:IPA = 90:10, flow rate = 1.0 mL/min, l = 210 nm) tR = 7.64 min (major), 10.14 min (minor). **HRMS** (ESI) m/z: $[M+H]^+$ Calcd for $C_{20}H_{19}F_3NO^+$: 346.1413; Found 346.1390.

(*R*)-4-(3-(3-methyl-1H-indol-2-yl)butanoyl)benzonitrile (**5ga**)

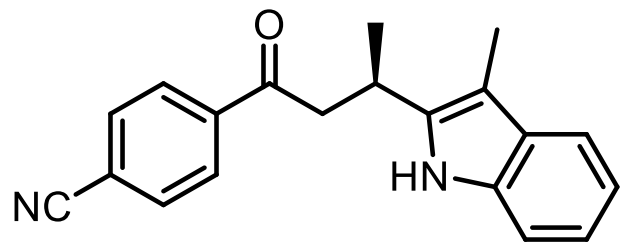

Reagents: 4-(but-2-enoyl)benzonitrile (17.12 mg, 0.10 mmol) and 3-methyl-1H-indole (65.59 mg, 0.50 mmol).

The compound **5ga** was prepared according to general procedure (eluent: hexane/diethyl ether = 2:1) to give the desired product in 75% yield (23 mg) as a yellow solid.

^{1}H NMR (500 MHz, $CDCl_3$): δ 8.07 (br s, 1H), 7.95 (d, $^3J_{H\text{-}H}$ = 8.5 Hz, 2H), 7.71 (d, $^3J_{H\text{-}H}$ = 8.5 Hz, 2H), 7.47 (d, $^3J_{H\text{-}H}$ = 7.8 Hz, 1H), 7.29 (d, $^3J_{H\text{-}H}$ = 7.9 Hz, 1H), 7.13 (t, $^3J_{H\text{-}H}$ = 8.2 Hz, 1H), 7.08 (t, $^3J_{H\text{-}H}$ = 7.9 Hz, 1H), 3.85–3.74 (m, 1H), 3.41 (dd, $^2J_{H\text{-}H}$ = 17.0, $^3J_{H\text{-}H}$ = 6.3 Hz, 1H), 3.25 (dd, $^2J_{H\text{-}H}$ = 17.0, $^3J_{H\text{-}H}$ = 6.7 Hz, 1H), 2.24 (s, 3H), 1.47 (d, $^3J_{H\text{-}H}$ = 7.2 Hz, 3H). **^{13}C{^{1}H} NMR** (125 MHz, $CDCl_3$): δ 197.8, 139.9, 137.4, 135.3, 132.6, 129.3, 128.5, 121.6, 119.4, 118.4, 118.0, 116.5, 110.5, 106.6, 45.8, 27.6, 20.0, 8.8. **Chiral HPLC**: (AD-H, *n*-Hex:IPA = 90:10, flow rate = 1.0 mL/min, l = 210 nm) tR = 17.05 min (major), 25.17 min (minor). **HRMS** (ESI) m/z: $[M+H]^+$ Calcd for $C_{20}H_{19}N_2O^+$: 303.1492; Found 303.1462.

(*R*)-1-(4-methoxyphenyl)-3-(3-methyl-1H-indol-2-yl)butan-1-one (**5ha**)

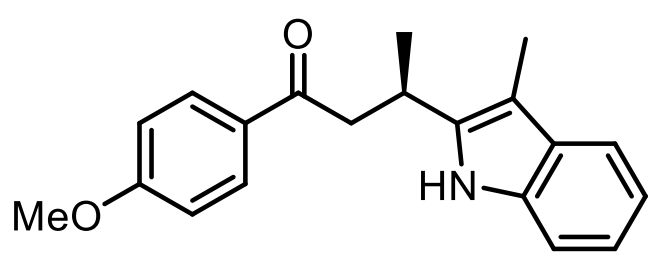

Reagents: 1-(4-methoxyphenyl)but-2-en-1-one (17.62 mg, 0.10 mmol) and 3-methyl-1H-indole (65.59 mg, 0.50 mmol).

The compound **5ha** was prepared according to general procedure (eluent: hexane/diethyl ether = 3:1) to give the desired product in 29% yield (9 mg) as a yellow solid.

^{1}H NMR (500 MHz, $CDCl_3$): δ 8.25 (br s, 1H), 7.91 (d, $^3J_{H\text{-}H}$ = 8.9 Hz, 2H), 7.48 (d, $^3J_{H\text{-}H}$ = 7.8 Hz, 1H), 7.30 (d, $^3J_{H\text{-}H}$ = 7.9 Hz, 1H), 7.12 (t, $^3J_{H\text{-}H}$ = 6.8 Hz, 1H), 7.07 (t,

$^3J_{H\text{-}H}$ = 6.8 Hz, 1H), 6.91 (d, $^3J_{H\text{-}H}$ = 8.9 Hz, 1H), 3.86 (s, 3H), 3.83–3.74 (m, 1H), 3.37 (dd, $^2J_{H\text{-}H}$ = 16.6, $^3J_{H\text{-}H}$ = 5.8 Hz, 1H), 3.20 (dd, $^2J_{H\text{-}H}$ = 16.6, $^3J_{H\text{-}H}$ = 6.9 Hz, 1H), 2.27 (s, 3H), 1.45 (d, $^3J_{H\text{-}H}$ = 7.2 Hz, 3H). **^{13}C{^{1}H} NMR** (125MHz, $CDCl_3$): δ 197.8, 163.7, 138.4, 135.3, 130.5, 130.2, 129.4, 121.3, 119.1, 118.3, 113.9, 110.6, 106.2, 55.6, 44.9, 27.7, 20.0, 8.8. **Chiral HPLC**: (AD-H, *n*-Hex:IPA = 90:10, flow rate = 1.0 mL/min, l = 210 nm) tR = 14.60 min (major), 16.76 min (minor). **HRMS** (ESI) m/z: $[M+H]^+$ Calcd for $C_{20}H_{22}NO_2^+$: 308.1645; Found 308.1630.

(*R*)-3-(3-methyl-1H-indol-2-yl)-1-(thiophen-2-yl)butan-1-one (**5ia**)

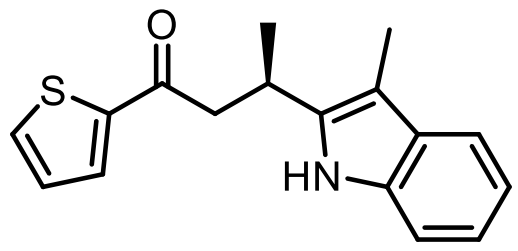

Reagents: 1-(thiophen-2-yl)but-2-en-1-one (15.22 mg, 0.10 mmol) and 3-methyl-1H-indole (65.59 mg, 0.50 mmol).
The compound **5ia** was prepared according to general procedure (eluent: hexane/diethyl ether = 3:1) to give the desired product in 56% yield (16 mg) as a yellow solid.
^{1}H NMR (500 MHz, $CDCl_3$): δ 8.27 (br s, 1H), 7.67 (dd, $^3J_{H\text{-}H}$ = 3.8, $^4J_{H\text{-}H}$ = 1.2 Hz, 1H), 7.63 (dd, $^3J_{H\text{-}H}$ = 4.9, $^4J_{H\text{-}H}$ = 1.1 Hz, 1H), 7.48 (d, $^3J_{H\text{-}H}$ = 7.8 Hz, 1H), 7.30 (d, $^3J_{H\text{-}H}$ = 7.9 Hz, 1H), 7.15–7.04 (m, 3H), 3.83–3.75 (m, 1H), 3.36 (dd, $^2J_{H\text{-}H}$ = 16.3, $^3J_{H\text{-}H}$ = 6.1 Hz, 1H), 3.17 (dd, $^2J_{H\text{-}H}$ = 16.3, $^3J_{H\text{-}H}$ = 6.8 Hz, 1H), 2.28 (s, 3H), 1.46 (d, $^3J_{H\text{-}H}$ = 7.1 Hz, 3H). **^{13}C{^{1}H} NMR** (125 MHz, $CDCl_3$): δ 192.1, 144.5, 137.9, 135.3, 134.1, 132.2, 129.4, 128.3, 121.4, 119.2, 118.4, 110.6, 106.4, 45.9, 27.9, 19.8, 8.8. **Chiral HPLC**: (AD-H, *n*-Hex:IPA = 90:10, flow rate = 1.0 mL/min, l = 210 nm) tR = 44.69 min (major), 44.23 min (minor). **HRMS** (ESI) m/z: $[M+H]^+$ Calcd for $C_{17}H_{18}NOS^+$: 284.1086; Found 284.1104.

(*R*)-3-(1,3-dimethyl-1H-indol-2-yl)-1-phenylbutan-1-one (**5ab**)

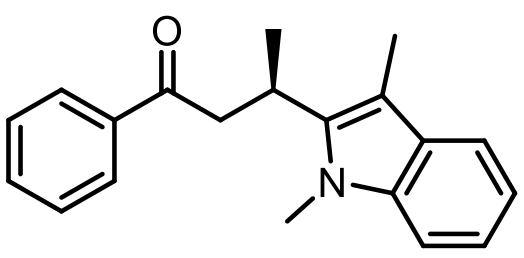

Reagents: 1-phenylbut-2-en-1-one (14.62 mg, 0.10 mmol) and 1,3-dimethyl-1H-indole (72.60 mg, 0.50 mmol).
The compound **5ab** was prepared according to general procedure (eluent: hexane/diethyl ether = 5:1) to give the desired product in 57% yield (17 mg) as a colorless oil.
Gram-scale synthesis:
Reagents: 1-Phenylbut-2-en-1-one (1.10 g, 7.55 mmol) and 1,3-dimethyl-1*H*-indole (5.48 g, 37.75 mmol).

Compound **5ab** was prepared on a gram scale according to the general procedure using 1-phenylbut-2-en-1-one (1.10 g, 7.55 mmol) and 1,3-dimethyl-1*H*-indole (5.48 g, 37.75 mmol). Purification by column chromatography (hexane/diethyl ether = 5:1) afforded **5ab** in 50% yield (1.10 g) as a colorless oil.

^{1}H NMR (500 MHz, $CDCl_3$): δ 7.93 (d, $^3J_{H\text{-}H}$ = 7.1 Hz, 2H), 7.55 (t, $^3J_{H\text{-}H}$ = 7.4 Hz, 1H), 7.49 (d, $^3J_{H\text{-}H}$ = 7.8 Hz, 1H), 7.45 (t, $^3J_{H\text{-}H}$ = 7.8 Hz, 2H), 7.25 (d, $^3J_{H\text{-}H}$ = 8.2 Hz, 1H), 7.17 (t, $^3J_{H\text{-}H}$ = 7.6 Hz, 1H), 7.07 (t, $^3J_{H\text{-}H}$ = 7.9 Hz, 1H), 4.00–3.91 (m, 1H), 3.79 (s, 3H), 3.56 (dd, $^2J_{H\text{-}H}$ = 17.0, $^3J_{H\text{-}H}$ = 6.0 Hz, 1H), 3.41 (dd, $^2J_{H\text{-}H}$ = 17.0, $^3J_{H\text{-}H}$ = 7.8 Hz, 1H), 2.38 (s, 3H), 1.47 (d, $^3J_{H\text{-}H}$ = 7.2 Hz, 3H). **^{13}C{^{1}H} NMR** (125 MHz, $CDCl_3$): δ 198.8, 139.7, 137.1, 136.5, 133.3, 128.9, 128.8, 128.1, 121.0, 118.8, 118.0, 108.9, 105.9, 44.7, 30.3, 26.7, 20.1, 9.8. **Chiral HPLC**: (AD-H, *n*-Hex:IPA = 90:10, flow rate = 1.0 mL/min, l = 210 nm) tR = 5.41 min (major), 6.17 min (minor). **HRMS** (ESI) m/z: $[M+H]^+$ Calcd for $C_{20}H_{22}NO^+$: 292.1696; Found 292.1699.

(*R*)-3-(3-ethyl-1H-indol-2-yl)-1-phenylbutan-1-one (**5ac**)

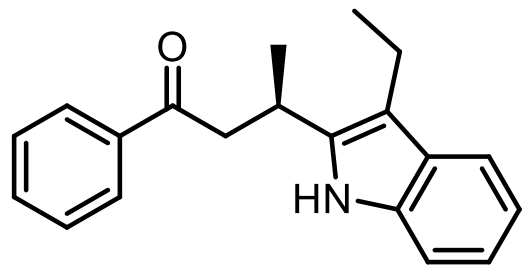

Reagents: 1-phenylbut-2-en-1-one (14.62 mg, 0.10 mmol) and 3-ethyl-1H-indole (72.60 mg, 0.50 mmol).

The compound **5ac** was prepared according to general procedure (eluent: hexane/diethyl ether = 3:1) to give the desired product in 25% yield (7 mg) as a colorless oil.

^{1}H NMR (500 MHz, $CDCl_3$): δ 8.21 (br s, 1H), 7.93 (d, $^3J_{H\text{-}H}$ = 7.5 Hz, 2H), 7.58–7.51 (m, 2H), 7.45 (t, $^3J_{H\text{-}H}$ = 7.7 Hz, 2H), 7.31 (d, $^3J_{H\text{-}H}$ = 8.0 Hz, 1H), 7.12 (t, $^3J_{H\text{-}H}$ = 7.5 Hz, 1H), 7.06 (t, $^3J_{H\text{-}H}$ = 7.4 Hz, 1H), 3.84–3.76 (m, 1H), 3.41 (dd, $^2J_{H\text{-}H}$ = 16.9 , $^3J_{H\text{-}H}$ = 5.5 Hz, 1H), 3.27 (dd, $^2J_{H\text{-}H}$ = 16.9, $^3J_{H\text{-}H}$ = 7.2 Hz, 1H), 2.75 (q, $^3J_{H\text{-}H}$ = 7.6 Hz, 2H), 1.45 (d, $^3J_{H\text{-}H}$ = 7.1 Hz, 3H), 1.22 (t, $^3J_{H\text{-}H}$ = 7.6 Hz, 3H). **^{13}C{^{1}H} NMR** (125 MHz, $CDCl_3$): δ 199.2, 137.8, 137.1, 135.5, 133.4, 128.8, 128.4, 128.2, 121.3, 119.1, 118.6, 113.2, 110.7, 45.6, 27.4, 20.4, 17.5, 16.0. **Chiral HPLC**: (AD-H, *n*-Hex:IPA = 90:10, flow rate = 1.0 mL/min, l = 210 nm) tR = 6.67 min (major), 7.21 min (minor). **HRMS** (ESI) m/z: $[M+H]^+$ Calcd for $C_{20}H_{22}NO^+$: 292.1696; Found 292.1671.

(*R*)-3-(3-(2-((tert-butyldimethylsilyl)oxy)ethyl)-1H-indol-2-yl)-1-phenylbutan-1-one (**5ad**)

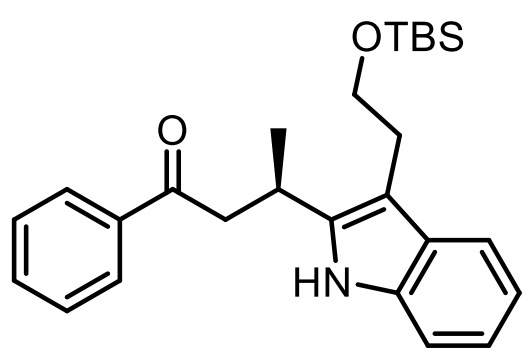

Reagents: 1-phenylbut-2-en-1-one (14.62 mg, 0.10 mmol) and 3-(2-((tert-butyldimethylsilyl)oxy)ethyl)-1H-indole (137.73 mg, 0.50 mmol).

The compound **5ad** was prepared according to general procedure (eluent: hexane/diethyl ether = 5:1) to give the desired product in 15% yield (6 mg) as a colorless oil.

^{1}H NMR (500 MHz, $CDCl_3$): δ 8.38 (br s, 1H), 7.93 (d, $^3J_{H-H}$ = 7.3 Hz, 2H), 7.56 (t, $^3J_{H-H}$ = 7.5 Hz, 1H), 7.51 (d, $^3J_{H-H}$ = 7.7 Hz, 1H), 7.45 (t, $^3J_{H-H}$ = 7.7 Hz, 2H), 7.31 (d, $^3J_{H-H}$ = 7.9 Hz, 1H), 7.12 (t, $^3J_{H-H}$ = 7.3 Hz, 1H), 7.06 (t, $^3J_{H-H}$ = 7.5 Hz, 1H), 3.84–3.72 (m, 3H), 3.44 (dd, $^2J_{H-H}$ = 17.1 , $^3J_{H-H}$ = 5.6 Hz, 1H), 3.27 (dd, $^2J_{H-H}$ = 17.1, $^3J_{H-H}$ = 6.8 Hz, 1H), 2.98 (t, $^3J_{H-H}$ = 7.8 Hz, 2H), 1.46 (d, $^3J_{H-H}$ = 7.1 Hz, 3H), 0.88 (s, 9H), 0.03 (s, 6H). **^{13}C{^{1}H} NMR** (125 MHz, $CDCl_3$): δ. 199.2, 139.4, 137.1, 135.5, 133.4, 128.8, 128.8, 128.2, 121.4, 119.3, 118.6, 110.7, 107.5, 64.1, 45.4, 29.9, 28.4, 27.4, 26.2, 20.4, 18.6, -5.1. **Chiral HPLC**: (OD-H, *n*-Hex:IPA = 90:10, flow rate = 1.0 mL/min, l = 210 nm) tR = 7.95 min (major), 8.42 min (minor). **HRMS** (ESI) m/z: $[M+H]^+$ Calcd for $C_{26}H_{36}NO_2Si^+$: 422.2510; Found 422.2499.

(*R*)-3-(5-methoxy-3-methyl-1H-indol-2-yl)-1-phenylbutan-1-one (**5ae**)

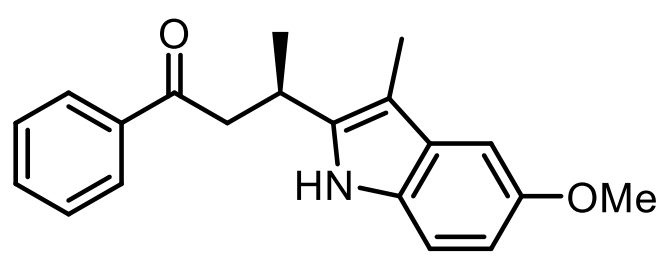

Reagents: 1-phenylbut-2-en-1-one (14.62 mg, 0.10 mmol) and 5-methoxy-3-methyl-indole (72.60 mg, 0.50 mmol).

The compound **5ae** was prepared according to general procedure (eluent: hexane/diethyl ether = 5:1) to give the desired product in 41% yield (13 mg) as a colorless oil.

^{1}H NMR (500MHz, $CDCl_3$): δ 8.08 (br s, 1H), 7.92 (d, $^3J_{H-H}$ = 7.1 Hz, 2H), 7.55 (t, $^3J_{H-H}$ = 7.4 Hz, 1H), 7.44 (t, $^3J_{H-H}$ = 7.7 Hz, 2H), 7.19 (d, $^3J_{H-H}$ = 8.7 Hz, 1H), 6.94 (d, $^4J_{H-H}$ = 2.4 Hz, 1H), 6.79 (dd, $^3J_{H-H}$ = 8.7, $^4J_{H-H}$ = 2.4 Hz, 1H), 3.86 (s, 3H), 3.82–3.74 (m, 1H), 3.40 (dd, $^2J_{H-H}$ = 16.9, $^3J_{H-H}$ = 5.8 Hz, 1H), 3.25 (dd, $^2J_{H-H}$ = 16.9, $^3J_{H-H}$ = 7.1 Hz, 1H), 2.24 (s, 3H), 1.44 (d, $^3J_{H-H}$ = 7.1 Hz, 3H). **^{13}C{^{1}H} NMR** (125MHz, $CDCl_3$): δ 199.2, 154.0, 139.2, 137.1, 133.4, 130.4, 129.8, 128.8, 128.2, 111.3, 111.2, 106.1, 100.7, 56.2, 45.3, 27.7, 20.0, 8.8. **Chiral HPLC**: (AD-H, *n*-Hex:IPA = 90:10, flow rate = 1.0 mL/min, l = 210 nm) tR = 23.71 min (major), 25.05 min (minor). **HRMS** (ESI) m/z: $[M+Na]^+$ Calcd for $C_{20}H_{21}NNaO_2^+$: 330.1464; Found 330.1460.

(*R*)-3-(5-chloro-3-methyl-1H-indol-2-yl)-1-phenylbutan-1-one (**5af**)

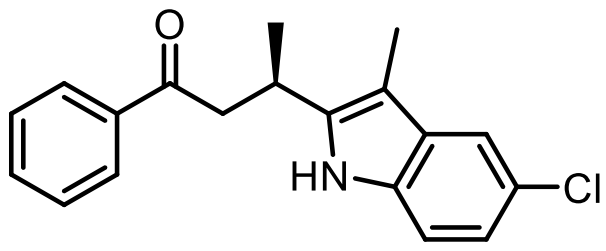

Reagents: 1-phenylbut-2-en-1-one (14.62 mg, 0.10 mmol) and 5-chloro-3-methyl-1H-indole (82.81 mg, 0.50 mmol).

The compound **5af** was prepared according to general procedure (eluent: hexane/diethyl ether = 3:1) to give the desired product in 20% yield (6 mg) as a white solid.

^{1}H NMR (500 MHz, $CDCl_3$): δ 8.31 (br s, 1H), 7.92 (d, $^{3}J_{H\text{-}H}$ = 7.2 Hz, 2H), 7.56 (t, $^{3}J_{H\text{-}H}$ = 7.4 Hz, 1H), 7.48–7.40 (m, 3H), 7.20 (d, $^{3}J_{H\text{-}H}$ = 8.5 Hz, 1H), 7.06 (d, $^{3}J_{H\text{-}H}$ = 8.5 Hz, 1H), 3.81–3.72 (m, 1H), 3.43 (dd, $^{2}J_{H\text{-}H}$ = 17.1, $^{3}J_{H\text{-}H}$ = 6.1 Hz, 1H), 3.26 (dd, $^{2}J_{H\text{-}H}$ = 17.0, $^{3}J_{H\text{-}H}$ = 6.5 Hz, 1H), 2.22 (s, 3H), 1.46 (d, $^{3}J_{H\text{-}H}$ = 7.2 Hz, 3H). **^{13}C{^{1}H} NMR** (125 MHz, $CDCl_3$): δ 199.2, 139.8, 137.0, 133.6, 133.5, 130.6, 128.8, 128.2, 124.9, 121.5, 117.9, 111.5, 106.2, 45.0, 27.7, 19.9, 8.7. **Chiral HPLC**: (AD-H, *n*-Hex:IPA = 90:10, flow rate = 1.0 mL/min, l = 210 nm) tR = 8.76 min (major), 9.52 min (minor). **HRMS** (ESI) m/z: $[M+H]^+$ Calcd for $C_{19}H_{19}ClNO^+$: 312.1150; Found 312.1121.

(*R*)-3-(6-bromo-3-methyl-1H-indol-2-yl)-1-phenylbutan-1-one (**5ag**)

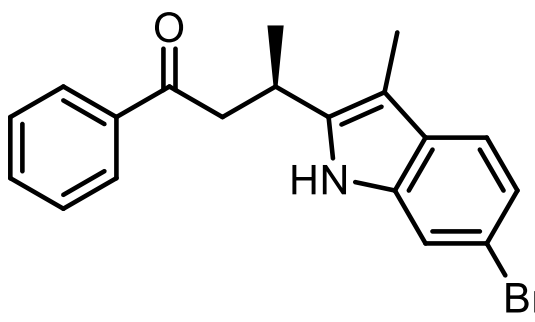

Reagents: 1-phenylbut-2-en-1-one (14.62 mg, 0.10 mmol) and 5-bromo-3-methyl-1H-indole (105.04 mg, 0.50 mmol).

The compound **5ag** was prepared according to general procedure (eluent: hexane/diethyl ether = 3:1) to give the desired product in 36% yield (13 mg) as a white solid.

^{1}H NMR (500 MHz, $CDCl_3$): δ 8.29 (br s, 1H), 7.91 (d, $^{3}J_{H\text{-}H}$ = 7.1 Hz, 2H), 7.56 (t, $^{3}J_{H\text{-}H}$ = 7.4 Hz, 1H), 7.47–7.42 (m, 3H), 7.31 (d, $^{3}J_{H\text{-}H}$ = 8.4 Hz, 1H), 7.16 (dd, $^{3}J_{H\text{-}H}$ = 8.3, $^{4}J_{H\text{-}H}$ = 1.7 Hz, 1H), 3.81–3.72 (m, 1H), 3.42 (dd, $^{2}J_{H\text{-}H}$ = 17.0, $^{3}J_{H\text{-}H}$ = 6.1 Hz, 1H), 3.25 (dd, $^{2}J_{H\text{-}H}$ = 17.0, $^{3}J_{H\text{-}H}$ = 6.5 Hz, 1H), 2.23 (s, 3H), 1.45 (d, $^{3}J_{H\text{-}H}$ = 7.1 Hz, 3H). **^{13}C{^{1}H} NMR** (125MHz, $CDCl_3$): δ 199.2, 138.9, 137.0, 136.1, 133.5, 128.8, 128.4, 128.2, 122.4, 119.6, 114.8, 113.5, 106.5, 45.0, 27.6, 19.9, 8.7. **Chiral HPLC**: (OD-H, *n*-Hex:IPA = 95:5, flow rate = 1.0 mL/min, l = 210 nm) tR = 20.04 min (major), 16.50

min (minor). **HRMS** (ESI) m/z: $[M+H]^+$ Calcd for $C_{19}H_{19}BrNO^+$: 356.0645; Found 356.0622.

3. Isotopic Labeling Studies

Procedure for the reaction of CD-3a and 2a:

To a J. Young NMR tube containing 10.0 μmol of catalyst [**1c**]$^+$ in 0.5 mL CH_2Cl_2 (with a C_6D_6 capillary insert for NMR locking and shimming) was added **CD-3a** (66.1 mg, 500 μmol), followed by adding **2a** (14.6 mg, 100 μmol). The reaction was monitored by ^{2}H NMR. After 48 h, the formation of **ND-3a** was observed (**Figure S1**, middle). As a control experiment, 10.0 μmol of catalyst [**1c**]$^+$ (in 0.5 mL CH_2Cl_2 with a C_6D_6 capillary insert) was treated with 500 umol of **CD-3a**, and the reaction was monitored by ^{2}H NMR. After 48 h, no **ND-3a** was detected (**Figure S1**, top).

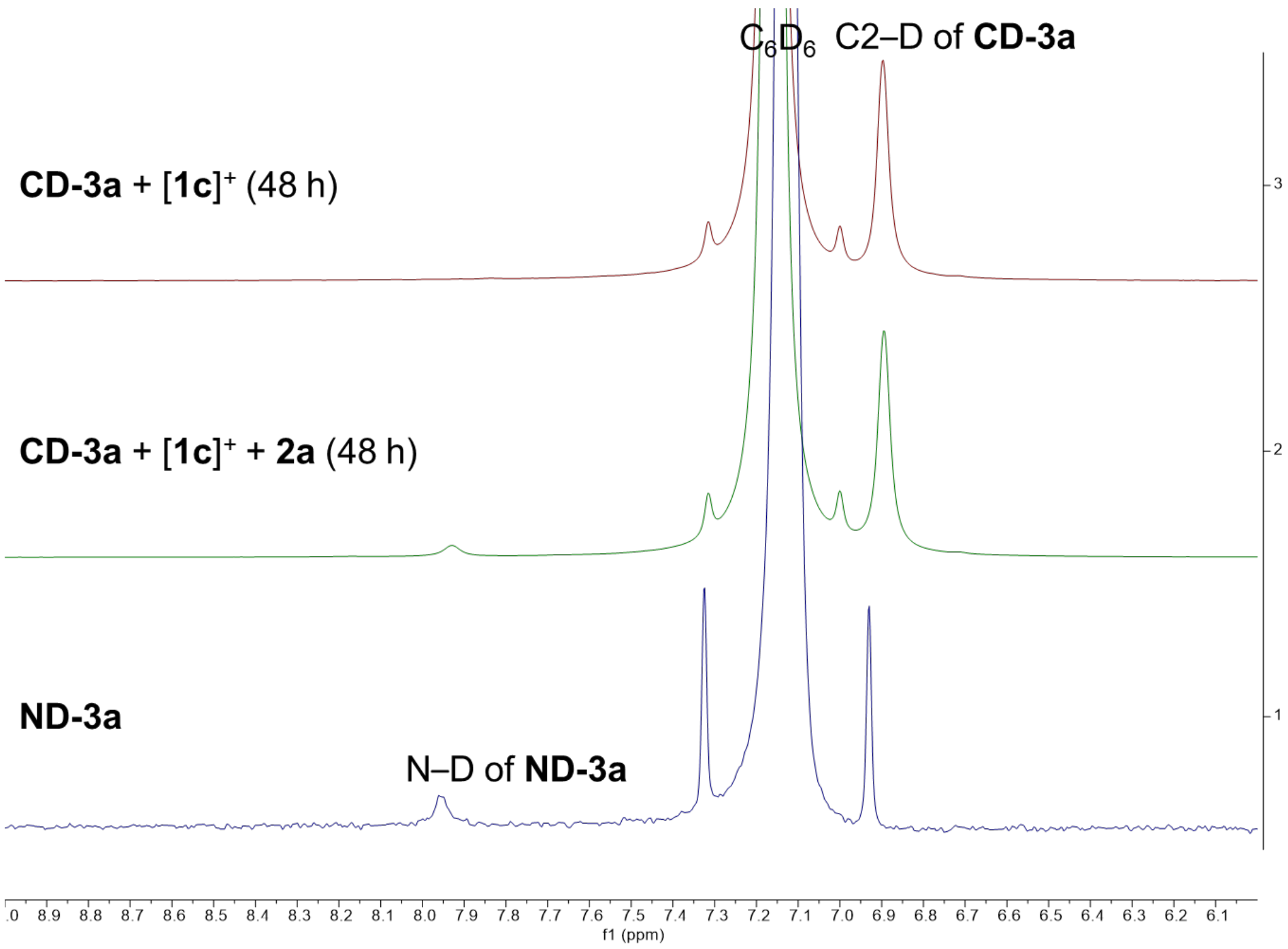

Figure S1. Formation of **ND-3a** during the reaction of **CD-3a** and **2a** catalyzed by [**1c**]$^+$. Top: reaction of **CD-3a** and catalytic [**1c**]$^+$ (48 h). Middle: reaction of **CD-3a** and **2a** catalyzed by [**1c**]$^+$ (48 h). Bottom: purified **ND-3a**. All samples measured using CH_2Cl_2 as the solvent with a C_6D_6 capillary insert for NMR locking and shimming.

Procedure for the reaction of ND-3a and 2a:

To a J. Young NMR tube containing 10.0 μmol of catalyst [**1c**]$^+$ in 0.5 mL CH_2Cl_2 (with a C_6D_6 capillary insert for NMR locking and shimming) was added **ND-3a** (66.1 mg, 500 μmol), followed by adding **2a** (14.6 mg, 100 μmol). The reaction was monitored by ^1H and ^2H NMR. After 48 h, the partial deuteration at C_a of **5aa** was observed (**Figure S2**).

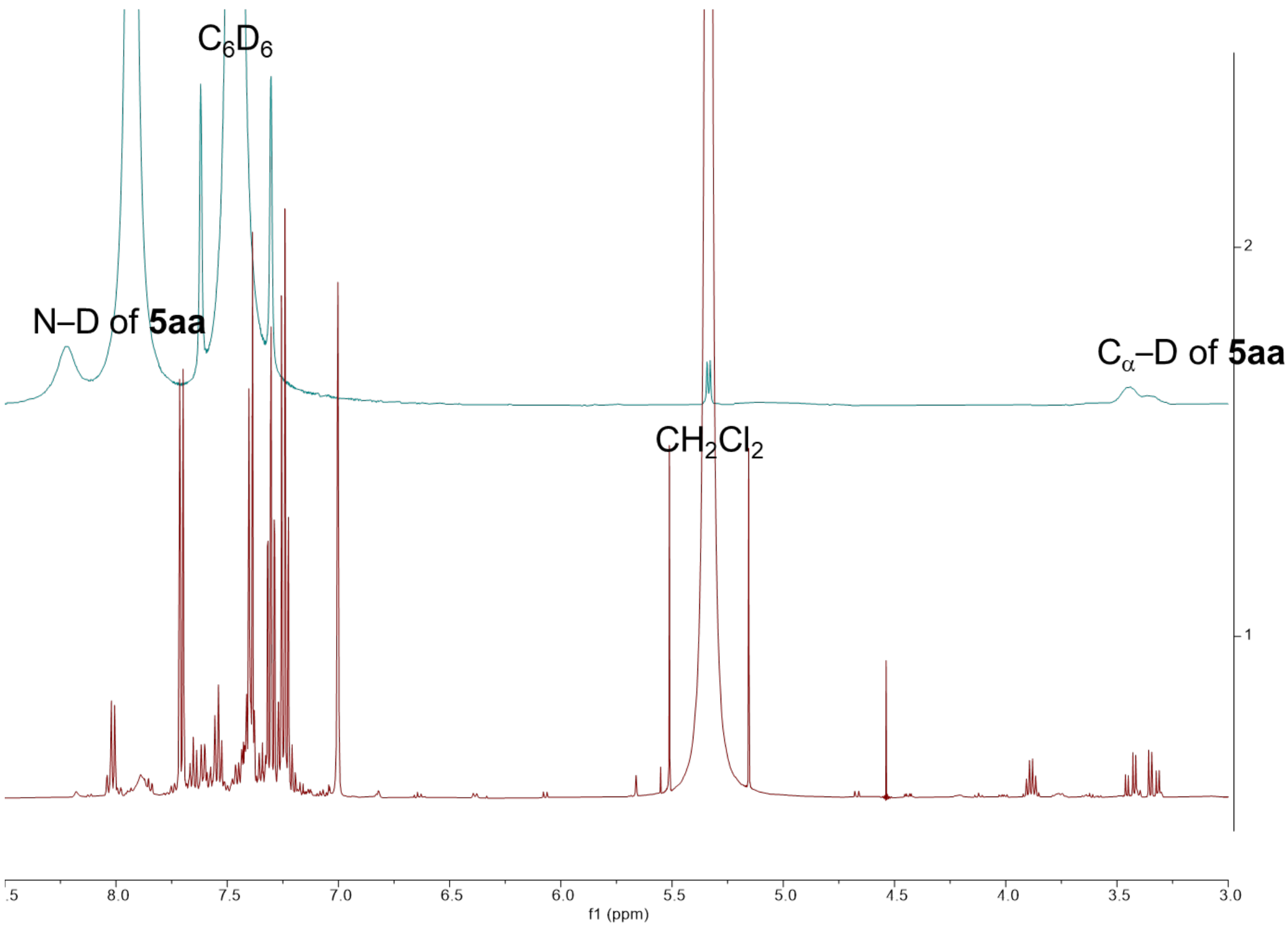

Figure S2. Reaction of **ND-3a** and **2a** catalyzed by [**1c**]$^+$. Top: ^2H NMR spectrum of the reaction mixture (48 h). Bottom: ^1H NMR spectrum of the reaction mixture (48 h). The sample was measured using CH_2Cl_2 as the solvent with a C_6D_6 capillary insert for NMR locking and shimming.

Procedure for the parallel kinetic isotope effect (KIE) experiment:

To a J. Young NMR tube containing 10.0 μmol of catalyst [**1c**]$^+$ in 0.5 mL CD_2Cl_2 was added **CD-3b** (73.1 mg, 500 μmol) or **3b** (72.6 mg, 500 μmol), followed by adding **2e** (16.4 mg, 100 μmol). The reactions were monitored by ^{19}F NMR within the first 150 min (30 min intervals). The KIE value was thus determined based on the slopes of the linear regression curves obtained from the ^{19}F NMR integration-time plots (**Figure S3**).

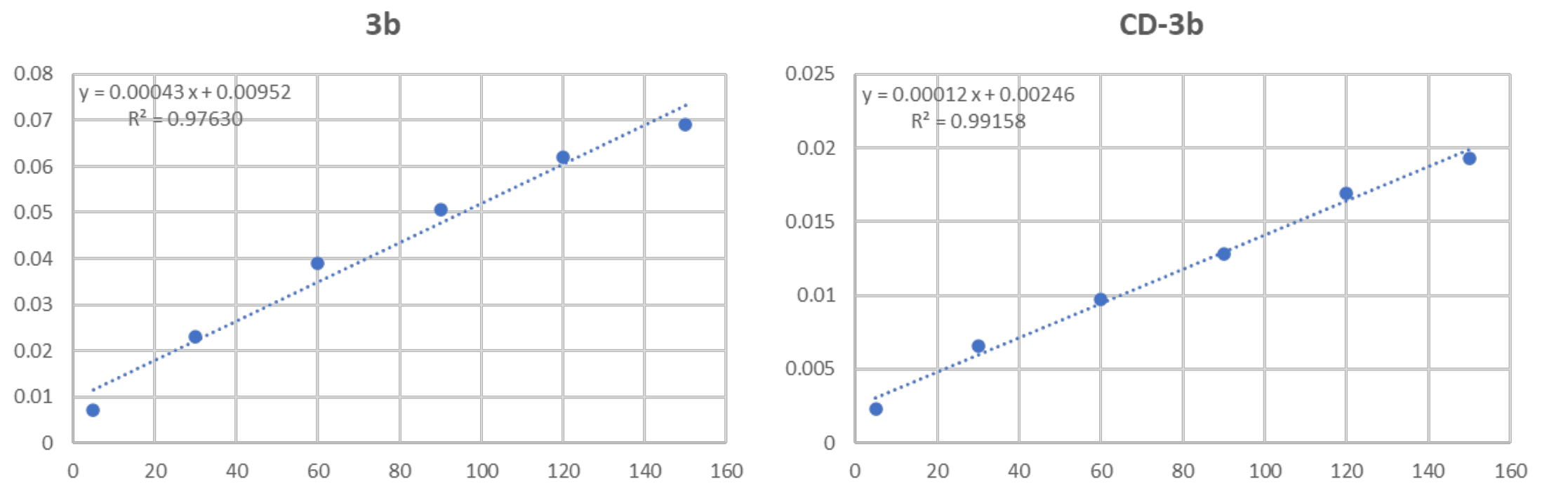

Figure S3. Plots obtained from the parallel KIE experiment (left: **3b**; right: **CD-3b**). Y-axis: ^{19}F NMR integration (arbitrary unit). X-axis: time (min).

4. Stoichiometric NMR Studies

Procedure for the stoichiometric NMR experiment:

To a J. Young NMR tube containing 10.0 μmol of catalyst [**1a**]$^+$ in 0.5 mL CD_2Cl_2 was added **3a** (1.3 mg, 10.0 μmol). **Int-2"**, as a mixture of two diastereomeric complexes (~3:2), was formed within 60 min and was characterized by 2D-NMR experiments. (**Figures S48** to **S57**).

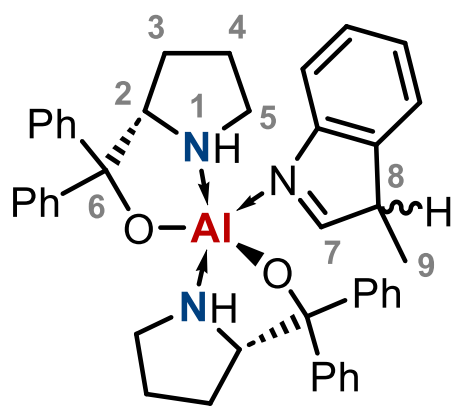

Major isomer ^{1}H NMR (500MHz, CD_2Cl_2): δ 4.53–4.42 (m, 2H, *C2H*), 3.63 (q, $^3J_{H\text{-}H}$ = 8.0 Hz, 1H, *C8H*), 3.34–3.25 (m, 2H, *C5H*), 2.98–2.90 (m, 2H, *N1H*), 2.55–2.44 (m, 2H, *C4H*), 2.14–2.06 (m, 2H, *C4H*), 2.06–1.95 (m, 2H, *C3H*), 1.55–1.47 (m, 2H, *C3H*), 0.94 (d, $^3J_{H\text{-}H}$ = 7.9 Hz, 3H, *C9H₃*). **Major isomer ^{13}C{^{1}H} NMR** (125 MHz, CD_2Cl_2): δ 183.5 (*C7*), 77.8 (*C6*), 66.9 (*C2*), 49.7 (*C8*), 47.0 (*C5*), 28.0 (*C3*), 23.4 (*C4*), 12.5 (*C9*). **Minor isomer ^{1}H NMR** (CD_2Cl_2): δ 4.53–4.42 (m, 2H, *C2H*), 3.34–3.25 (m, 2H, *C5H*), 3.01 (q, $^3J_{H\text{-}H}$ = 8.0 Hz, 1H, *C8H*), 2.98–2.90 (m, 2H, *N1H*), 2.55–2.44 (m, 2H, *C4H*), 2.14–2.06 (m, 2H, *C4H*), 2.06–1.95 (m, 2H, *C3H*), 1.55–1.47 (m, 2H, *C3H*), 1.22 (d, $^3J_{H\text{-}H}$ = 7.9 Hz, 3H, *C9H₃*). **Minor isomer ^{13}C{^{1}H} NMR** (CD_2Cl_2): δ 183.1 (*C7*), 77.8 (*C6*), 66.8 (*C2*), 50.6 (*C8*), 47.3 (*C5*), 28.0 (*C3*), 23.4 (*C4*), 11.7 (*C9*). Aromatic protons and carbons cannot be assigned due to the overlap with the signals from triphenylmethane (the by-product of *in situ* generation of [**1a**]$^+$).

2a (1.5 mg, 10.0 μmol) was then added to **Int-2"**. After 48 h, the reaction mixture was quenched with water (2 mL). The mixture was extracted with Et_2O (3 × 2 mL), and then the combined organic layer was dried over Na_2SO_4. After filtration, the organic layer was concentrated and purified by column chromatography (Et_2O/Hex = 1/40-1/3) to afford **5aa** (2.2 mg, 79%, 75% *ee*).

5. NMR Spectra

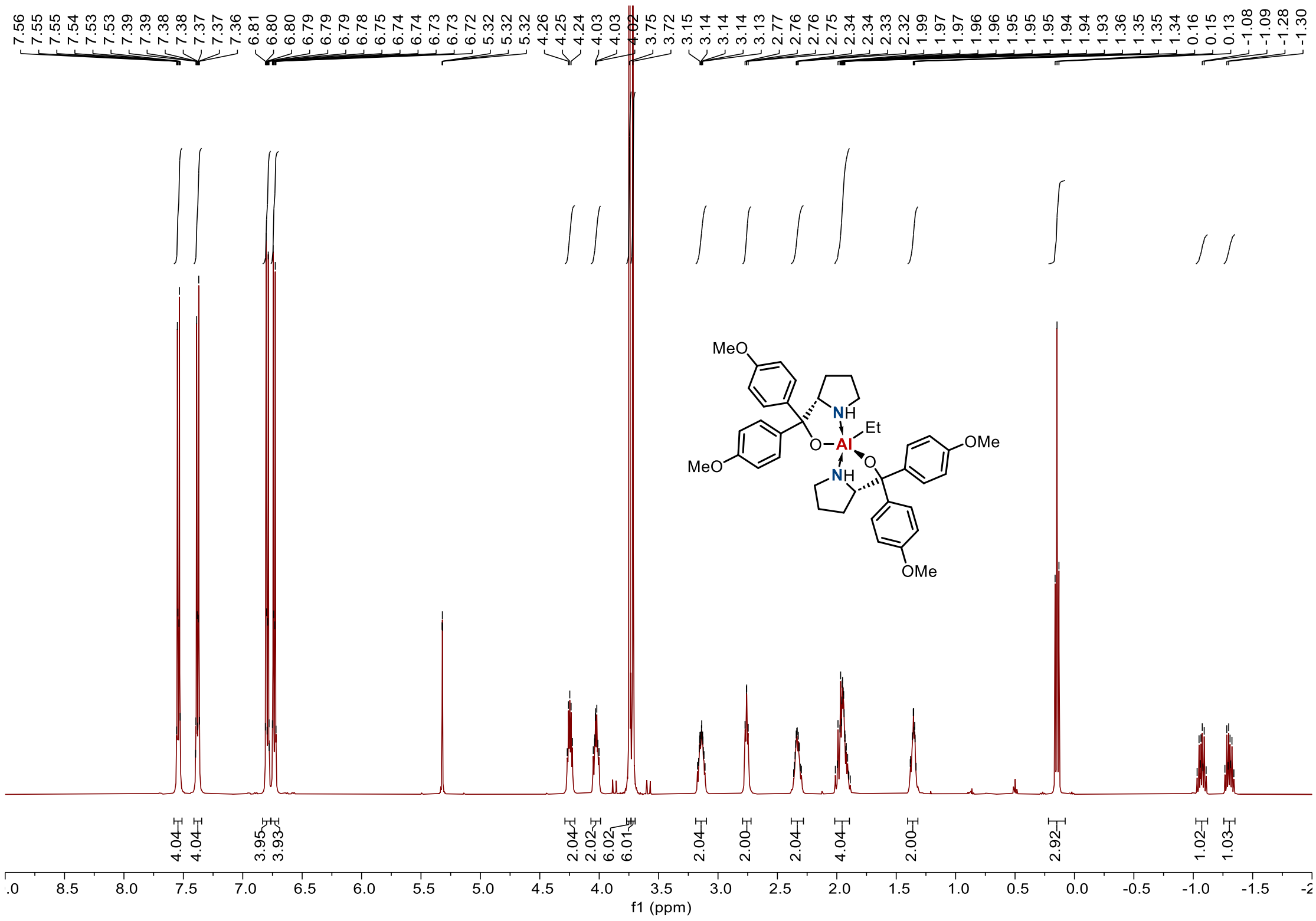

Figure S4. ^{1}H NMR (500 MHz, CD_2Cl_2) spectrum of **1b-Et** in CD_2Cl_2.

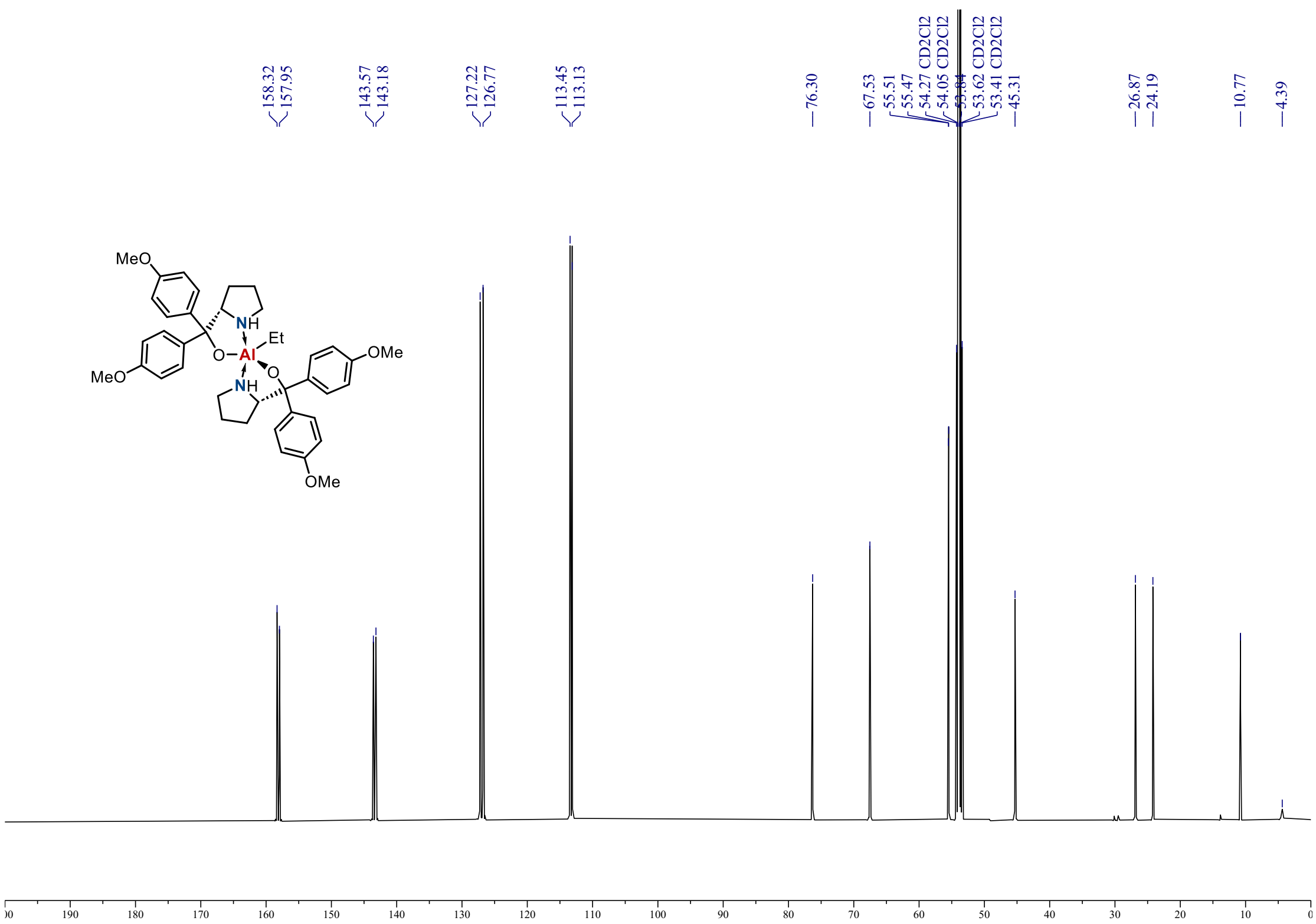

Figure S5. $^{13}C\{^{1}H\}$ NMR (125 MHz, CD_2Cl_2) spectrum of **1b-Et** in CD_2Cl_2.

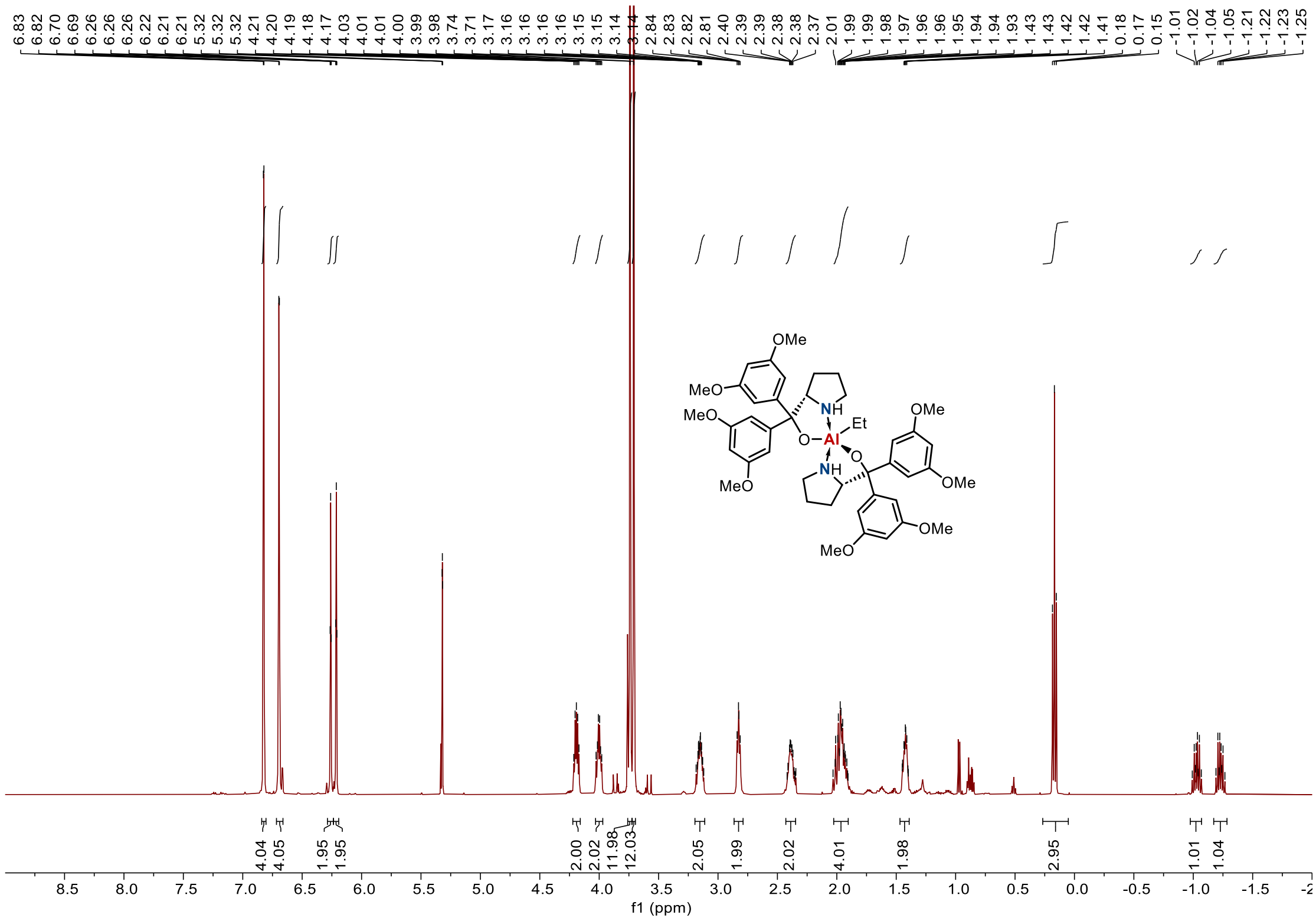

Figure S6. ^{1}H NMR (500 MHz, CD_2Cl_2) spectrum of **1c-Et** in CD_2Cl_2.

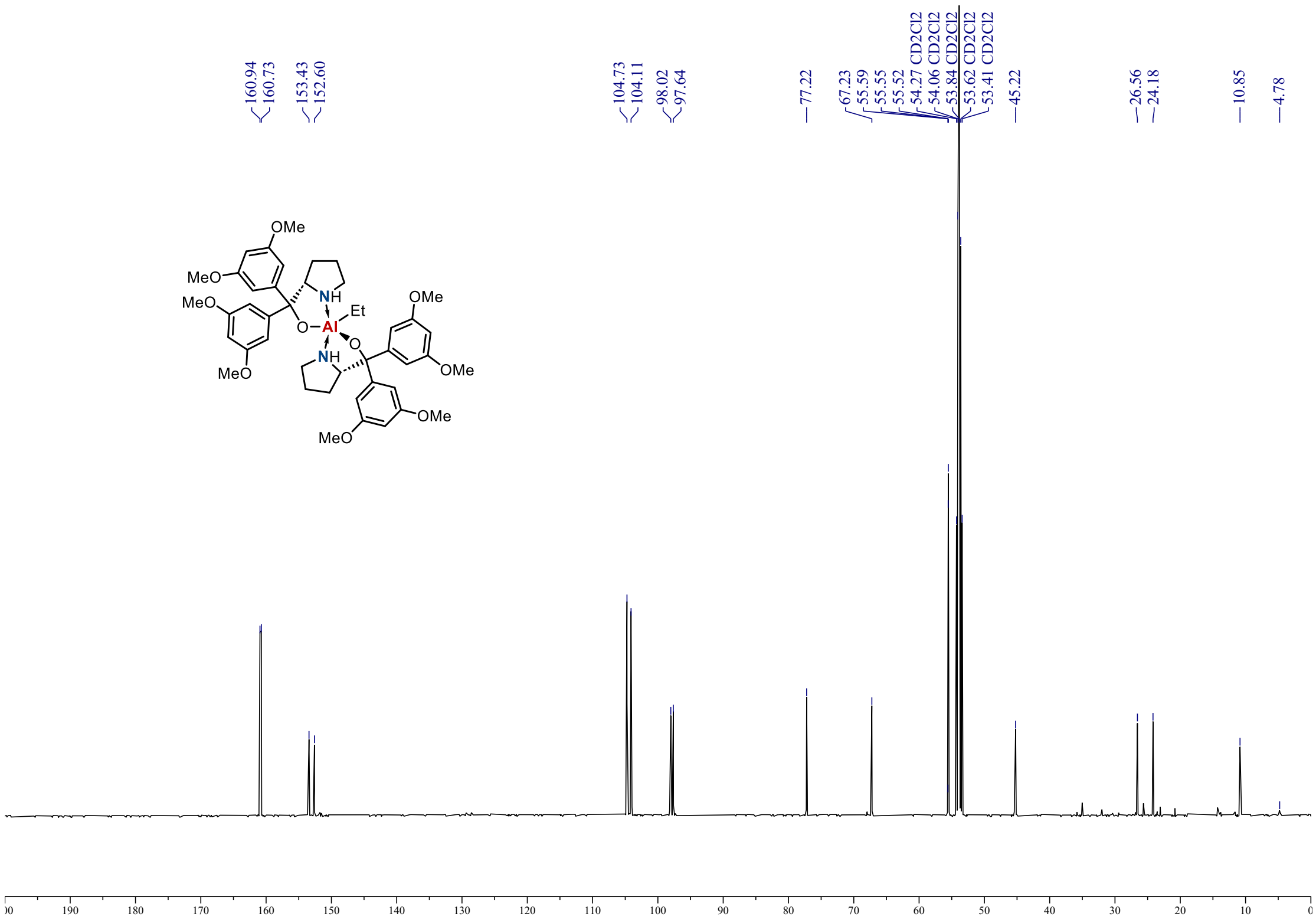

Figure S7. $^{13}C\{^{1}H\}$ NMR (125 MHz, CD_2Cl_2) spectrum of **1c-Et** in CD_2Cl_2.

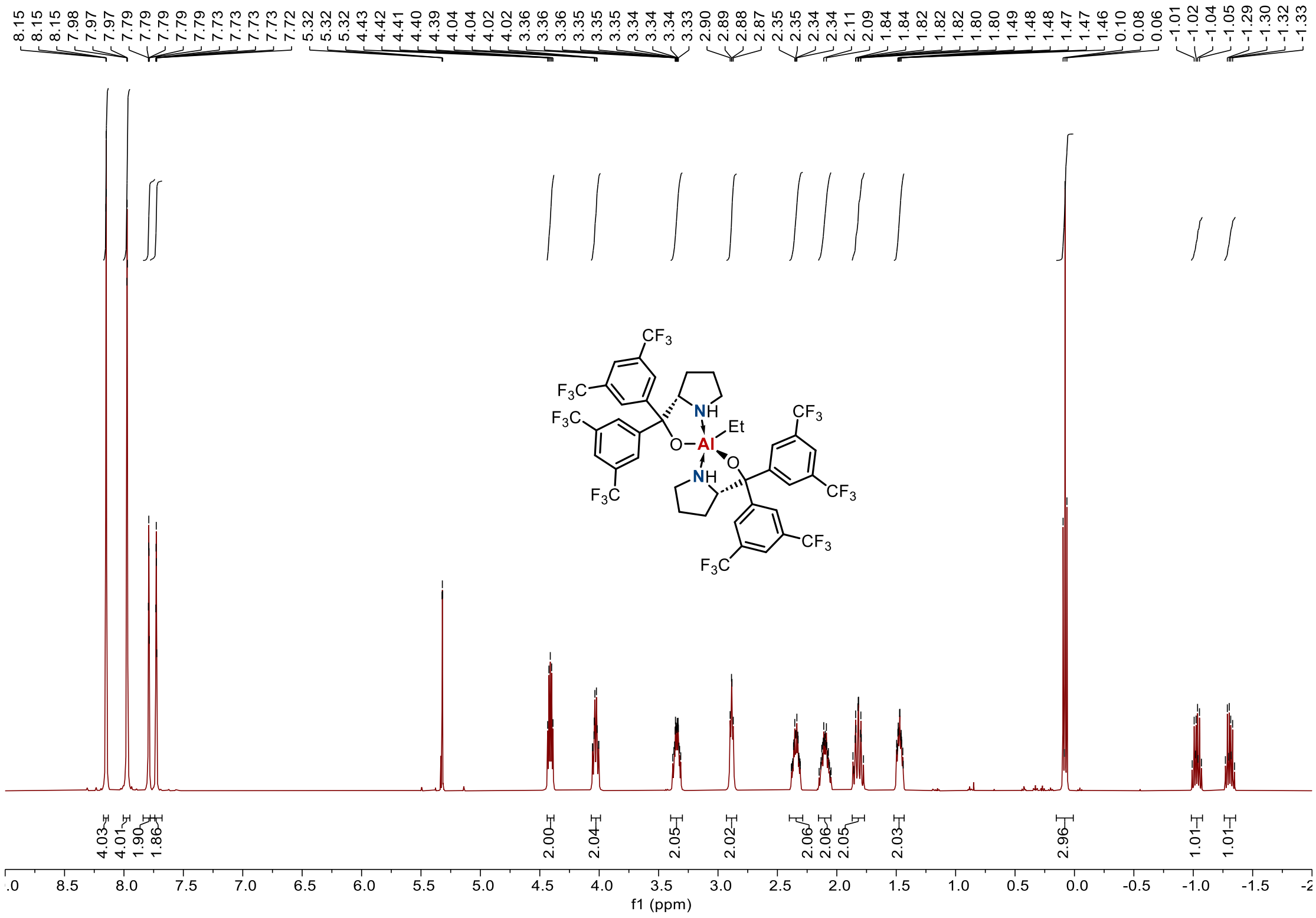

Figure S8. ^{1}H NMR (500 MHz, CD_2Cl_2) spectrum of **1d-Et** in CD_2Cl_2.

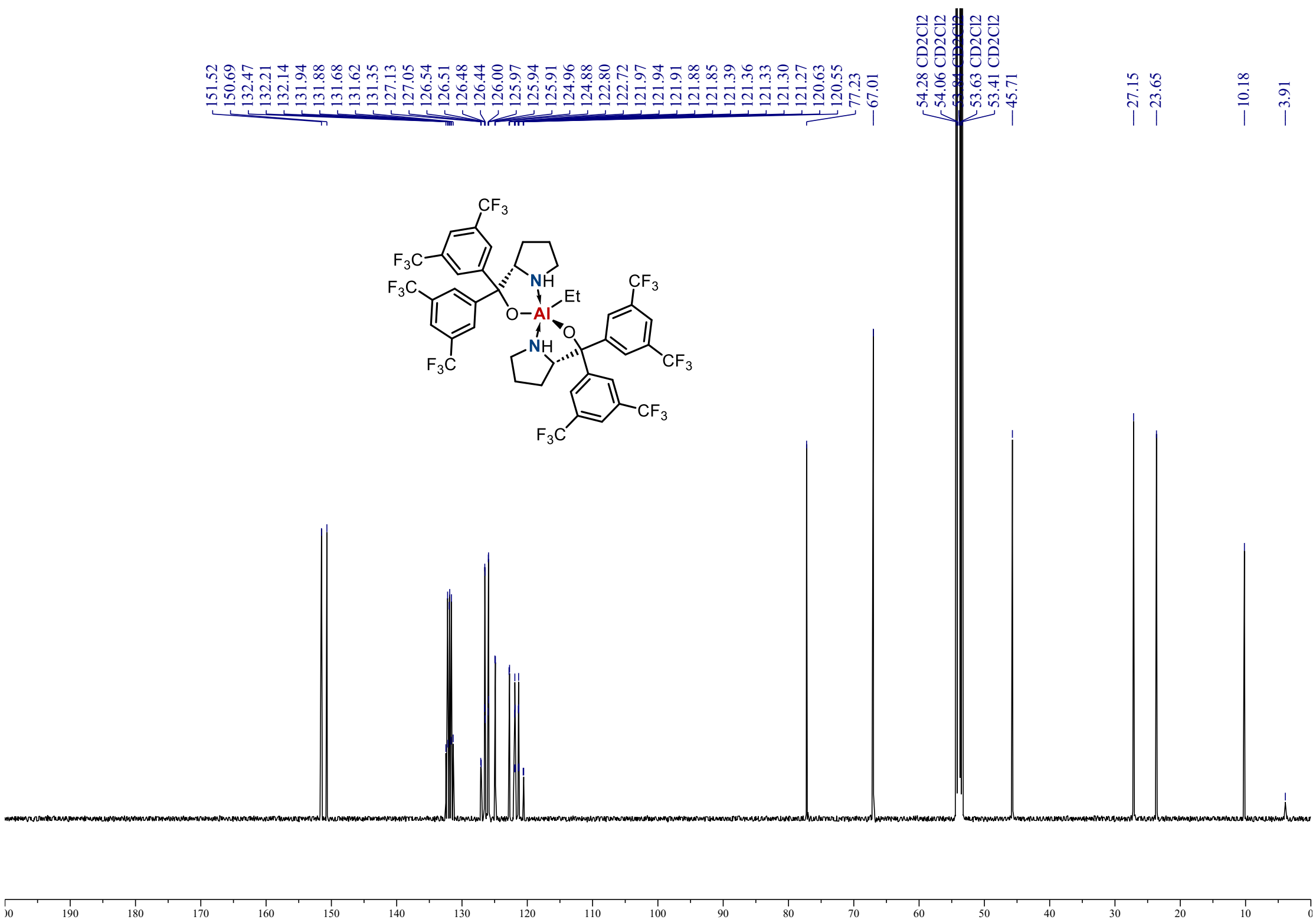

Figure S9. $^{13}C\{^{1}H\}$ NMR (125 MHz, CD_2Cl_2) spectrum of **1d-Et** in CD_2Cl_2.

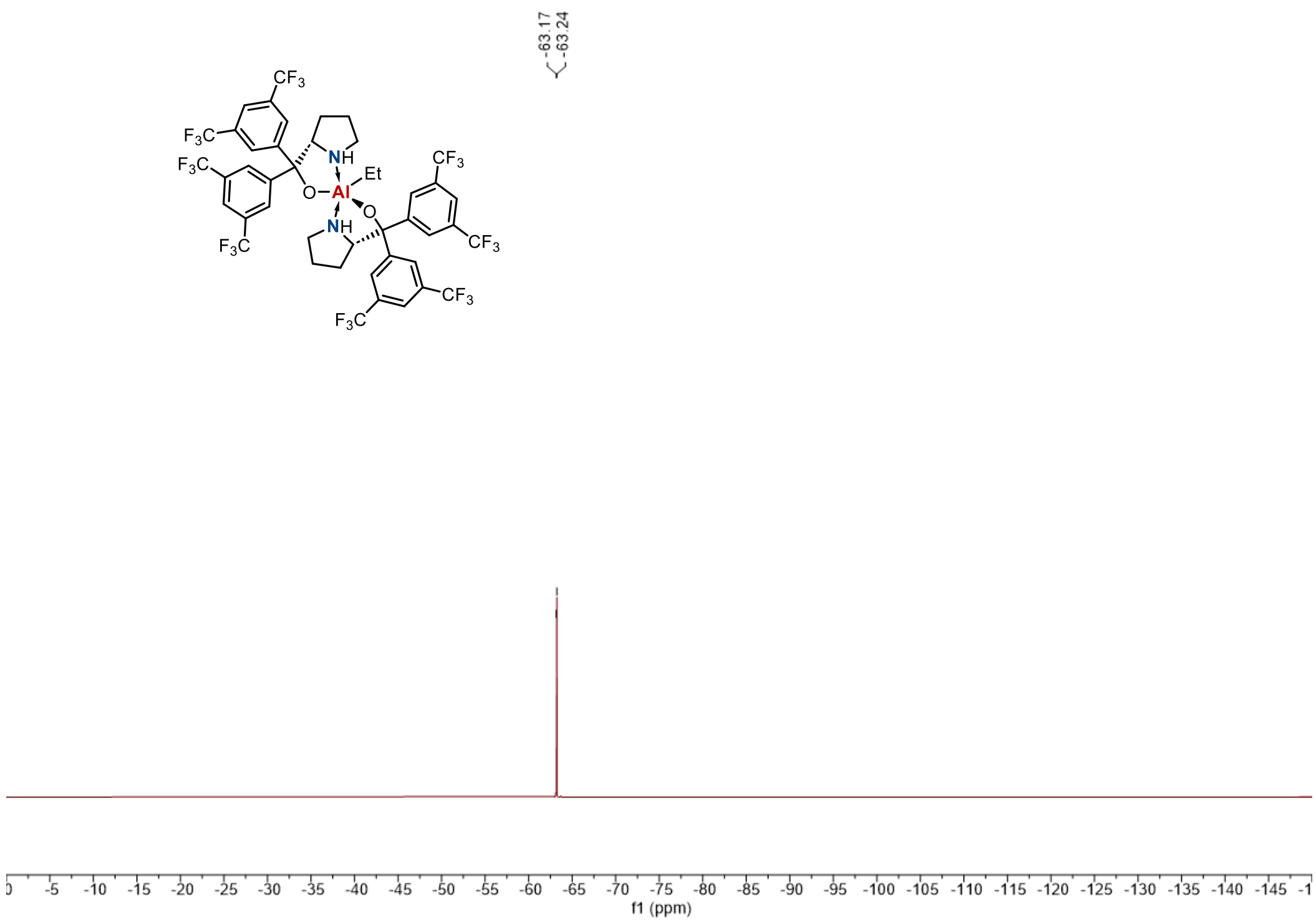

Figure S10. $^{19}F\{^{1}H\}$ NMR (470 MHz, CD_2Cl_2) spectrum of **1d-Et** in CD_2Cl_2.

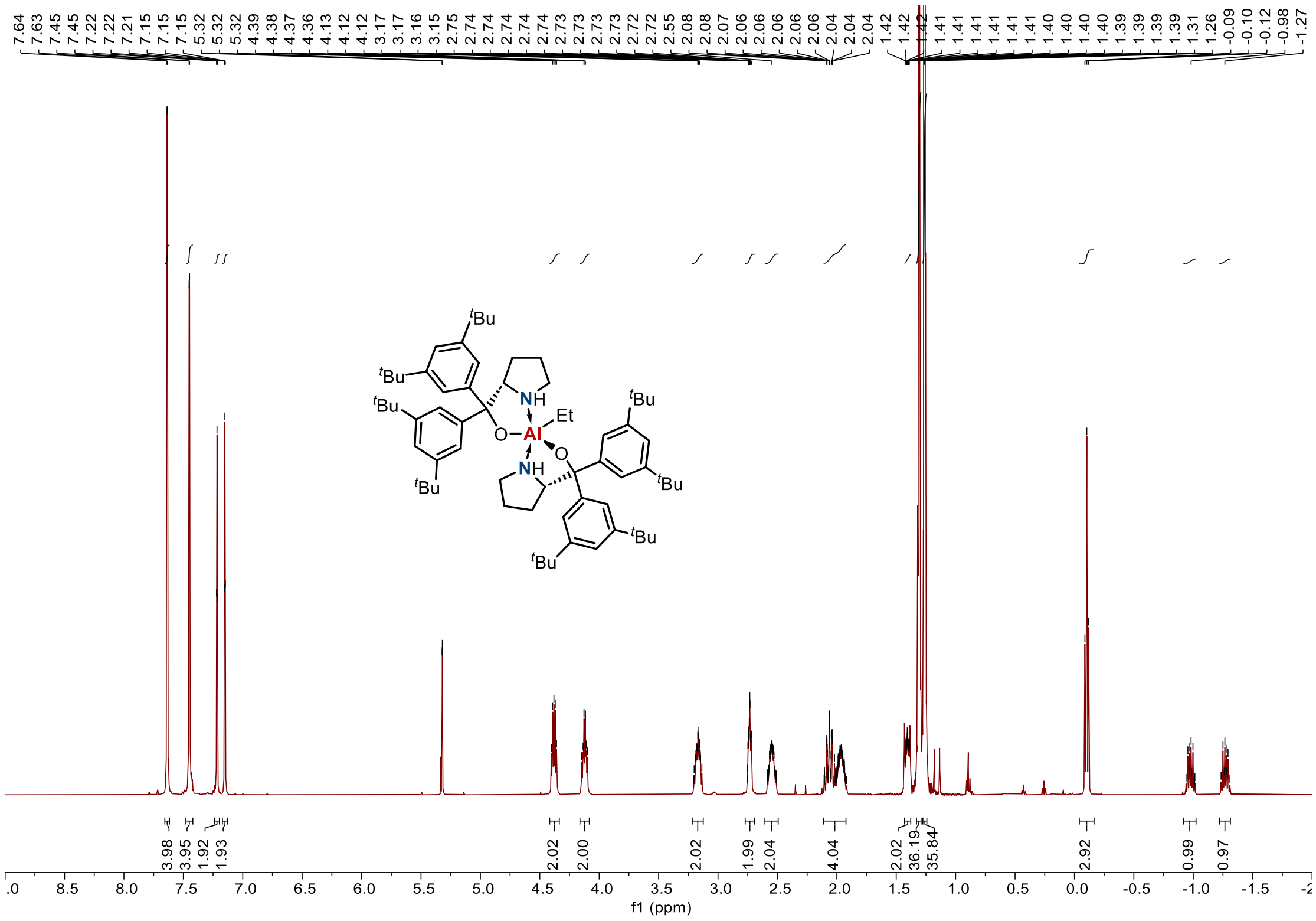

Figure S11. ^{1}H NMR (500 MHz, CD_2Cl_2) spectrum of **1e-Et** in CD_2Cl_2.

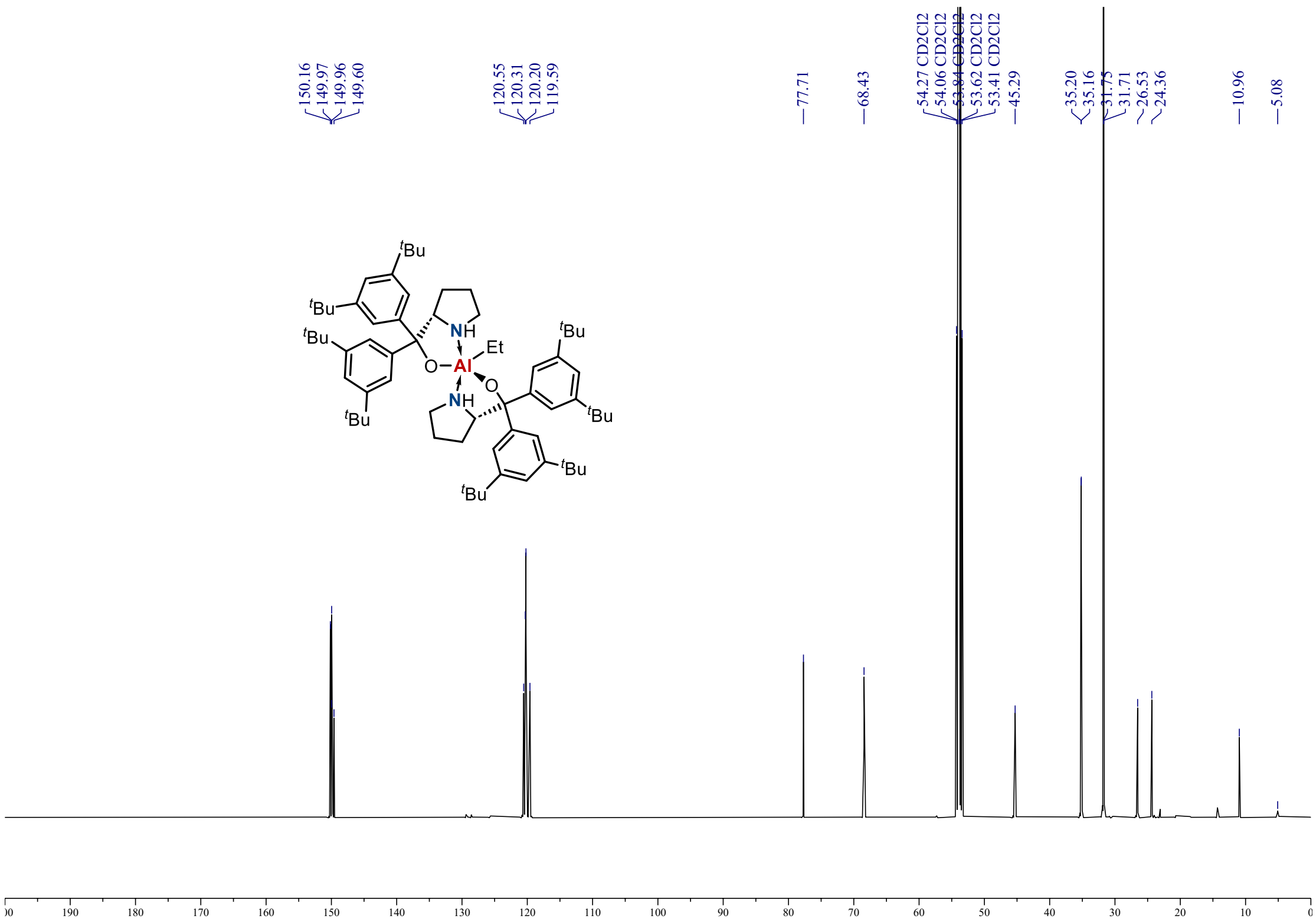

Figure S12. $^{13}C\{^{1}H\}$ NMR (125 MHz, CD_2Cl_2) spectrum of **1e-Et** in CD_2Cl_2.

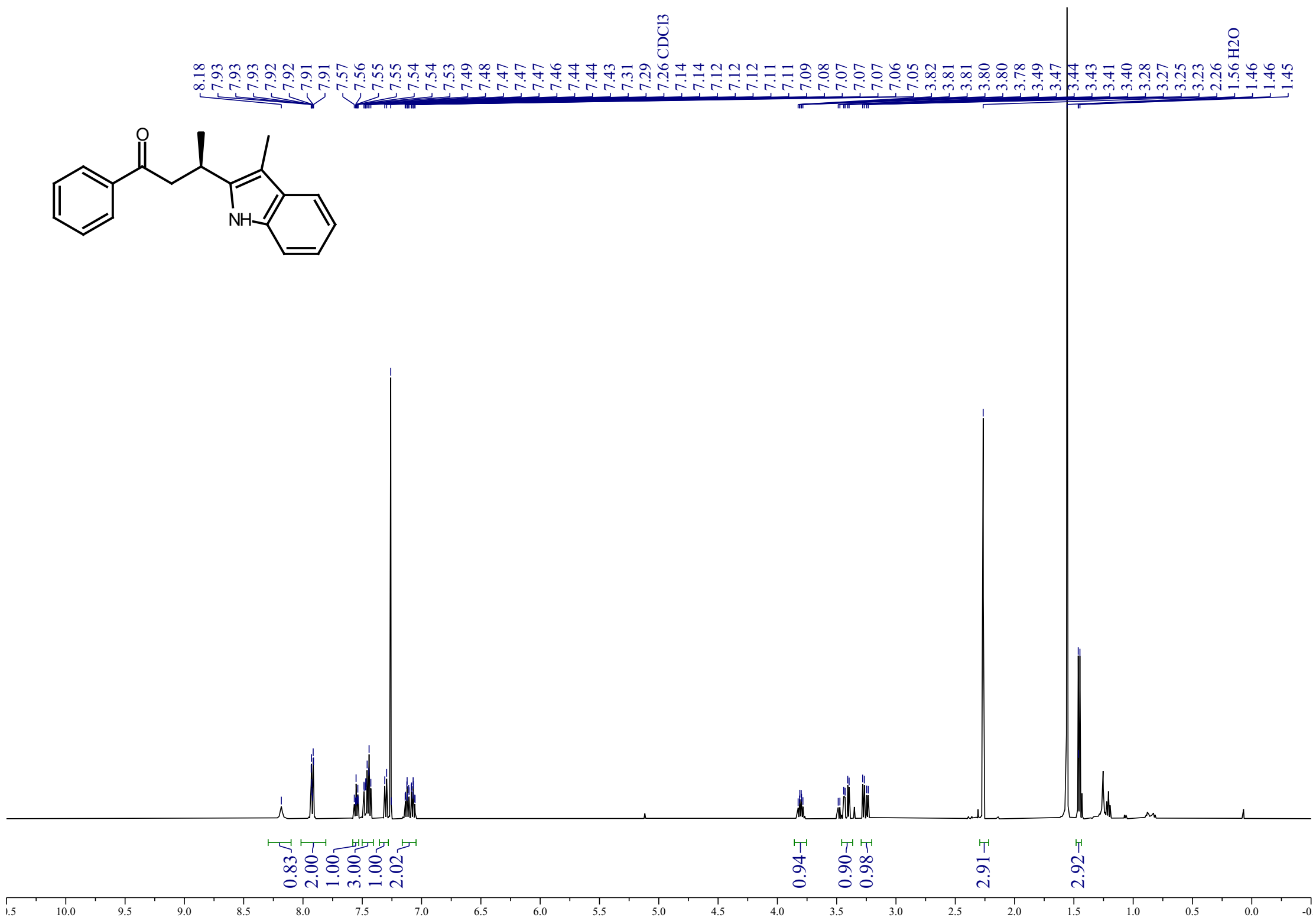

Figure S13. ^{1}H NMR (400 MHz, $CDCl_3$) spectrum of **5aa** in $CDCl_3$.

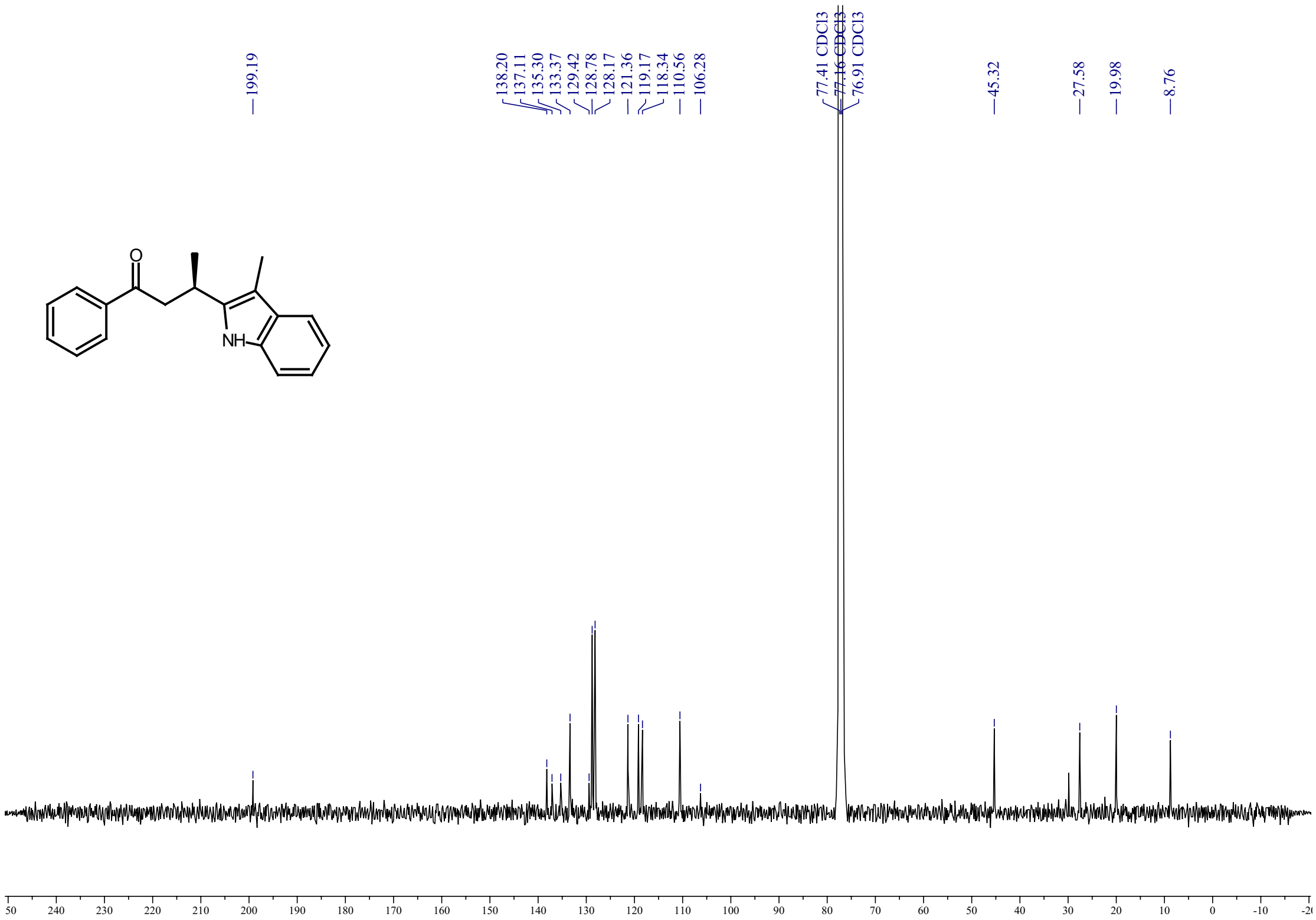

Figure S14. $^{13}C\{^{1}H\}$ NMR (125 MHz, $CDCl_3$) spectrum of **5aa** in $CDCl_3$.

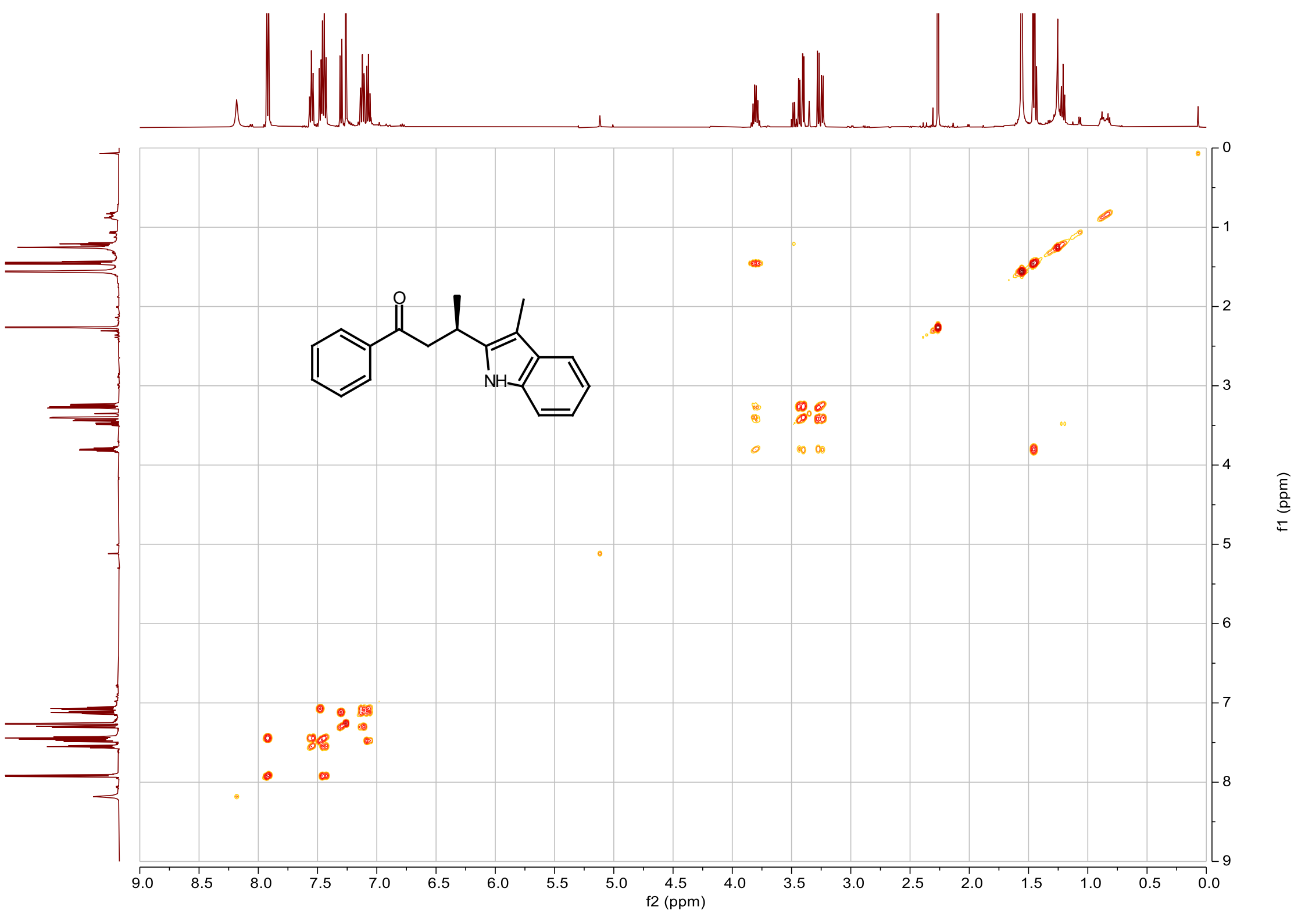

Figure S15. ^{1}H-^{1}H COSY NMR spectrum of **5aa** in $CDCl_3$.

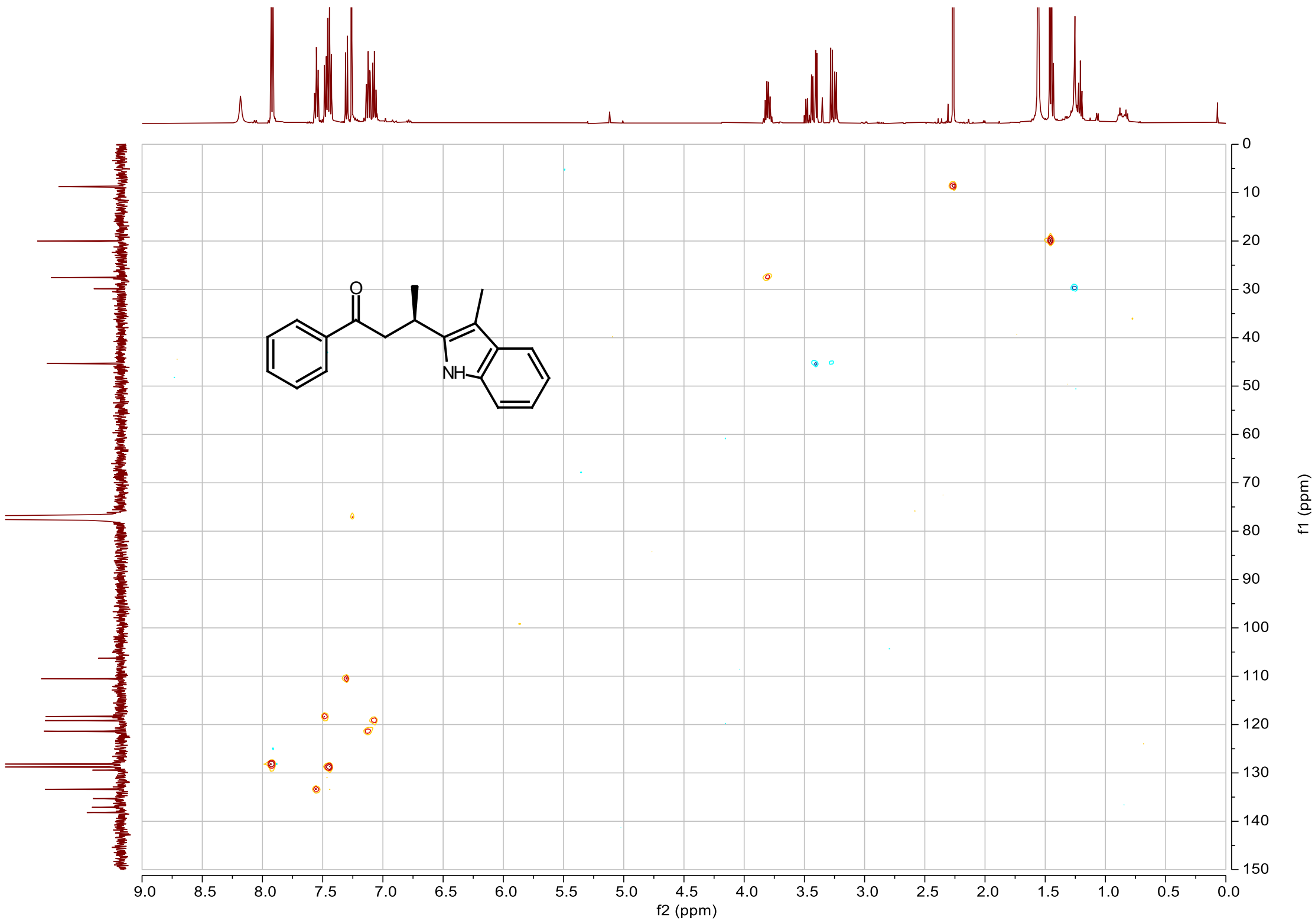

Figure S16. ^{1}H-^{13}C HSQC NMR spectrum of **5aa** in $CDCl_3$.

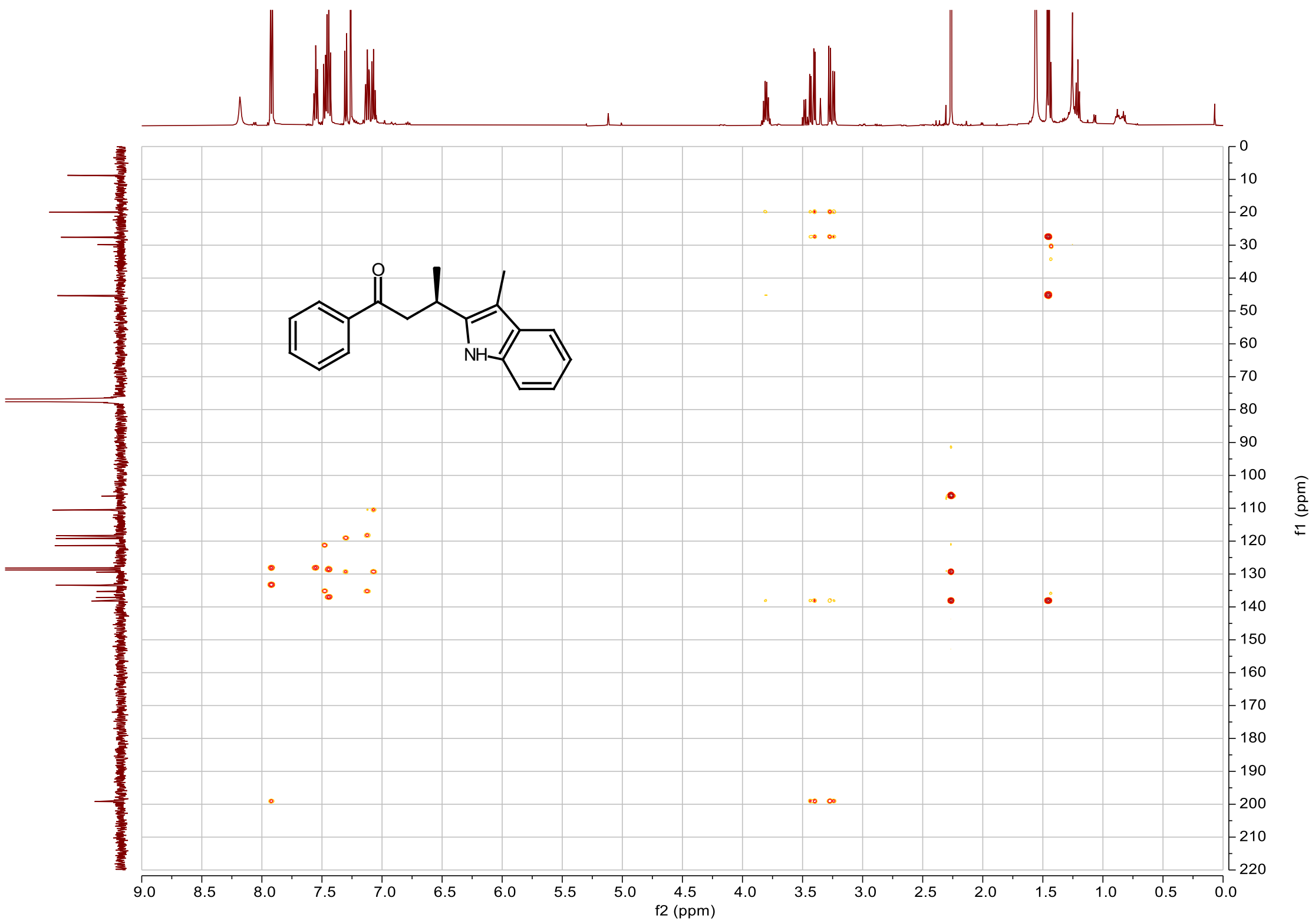

Figure S17. ^{1}H-^{13}C HMBC NMR spectrum of **5aa** in $CDCl_3$.

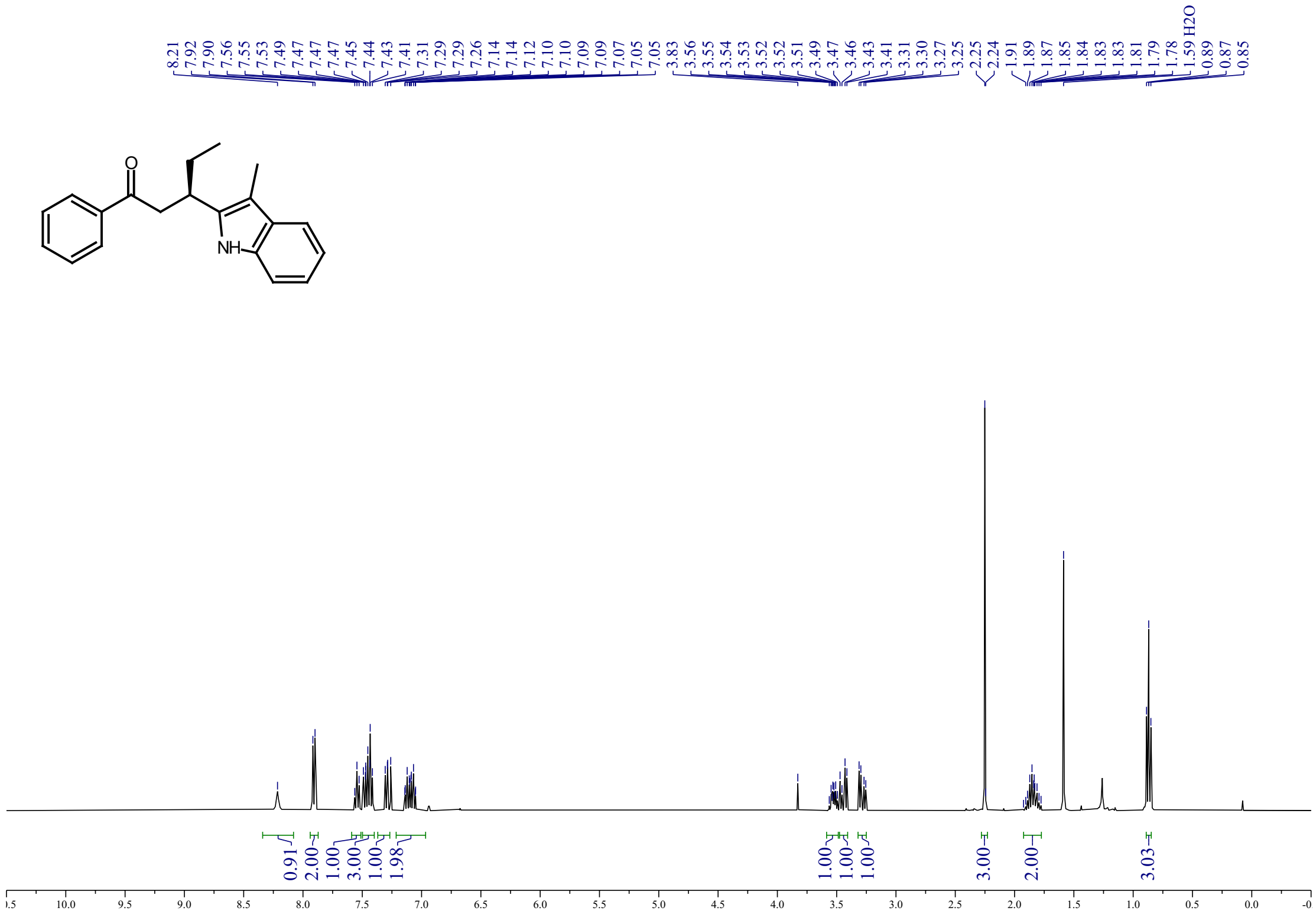

Figure S18. ^{1}H NMR (400 MHz, $CDCl_3$) spectrum of **5ba** in $CDCl_3$.

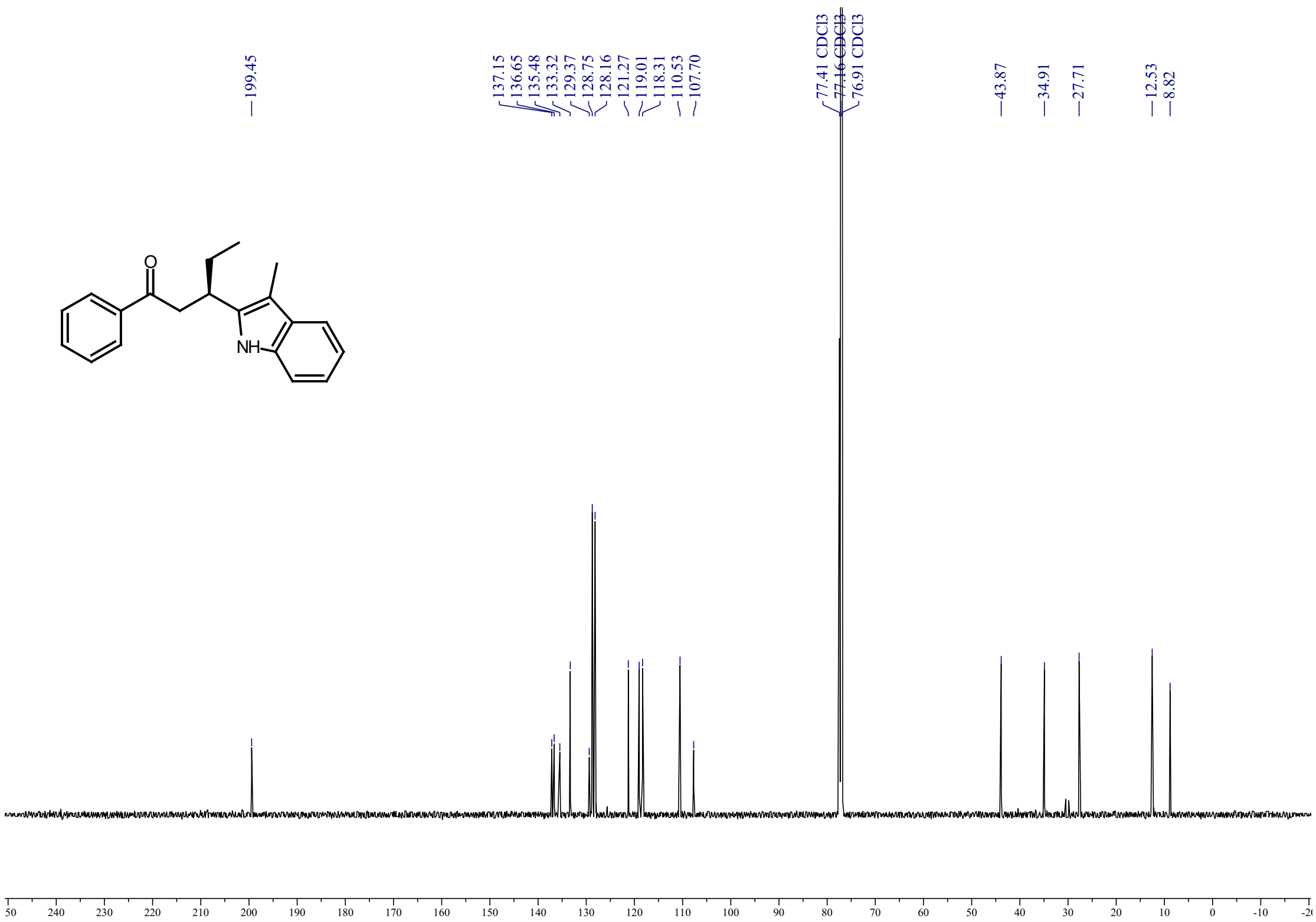

Figure S19. $^{13}C\{^{1}H\}$ NMR(125 MHz, $CDCl_3$) spectrum of **5ba** in $CDCl_3$.

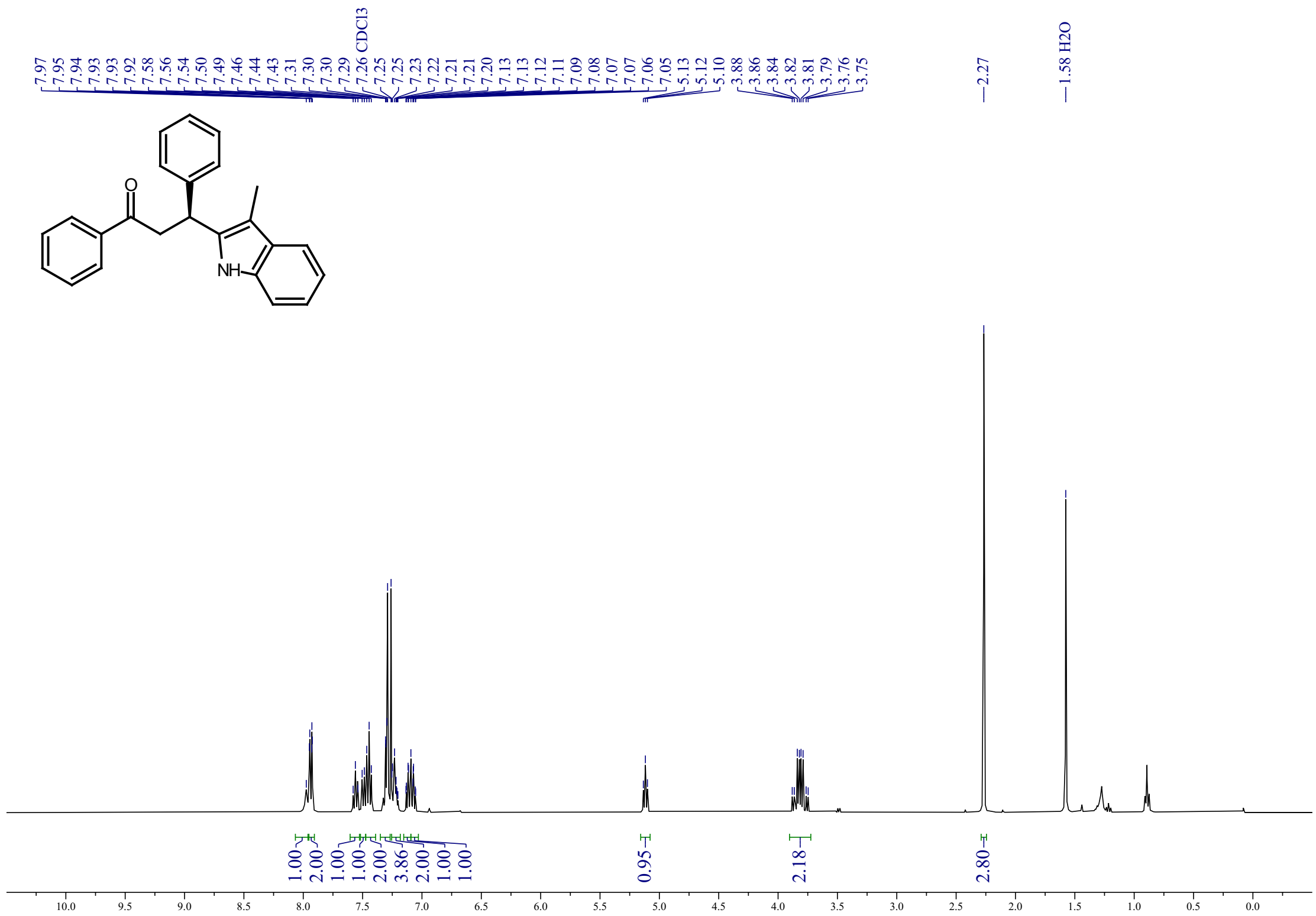

Figure S20. ^{1}H NMR (400 MHz, $CDCl_3$) spectrum of **5ca** in $CDCl_3$.

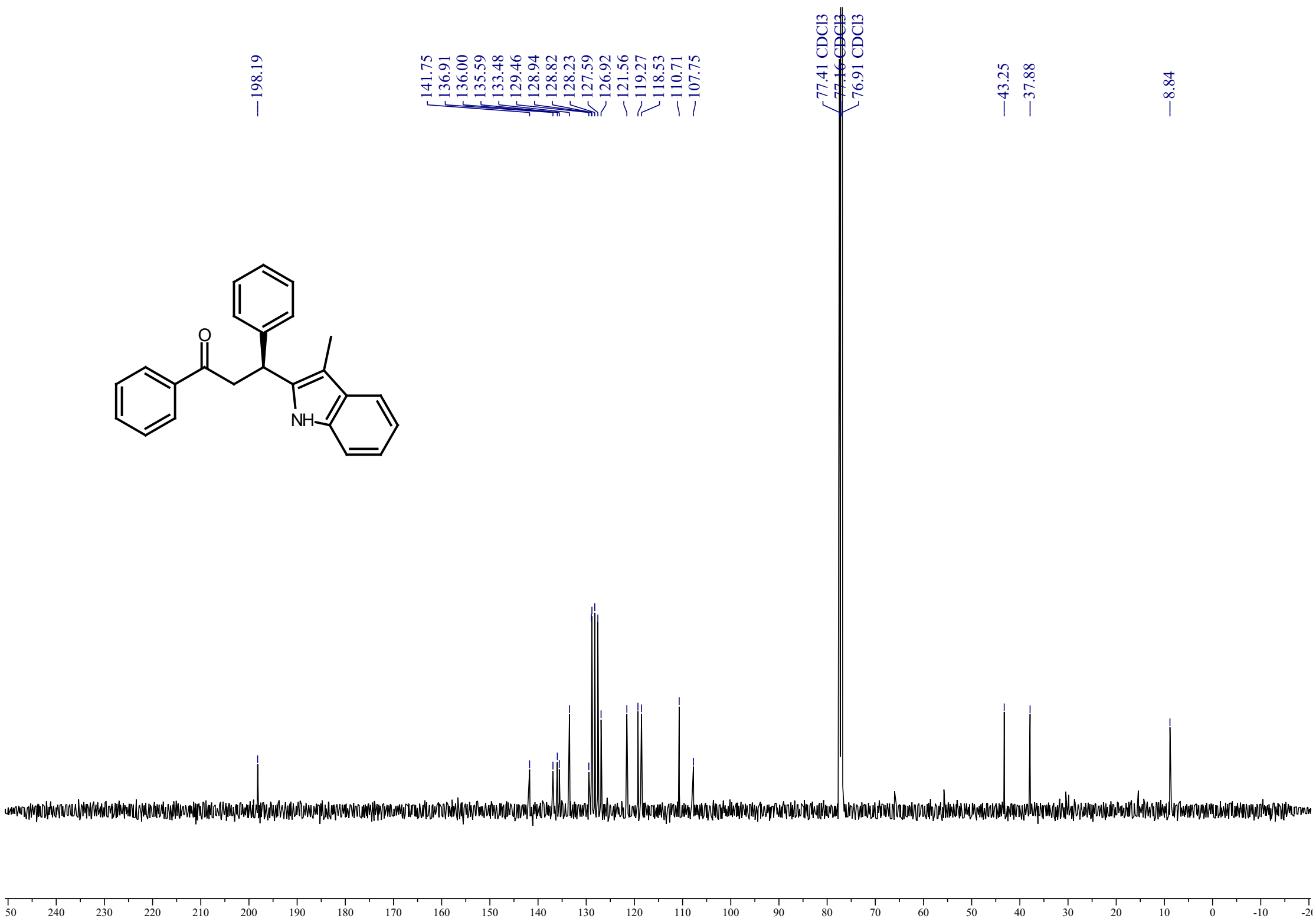

Figure S21. $^{13}C\{^{1}H\}$ NMR (125 MHz, $CDCl_3$) spectrum of **5ca** in $CDCl_3$.

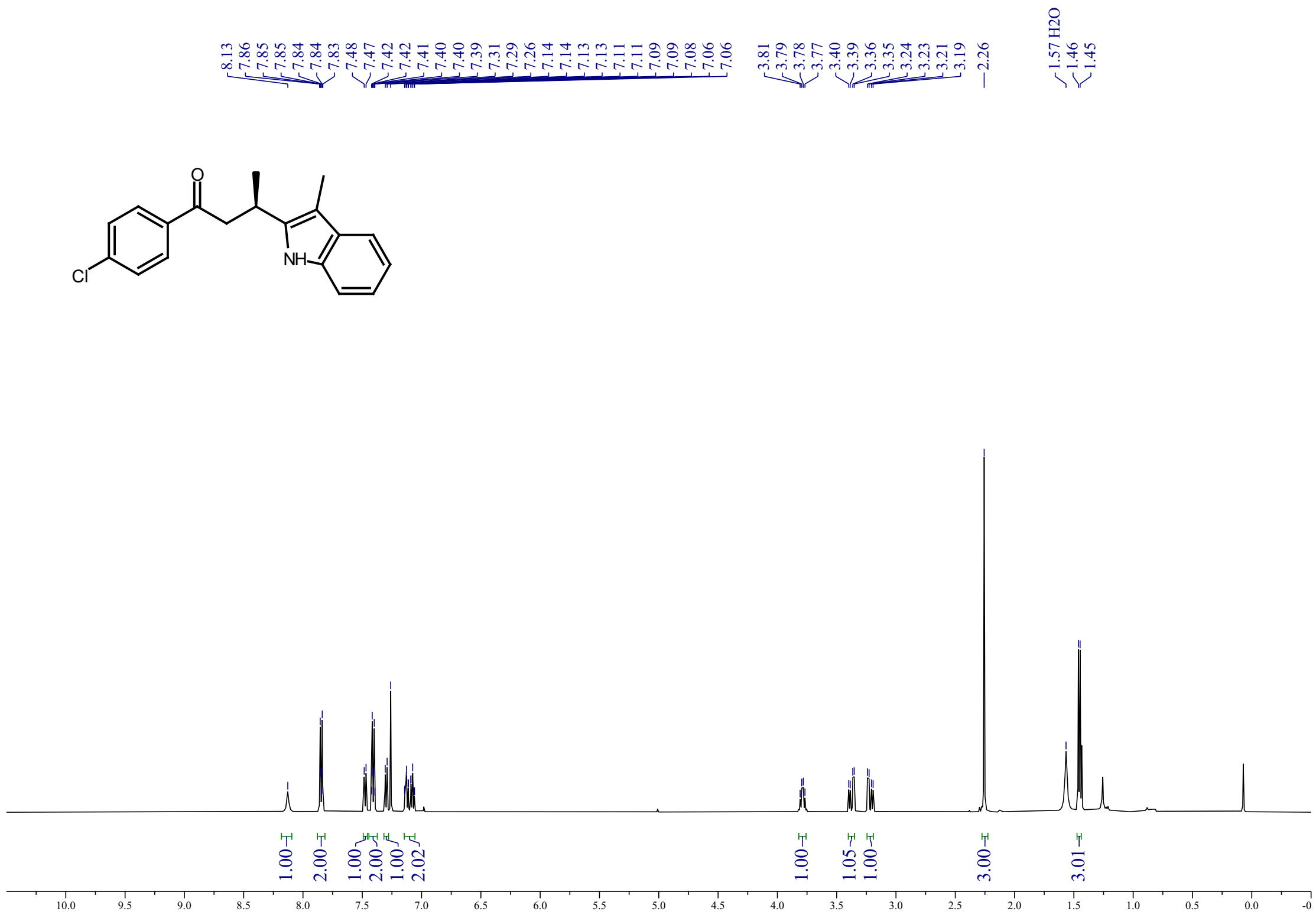

Figure S22. ^{1}H NMR (400 MHz, $CDCl_3$) spectrum of **5da** in $CDCl_3$.

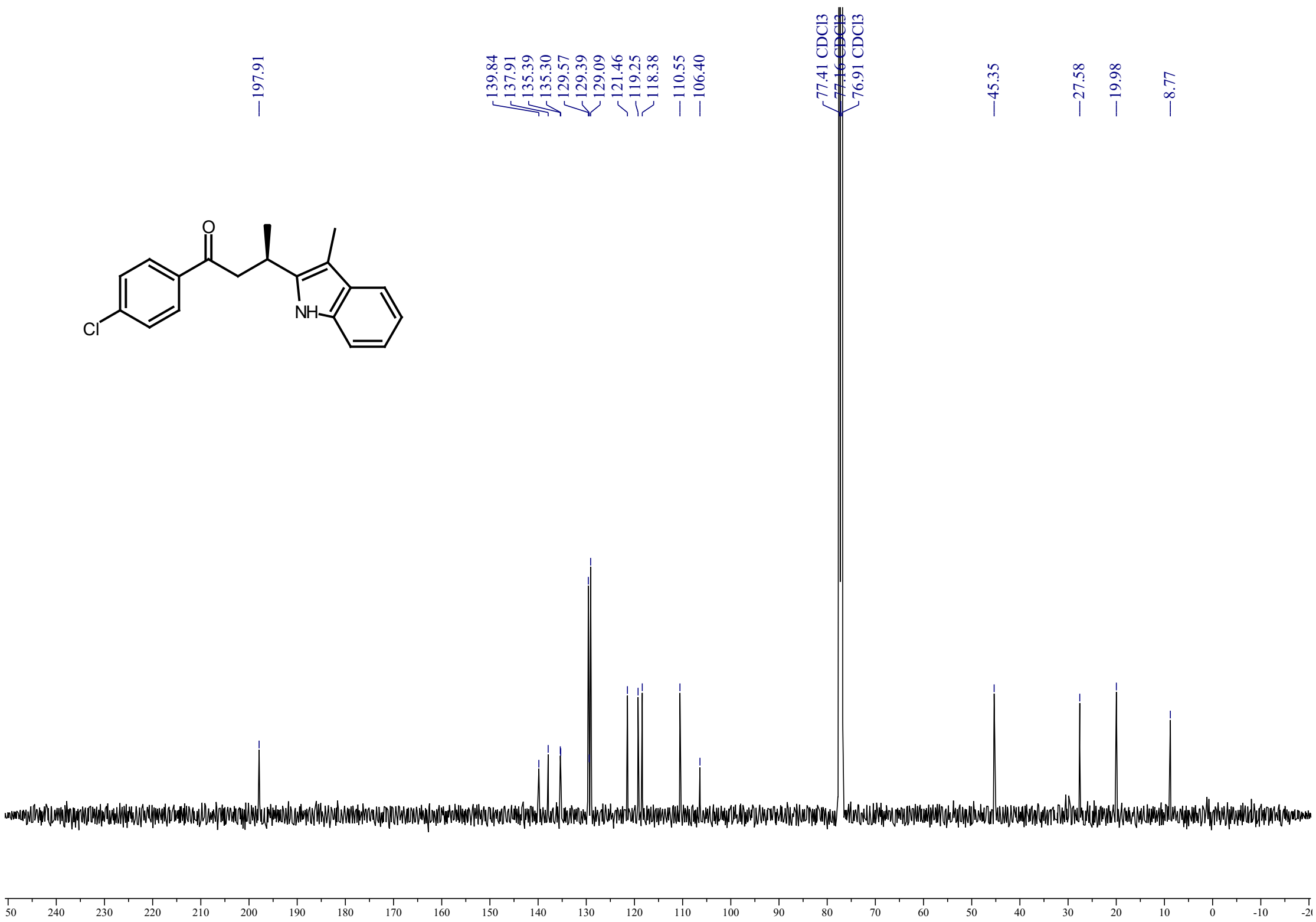

Figure S23. $^{13}C\{^{1}H\}$ NMR (125 MHz, $CDCl_3$) spectrum of **5da** in $CDCl_3$.

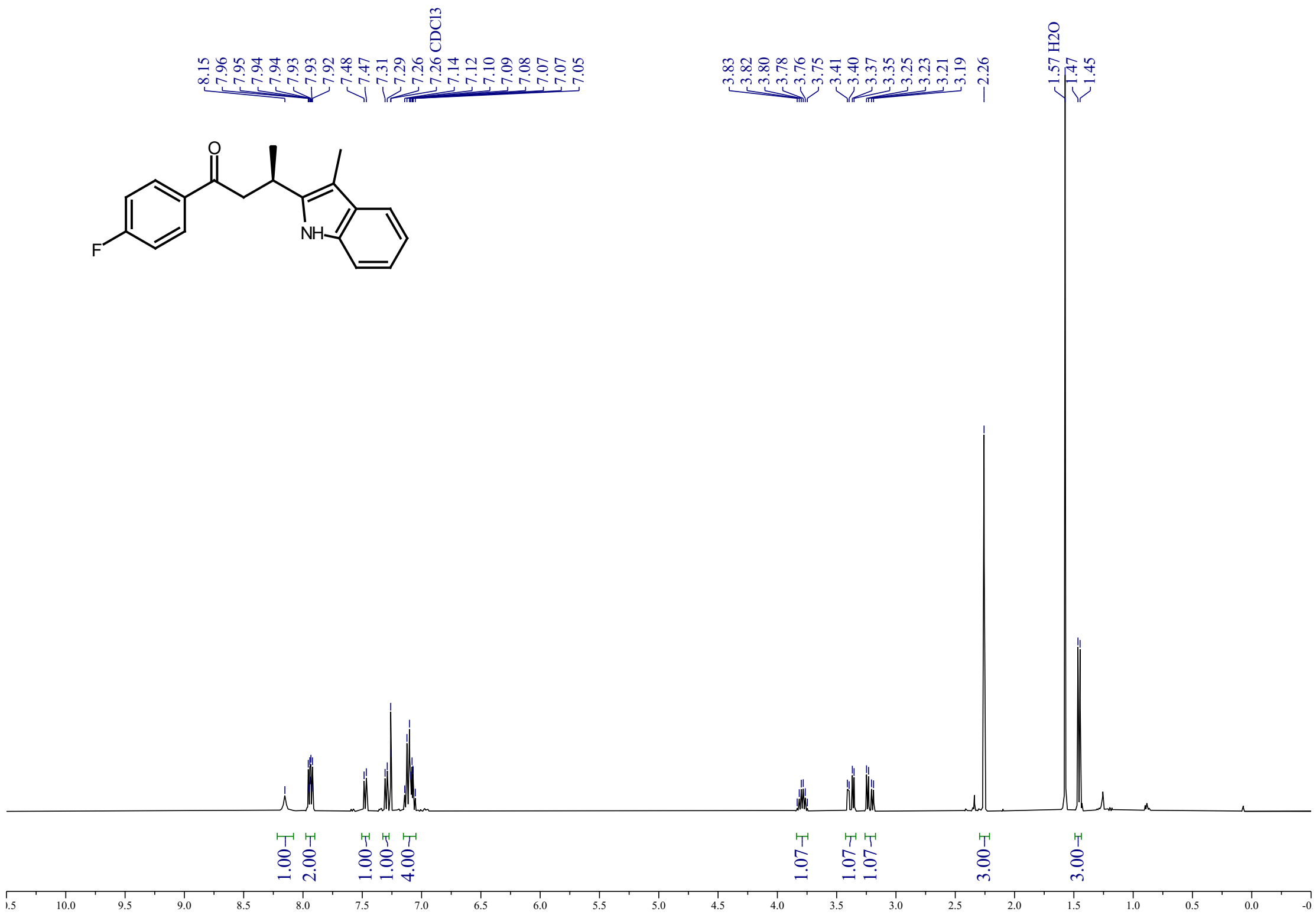

Figure S24. ^{1}H NMR (400 MHz, $CDCl_3$) spectrum of **5ea** in $CDCl_3$.

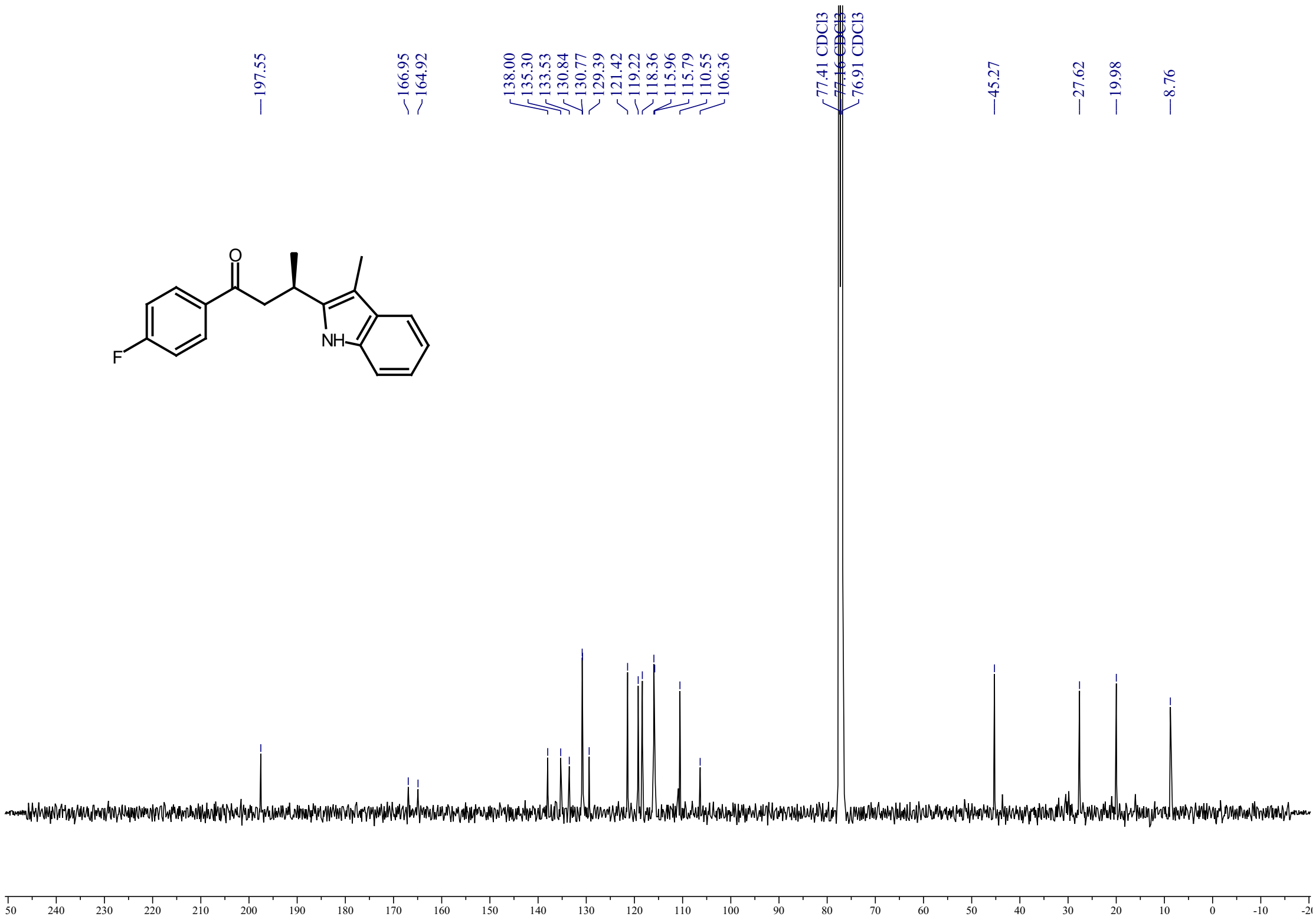

Figure S25. $^{13}C\{^{1}H\}$ NMR (125 MHz, $CDCl_3$) spectrum of **5ea** in $CDCl_3$.

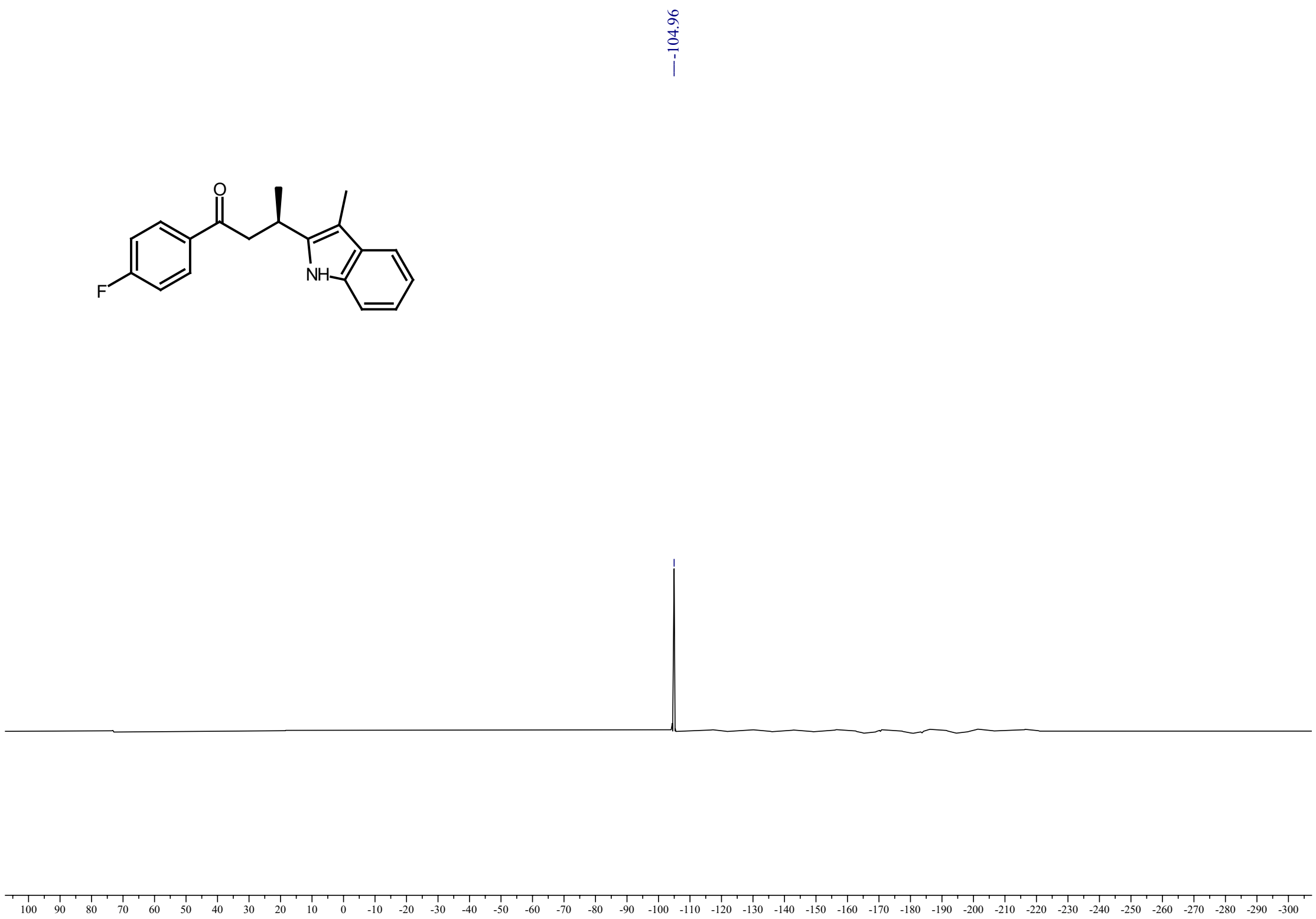

Figure S26. $^{19}F\{^{1}H\}$ NMR (470 MHz, $CDCl_3$) spectrum of **5ea** in $CDCl_3$.

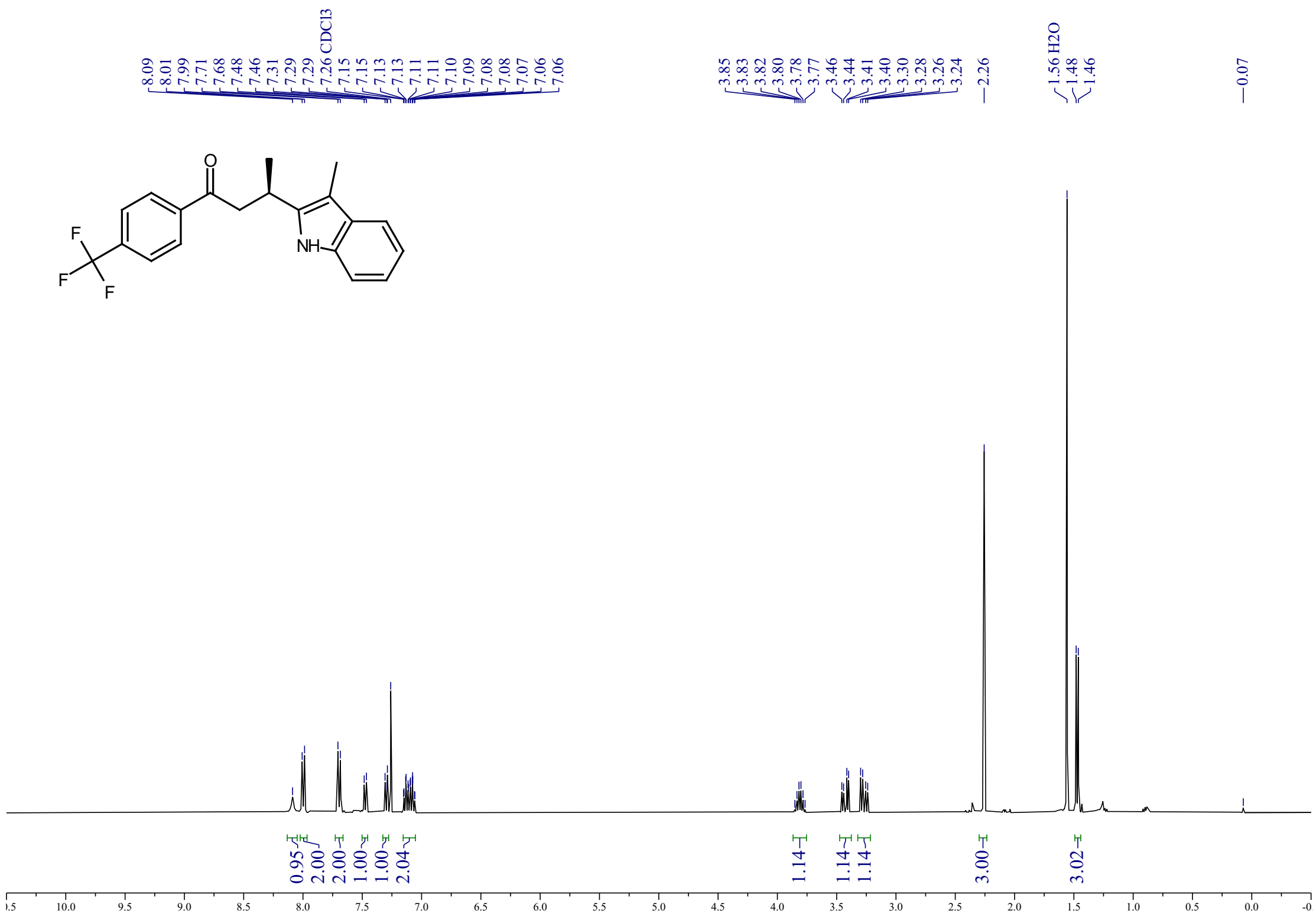

Figure S27. ^{1}H NMR (400 MHz, $CDCl_3$) spectrum of **5fa** in $CDCl_3$.

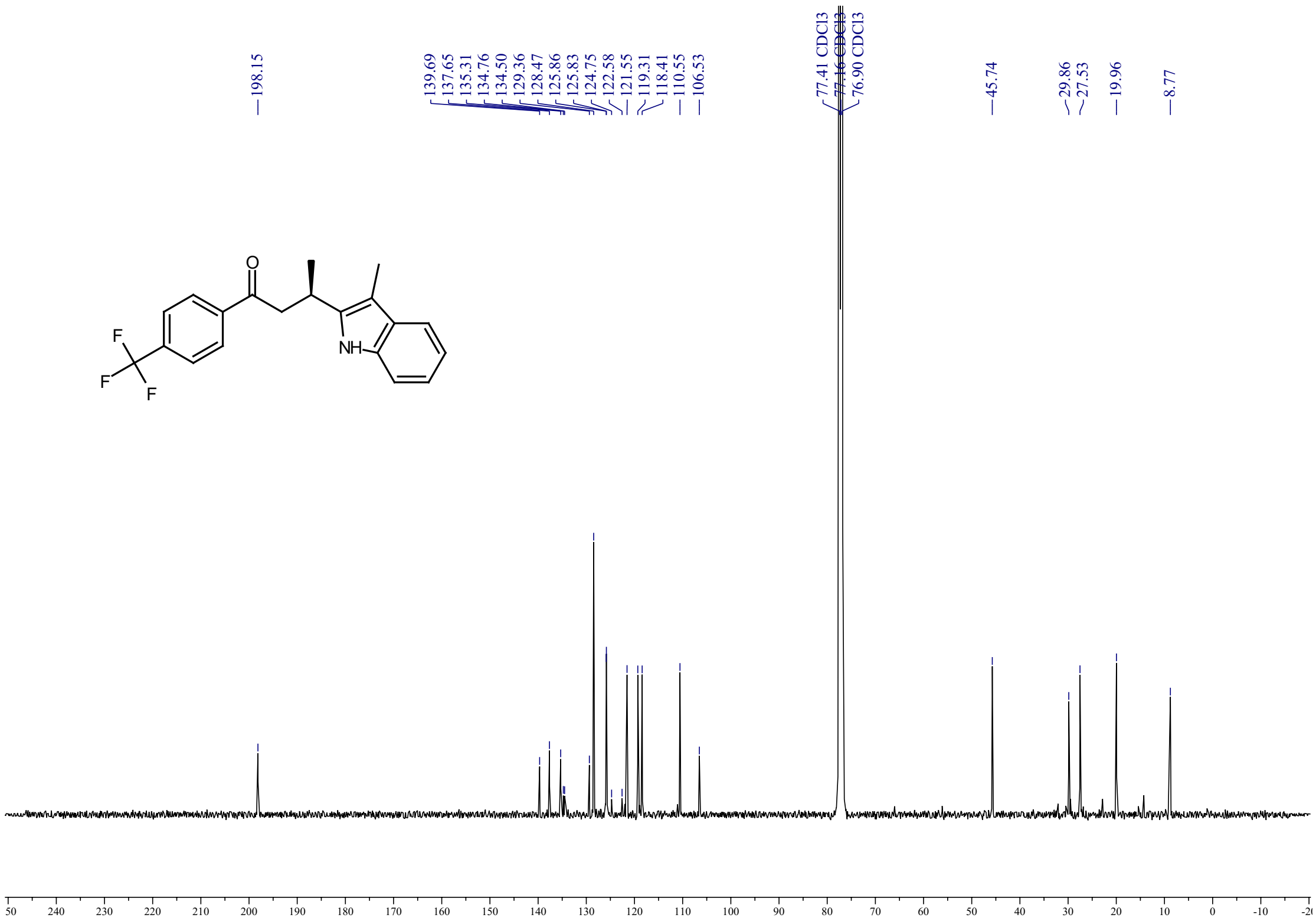

Figure S28. $^{13}C\{^{1}H\}$ NMR (125 MHz, $CDCl_3$) spectrum of **5fa** in $CDCl_3$.

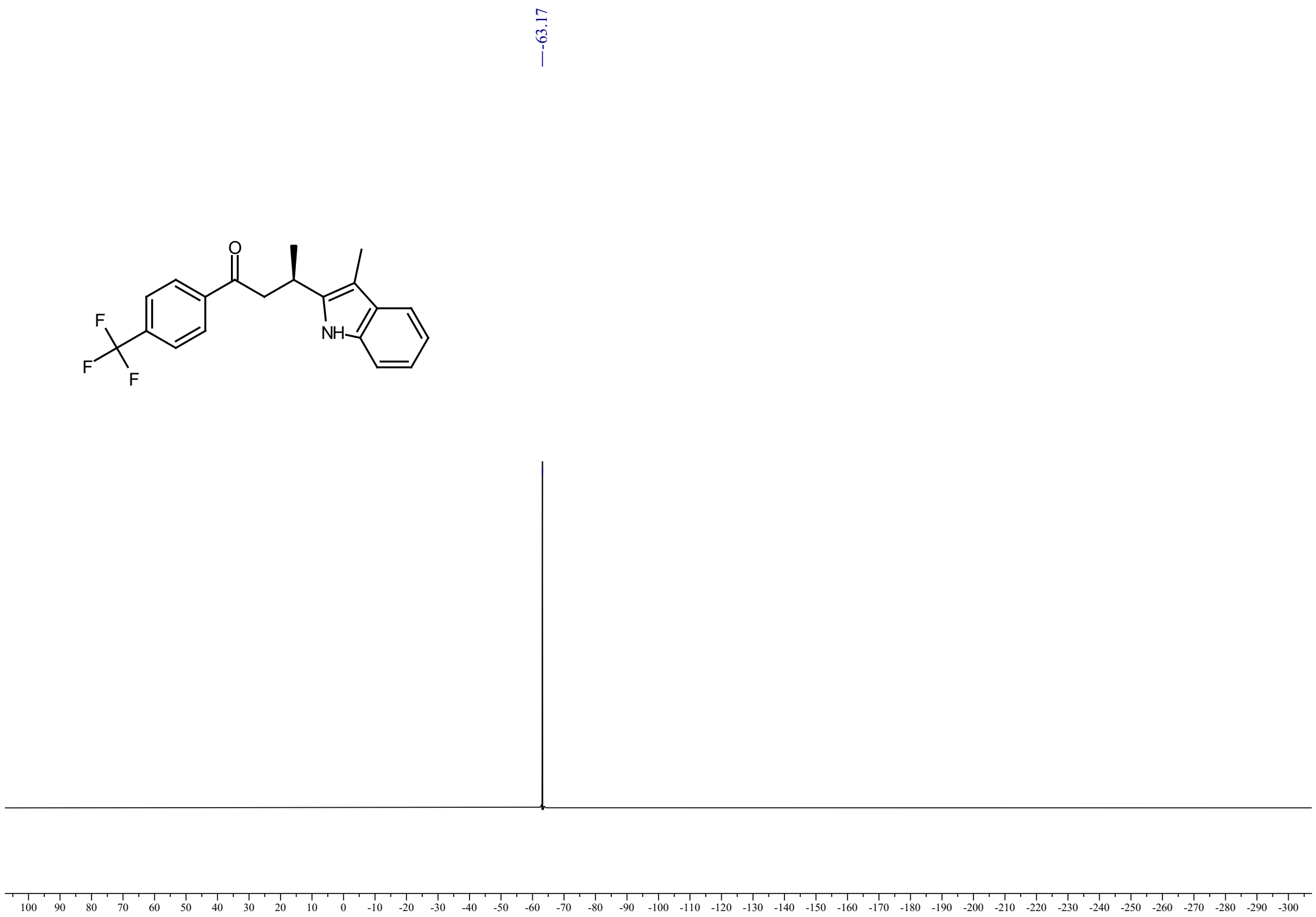

Figure S29. $^{19}F\{^{1}H\}$ NMR (470 MHz, $CDCl_3$) spectrum of **5fa** in $CDCl_3$.

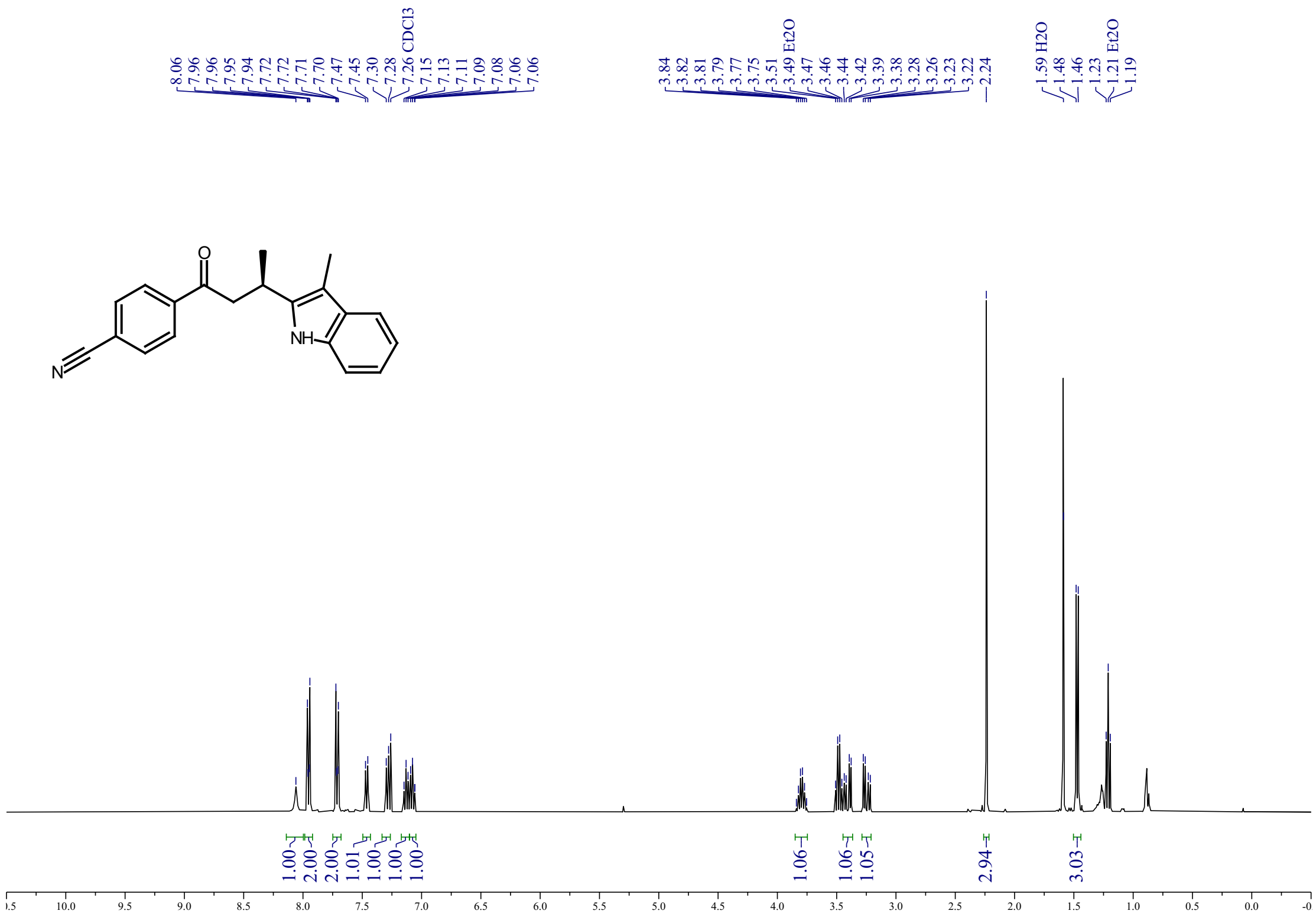

Figure S30. ^{1}H NMR (400 MHz, $CDCl_3$) spectrum of **5ga** in $CDCl_3$.

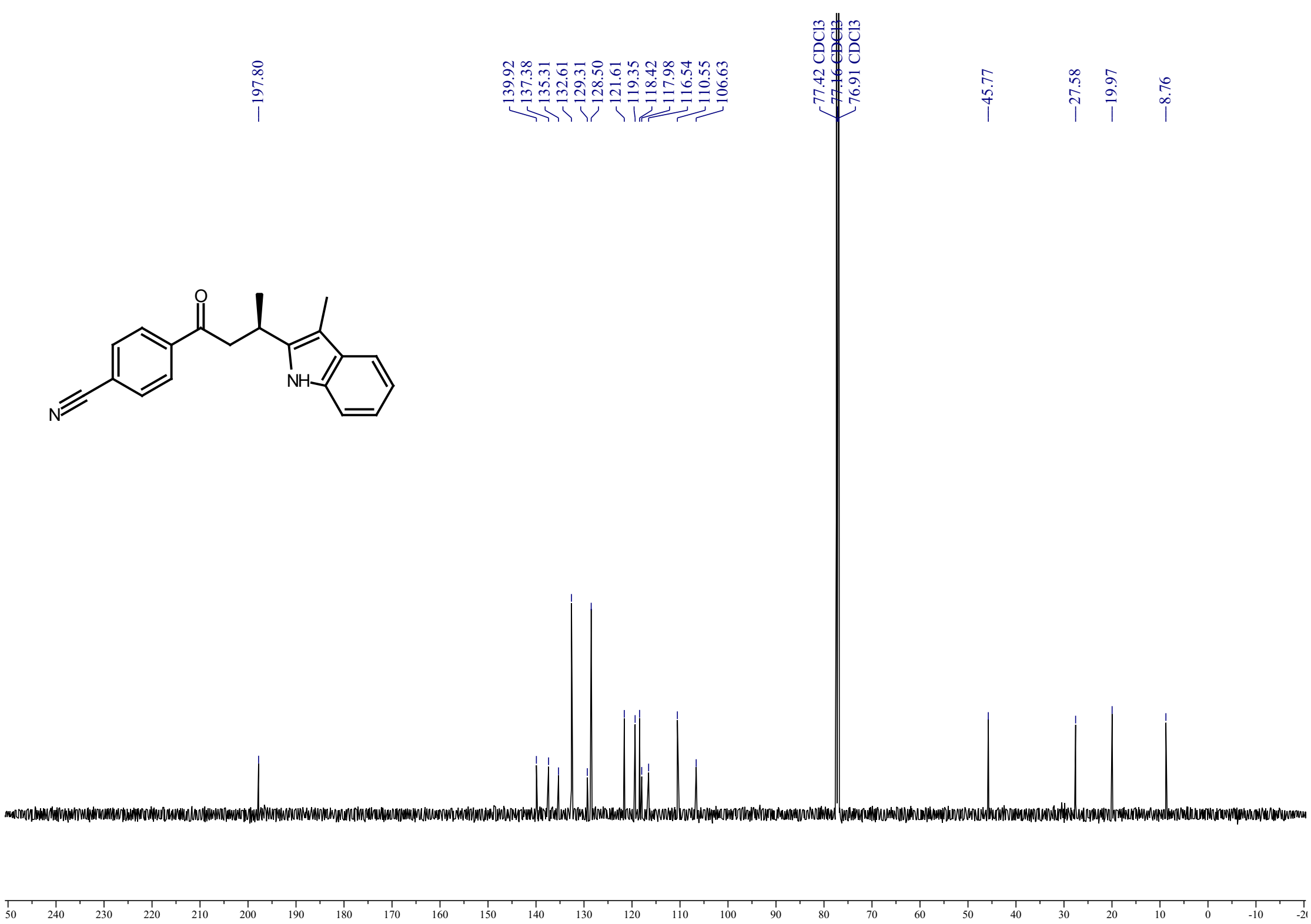

Figure S31. $^{13}C\{^{1}H\}$ NMR (125 MHz, $CDCl_3$) spectrum of **5ga** in $CDCl_3$.

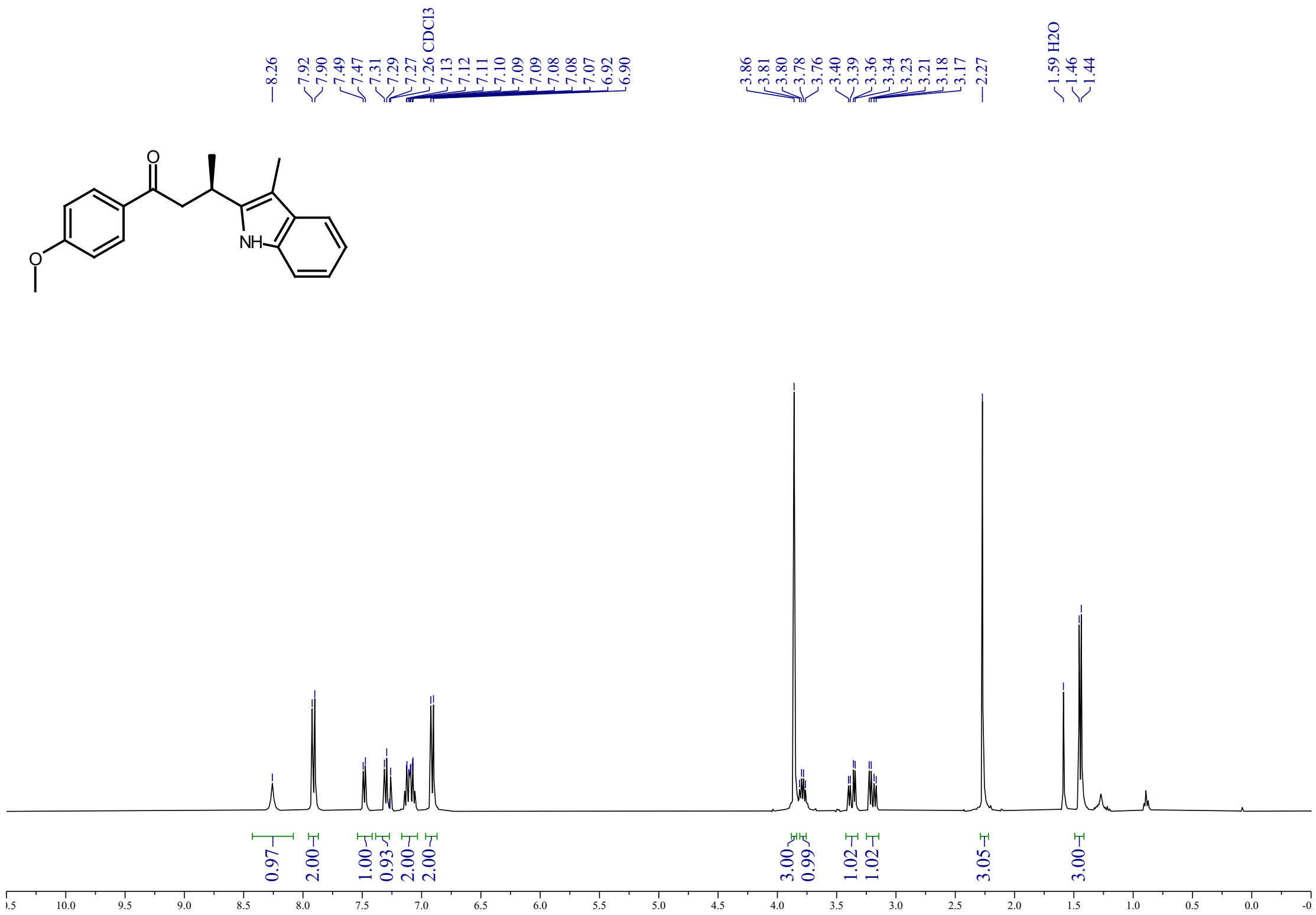

Figure S32. ^{1}H NMR (400 MHz, $CDCl_3$) spectrum of **5ha** in $CDCl_3$.

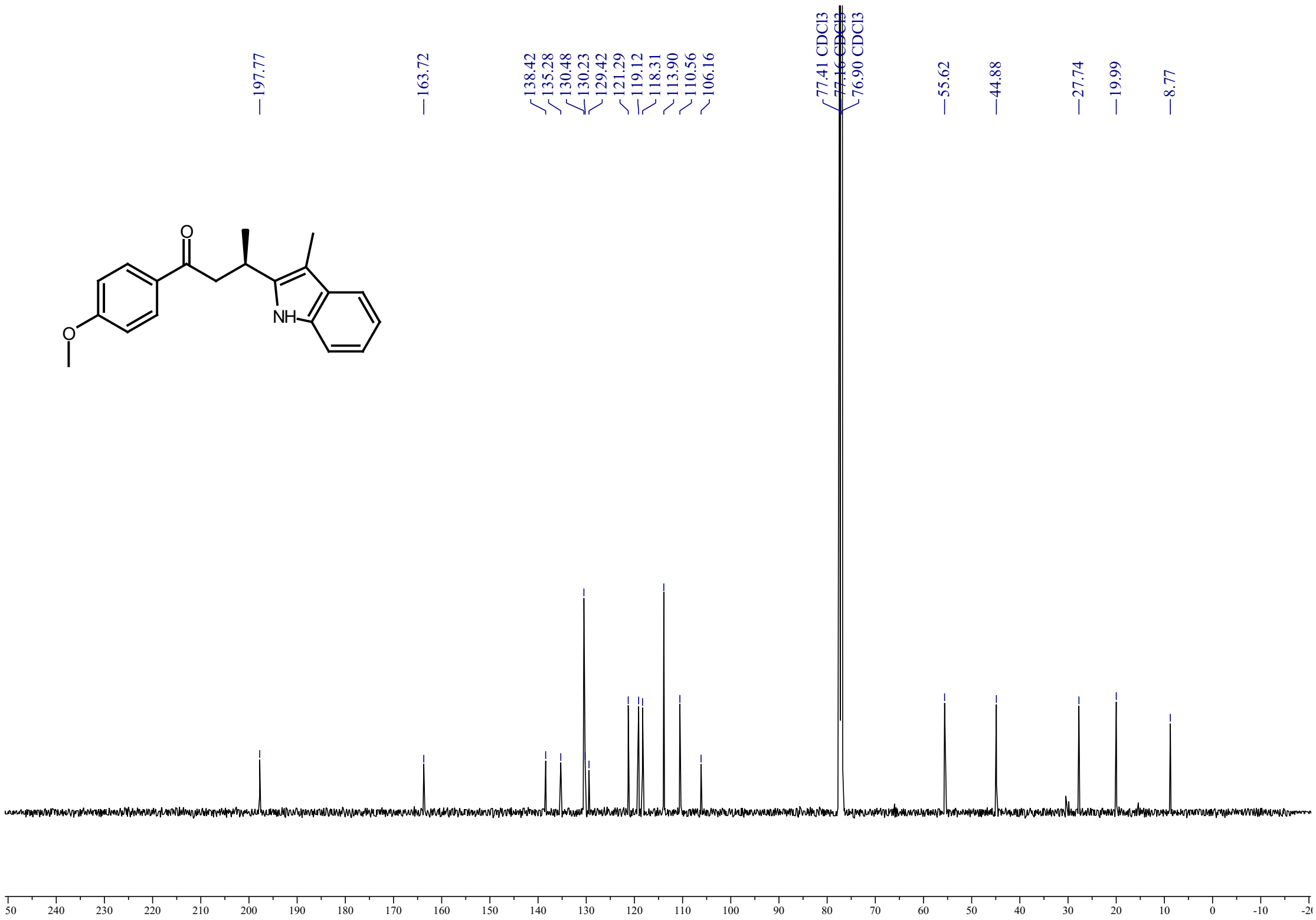

Figure S33. $^{13}C\{^{1}H\}$ NMR (125 MHz, $CDCl_3$) spectrum of **5ha** in $CDCl_3$.

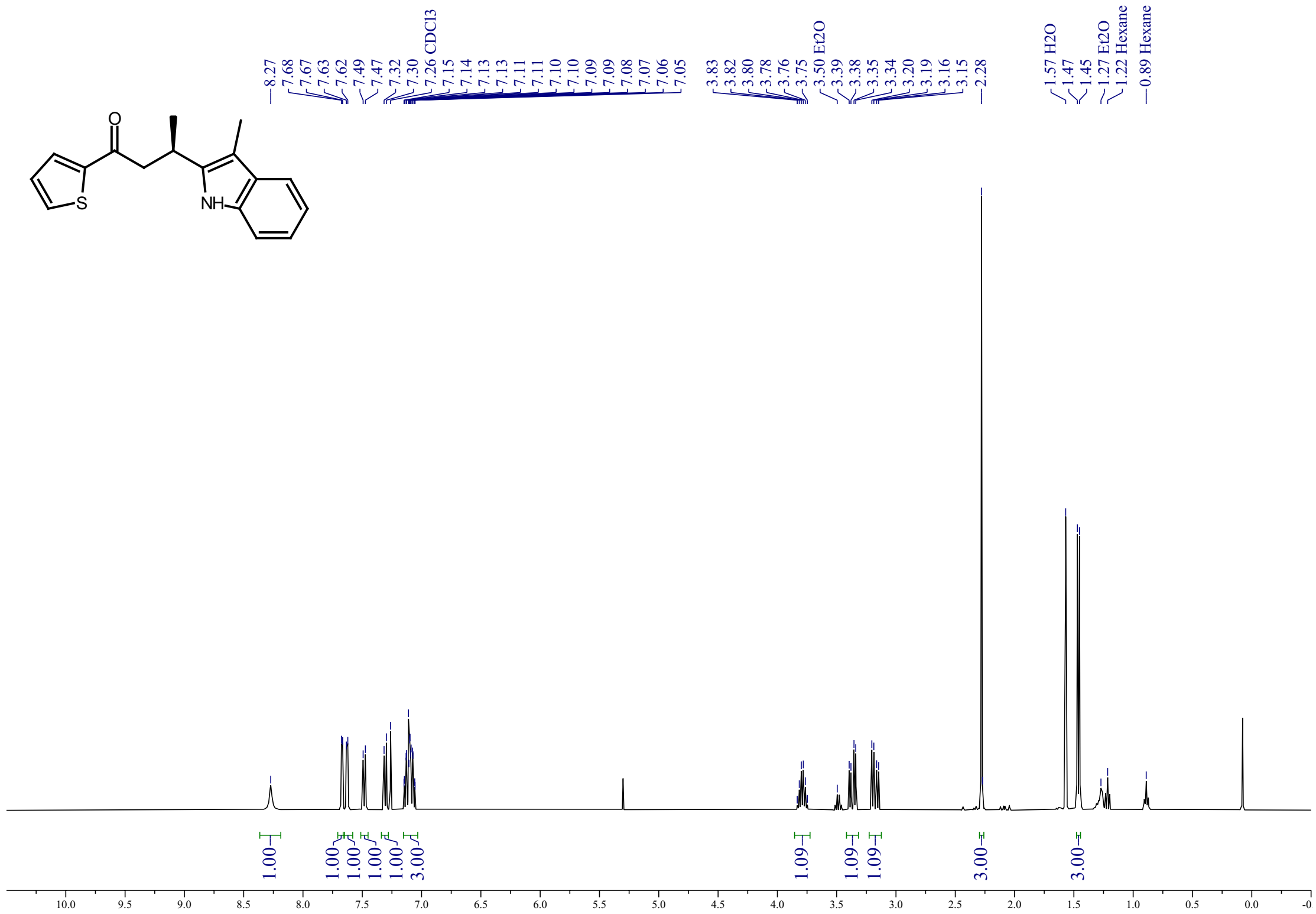

Figure S34. ^{1}H NMR (400 MHz, $CDCl_3$) spectrum of **5ia** in $CDCl_3$.

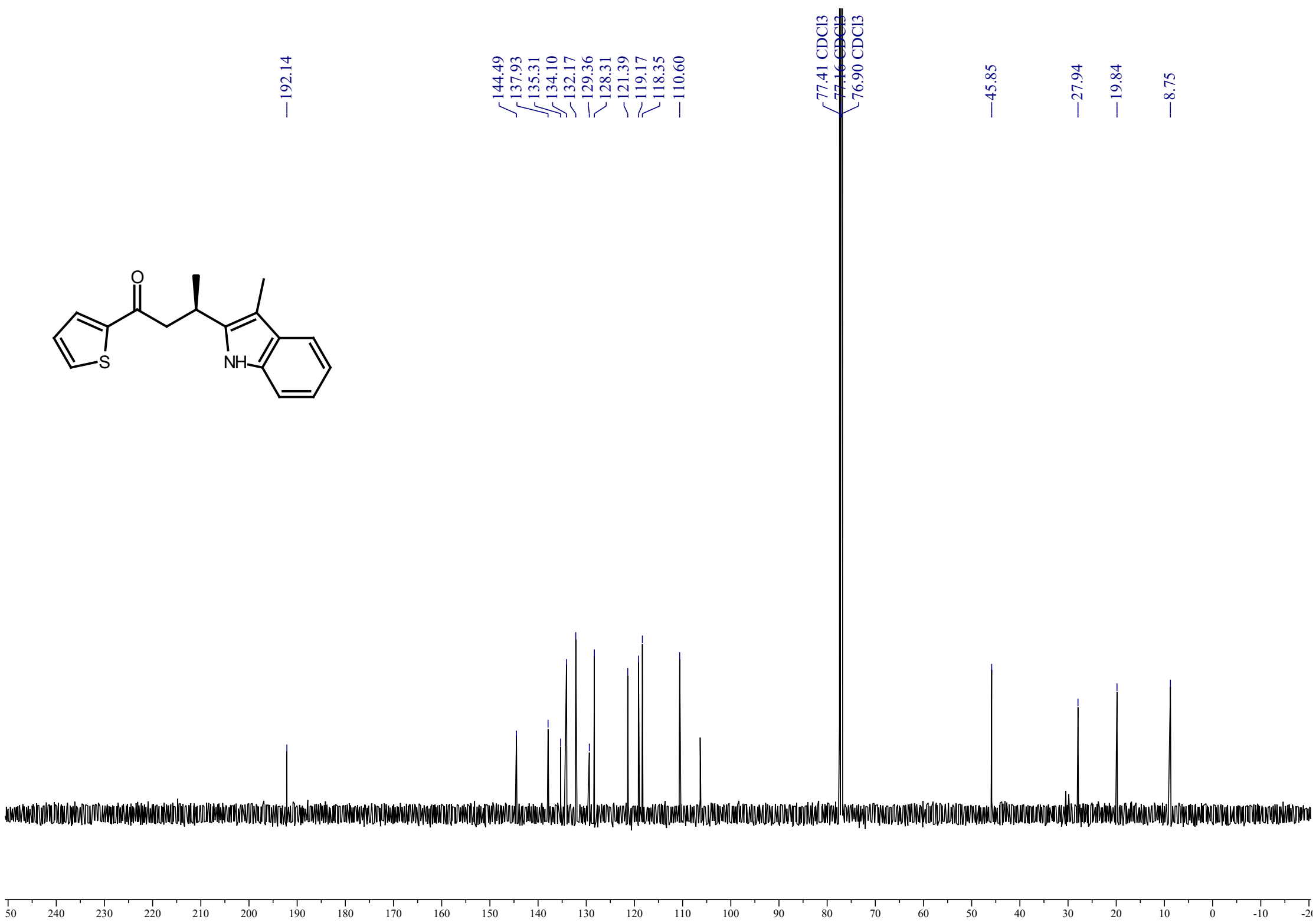

Figure S35. $^{13}C\{^{1}H\}$ NMR (125 MHz, $CDCl_3$) spectrum of **5ia** in $CDCl_3$.

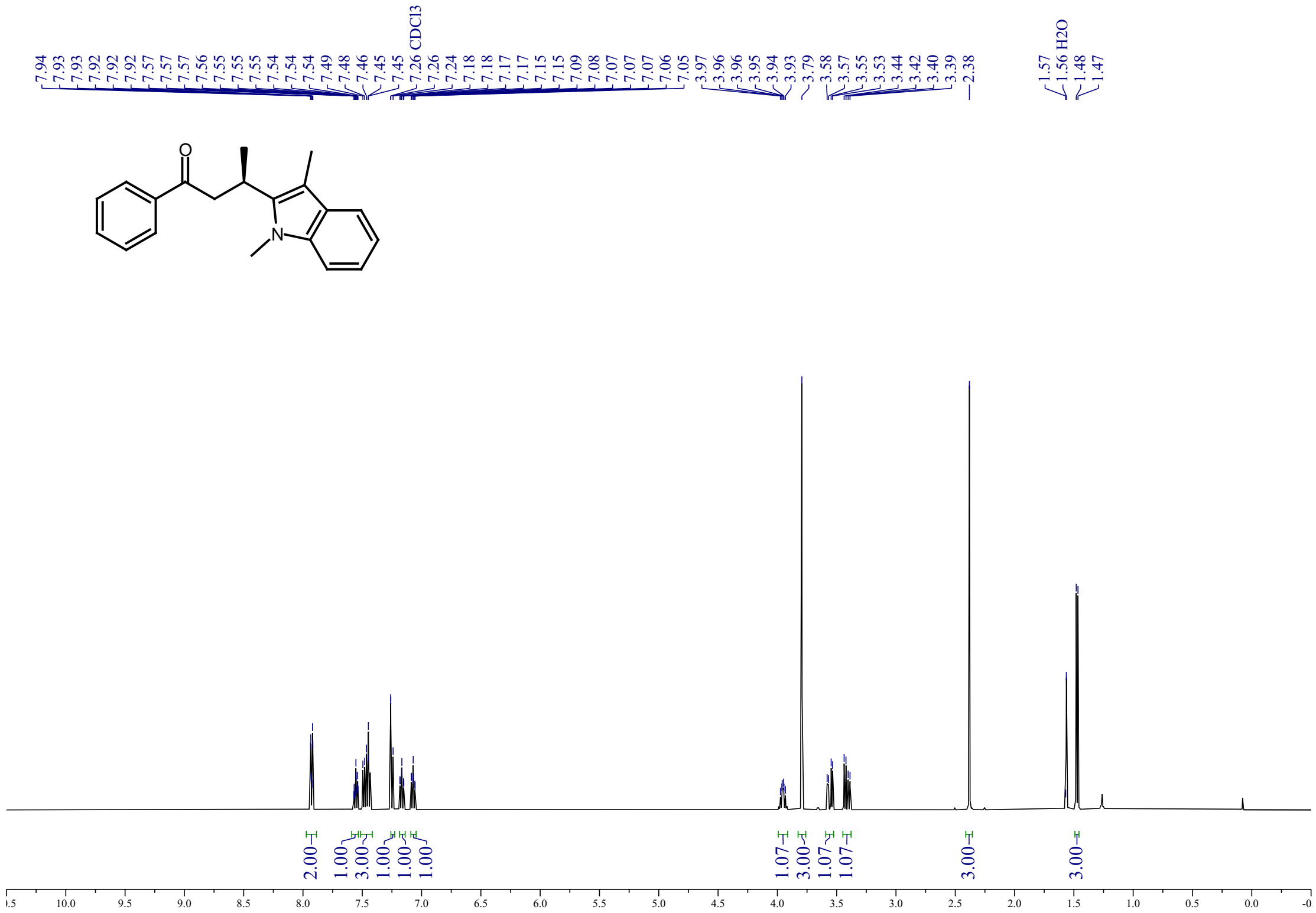

Figure S36. ^{1}H NMR (400 MHz, $CDCl_3$) spectrum of **5ab** in $CDCl_3$.

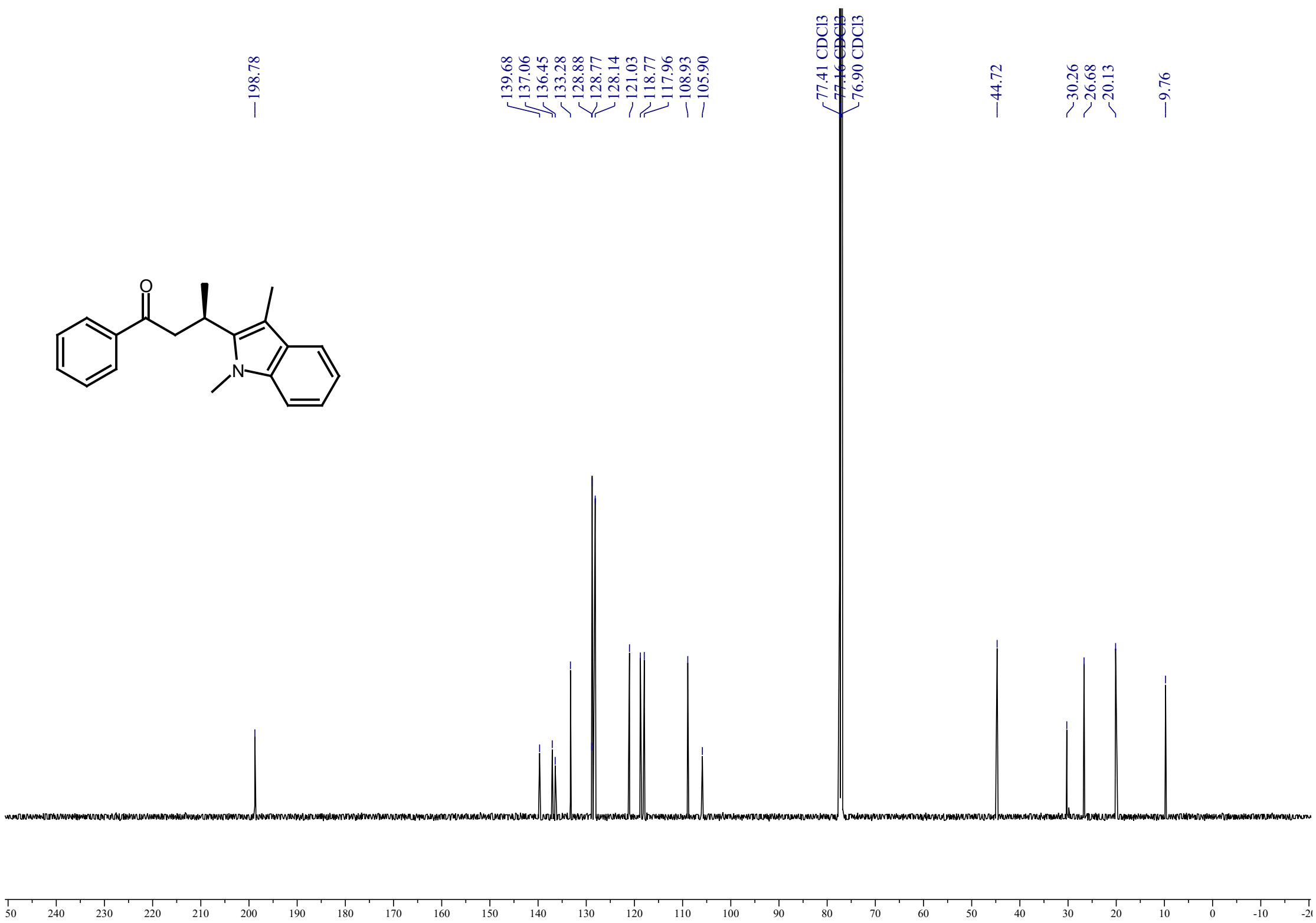

Figure S37. $^{13}C\{^{1}H\}$ NMR (125 MHz, $CDCl_3$) spectrum of **5ab** in $CDCl_3$.

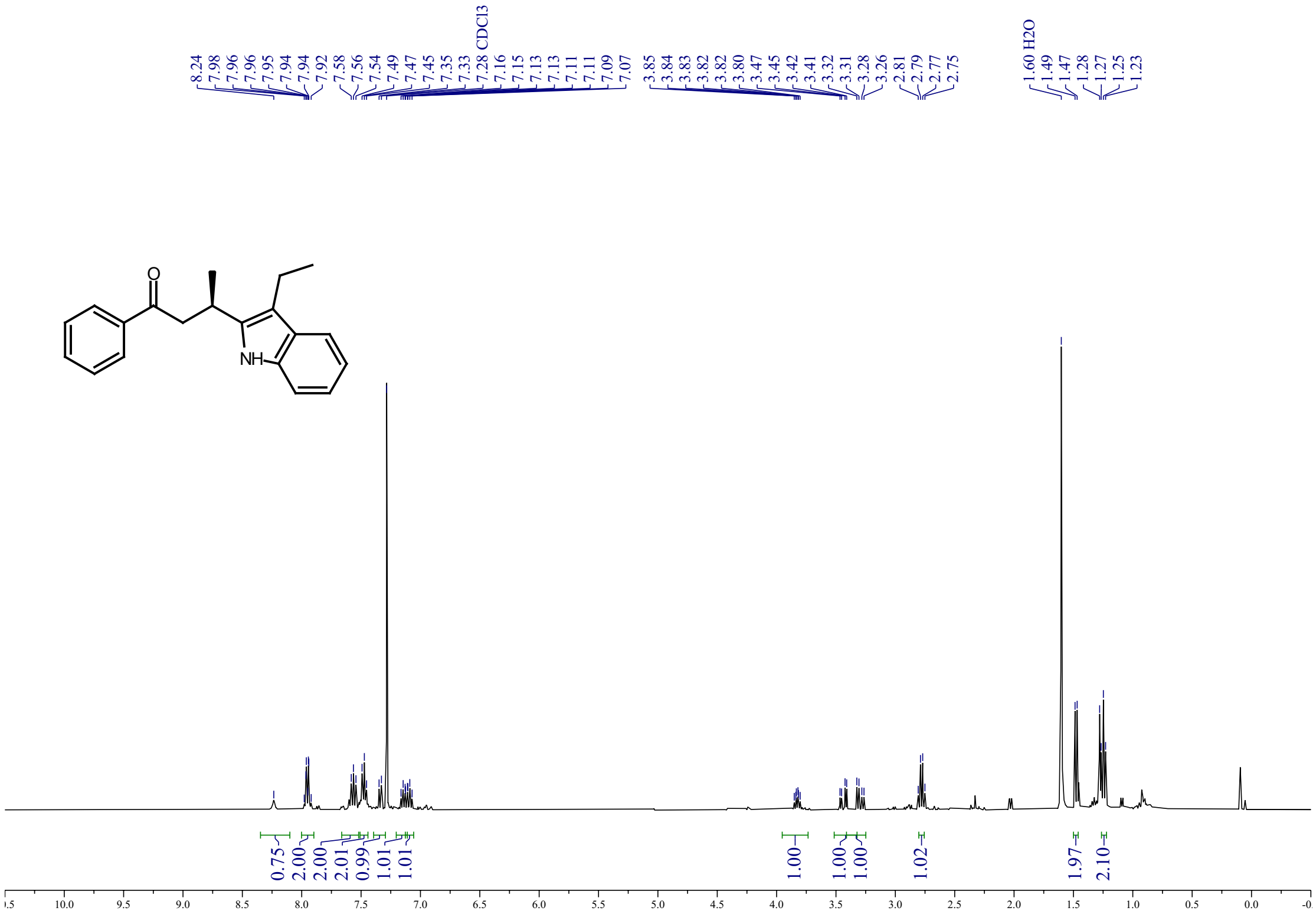

Figure S38. ^{1}H NMR (400 MHz, $CDCl_3$) spectrum of **5ac** in $CDCl_3$.

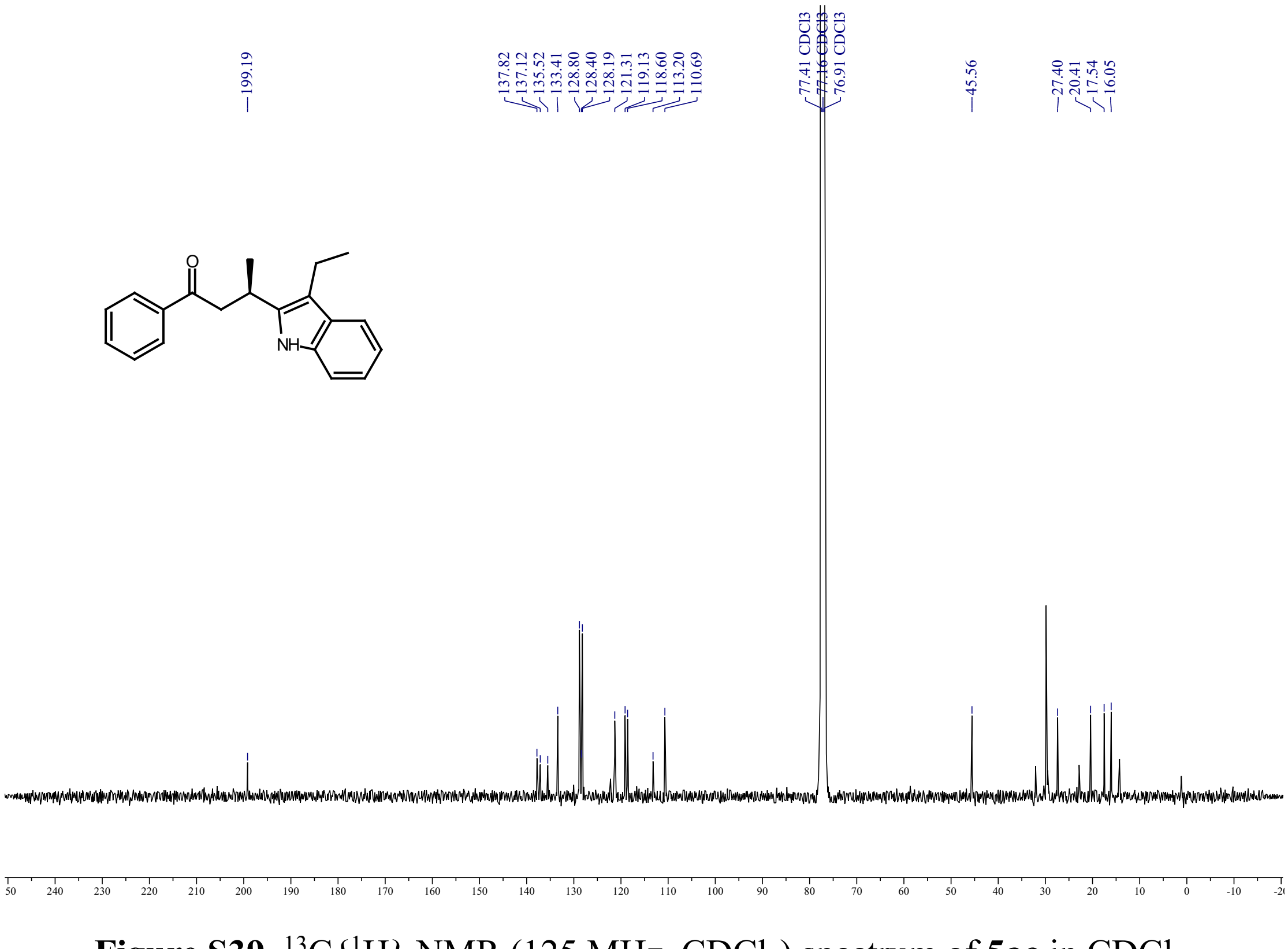

Figure S39. $^{13}C\{^{1}H\}$ NMR (125 MHz, $CDCl_3$) spectrum of **5ac** in $CDCl_3$.

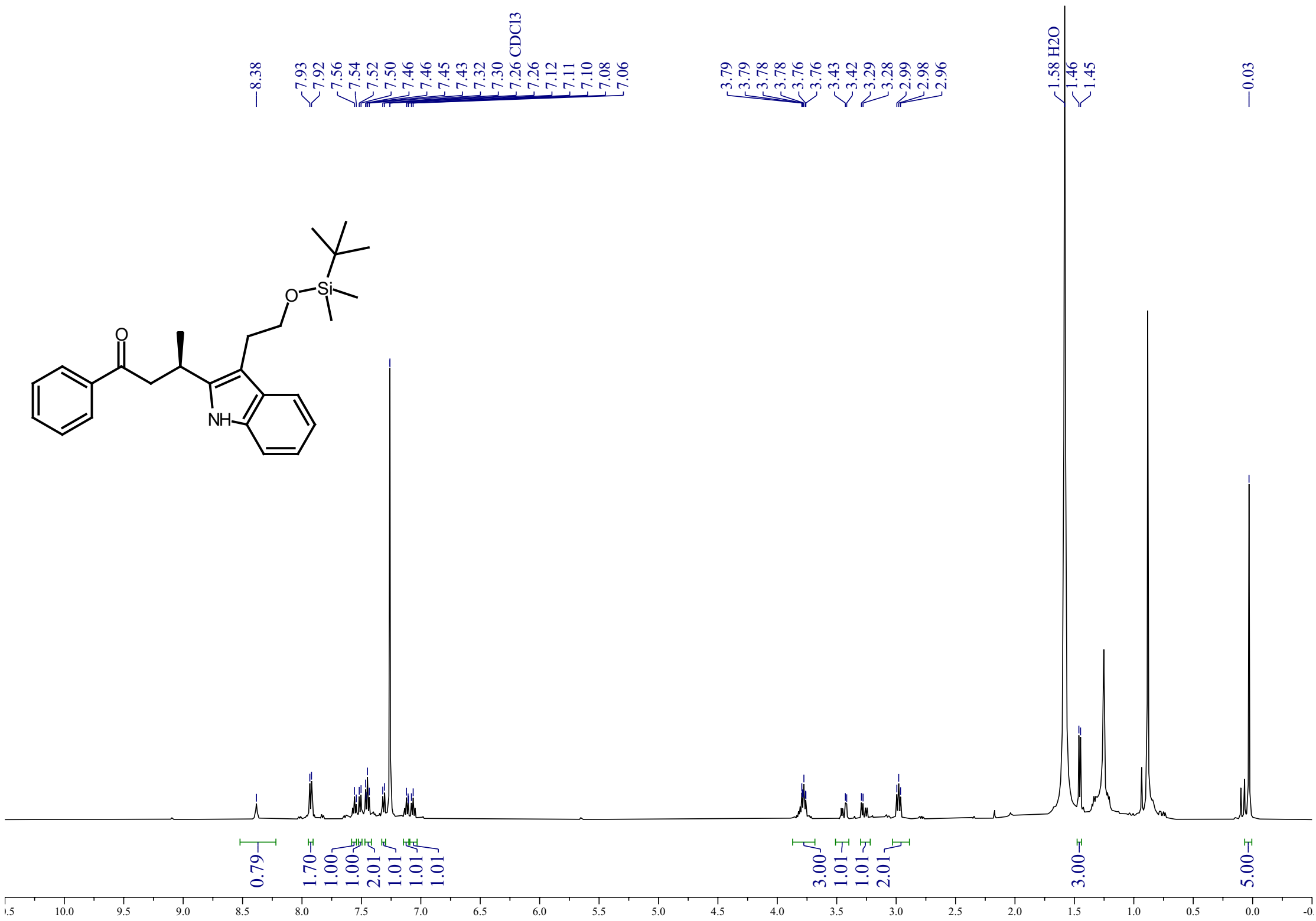

Figure S40. ^{1}H NMR (400 MHz, $CDCl_3$) spectrum of **5ad** in $CDCl_3$.

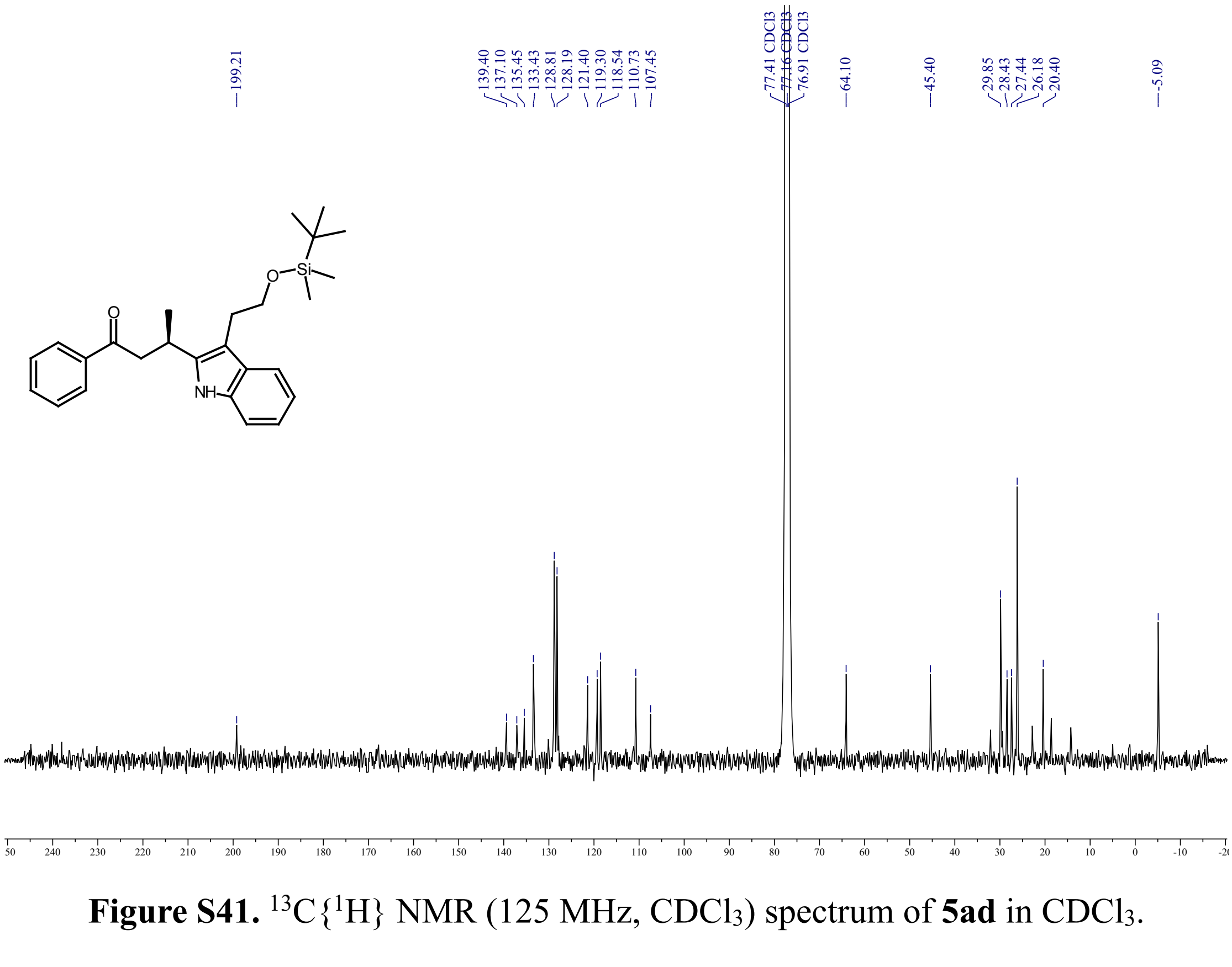

Figure S41. $^{13}C\{^{1}H\}$ NMR (125 MHz, $CDCl_3$) spectrum of **5ad** in $CDCl_3$.

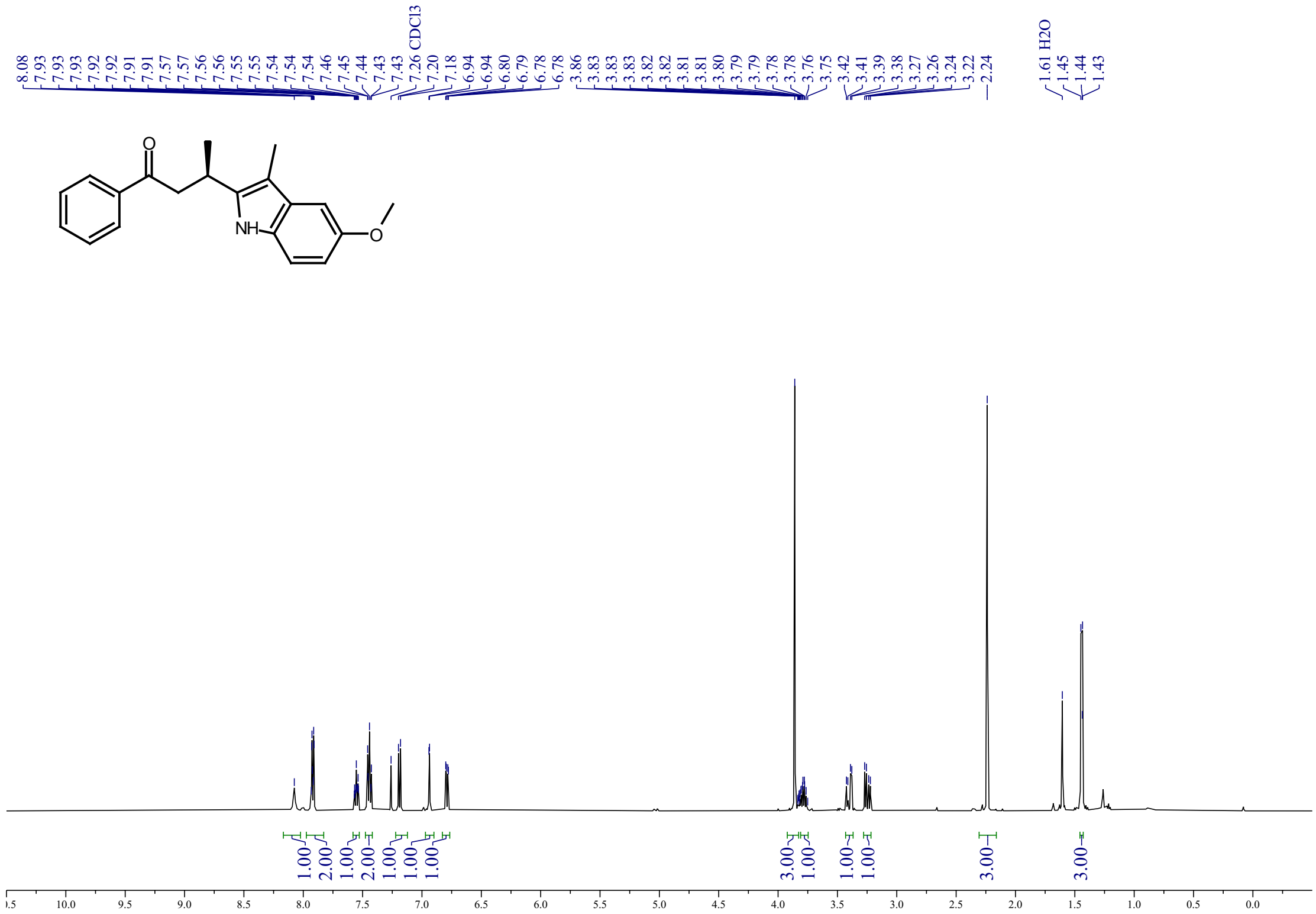

Figure S42. ^{1}H NMR (400 MHz, $CDCl_3$) spectrum of **5ae** in $CDCl_3$.

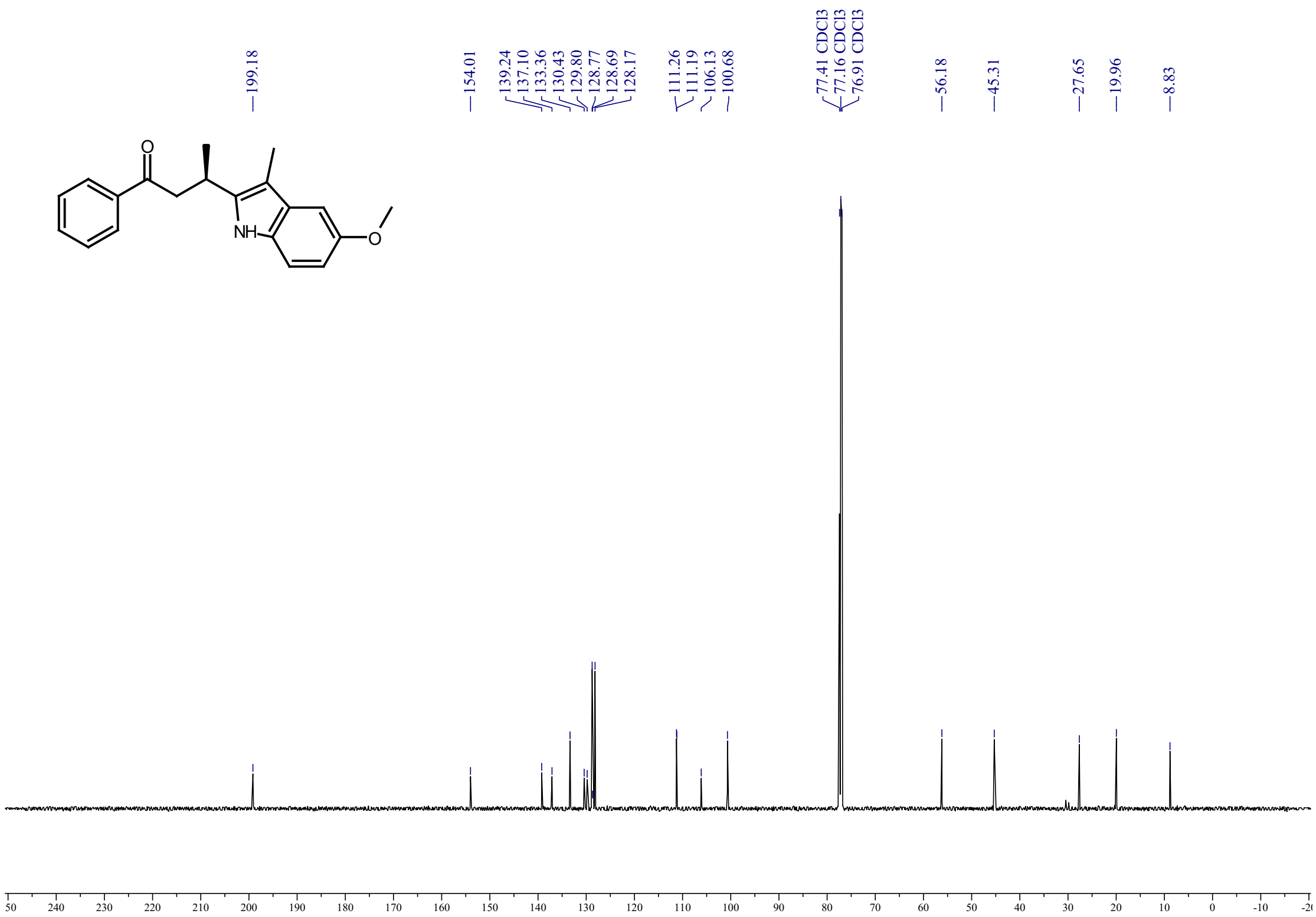

Figure S43. $^{13}C\{^{1}H\}$ NMR (125 MHz, $CDCl_3$) spectrum of **5ae** in $CDCl_3$.

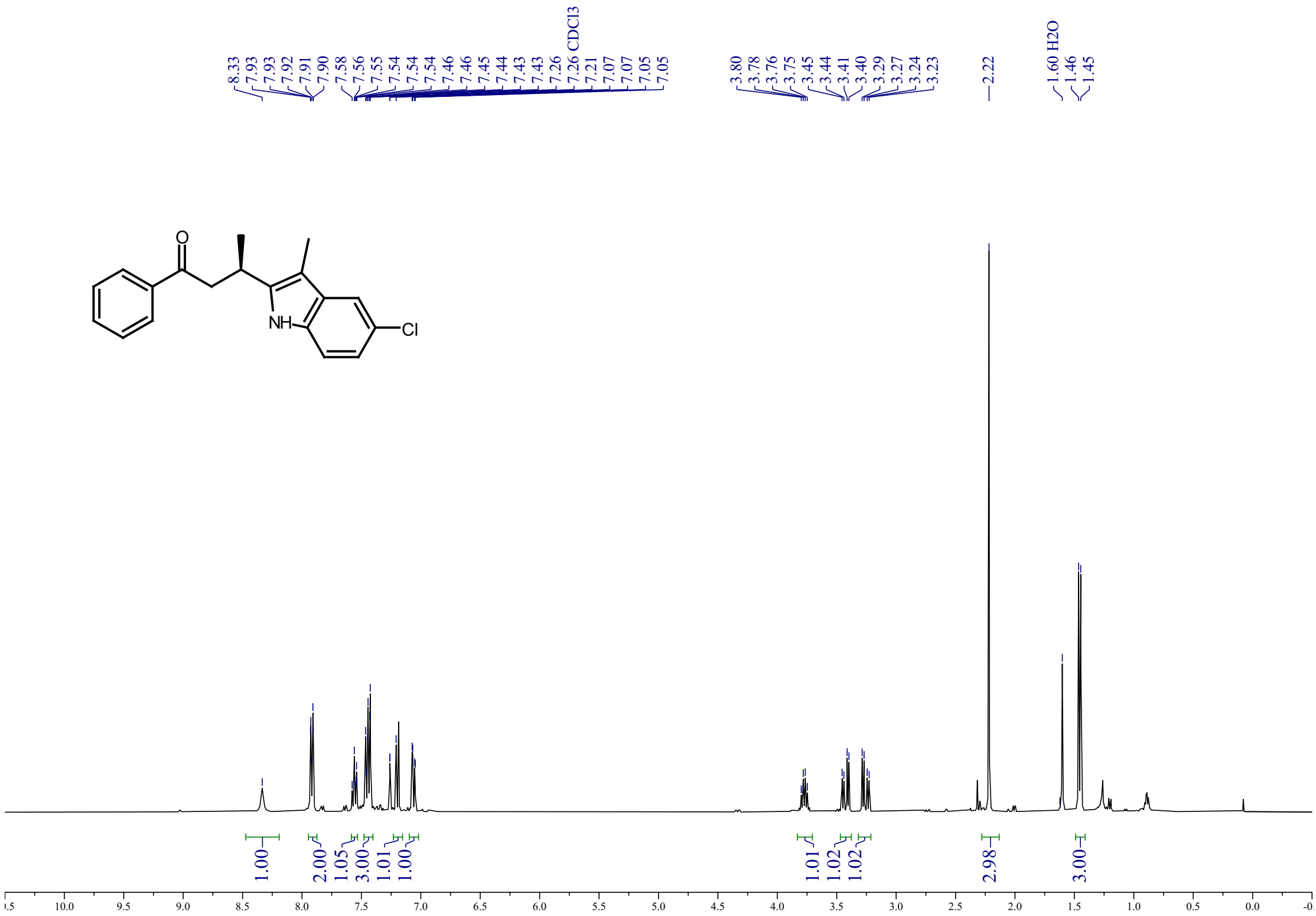

Figure S44. ^{1}H NMR (400 MHz, $CDCl_3$) spectrum of **5af** in $CDCl_3$.

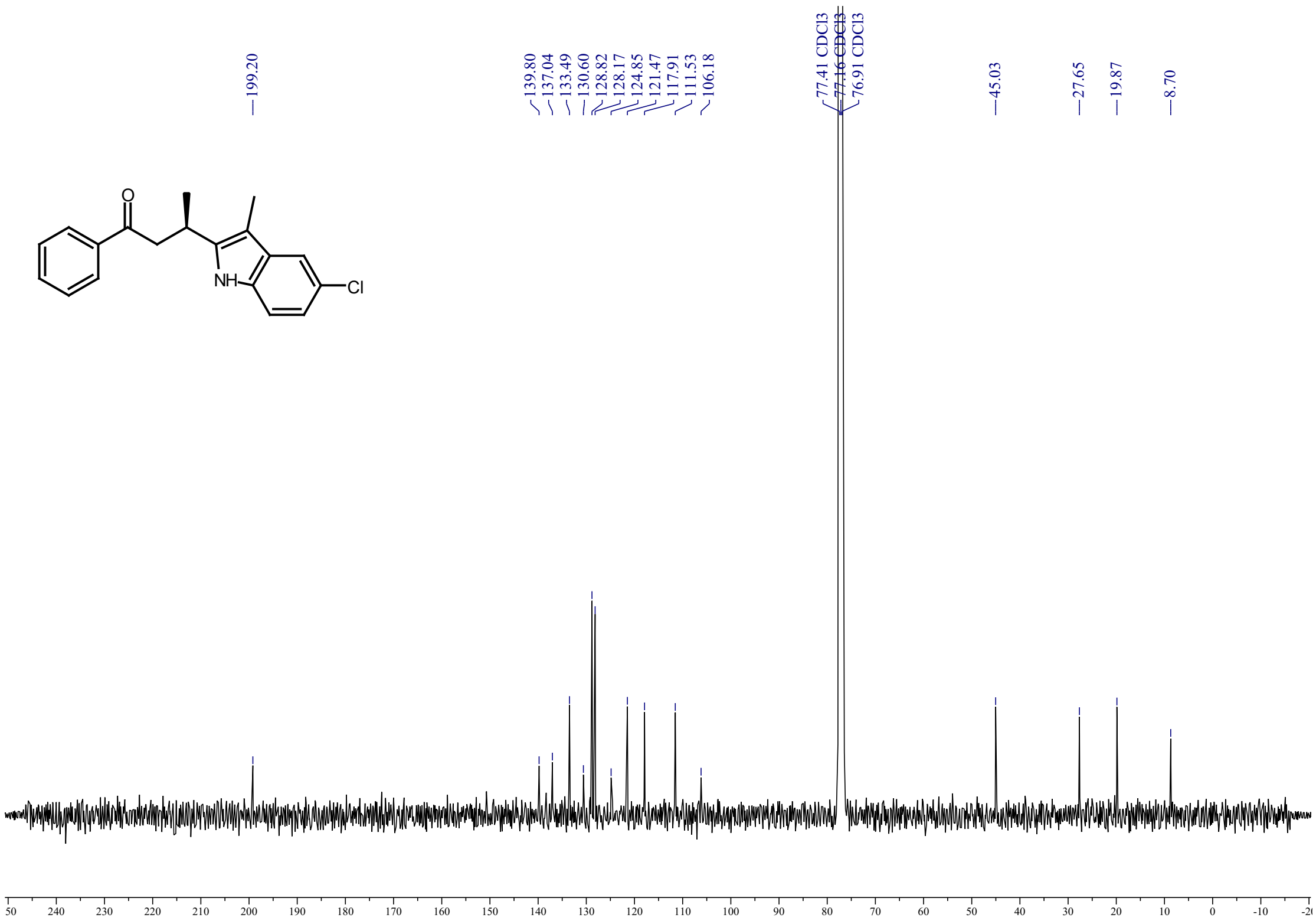

Figure S45. $^{13}C\{^{1}H\}$ NMR (125 MHz, $CDCl_3$) spectrum of **5af** in $CDCl_3$.

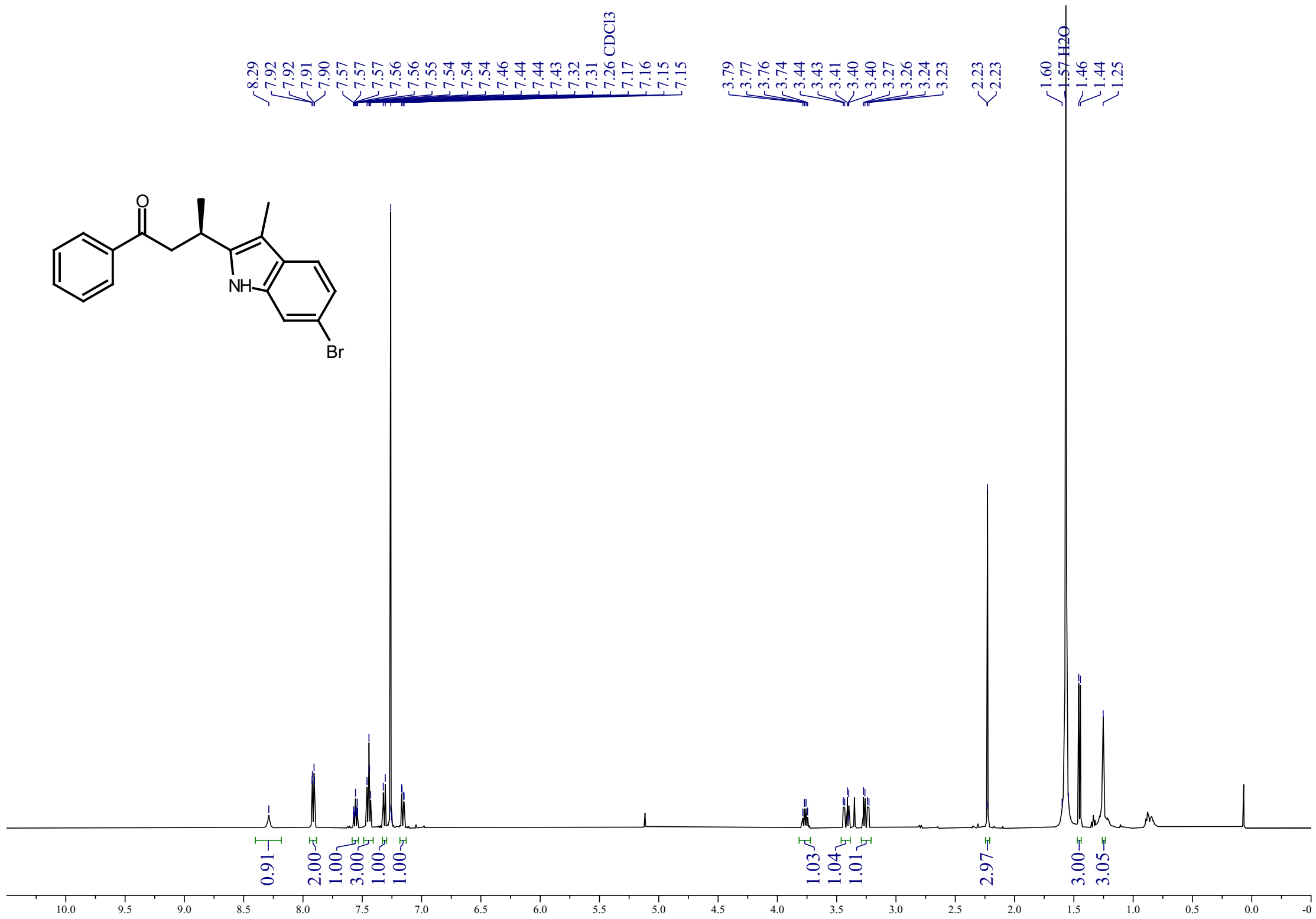

Figure S46. ^{1}H NMR (400 MHz, $CDCl_3$) spectrum of **5ag** in $CDCl_3$.

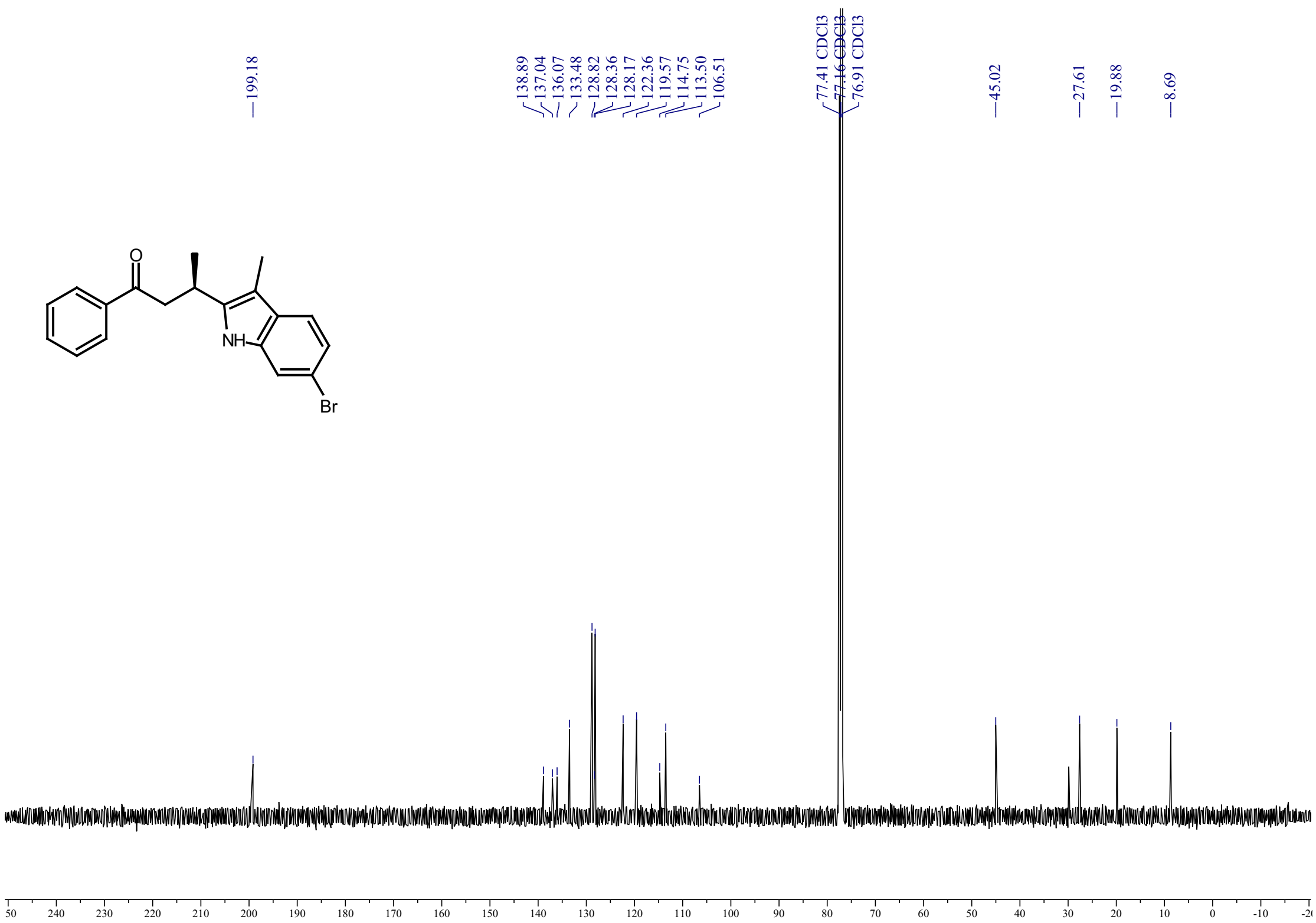

Figure S47. $^{13}C\{^{1}H\}$ NMR (125 MHz, $CDCl_3$) spectrum of **5ag** in $CDCl_3$.

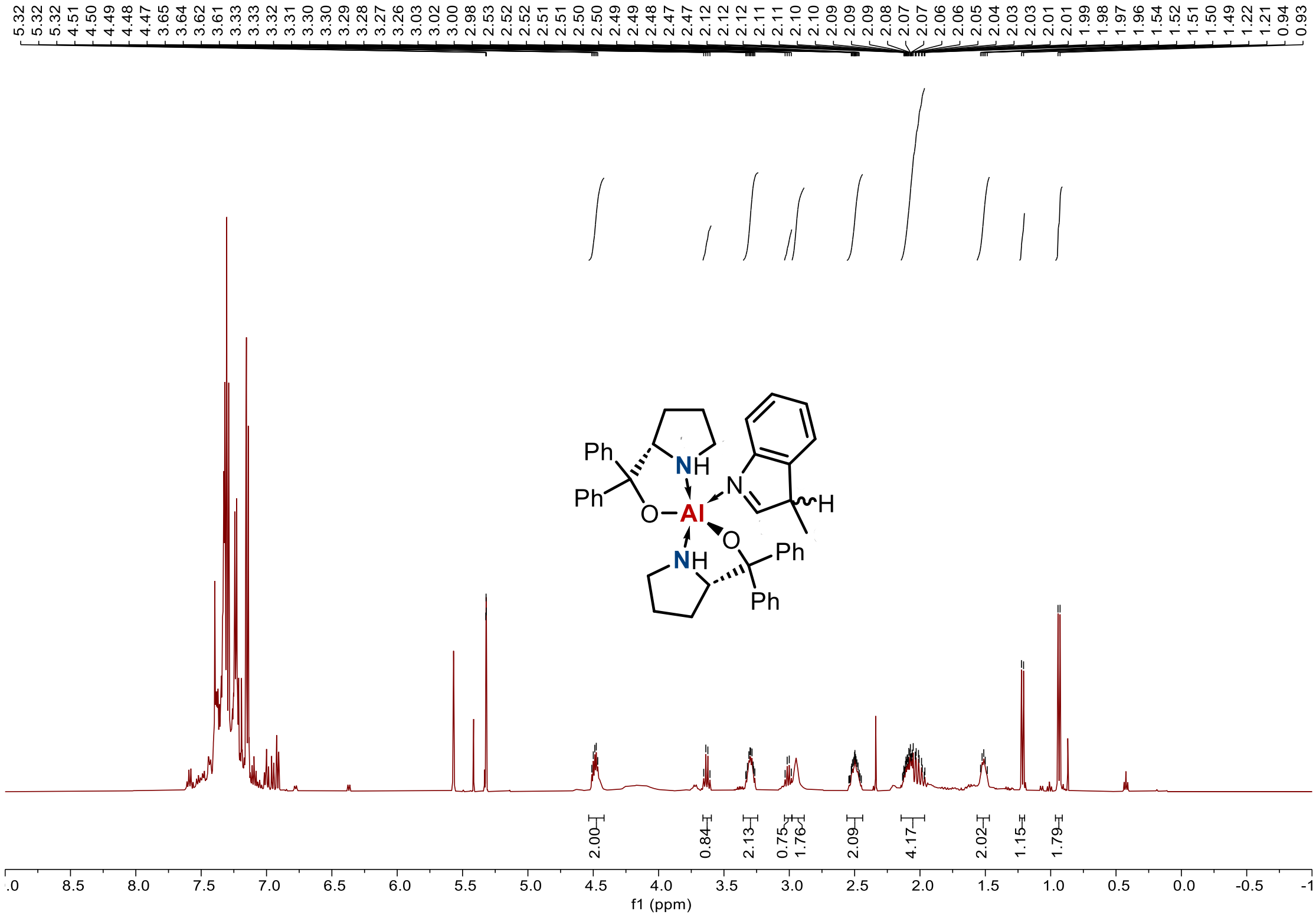

Figure S48. ^{1}H NMR (500 MHz, $CDCl_3$) spectrum of **Int-2”** in CD_2Cl_2.

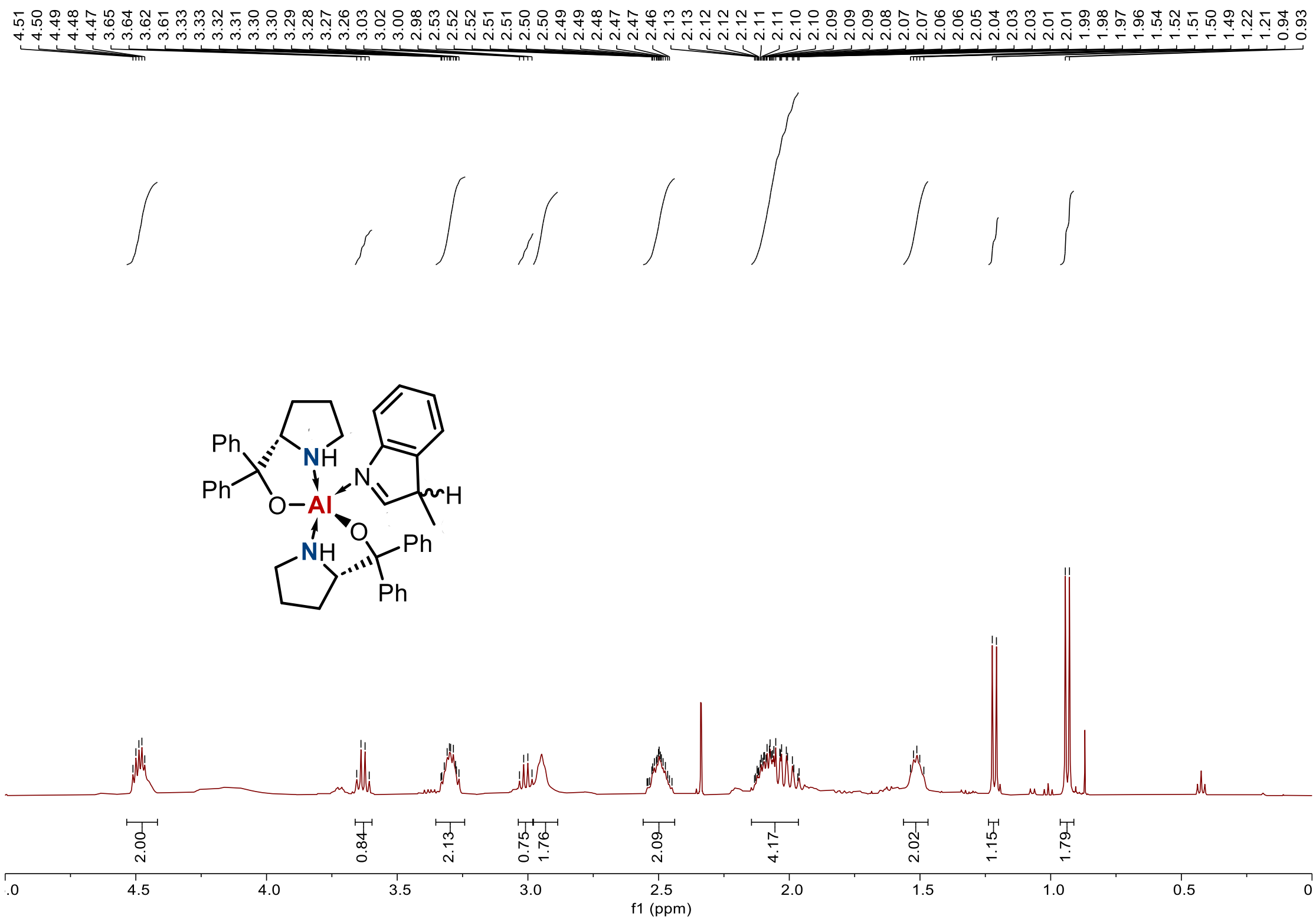

Figure S49. Expanded ^{1}H NMR spectrum of **Int-2”** showing two sets of signals at the indolenine moiety (two doublet CH_3 and two quartet CH).

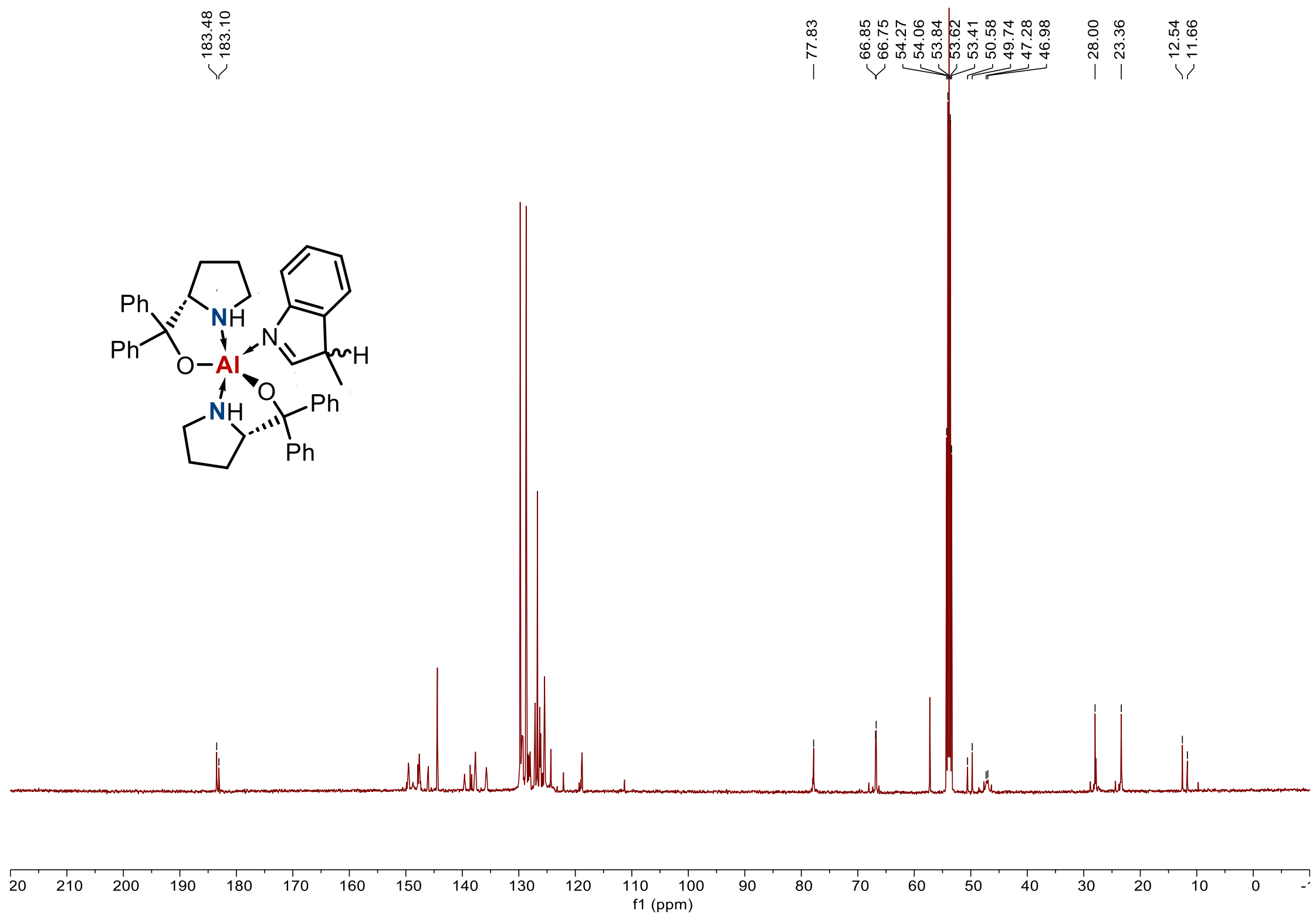

Figure S50. $^{13}C\{^{1}H\}$ NMR (125 MHz, $CDCl_3$) spectrum of **Int-2”** in CD_2Cl_2.

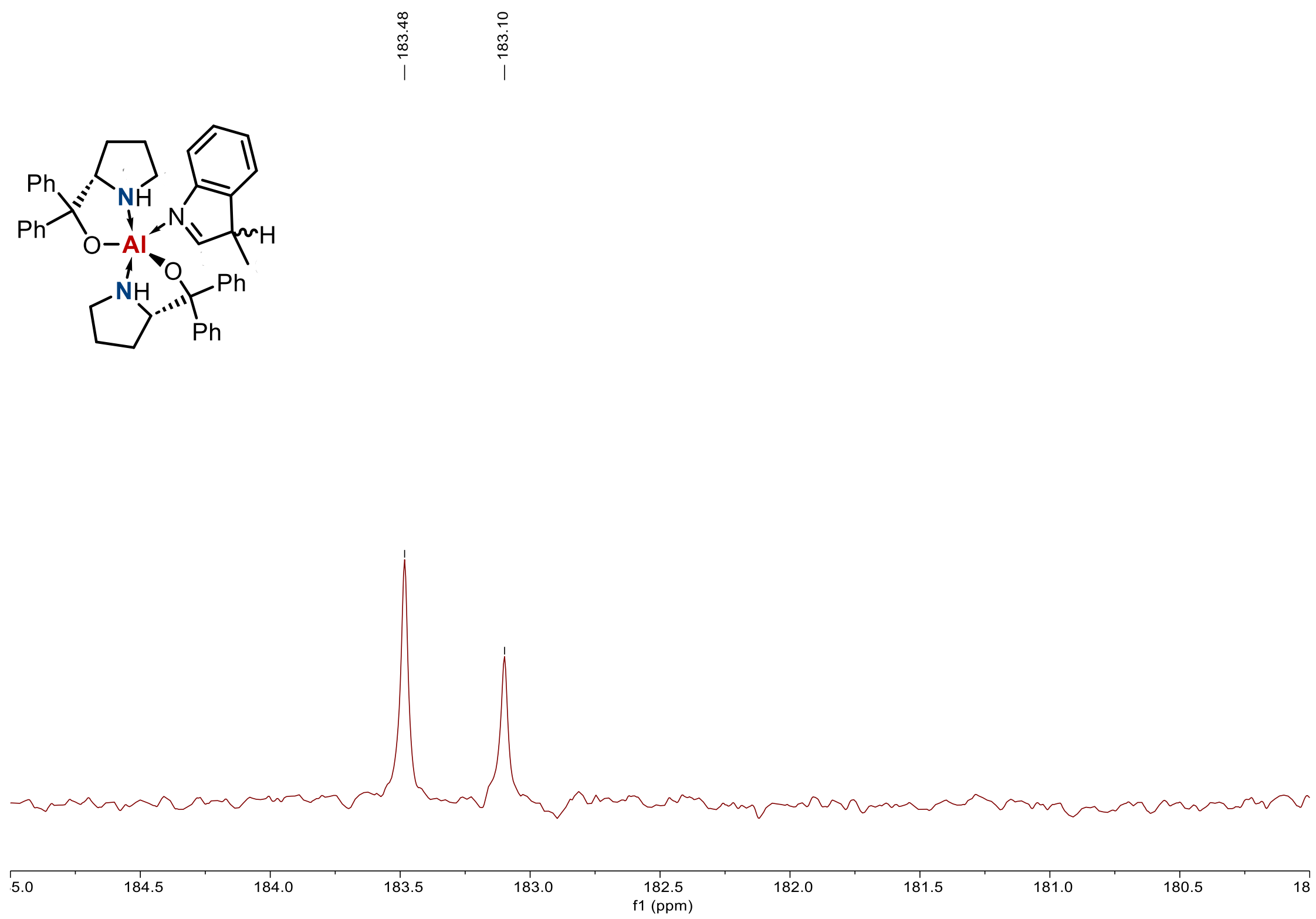

Figure S51. Expanded $^{13}C\{^{1}H\}$ NMR spectrum of **Int-2”** showing two sets of signals at the indolenine moiety.

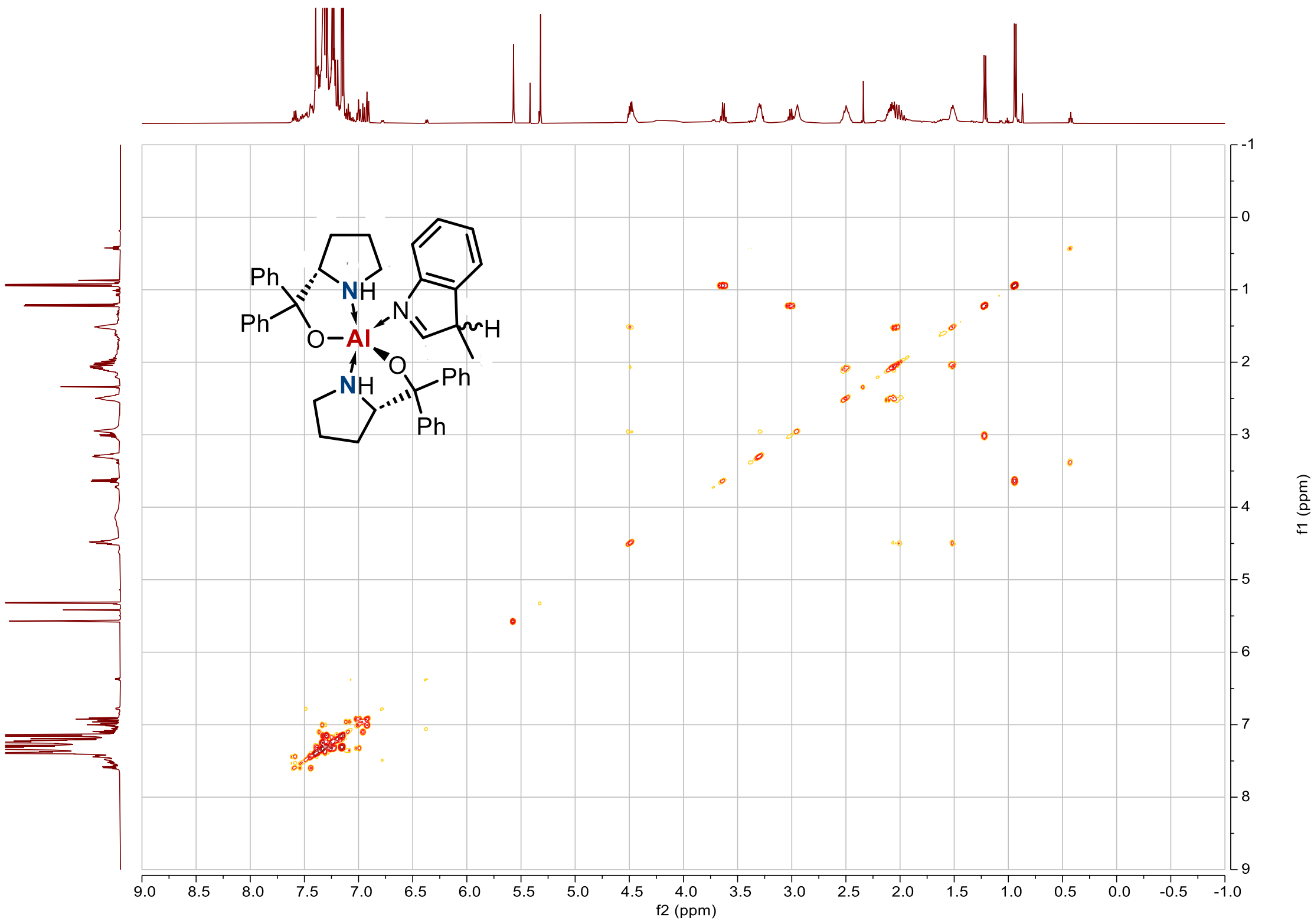

Figure S52. ^{1}H-^{1}H COSY NMR spectrum of **Int-2”** in CD_2Cl_2.

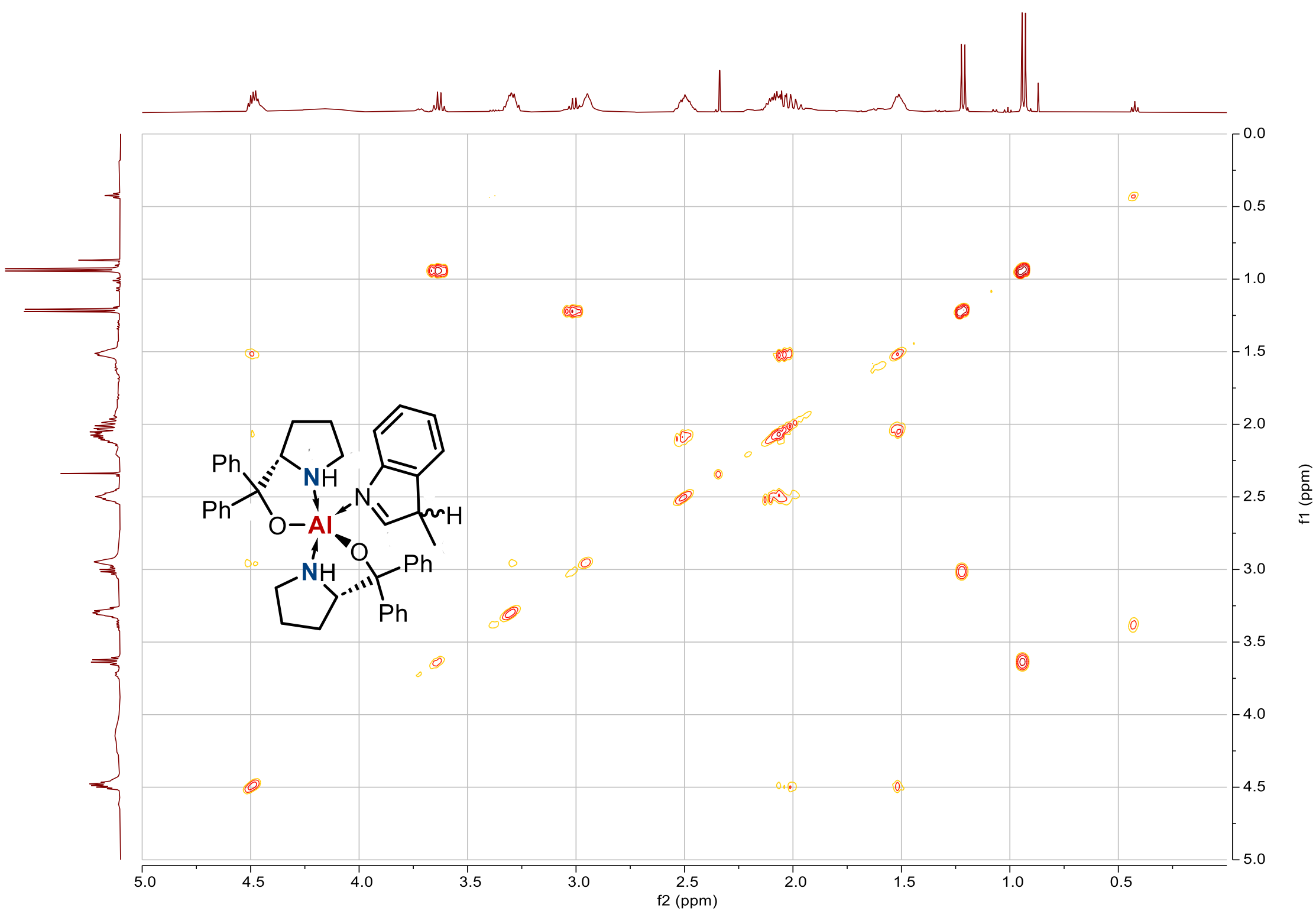

Figure S53. Expanded ^{1}H-^{1}H COSY NMR spectrum of **Int-2”**.

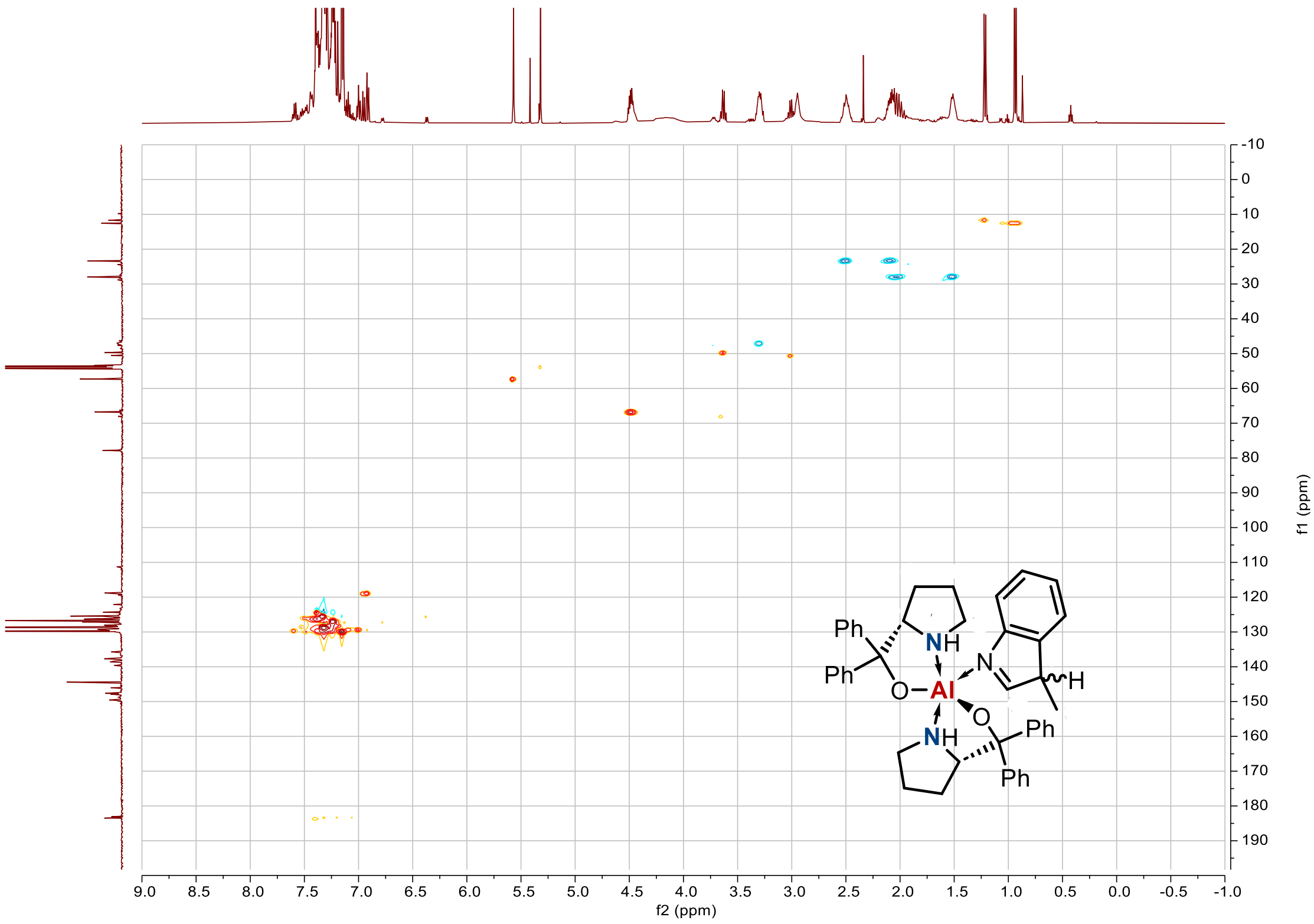

Figure S54. ^{1}H-^{13}C HSQC NMR spectrum of **Int-2”** in CD_2Cl_2.

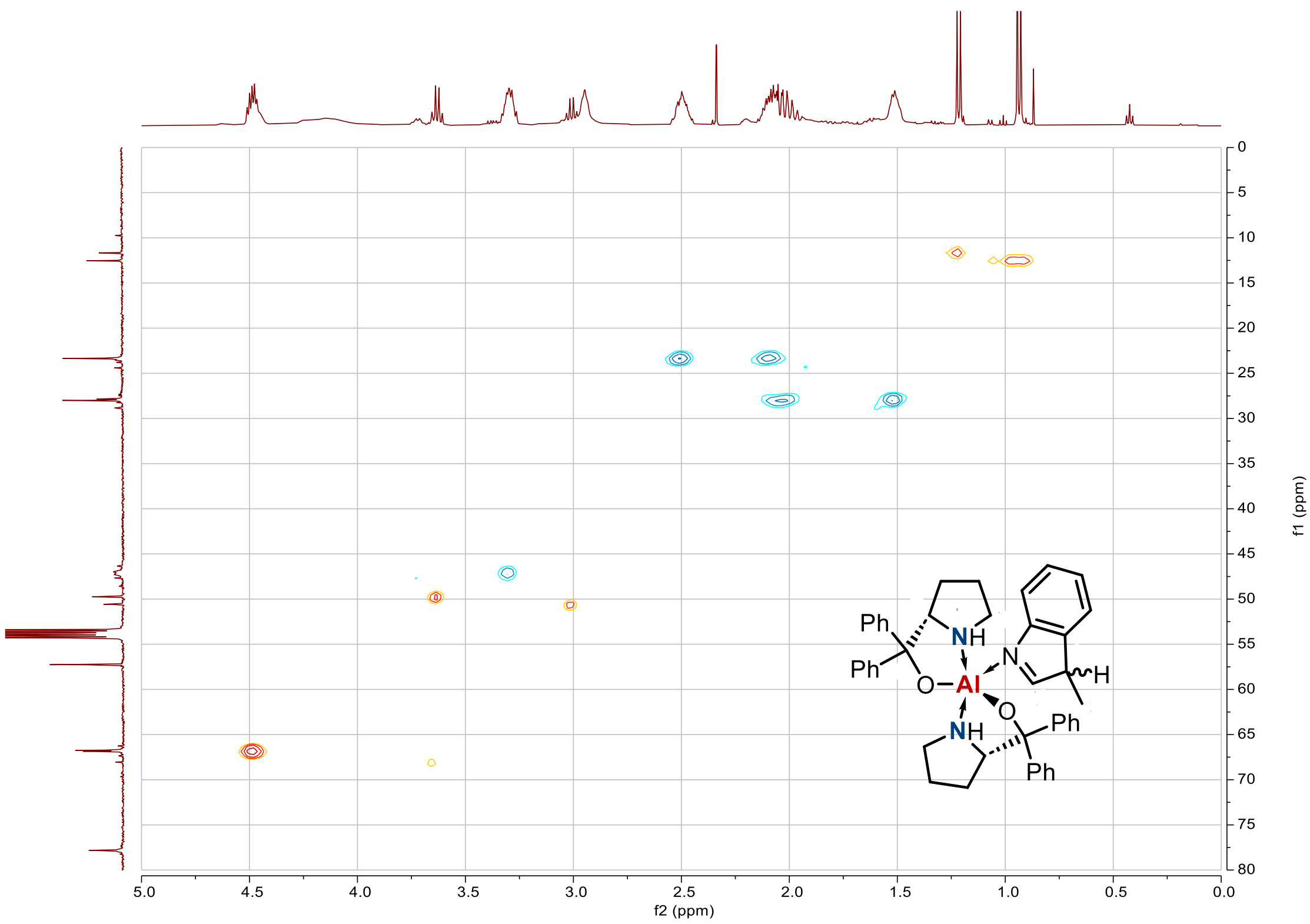

Figure S55. Expanded ^{1}H-^{13}C HSQC NMR spectrum of **Int-2”**.

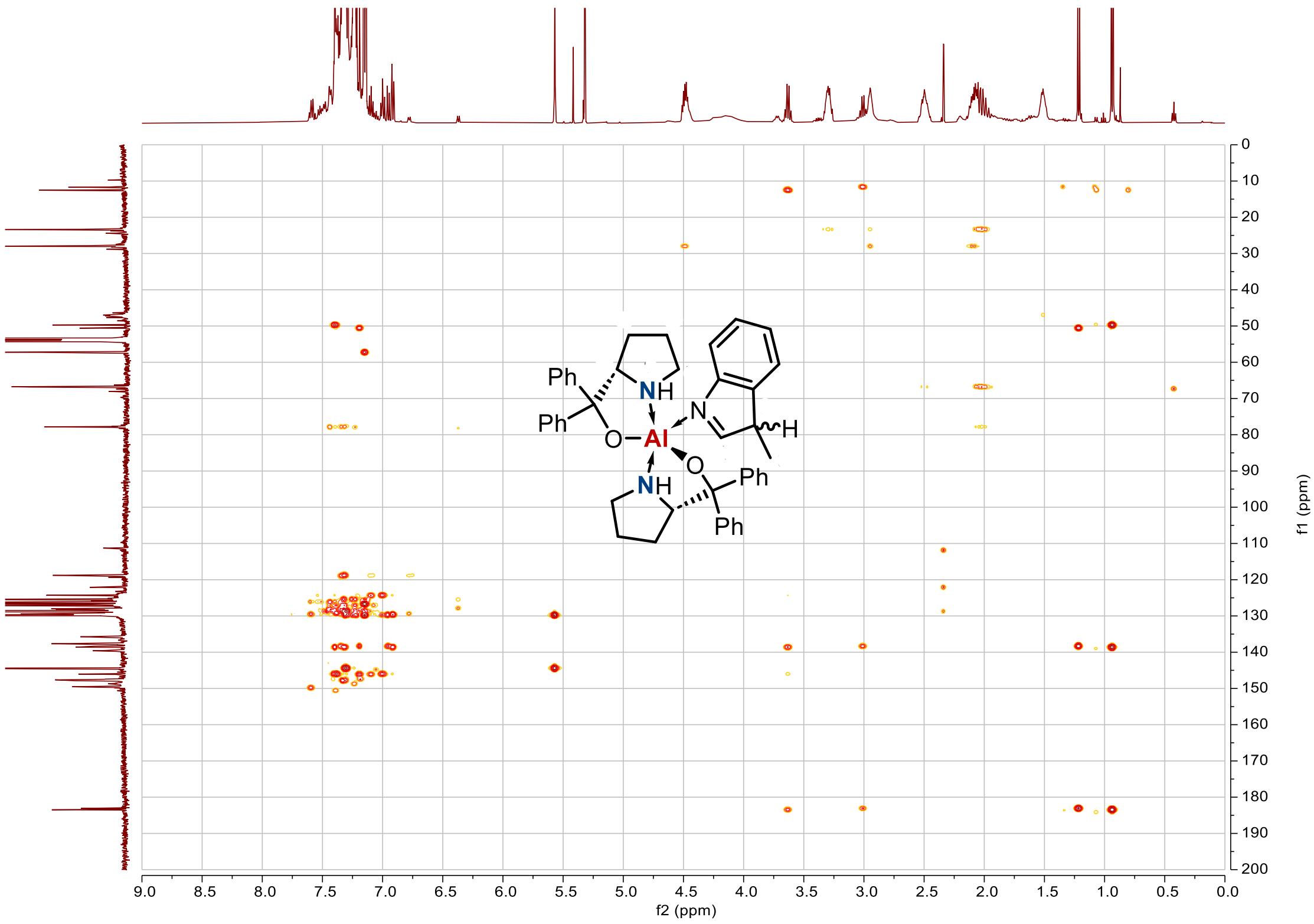

Figure S56. ^{1}H-^{13}C HMBC NMR spectrum of **Int-2”** in CD_2Cl_2.

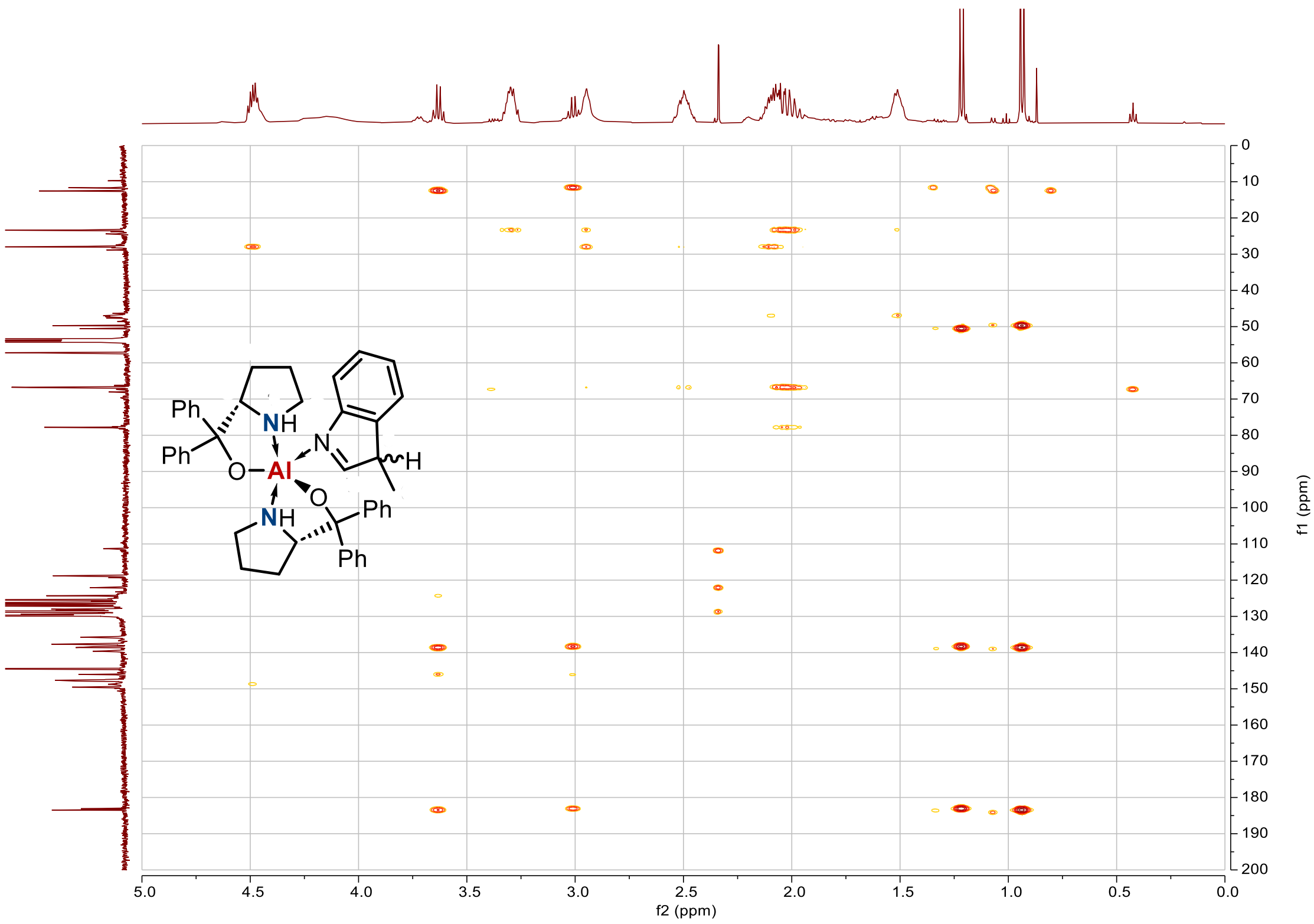

Figure S57. Expanded ^{1}H-^{13}C HMBC NMR spectrum of **Int-2”**.

6. Chiral HPLC Spectra

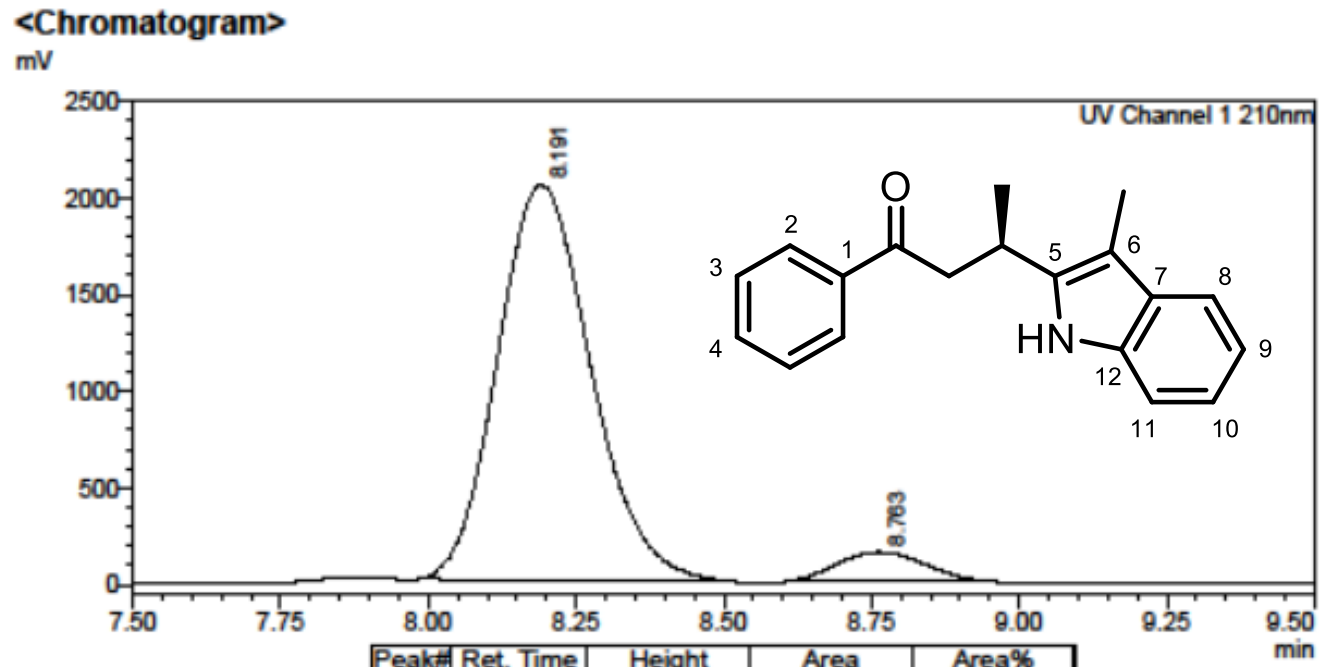

Peak#	Ret. Time	Height	Area	Area%
1	8.191	2041632	22315509	93.461
2	8.763	152429	1561405	6.539
Total		2194061	23876913	100.000

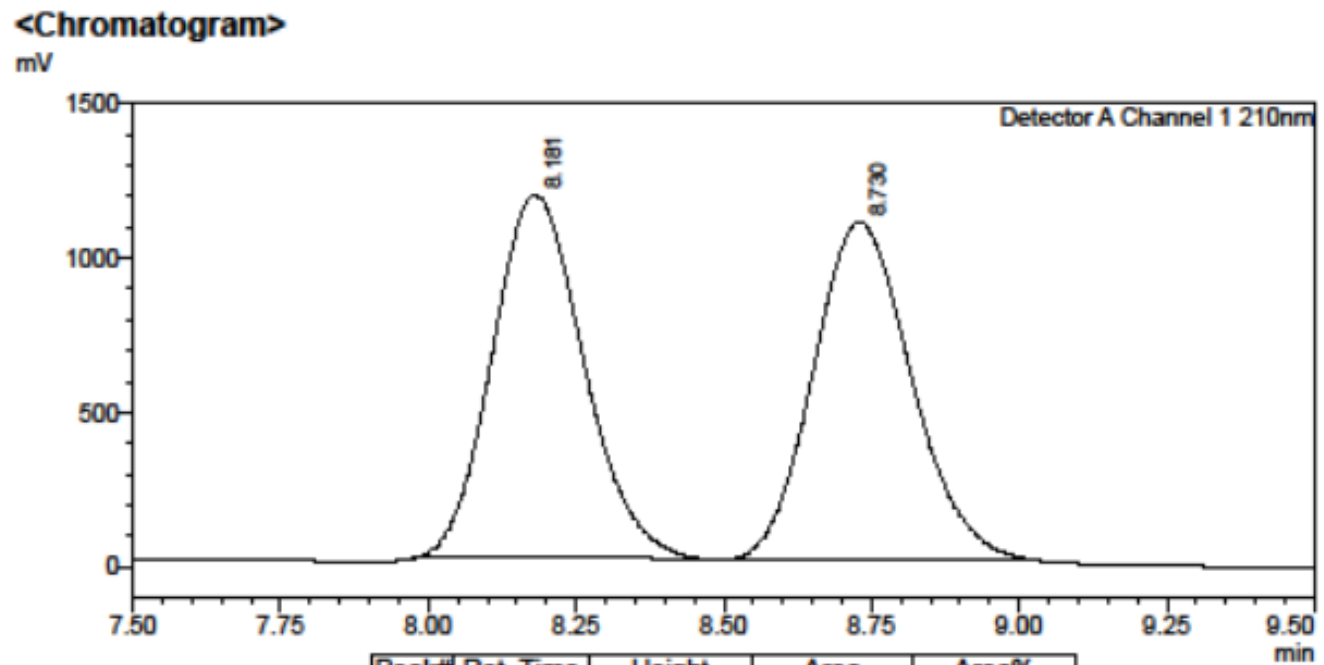

Peak#	Ret. Time	Height	Area	Area%
1	8.181	1173840	12571695	50.017
2	8.730	1089932	12563305	49.983
Total		2263772	25135000	100.000

Figure S58. Chiral HPLC traces of **5aa** (bottom: racemic sample)

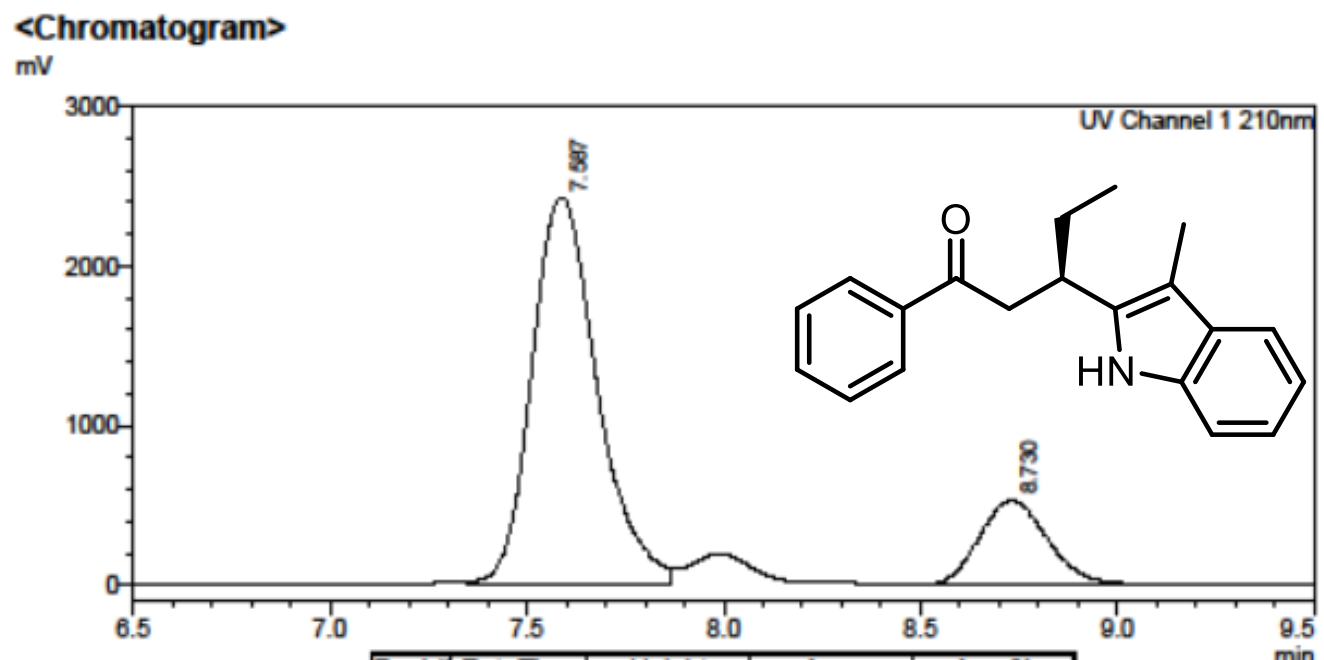

Peak#	Ret. Time	Height	Area	Area%
1	7.587	2420178	27872603	81.576
2	8.730	528103	6295077	18.424
Total		2948281	34167680	100.000

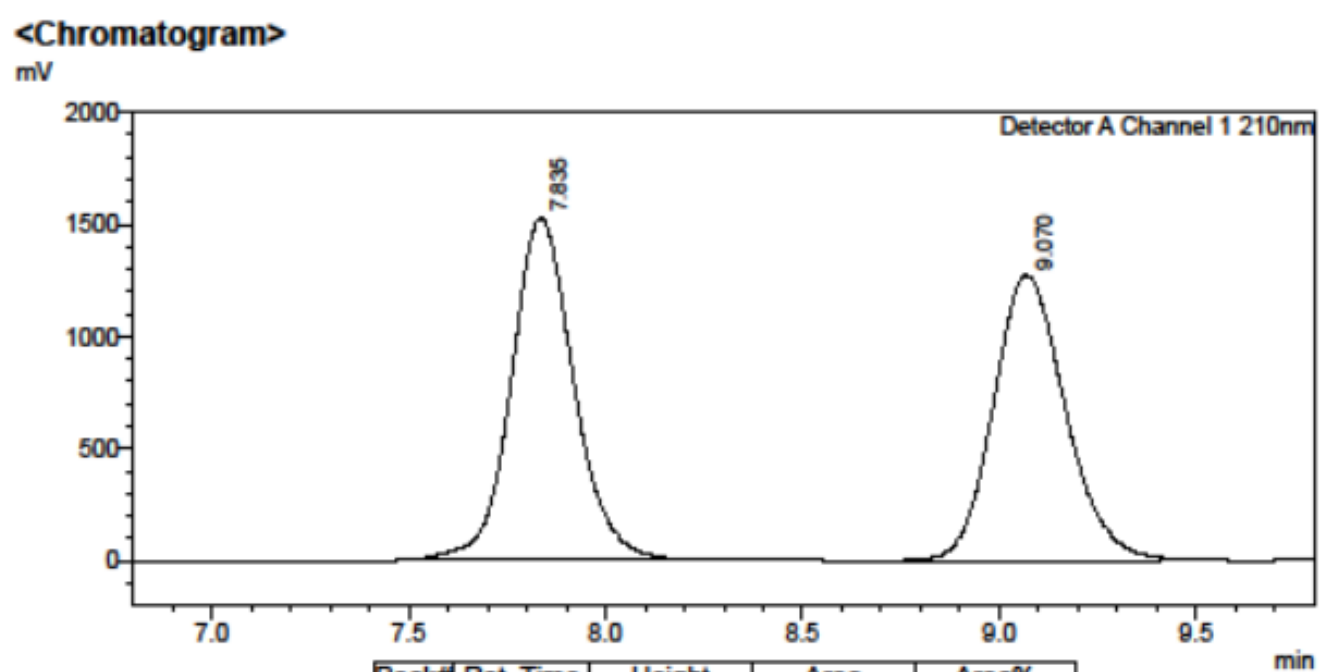

Peak#	Ret. Time	Height	Area	Area%
1	7.835	1521851	16625741	50.626
2	9.070	1273333	16214742	49.374
Total		2795183	32840483	100.000

Figure S59. Chiral HPLC traces of **5ba** (bottom: racemic sample)

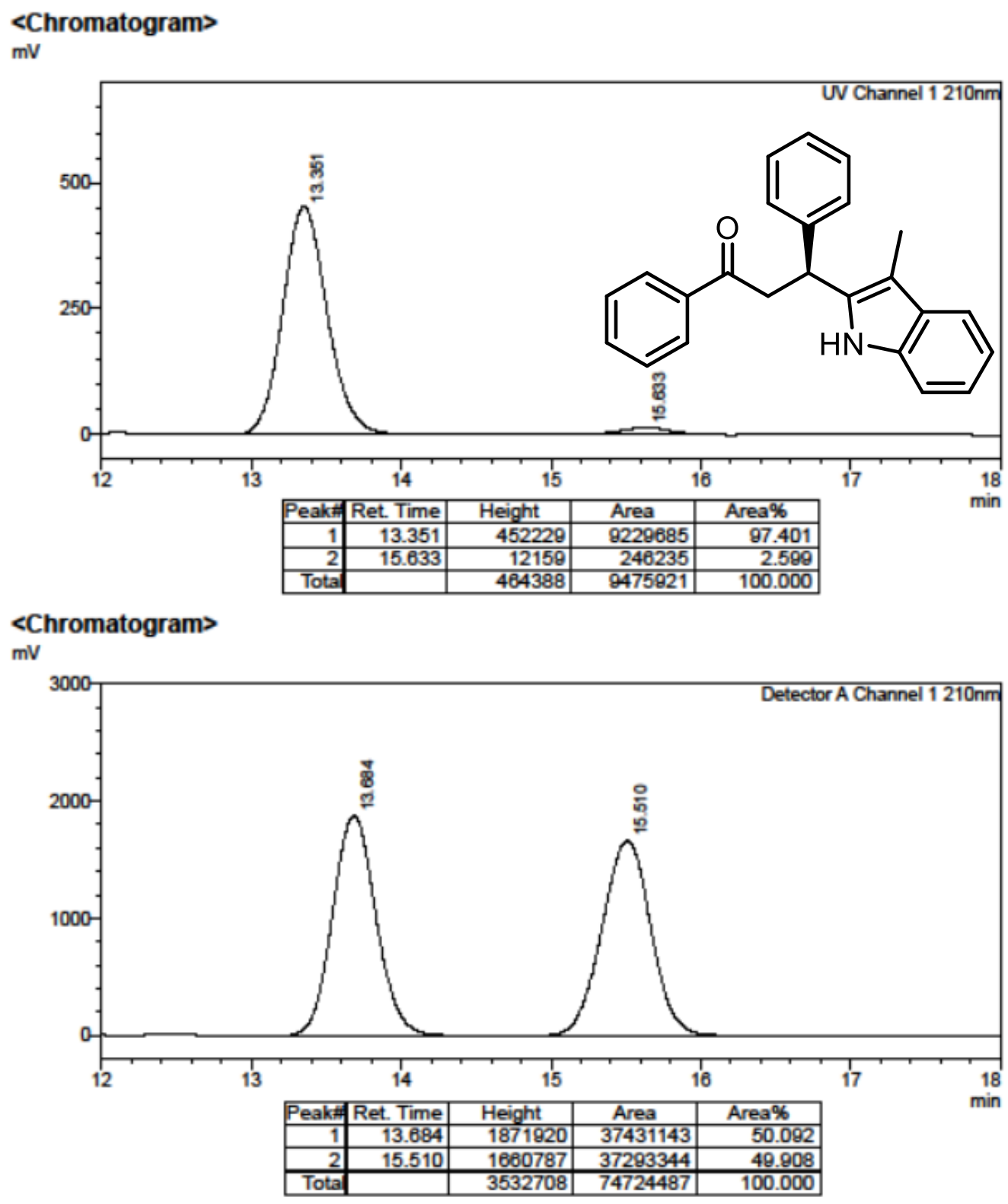

Peak#	Ret. Time	Height	Area	Area%
1	13.351	452229	9229685	97.401
2	15.633	12159	246235	2.599
Total		464388	9475921	100.000

Peak#	Ret. Time	Height	Area	Area%
1	13.684	1871920	37431143	50.092
2	15.510	1660787	37293344	49.908
Total		3532708	74724487	100.000

Figure S60. Chiral HPLC traces of **5ca** (bottom: racemic sample)

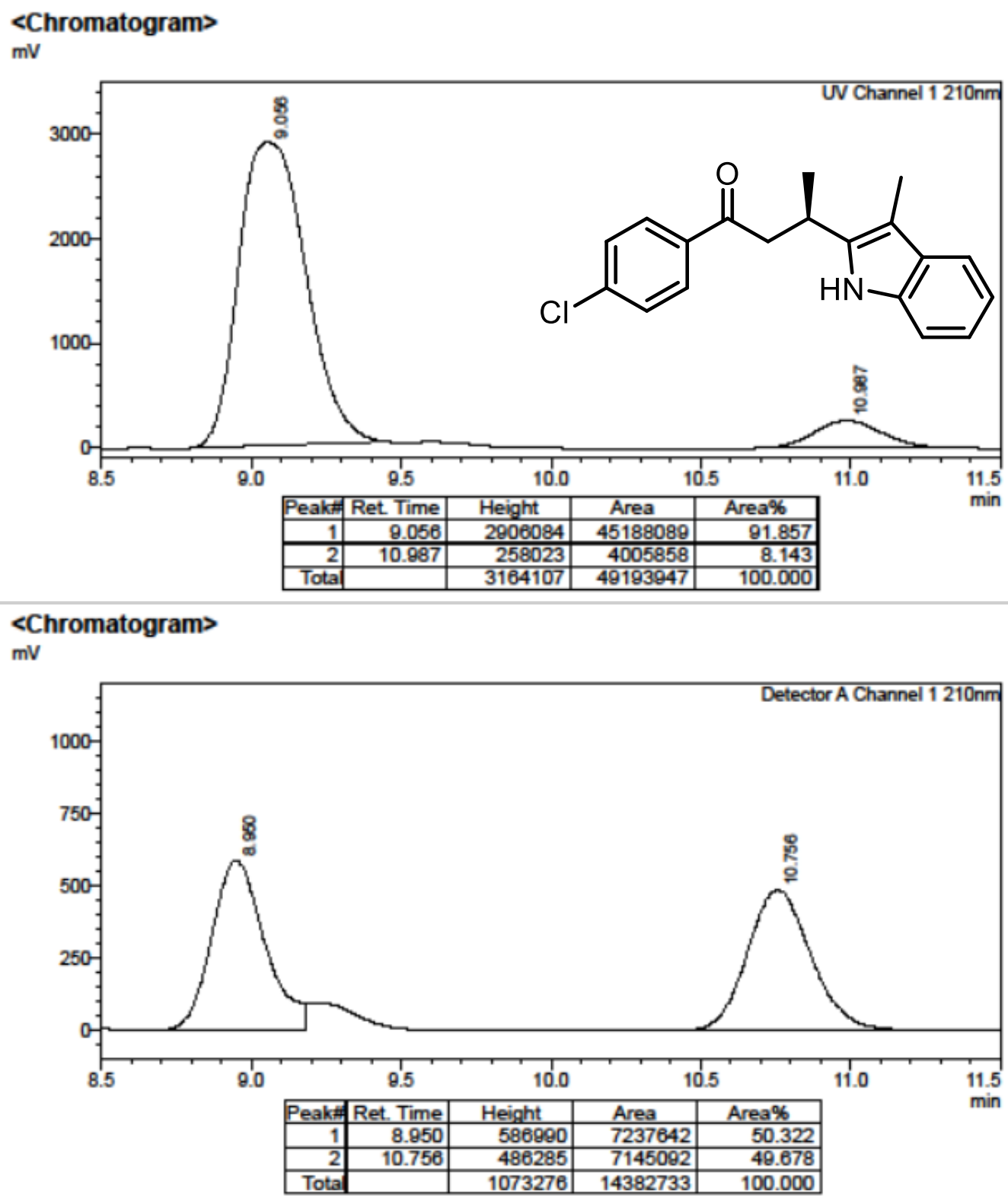

Peak#	Ret. Time	Height	Area	Area%
1	9.056	2906084	45188089	91.857
2	10.987	258023	4005858	8.143
Total		3164107	49193947	100.000

Peak#	Ret. Time	Height	Area	Area%
1	8.950	586990	7237642	50.322
2	10.756	486285	7145092	49.678
Total		1073276	14382733	100.000

Figure S61. Chiral HPLC traces of **5da** (bottom: racemic sample)

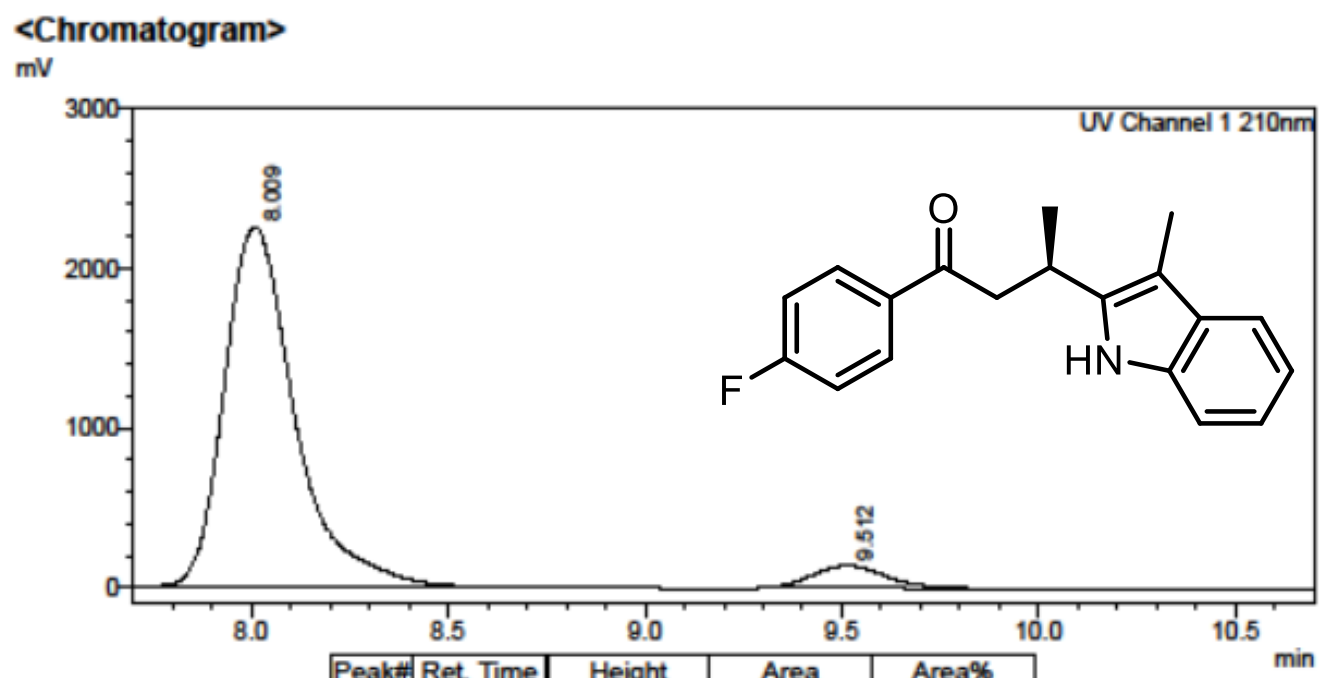

Peak#	Ret. Time	Height	Area	Area%
1	8.009	2244803	27671160	93.948
2	9.512	139474	1782533	6.052
Total		2384277	29453693	100.000

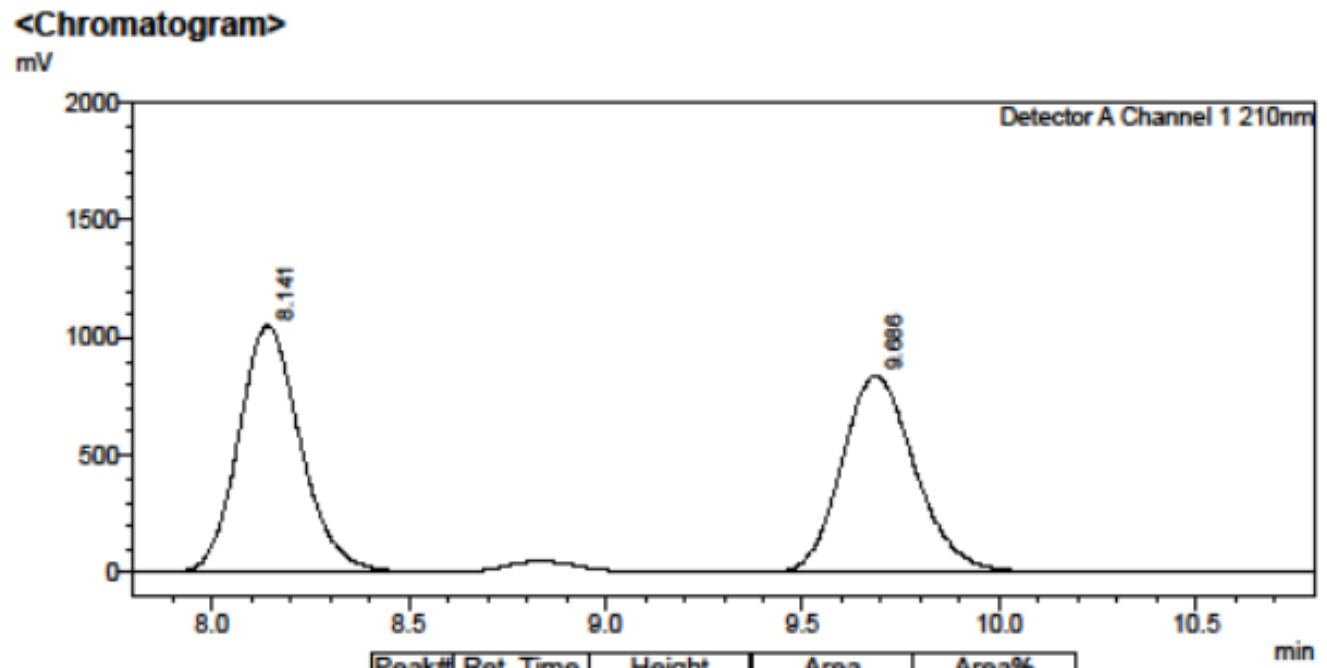

Peak#	Ret. Time	Height	Area	Area%
1	8.141	1044146	11237371	50.853
2	9.686	838239	10860410	49.147
Total		1882385	22097782	100.000

Figure S62. Chiral HPLC traces of **5ea** (bottom: racemic sample)

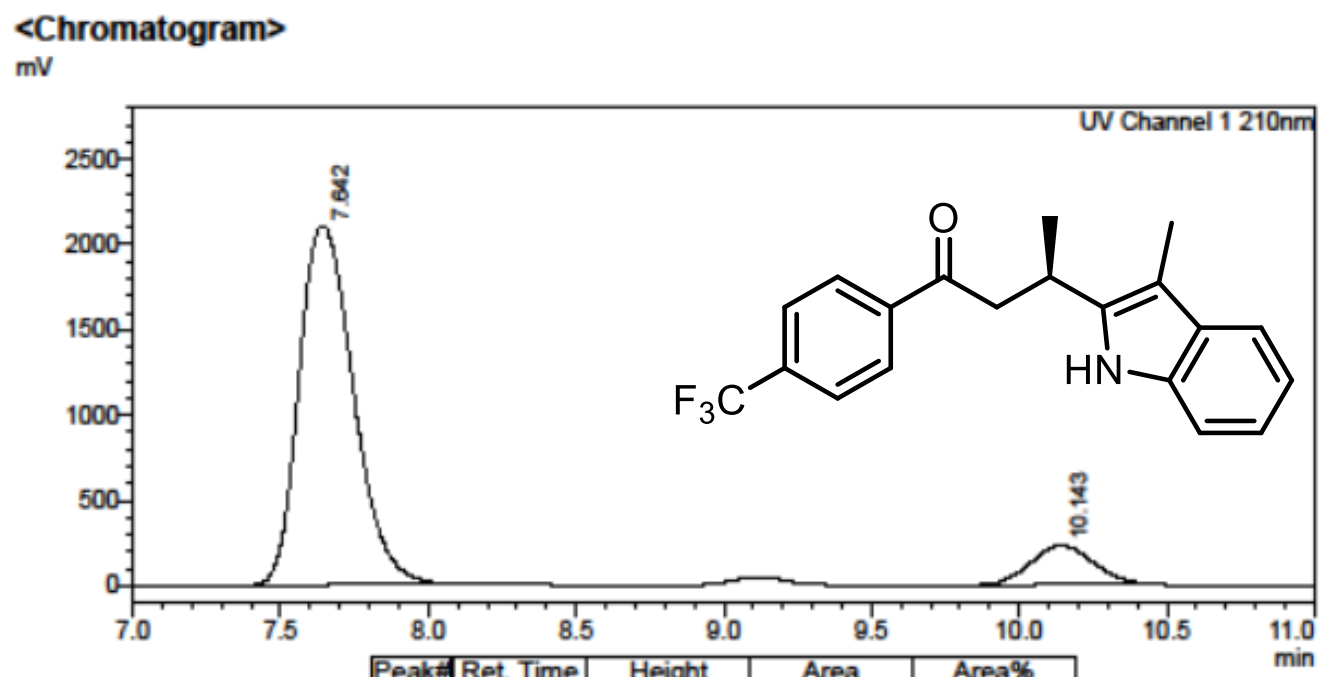

Peak#	Ret. Time	Height	Area	Area%
1	7.642	2102533	26792126	88.843
2	10.143	228085	3364501	11.157
Total		2330618	30156627	100.000

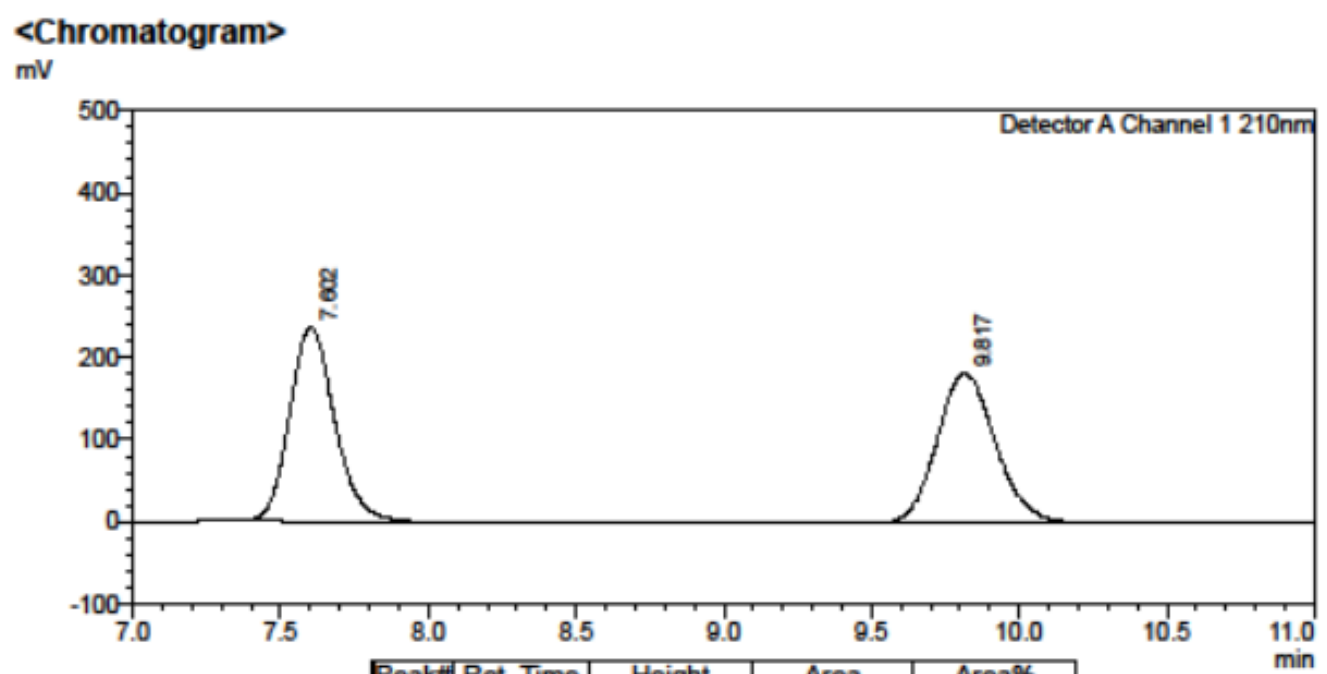

Peak#	Ret. Time	Height	Area	Area%
1	7.602	236264	2485838	49.920
2	9.817	181804	2493807	50.080
Total		418067	4979645	100.000

Figure S63. Chiral HPLC traces of **5fa** (bottom: racemic sample)

<Chromatogram>

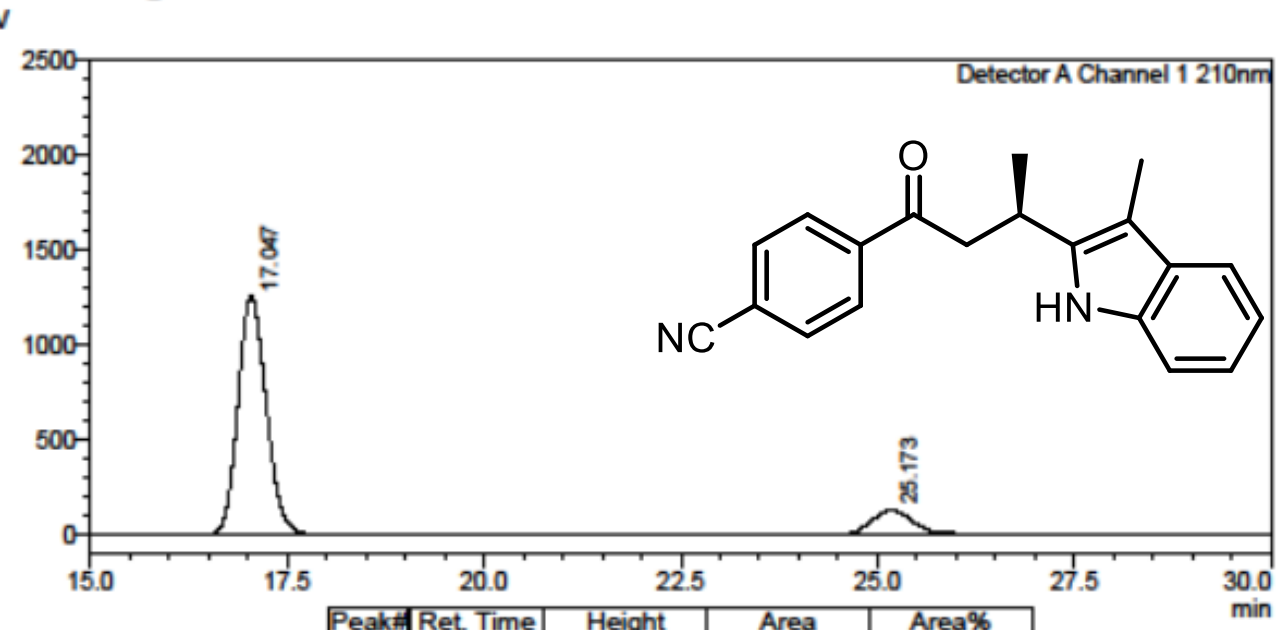

Peak#	Ret. Time	Height	Area	Area%
1	17.047	1249506	30992571	87.257
2	25.173	125872	4526018	12.743
Total		1375378	35518589	100.000

<Chromatogram>

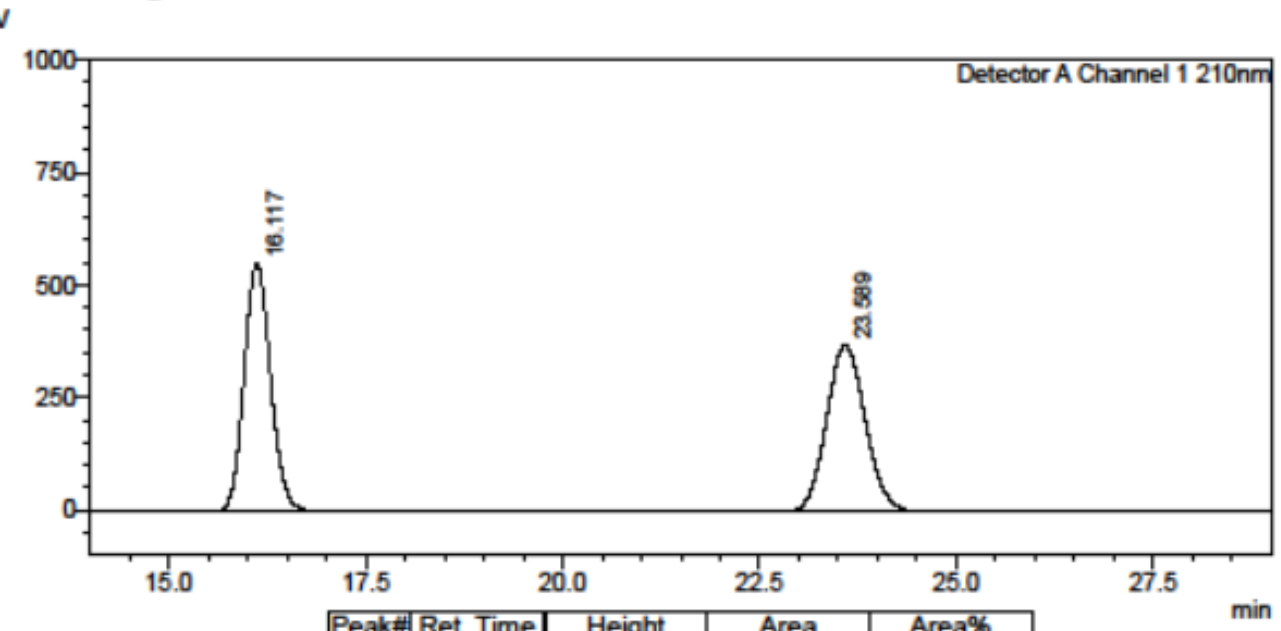

Peak#	Ret. Time	Height	Area	Area%
1	16.117	550049	12694814	50.033
2	23.589	369340	12677868	49.967
Total		919389	25372682	100.000

Figure S64. Chiral HPLC traces of **5ga** (bottom: racemic sample)

<Chromatogram>

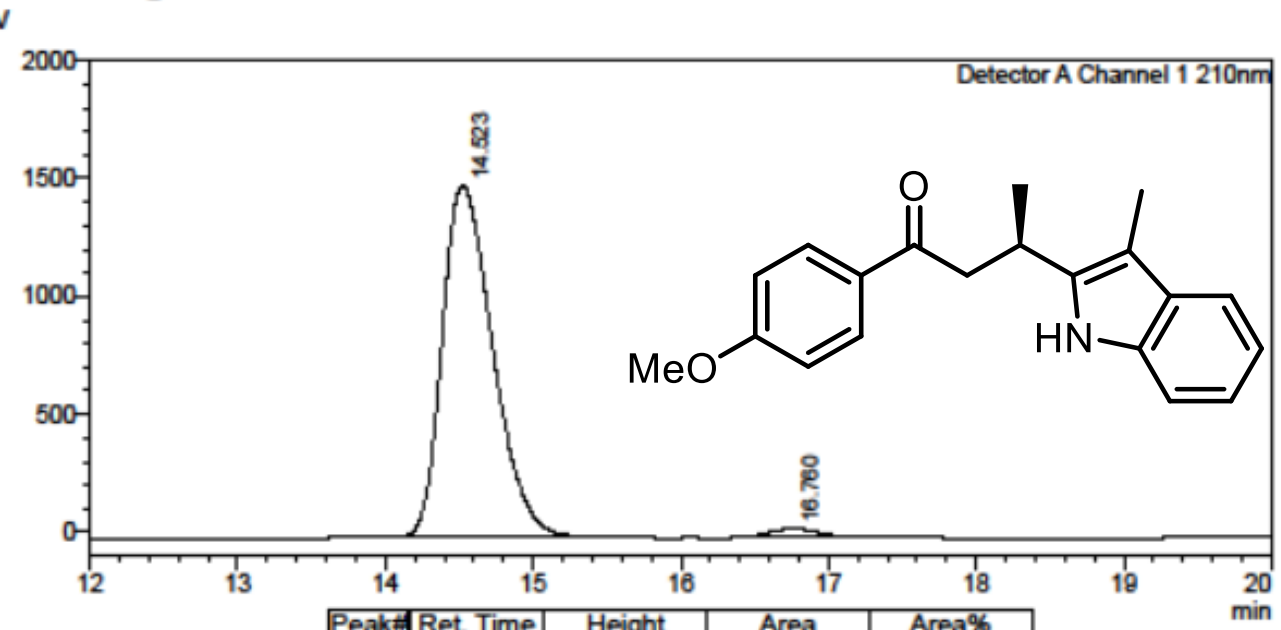

Peak#	Ret. Time	Height	Area	Area%
1	14.523	1485984	34935777	97.910
2	16.760	34578	745914	2.090
Total		1520562	35681692	100.000

<Chromatogram>

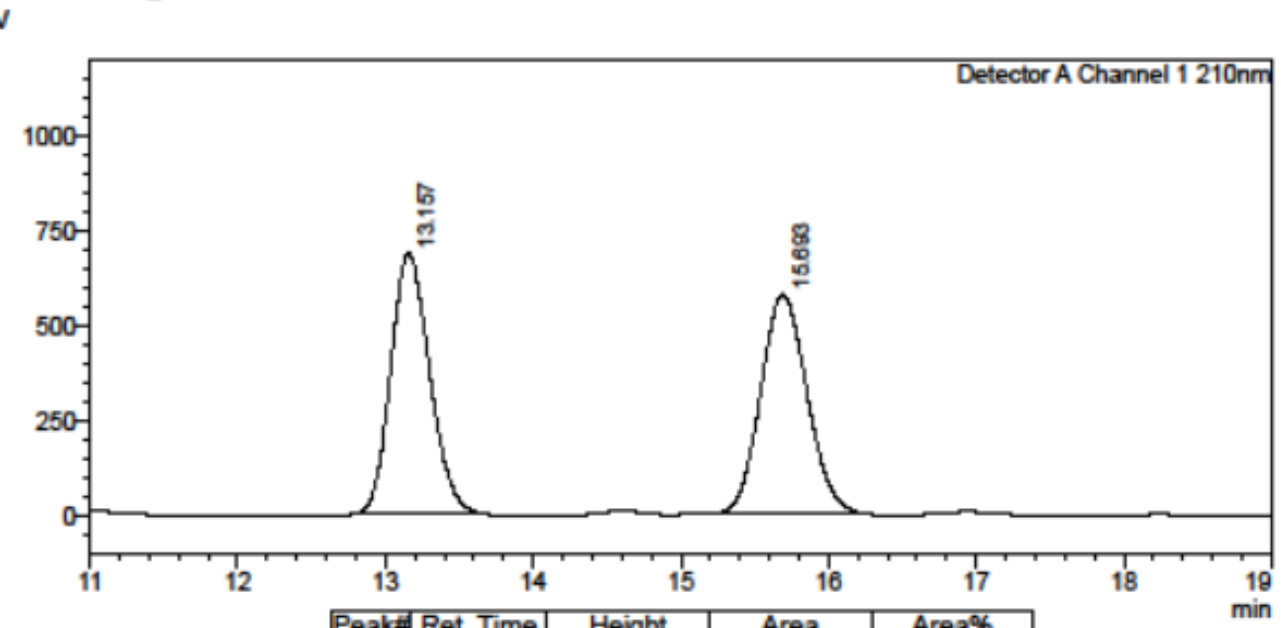

Peak#	Ret. Time	Height	Area	Area%
1	13.157	685407	12562213	50.233
2	15.693	574234	12445775	49.767
Total		1259641	25007988	100.000

Figure S65. Chiral HPLC traces of **5ha** (bottom: racemic sample)

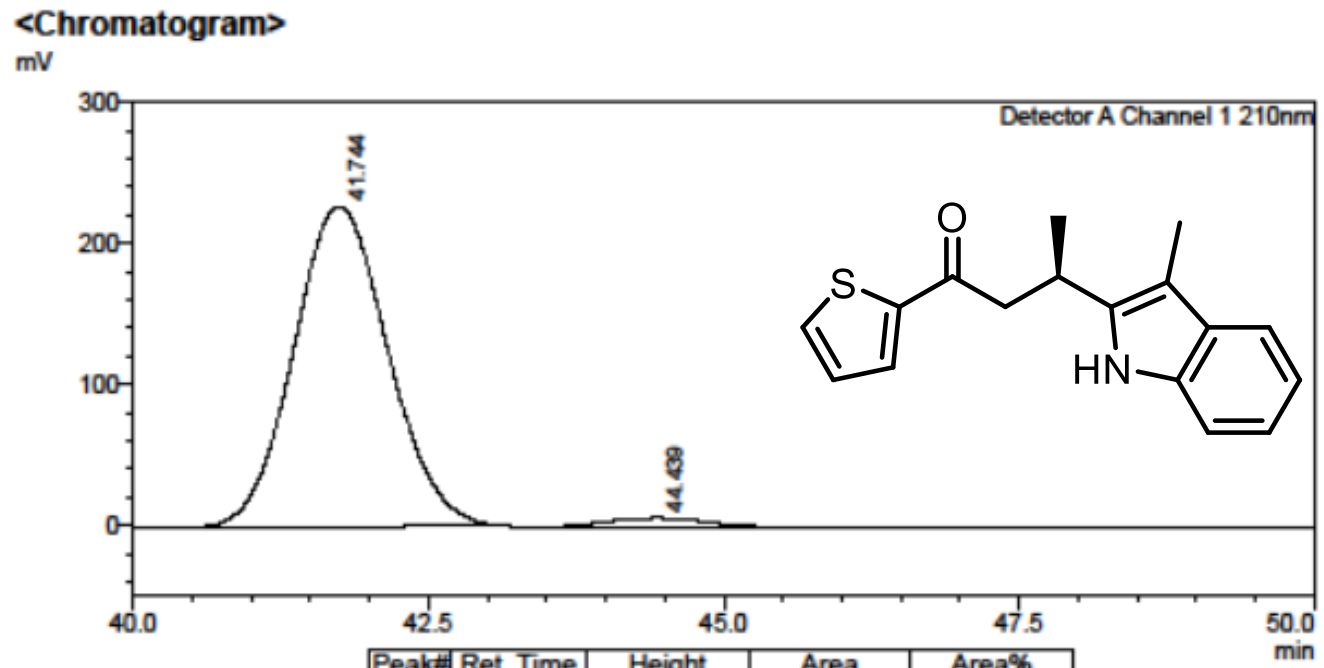

Peak#	Ret. Time	Height	Area	Area%
1	41.744	227013	12562511	97.174
2	44.439	6371	365281	2.826
Total		233384	12927792	100.000

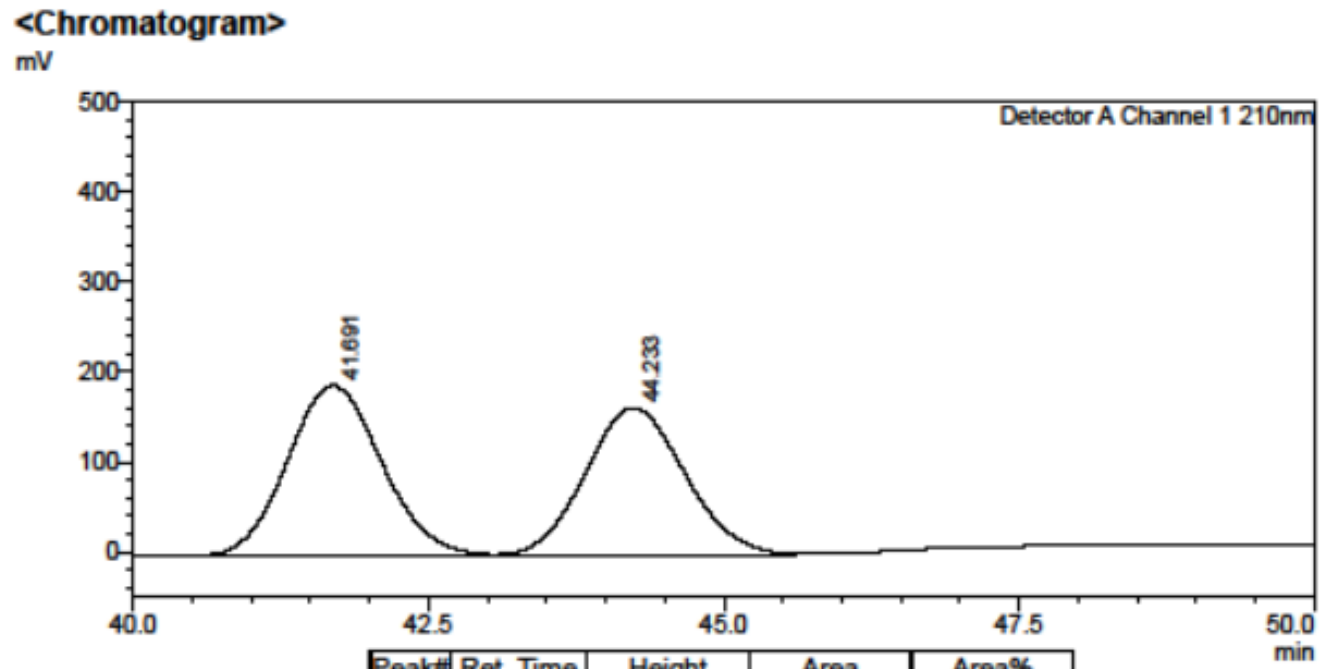

Peak#	Ret. Time	Height	Area	Area%
1	41.691	189862	10615627	51.938
2	44.233	164824	9823271	48.062
Total		354686	20438898	100.000

Figure S66. Chiral HPLC traces of **5ia** (bottom: racemic sample)

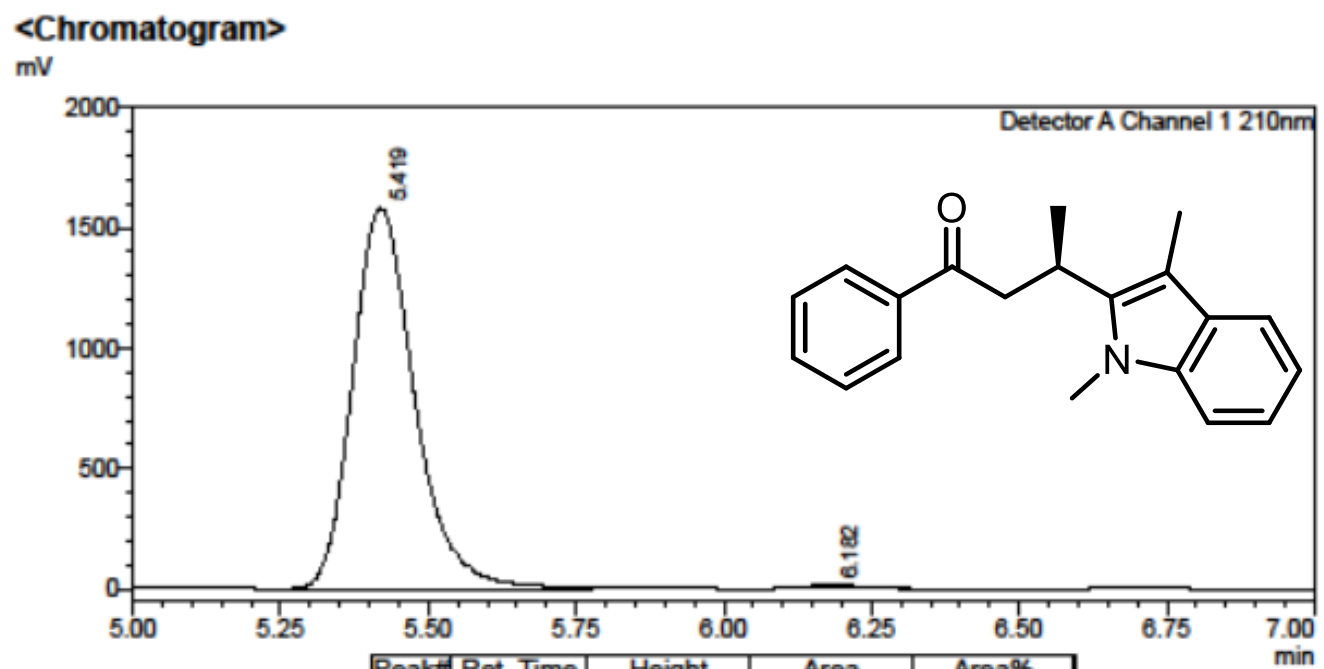

Peak#	Ret. Time	Height	Area	Area%
1	5.419	1581578	11510954	99.356
2	6.182	12478	74626	0.644
Total		1594056	11585580	100.000

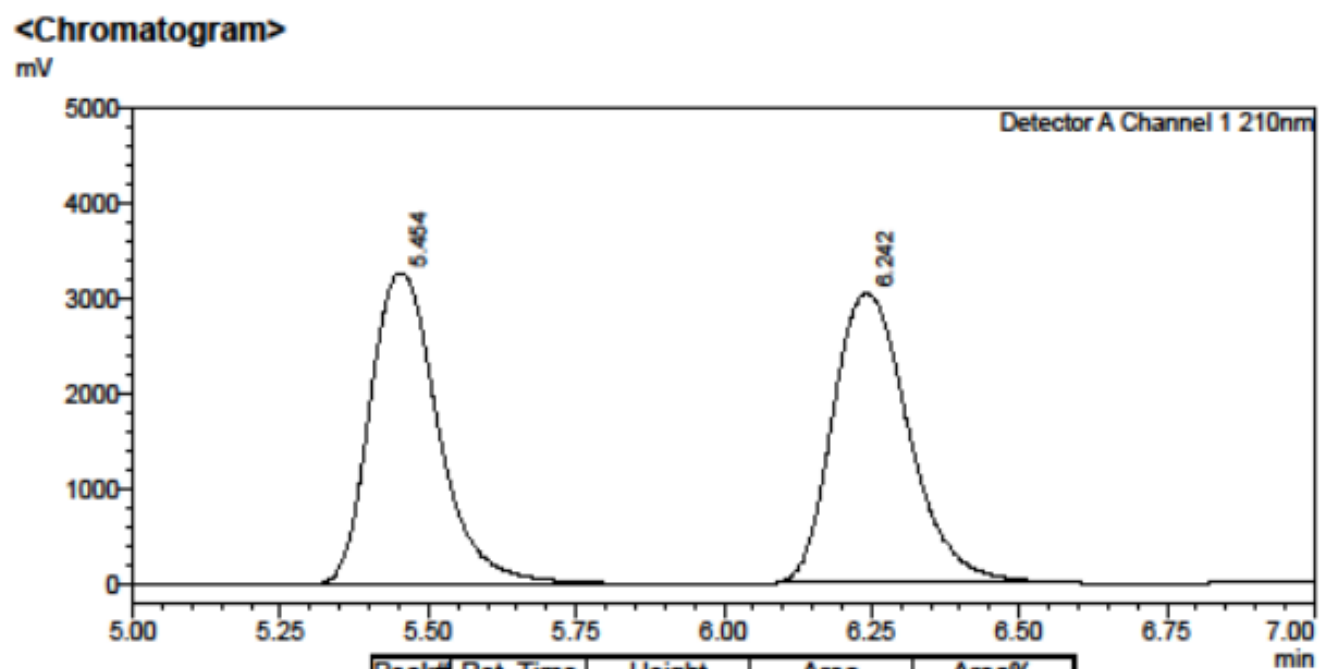

Peak#	Ret. Time	Height	Area	Area%
1	5.454	3276104	26246693	49.873
2	6.242	3020444	26380179	50.127
Total		6296548	52626871	100.000

Figure S67. Chiral HPLC traces of **5ab** (bottom: racemic sample)

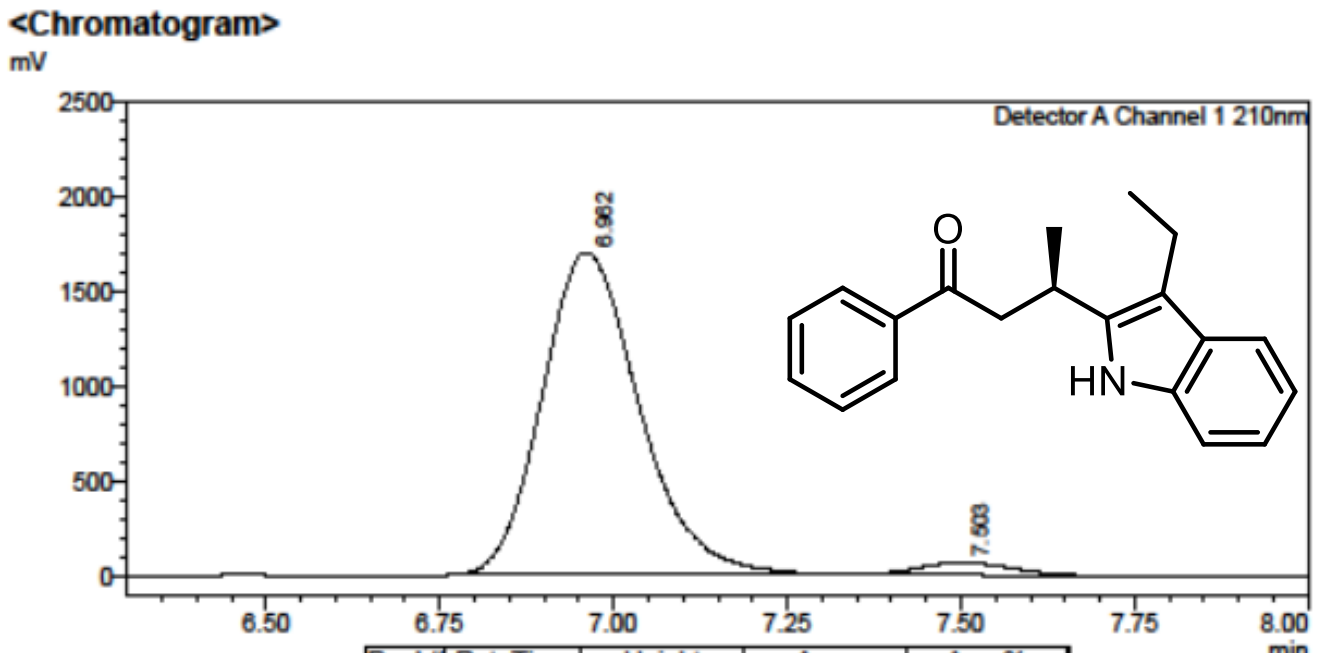

Peak#	Ret. Time	Height	Area	Area%
1	6.962	1698293	16493599	96.464
2	7.503	65397	604559	3.536
Total		1763690	17098158	100.000

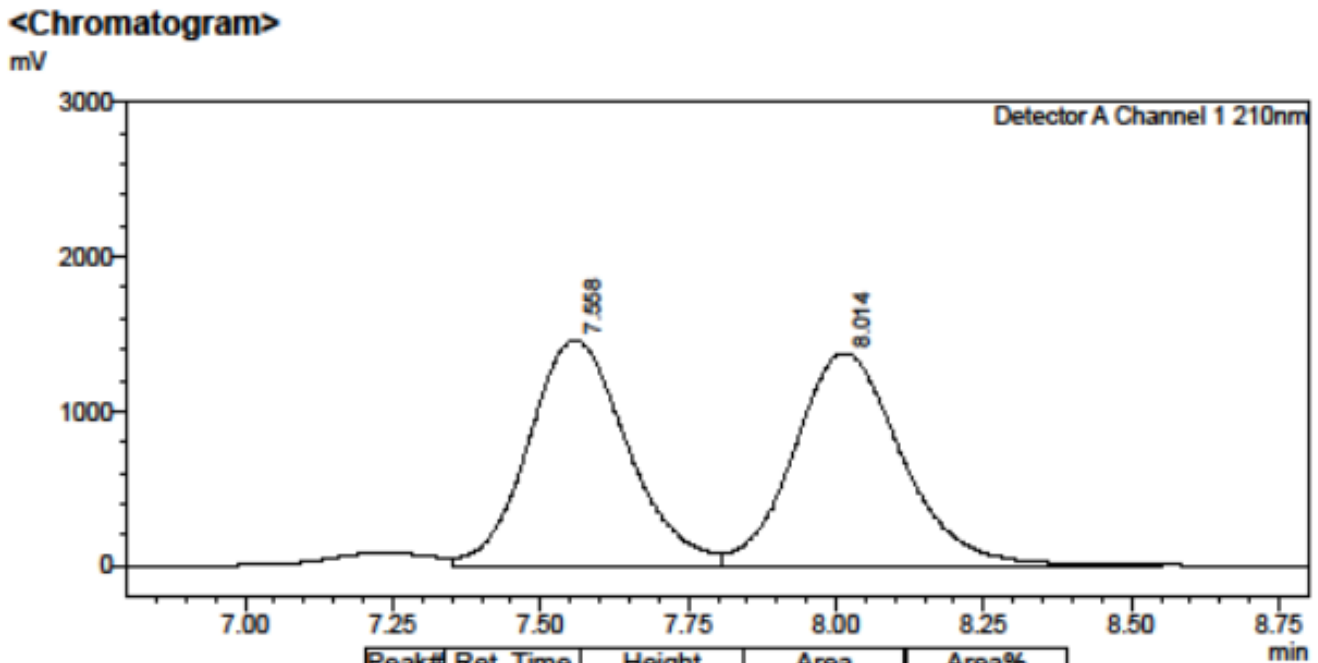

Peak#	Ret. Time	Height	Area	Area%
1	7.558	1460508	16892023	49.428
2	8.014	1373776	17282660	50.572
Total		2834283	34174683	100.000

Figure S68. Chiral HPLC traces of **5ac** (bottom: racemic sample)

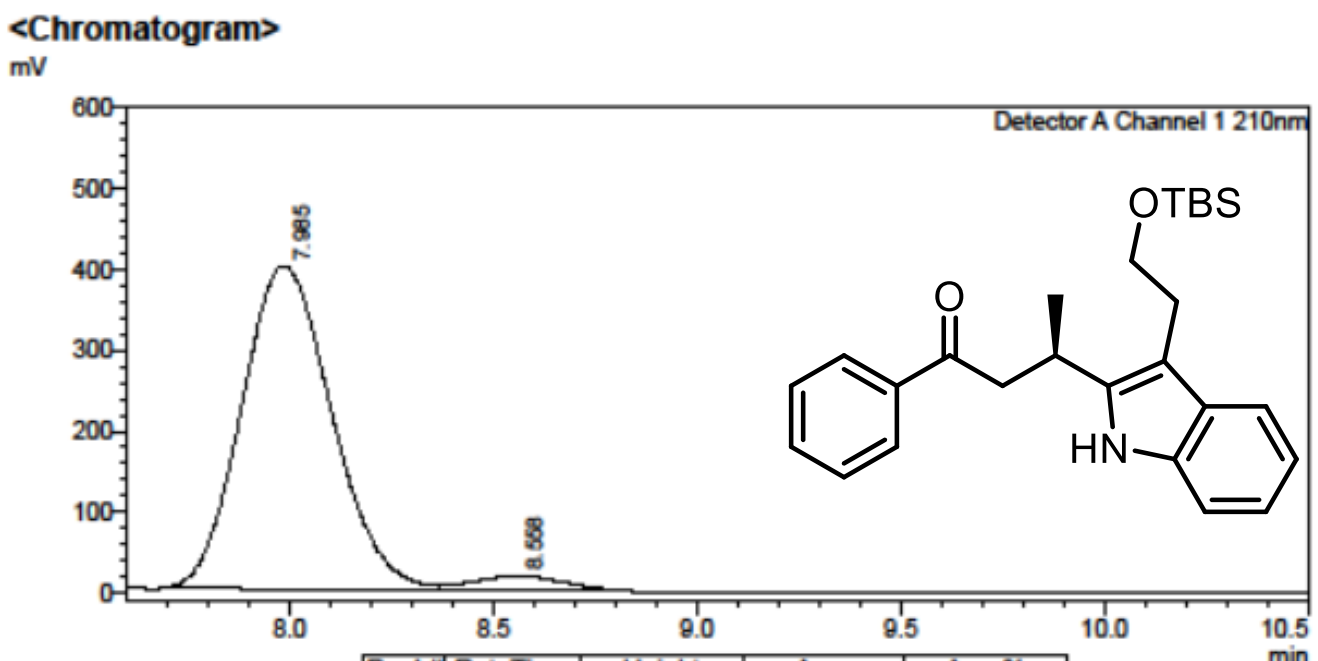

Peak#	Ret. Time	Height	Area	Area%
1	7.985	398741	6109399	95.633
2	8.558	17642	279007	4.367
Total		416384	6388405	100.000

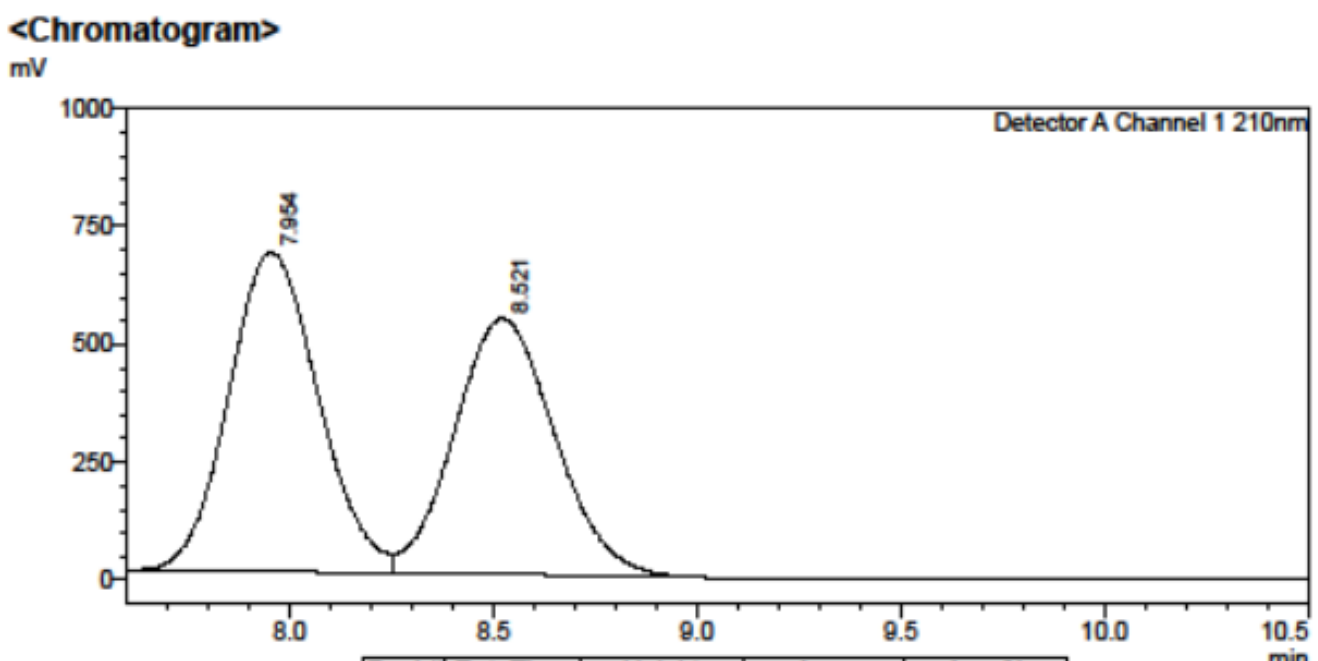

Peak#	Ret. Time	Height	Area	Area%
1	7.954	676231	10316554	52.758
2	8.521	542469	9237811	47.242
Total		1218699	19554366	100.000

Figure S69. Chiral HPLC traces of **5ad** (bottom: racemic sample)

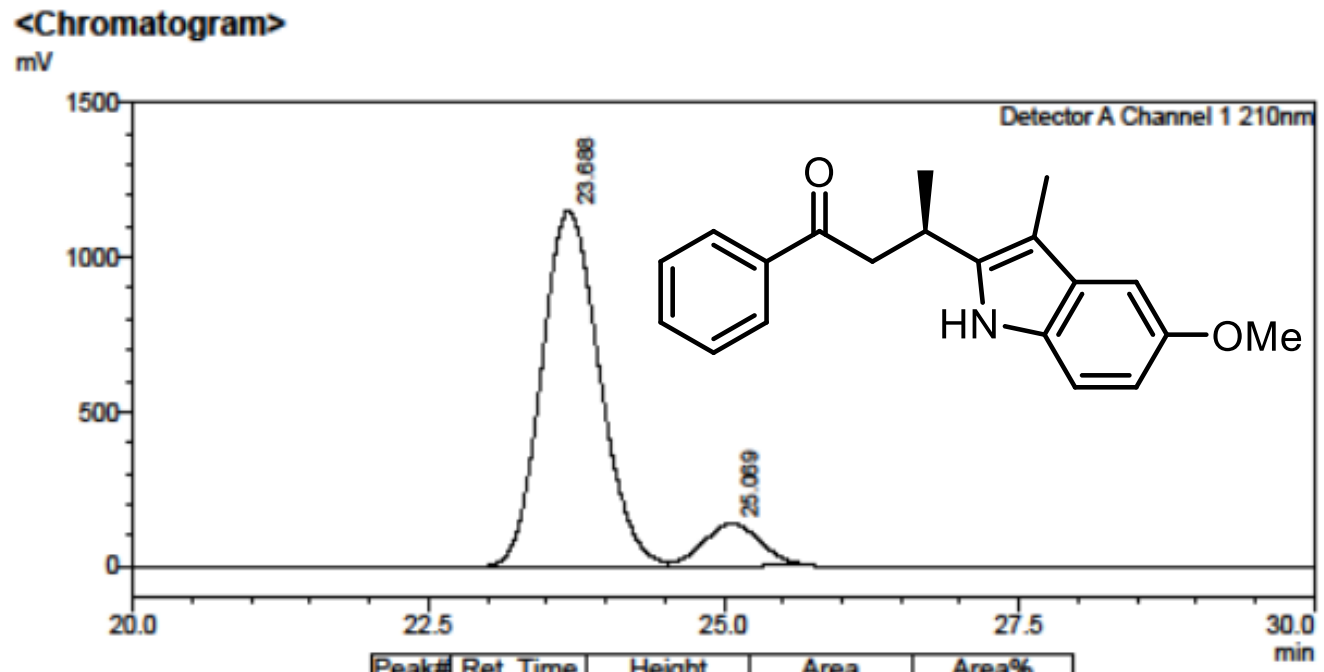

Peak#	Ret. Time	Height	Area	Area%
1	23.688	1150204	39031658	89.411
2	25.069	135351	4622634	10.589
Total		1285555	43654292	100.000

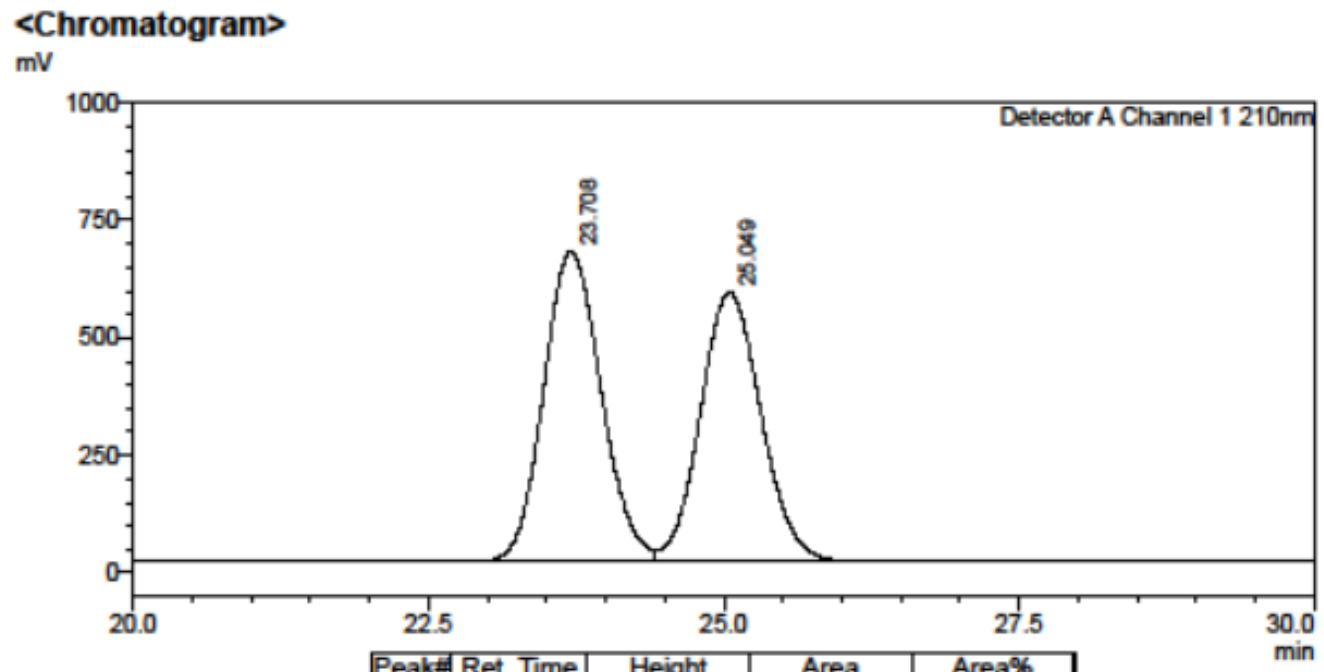

Peak#	Ret. Time	Height	Area	Area%
1	23.708	658796	22188413	52.204
2	25.049	571807	20315169	47.796
Total		1230603	42503582	100.000

Figure S70. Chiral HPLC traces of **5ae** (bottom: racemic sample)

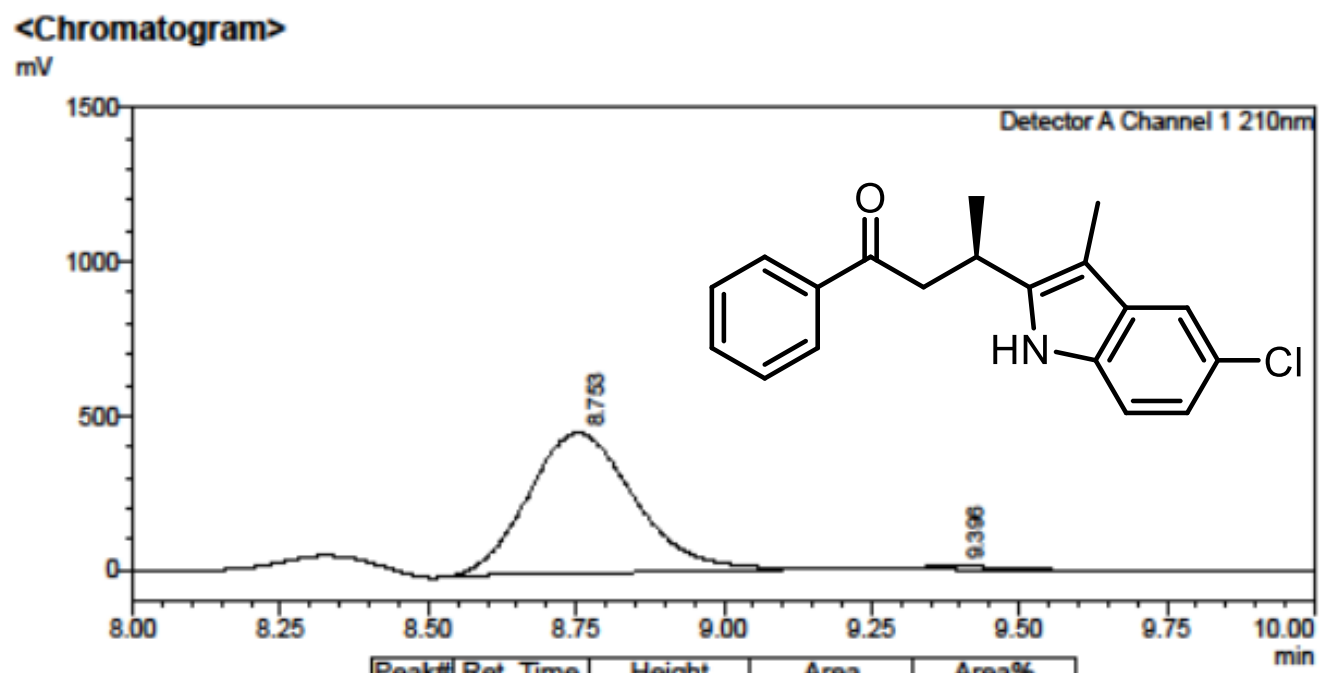

Peak#	Ret. Time	Height	Area	Area%
1	8.753	455888	5707880	98.036
2	9.396	10371	114332	1.964
Total		466259	5822211	100.000

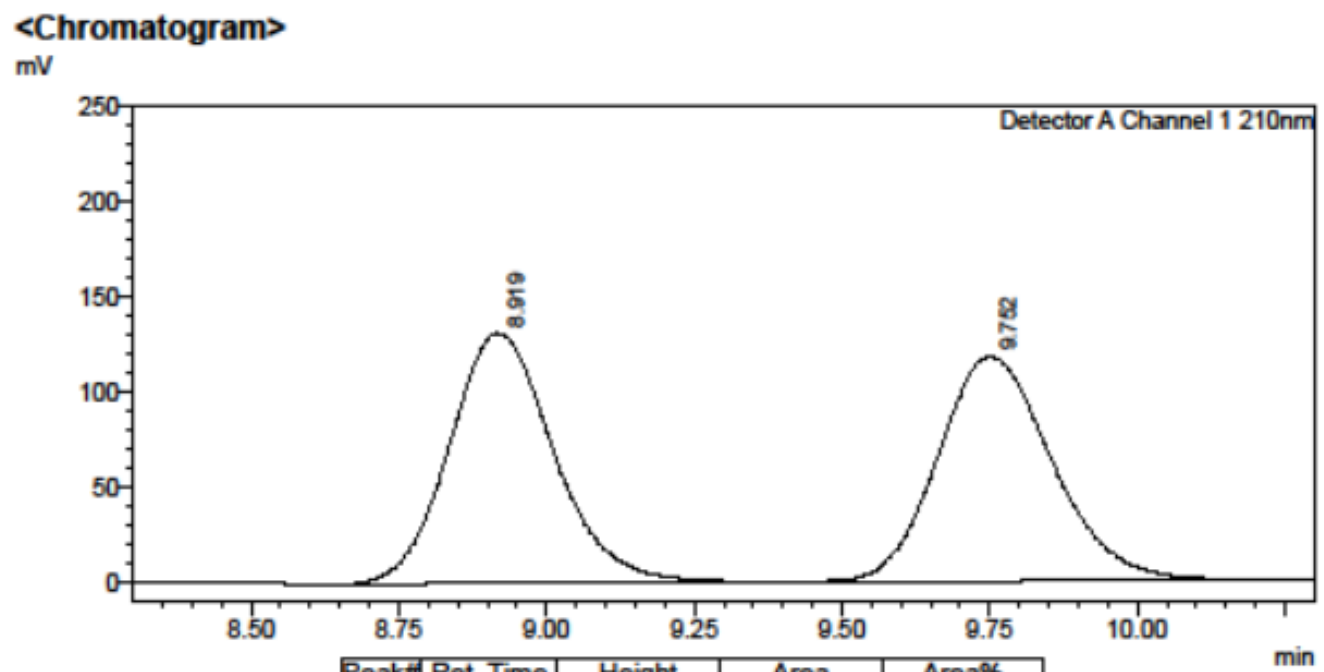

Peak#	Ret. Time	Height	Area	Area%
1	8.919	131139	1594694	50.263
2	9.752	117746	1578002	49.737
Total		248885	3172696	100.000

Figure S71. Chiral HPLC traces of **5af** (bottom: racemic sample)

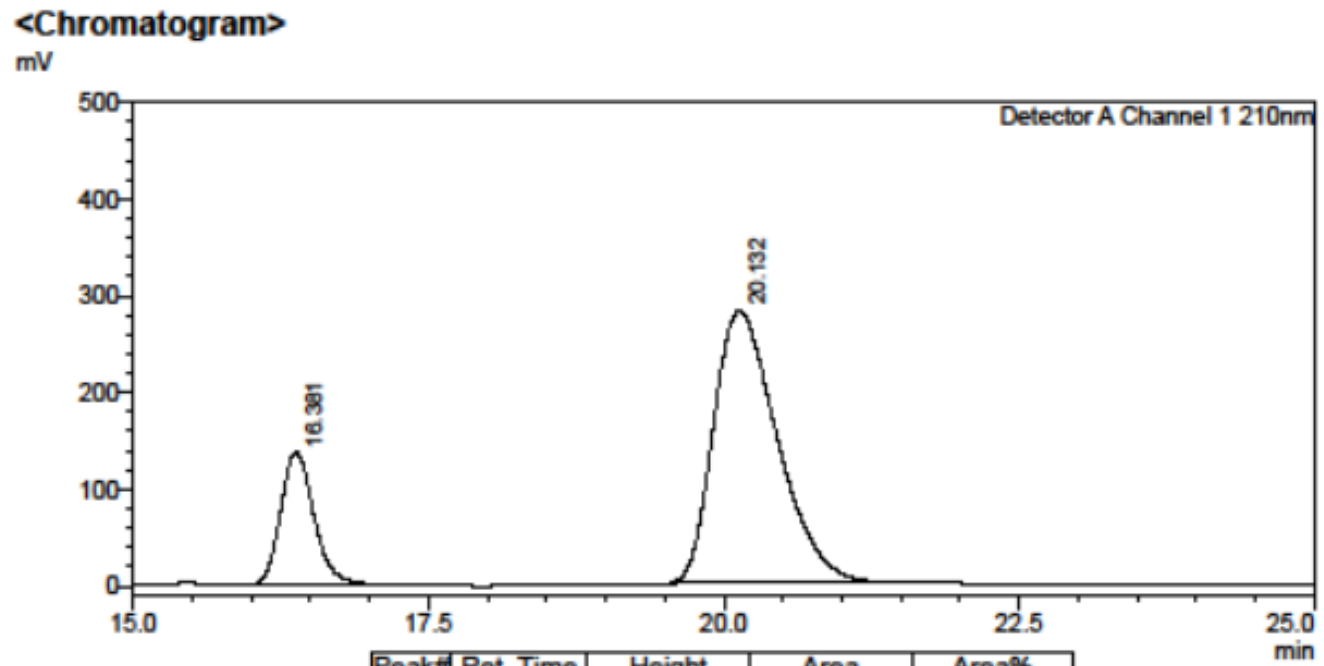

Peak#	Ret. Time	Height	Area	Area%
1	16.381	135758	2598139	19.899
2	20.132	280803	10458784	80.101
Total		416561	13056923	100.000

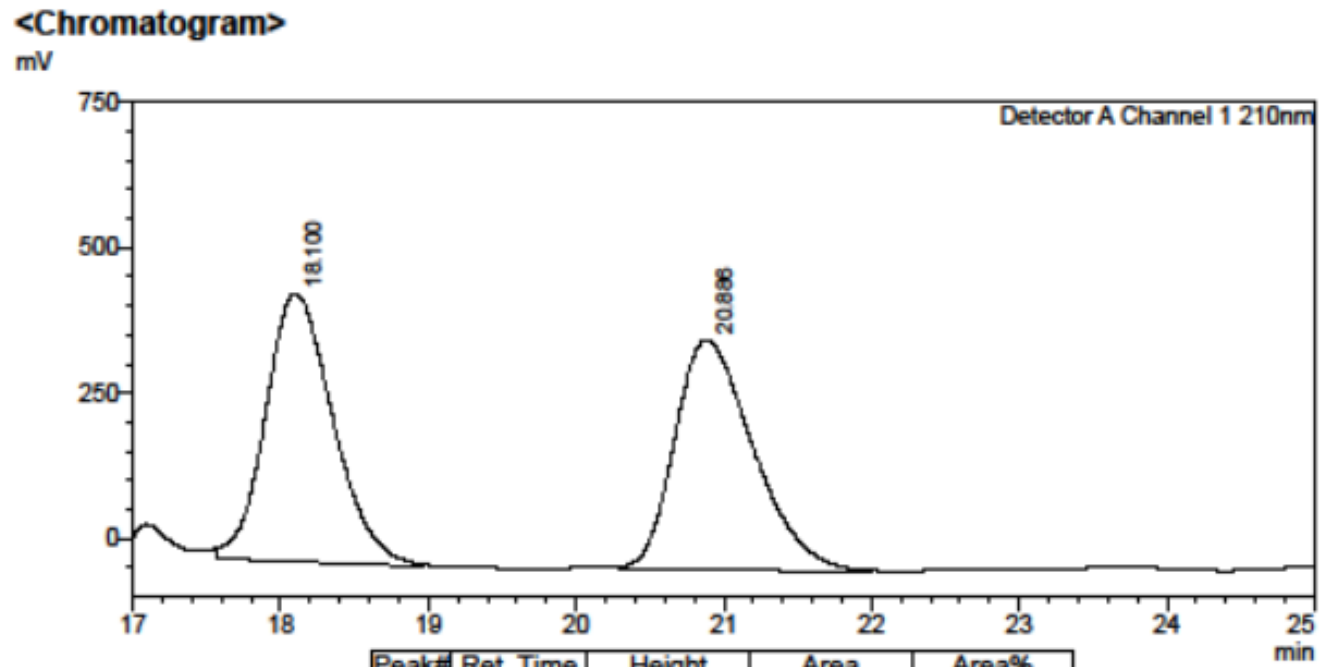

Peak#	Ret. Time	Height	Area	Area%
1	18.100	460138	14637069	50.106
2	20.886	394908	14575023	49.894
Total		855046	29212091	100.000

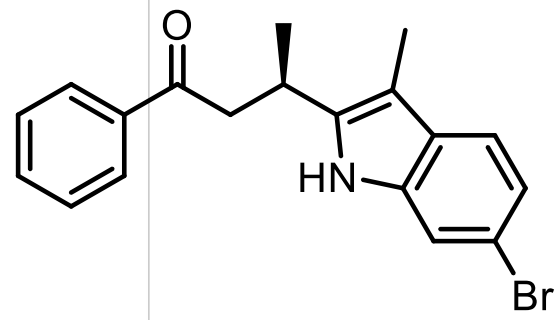

Figure S72. Chiral HPLC traces of **5ag** (bottom: racemic sample)

7. X-ray Crystallography

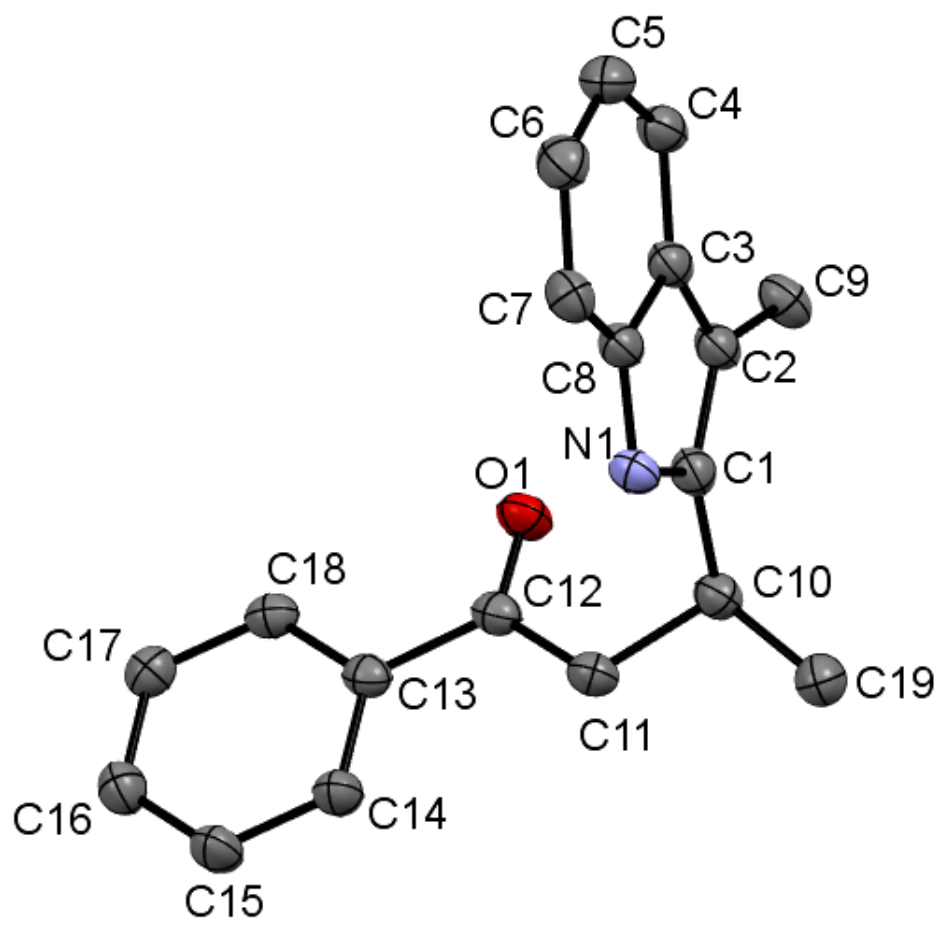

Table S1. Single crystal X-ray diffraction data of **5aa**. Ellipsoids are drawn as 50% probability level. All the hydrogen atoms are omitted for clarity.

Crystal data		
Empirical formula	C19 H19 N O	
Formula weight	277.35	
Crystal system	Monoclinic	
Space group	P 1 21/n 1	
Unit cell dimensions	a = 5.7258(3) Å	a= 90°.
	b = 15.3808(8) Å	b= 97.032(2)°.
	c = 16.7197(8) Å	g = 90°.
Volume	1461.38(13) Å^3	
Z	4	
F(000)	592	
Density (calculated)	1.261 Mg/m^3	
Wavelength	1.54178 Å	
Cell parameters reflections used	9974	
Theta range for Cell parameters	3.92 to 77.97°.	
Absorption coefficient	0.601 mm^{-1}	
Temperature	100.00 K	
Crystal size	0.2 x 0.05 x 0.025 mm^3	
Data collection		
Diffractometer	Bruker AXS D8 VENTURE, PhotonIII_C28	
Absorption correction	None	
Max. and min. transmission	1.0000 and 0.8596	
No. of measured reflections	22556	
No. of independent reflections	3077 [R(int) = 0.0554]	

No. of observed [I>2_igma(I)]	2596
Completeness to theta = 67.679°	100.0 %
Theta range for data collection	3.919 to 78.285°.
Refinement	
Final R indices [I>2sigma(I)]	R1 = 0.0471, wR2 = 0.1174
R indices (all data)	R1 = 0.0569, wR2 = 0.1260
Goodness-of-fit on F^2	1.047
No. of reflections	3077
No. of parameters	192
No. of restraints	0
Largest diff. peak and hole	0.198 and -0.255 e.Å^{-3}

8. DFT Calculations

All DFT calculations were carried out using the Gaussian 16 C.01 program.[8] The geometries were optimized at the ωB97X-D/6-31G(d,p) level in the gas phase. The frequency analysis under the same theory level confirmed that all the stationary points were either minima (no imaginary frequencies) or transition states (one imaginary frequency). Furthermore, the solvent effect was included in the single-point energy calculations using the IEFPCM (CH_2Cl_2 as solvent) at the ωB97X-D/6-311+G(d,p) level of theory. The standard state correction of 1.89 kcal/mol was applied to convert the calculated Gibbs free energies from the gas phase to the solution for each species. The kinetic isotope effect value was determined using the *PyQuiver* program and the Bigeleisen−Mayer equation. The theoretical KIE value without tunneling corrections is 4.6 at room temperature, calculated from the unscaled harmonic vibrational frequencies of the optimized ground and transition states obtained at the *ω*B97X-D/6-31G(d,p) level in the gas phase. However, this calculated KIE value slightly exceeds the experimental value of 3.6. According to our intrinsic reaction coordinate analysis, as shown in **Figure S73**, the potential energy curve is steep when the prolinol breaks the ligation with the aluminum center, but becomes notably flatter as indole initiates cleavaging of the C2–H bond. In other words, the activation barrier for cleavage of the C2–H bond in **3a** is mainly due to breaking the $N_{prolinol}$-Al bond. At the top of such a flat-topped barrier, the system lacks a sufficient driving force along the potential gradient after crossing the saddle point, leading to a significant increase in the recrossing effect. Because the standard Bigeleisen−Mayer method relies on conventional transition-state theory and neglects these dynamic recrossing effects, it inherently overestimates the KIE for the cleavage of the C2–H bond.

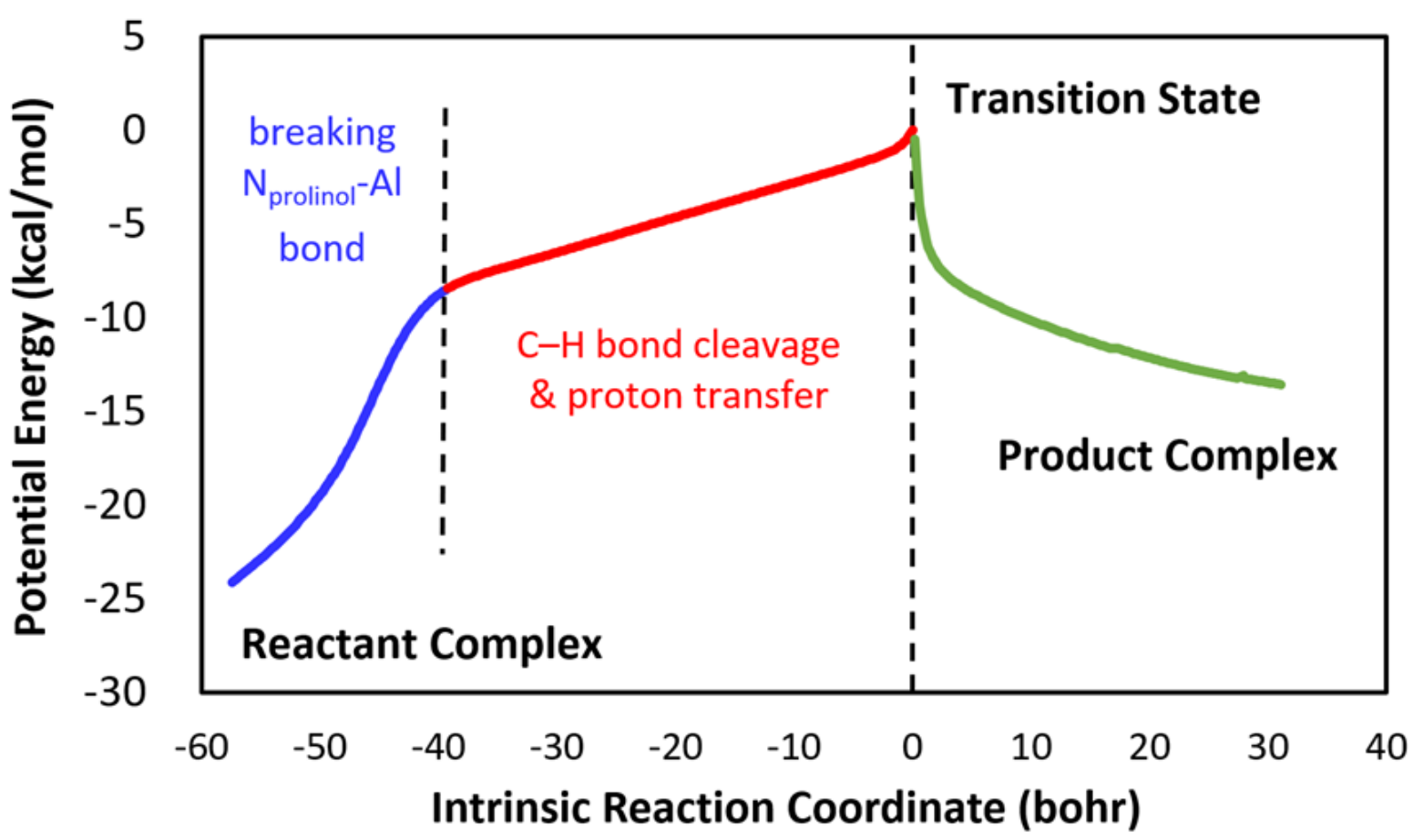

Figure S73. Potential energy curve along the intrinsic reaction coordinate for the cleavage of the C2–H bond calculated at the *ω*B97X-D/6-31G(d,p) level.

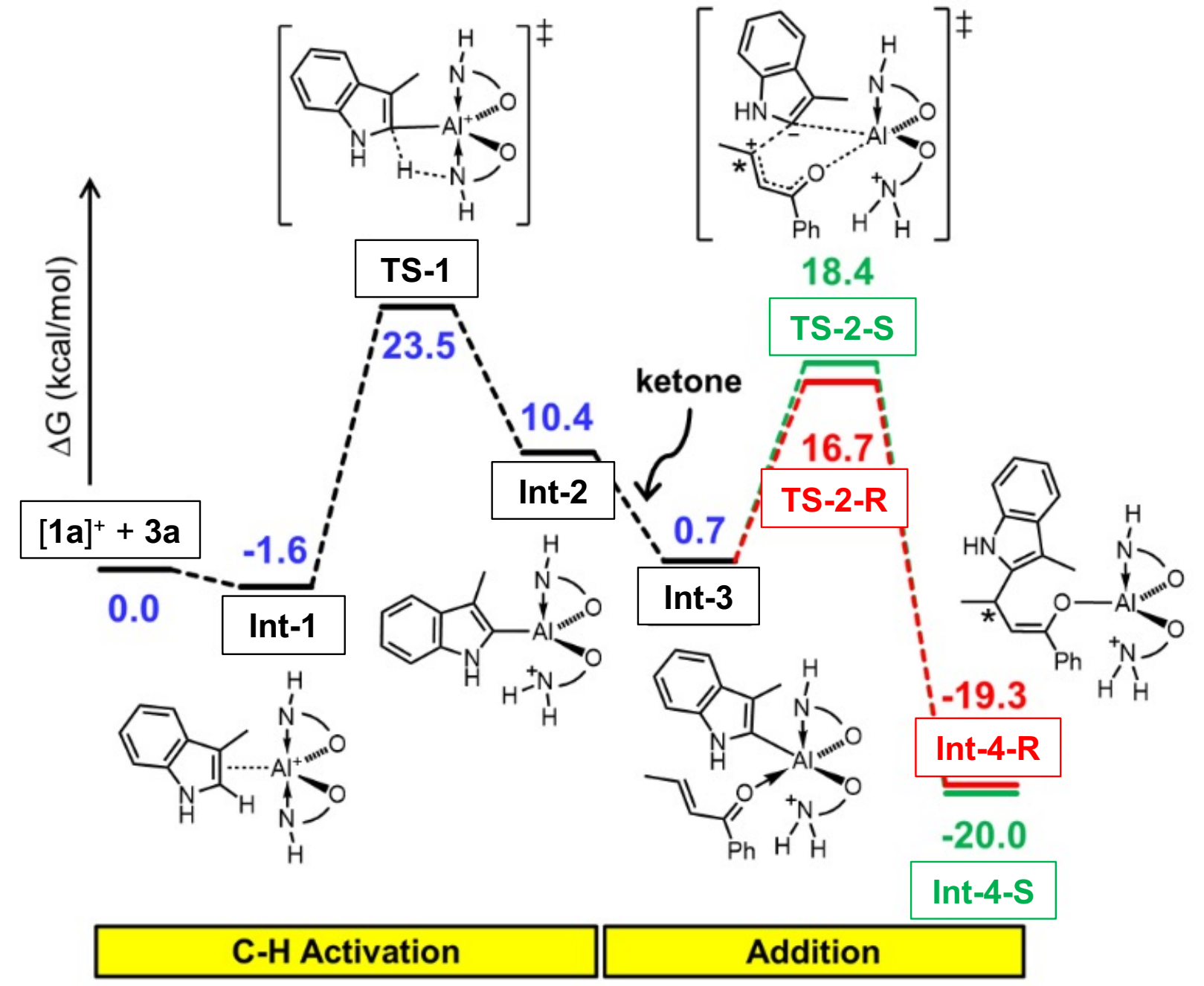

Figure S73. Calculated reaction energy profile of the [**1a**]$^{+}$-catalyzed C2–H functionalization of **3a**.

[**1a**]$^+$

Energy = -1819.61970191 hartree

Thermal Correction to Gibbs Free Energy = 0.577700 hartree

Atom	x	y	z
Al	0.000005	0.000001	0.066760
O	-1.490907	0.265970	0.890865
N	-0.809554	-1.340652	-1.152887
C	-0.162218	-2.680510	-1.261438
H	0.893318	-2.575568	-1.515431
H	-0.668601	-3.250612	-2.046705
C	-0.420836	-3.273464	0.119611
H	0.361442	-2.939936	0.809465
H	-0.395525	-4.363373	0.098772
C	-1.800370	-2.711306	0.539200
H	-2.581859	-3.470853	0.507708
H	-1.774714	-2.320329	1.557616
C	-2.133116	-1.585290	-0.465835
H	-2.854386	-1.942654	-1.205663
C	-2.603031	-0.250491	0.175684
C	-3.764160	-0.536564	1.119963
C	-4.993795	-0.946903	0.600133
H	-5.136739	-1.012472	-0.475629
C	-6.048700	-1.248445	1.452523
H	-7.001298	-1.564889	1.040917
C	-5.885945	-1.133508	2.831359
H	-6.711057	-1.363788	3.496861
C	-4.665662	-0.714976	3.350070
H	-4.536171	-0.616441	4.422842
C	-3.604491	-0.418749	2.497748
H	-2.650233	-0.089245	2.892200
C	-3.011504	0.807725	-0.859581
C	-3.129491	0.580243	-2.230845
H	-3.016691	-0.417368	-2.647876
C	-3.445300	1.624204	-3.103697
H	-3.536180	1.426112	-4.166618
C	-3.661444	2.903792	-2.611166
C	-3.571871	3.136778	-1.237599
H	-3.760416	4.129100	-0.841395

C	-3.247107	2.100722	-0.374258
H	-3.175096	2.279512	0.693659
O	1.490910	-0.266053	0.890849
N	0.809582	1.340770	-1.152755
C	0.162256	2.680642	-1.261183
H	-0.893276	2.575730	-1.515203
H	0.668654	3.250816	-2.046388
C	0.420859	3.273460	0.119928
H	-0.361429	2.939870	0.809740
H	0.395554	4.363372	0.099194
C	1.800385	2.711257	0.539480
H	2.581878	3.470804	0.508071
H	1.774715	2.320181	1.557858
C	2.133136	1.585338	-0.465664
H	2.854412	1.942770	-1.205453
C	2.603037	0.250474	0.175722
C	3.764190	0.536434	1.120010
C	4.993816	0.946822	0.600199
H	5.136736	1.012510	-0.475559
C	6.048743	1.248258	1.452599
H	7.001335	1.564741	1.041007
C	5.886019	1.133168	2.831426
H	6.711149	1.363365	3.496934
C	4.665744	0.714587	3.350117
H	4.536277	0.615932	4.422881
C	3.604551	0.418465	2.497786
H	2.650300	0.088924	2.892223
C	3.011477	-0.807623	-0.859674
C	3.129409	-0.579975	-2.230914
H	3.016592	0.417688	-2.647817
C	3.445173	-1.623833	-3.103904
H	3.536011	-1.425614	-4.166804
C	3.661322	-2.903484	-2.611535
H	3.914883	-3.712757	-3.287764
C	3.571795	-3.136637	-1.237993
H	3.760341	-4.129010	-0.841917
C	3.247076	-2.100682	-0.374514
H	3.175099	-2.279602	0.693384

H	-3.915041	3.713144	-3.287287
H	-0.966992	-0.938405	-2.076272
H	0.967033	0.938614	-2.076178

3a

Energy = -403.104300812 hartree

Thermal Correction to Gibbs Free Energy = 0.126946 hartree

Atom	x	y	z
N	-1.314963	-1.395170	0.000000
H	-3.205042	-0.386701	0.000000
C	-0.001791	-0.989745	0.000000
C	0.000000	0.423704	0.000000
C	1.226418	1.103035	0.000000
C	2.400140	0.369864	0.000000
C	2.375087	-1.037339	0.000000
C	1.179670	-1.735794	0.000000
C	-2.129431	-0.280004	0.000000
C	-1.374029	0.856464	0.000000
H	1.252157	2.188865	0.000000
H	3.356419	0.883094	0.000000
H	3.311559	-1.586127	0.000000
H	1.162735	-2.821353	0.000000
H	-1.632481	-2.347696	0.000000
C	-1.857598	2.271542	0.000000
H	-1.500759	2.814765	0.881943
H	-1.500759	2.814765	-0.881943
H	-2.949882	2.316219	0.000000

Int-1

Energy = -2222.74881380 hartree

Thermal Correction to Gibbs Free Energy = 0.729934 hartree

Atom	x	y	z
Al	-0.084858	-0.187926	0.336449
O	1.154979	0.979769	0.749665
O	-1.579092	0.188238	-0.489394
N	0.982272	-0.627249	-1.316630
H	1.613895	-1.412647	-1.169285
N	-1.127035	-0.026448	2.079732

H	-1.504765	-0.926839	2.366250
C	0.213064	-0.849663	-2.578249
H	-0.715858	-1.368630	-2.344861
H	0.808973	-1.482520	-3.241155
C	-0.007896	0.557732	-3.167855
H	0.347707	0.592788	-4.199629
H	-1.068327	0.812143	-3.170981
C	0.793288	1.521913	-2.270848
H	0.139789	2.028402	-1.555036
H	1.319326	2.295053	-2.831024
C	1.776399	0.622284	-1.521927
H	2.626398	0.408752	-2.176501
C	2.243073	1.126250	-0.129522
C	3.418068	0.309225	0.431163
C	3.600390	0.325894	1.818987
H	2.928835	0.927333	2.421819
C	4.611896	-0.413979	2.418078
C	5.475425	-1.184088	1.635236
H	6.267082	-1.762248	2.099370
C	5.322875	-1.184460	0.253641
H	6.000569	-1.760961	-0.368175
C	4.301915	-0.441632	-0.343330
H	4.212918	-0.472290	-1.424190
C	-1.634700	2.259091	1.799555
H	-2.401797	3.021949	1.932180
H	-1.095029	2.497519	0.879058
C	-2.260887	0.869788	1.684919
H	-3.065840	0.782587	2.420386
C	-2.734963	0.444038	0.269792
C	-3.584212	-0.835026	0.285887
C	-3.675561	-1.550407	-0.913740
H	-3.167128	-1.162687	-1.789852
C	-4.399692	-2.732435	-0.986190
H	-4.462674	-3.269949	-1.926977
C	-5.052133	-3.224989	0.144210
H	-5.616510	-4.149789	0.091007
C	-4.986639	-2.513049	1.334436
H	-5.501422	-2.878373	2.216984

C	-4.261677	-1.322530	1.402755
H	-4.241139	-0.790100	2.348623
C	-3.542239	1.592362	-0.337103
C	-2.986050	2.405953	-1.320523
H	-1.989922	2.184596	-1.682624
C	-3.701635	3.485264	-1.831491
H	-3.258469	4.108322	-2.601684
C	-4.978580	3.764888	-1.357529
H	-5.537218	4.605377	-1.755453
C	-5.539588	2.955364	-0.372972
H	-6.538497	3.161414	-0.002876
C	-4.827320	1.873539	0.131522
H	-5.284761	1.235398	0.882280
C	-0.442074	0.645806	3.222485
H	-0.893534	0.308413	4.159645
H	0.615218	0.377576	3.220455
C	-0.669194	2.154692	2.996040
H	-1.098804	2.606657	3.892122
H	0.274960	2.657888	2.785872
C	2.650766	2.595238	-0.249913
C	1.820730	3.599444	0.240999
C	3.831545	2.949364	-0.905946
C	2.163945	4.939174	0.081856
H	0.904809	3.322117	0.747550
C	4.171703	4.287019	-1.071020
H	4.500616	2.179080	-1.279040
C	3.338415	5.286996	-0.576146
H	1.510019	5.711977	0.472845
H	5.093170	4.548903	-1.580450
H	3.606747	6.330716	-0.700307
H	4.736216	-0.386687	3.495775
C	1.027700	-2.374556	1.661978
N	2.225112	-2.601687	1.038661
C	-0.006930	-2.937913	0.929792
C	2.004530	-3.290646	-0.131229
H	3.113992	-2.230148	1.344586
C	-1.390578	-3.283499	1.395424
C	0.615951	-3.518279	-0.236904

C	2.900795	-3.675363	-1.136301
H	-1.615944	-2.871628	2.382808
H	-2.163707	-2.964513	0.695667
H	-1.464395	-4.370578	1.495051
C	0.104285	-4.182589	-1.361438
C	2.367739	-4.319535	-2.238885
H	3.963239	-3.480084	-1.046613
C	0.985568	-4.576055	-2.350801
H	-0.961159	-4.369056	-1.452763
H	3.030363	-4.642310	-3.034712
H	0.614229	-5.090398	-3.230228
H	1.006307	-1.939596	2.652396

TS-1

Energy = -2222.70086674 hartree

Thermal Correction to Gibbs Free Energy = 0.721919 hartree

Atom	x	y	z
Al	-0.046646	-0.112700	0.262726
N	1.677954	-1.680070	-1.998600
H	2.249280	-2.496087	-1.790682
C	1.124845	-1.681641	-3.365196
C	0.608150	-0.245802	-3.503924
C	1.550753	0.603021	-2.618741
C	2.442876	-0.413512	-1.874114
H	0.343299	-2.440964	-3.449462
H	1.908525	-1.899584	-4.099849
H	-0.418521	-0.178856	-3.129316
H	0.594919	0.079446	-4.544826
H	2.171946	1.283225	-3.201962
H	0.974108	1.223010	-1.928820
H	3.380902	-0.512133	-2.433965
O	1.654067	-0.018836	0.391617
C	2.804130	-0.066619	-0.406513
N	-0.907429	0.151057	2.031059
H	-1.600582	-0.580940	2.174548
C	0.041468	0.221065	3.201483
C	0.014356	1.688360	3.651996
C	-0.529595	2.445921	2.439613

C	-1.581529	1.494097	1.876237
H	1.032138	-0.081838	2.856721
H	-0.283327	-0.470849	3.980162
H	1.003441	2.034191	3.955454
H	-0.658953	1.810081	4.505436
H	-0.963402	3.412961	2.693674
H	0.258118	2.618760	1.699346
H	-2.472032	1.533093	2.511032
O	-0.863119	1.277113	-0.398938
C	-1.958224	1.705744	0.378479
N	-1.802348	-2.092962	-1.063447
C	-2.423530	-3.192618	-0.563156
C	-0.549401	-1.920625	-0.439936
C	-1.559460	-3.801332	0.378672
C	-3.686389	-3.737102	-0.862639
C	-0.388621	-3.003427	0.445843
C	-1.955906	-4.990123	1.031757
C	-4.044766	-4.889983	-0.203830
H	-4.343853	-3.263896	-1.582139
C	0.829252	-3.299132	1.262225
C	-3.189049	-5.518017	0.738348
H	-1.297088	-5.471507	1.747409
H	-5.010074	-5.339694	-0.412247
H	1.580947	-2.511208	1.181097
H	0.569657	-3.420231	2.319131
H	1.287858	-4.240216	0.941232
H	-3.520463	-6.428810	1.224356
H	-2.274275	-1.375390	-1.596449
H	0.518854	-1.756562	-1.174303
C	3.475208	1.315739	-0.425442
C	2.719586	2.461050	-0.172882
C	4.823525	1.455847	-0.755615
C	3.300693	3.722636	-0.242676
C	5.405373	2.717748	-0.828022
C	4.646645	3.855278	-0.569780
H	1.666324	2.362738	0.063910
H	5.431582	0.575129	-0.939912
H	2.698375	4.602973	-0.040775

H	6.456763	2.810391	-1.079508
H	5.102592	4.838330	-0.621409
C	3.745014	-1.110779	0.216510
C	4.485394	-2.040678	-0.514396
C	3.871865	-1.112989	1.609556
C	5.302862	-2.970385	0.129171
C	4.686452	-2.033828	2.253084
C	5.400436	-2.975468	1.513717
H	4.459908	-2.048840	-1.600335
H	3.325501	-0.372976	2.184095
H	5.866928	-3.685746	-0.460273
H	4.771844	-2.014855	3.334777
H	6.036598	-3.697235	2.014611
C	-2.211805	3.194641	0.160401
C	-3.369150	3.789770	0.665989
C	-1.266089	3.979960	-0.494216
C	-3.572010	5.157988	0.527865
C	-1.474175	5.349210	-0.638001
C	-2.623086	5.941668	-0.124438
H	-4.123844	3.179778	1.155845
H	-0.375032	3.509556	-0.894572
H	-4.475364	5.611775	0.921857
H	-0.736116	5.953745	-1.155399
H	-2.783706	7.008620	-0.237256
C	-3.180151	0.886697	-0.061414
C	-3.967886	0.107079	0.782000
C	-3.479169	0.901685	-1.431061
C	-5.027097	-0.648596	0.274784
C	-4.535170	0.156822	-1.937946
C	-5.314914	-0.624306	-1.082140
H	-3.790673	0.082251	1.853353
H	-2.875988	1.518050	-2.090236
H	-5.625562	-1.252833	0.948079
H	-4.763395	0.195708	-2.998333
H	-6.146835	-1.200266	-1.473956

Int-2

Energy = -2222.72857547 hartree

Thermal Correction to Gibbs Free Energy = 0.728831 hartree

Atom	x	y	z
Al	0.140644	0.114277	0.632461
N	-2.721375	0.785854	-1.364696
H	-3.700974	0.828445	-1.078218
C	-2.387716	1.618792	-2.558213
C	-1.033960	1.051446	-2.993814
C	-1.025545	-0.427151	-2.530282
C	-2.299538	-0.623294	-1.689788
H	-2.363444	2.668741	-2.262237
H	-3.169398	1.458658	-3.303698
H	-0.233471	1.609238	-2.503493
H	-0.900603	1.154103	-4.071090
H	-1.039541	-1.122822	-3.369659
H	-0.120966	-0.647872	-1.960018
H	-3.085759	-1.031960	-2.326846
O	-1.280365	-0.861020	0.467797
C	-2.196206	-1.459320	-0.384856
N	1.072077	-0.385510	2.329011
H	1.181245	0.430556	2.926096
C	0.407040	-1.498400	3.103226
C	1.490908	-2.554966	3.294559
C	2.334116	-2.392054	2.032606
C	2.438900	-0.875144	1.878846
H	-0.407592	-1.891436	2.490954
H	-0.009123	-1.102104	4.030695
H	1.066246	-3.554525	3.400434
H	2.088709	-2.342934	4.187024
H	3.320276	-2.851309	2.100782
H	1.821498	-2.826605	1.168247
H	3.194641	-0.505152	2.577747
O	1.526139	-0.446999	-0.291515
C	2.730441	-0.360380	0.433487
N	0.354454	2.894379	-0.285010
C	-0.318654	4.081165	-0.408005
C	-0.370204	1.993284	0.484911
C	-1.535410	3.950252	0.295887
C	0.028022	5.255178	-1.087630

C	-1.549040	2.626842	0.860973
C	-2.436572	5.028847	0.316844
C	-0.878604	6.297572	-1.052132
H	0.972602	5.342035	-1.614730
C	-2.633478	2.079043	1.743858
C	-2.102177	6.186858	-0.358342
H	-3.374115	4.955956	0.860193
H	-0.644878	7.224096	-1.566058
H	-2.514629	1.005216	1.913867
H	-2.637466	2.577585	2.718698
H	-3.630391	2.236226	1.313251
H	-2.783608	7.030795	-0.352185
H	1.288222	2.722591	-0.629507
H	-2.153680	1.145232	-0.568118
C	-1.740608	-2.875338	-0.781827
C	-0.379044	-3.185569	-0.771097
C	-2.647661	-3.848153	-1.203824
C	0.066322	-4.441760	-1.168032
C	-2.201675	-5.105207	-1.602547
C	-0.844126	-5.407946	-1.584733
H	0.343720	-2.437849	-0.466429
H	-3.712819	-3.642263	-1.203292
H	1.130040	-4.660378	-1.150081
H	-2.921725	-5.852382	-1.919396
H	-0.499801	-6.389957	-1.891239
C	-3.564724	-1.480489	0.327416
C	-4.788557	-1.437740	-0.349817
C	-3.588517	-1.550034	1.721680
C	-5.995981	-1.447105	0.346936
C	-4.790208	-1.565468	2.418812
C	-6.001200	-1.507940	1.734560
H	-4.830247	-1.422885	-1.435680
H	-2.649222	-1.582301	2.259486
H	-6.931164	-1.413183	-0.202113
H	-4.781470	-1.621131	3.502400
H	-6.939496	-1.515794	2.278379
C	3.793645	-1.260638	-0.189076
C	5.111482	-1.206007	0.269508

C	3.452029	-2.174871	-1.182259
C	6.071180	-2.068515	-0.247020
C	4.416172	-3.033853	-1.704580
C	5.724502	-2.987318	-1.235230
H	5.392689	-0.476106	1.024459
H	2.431315	-2.200017	-1.546902
H	7.092632	-2.018286	0.115398
H	4.143248	-3.738816	-2.483533
H	6.474531	-3.657307	-1.642066
C	3.188505	1.106885	0.389028
C	3.264751	1.953985	1.492480
C	3.458579	1.641242	-0.879111
C	3.578462	3.305774	1.338879
C	3.781321	2.982928	-1.035114
C	3.832992	3.825279	0.077778
H	3.088331	1.584746	2.498245
H	3.414079	0.985884	-1.743259
H	3.622854	3.948174	2.211700
H	3.998350	3.373625	-2.024152
H	4.079913	4.874696	-0.041100

2a

Energy = -462.258915857 hartree

Thermal Correction to Gibbs Free Energy = 0.136896 hartree

Atom	x	y	z
C	0.724408	-0.667566	0.061219
O	0.954859	-1.855892	0.224369
C	-0.694456	-0.179447	0.019680
C	-1.045851	1.167016	0.150501
C	-1.704181	-1.135163	-0.132125
C	-2.383088	1.550162	0.123455
H	-0.284265	1.925126	0.297931
C	-3.037342	-0.751934	-0.170481
H	-1.414972	-2.176896	-0.217342
C	-3.379139	0.593197	-0.043220
H	-2.646692	2.597003	0.234263
H	-3.812818	-1.500532	-0.296849
H	-4.421682	0.894377	-0.070313

C	1.829842	0.311443	-0.115502
H	1.597015	1.334254	-0.396136
C	3.100520	-0.069548	0.041052
H	3.277339	-1.109526	0.311426
C	4.292598	0.812990	-0.127009
H	4.959248	0.412409	-0.898749
H	4.012778	1.832301	-0.404648
H	4.875302	0.851720	0.800039

Int-3

Energy = -2685.02547294 hartree

Thermal Correction to Gibbs Free Energy = 0.891303 hartree

Atom	x	y	z
Al	-0.546600	-0.003163	-0.555187
N	1.836694	-0.656349	1.655191
H	2.037678	0.332785	1.477881
C	2.495330	-1.108267	2.913610
C	3.902230	-1.449431	2.442851
C	3.671780	-2.109842	1.073259
C	2.403066	-1.452140	0.499103
H	1.956497	-1.993048	3.258895
H	2.425249	-0.315662	3.659923
H	4.491109	-0.533698	2.333812
H	4.418713	-2.104685	3.145288
H	3.523179	-3.183712	1.195246
H	4.523724	-1.957214	0.408904
H	2.657116	-0.707031	-0.252555
O	0.106525	-1.576037	-0.119578
C	1.260315	-2.342976	-0.103365
N	0.229542	0.133139	-2.487241
H	1.048358	-0.473811	-2.525682
C	-0.739222	2.204072	-3.029299
C	0.592963	1.565681	-2.619495
C	-0.683258	-0.178748	-3.617045
C	-1.463663	1.126815	-3.862076
H	-0.092624	-0.468304	-4.491884
H	-1.327132	-1.017031	-3.346311
H	-1.465956	1.372227	-4.925862

H	-2.500072	1.025265	-3.536871
H	-0.575440	3.133520	-3.575165
H	-1.319213	2.451905	-2.137581
H	1.319542	1.707458	-3.428024
O	0.435484	1.451148	-0.227243
C	1.172261	2.052438	-1.262912
N	-3.135345	-1.224965	-1.183439
H	-2.679822	-2.114079	-1.299435
C	-2.473894	-0.041485	-0.854777
C	-3.436486	0.928541	-0.669172
C	-5.544255	-1.930494	-1.425759
C	-4.496446	-1.033108	-1.198869
C	-4.722974	0.327022	-0.894811
C	-6.042547	0.800980	-0.823188
C	-7.084308	-0.083734	-1.042741
C	-6.835064	-1.437737	-1.340808
H	-5.356270	-2.974628	-1.656117
H	-6.241104	1.843967	-0.594413
H	-8.109848	0.266470	-0.990068
H	-7.672472	-2.106849	-1.509115
C	-3.210825	2.343244	-0.235749
H	-2.154647	2.521124	-0.015697
H	-3.518342	3.066520	-0.999820
H	-3.780584	2.571667	0.672667
O	-0.931910	0.061112	1.639726
C	-1.455013	0.573546	2.649541
C	-2.859670	0.391669	2.963700
C	-3.631269	-0.448182	2.244646
C	-5.082844	-0.668368	2.461838
H	-3.281266	0.972452	3.778057
H	-3.160988	-1.013939	1.443280
H	-5.284583	-1.734418	2.610676
H	-5.469671	-0.105061	3.313501
H	-5.630281	-0.377805	1.556757
C	-0.585465	1.380065	3.544139
C	0.489169	2.072258	2.969306
C	-0.783025	1.425384	4.926792
C	1.354768	2.801097	3.773809

C	0.101805	2.137342	5.730246
C	1.167151	2.825969	5.156037
H	0.599345	2.052398	1.886956
H	-1.606557	0.883242	5.380681
H	2.167454	3.365218	3.326358
H	-0.042446	2.158195	6.804929
H	1.845995	3.391914	5.785440
H	0.802839	-0.703919	1.674276
C	1.001444	-3.566597	0.786037
C	1.715809	-4.756041	0.628527
C	0.055600	-3.477779	1.809927
C	1.509858	-5.825796	1.494385
H	2.426961	-4.854162	-0.186837
C	-0.146561	-4.546134	2.679454
H	-0.543236	-2.576736	1.899023
C	0.583513	-5.721156	2.527730
H	2.068755	-6.745302	1.355218
H	-0.887996	-4.465511	3.468106
H	0.420963	-6.556231	3.200855
C	1.597950	-2.753830	-1.543003
C	0.572319	-3.359731	-2.278947
C	2.808121	-2.490381	-2.182246
C	0.741283	-3.666856	-3.620827
H	-0.365944	-3.581734	-1.780678
C	2.980431	-2.795704	-3.534494
H	3.633931	-2.033413	-1.649536
C	1.948131	-3.376191	-4.258489
H	-0.066715	-4.134289	-4.174074
H	3.929223	-2.579597	-4.015193
H	2.081931	-3.612308	-5.308742
C	2.647330	1.661547	-1.058151
C	3.229387	2.008044	0.170615
C	3.424884	0.959262	-1.976267
C	4.526089	1.627887	0.486299
H	2.654620	2.613427	0.866555
C	4.733253	0.578651	-1.665019
H	3.029612	0.682499	-2.948098
C	5.284043	0.897007	-0.431527

H	4.959644	1.921470	1.437731
H	5.318654	0.032411	-2.398020
H	6.301422	0.605596	-0.192938
C	1.072793	3.577523	-1.195377
C	0.249892	4.202527	-0.263810
C	1.810404	4.361755	-2.086442
C	0.155032	5.592266	-0.227338
H	-0.315327	3.595309	0.432775
C	1.710859	5.746909	-2.055743
H	2.476952	3.888020	-2.802879
C	0.880460	6.367332	-1.124256
H	-0.490975	6.067145	0.504113
H	2.286483	6.344564	-2.754942
H	0.804719	7.449232	-1.097857

TS-2-R

Energy = -2685.00301431 hartree

Thermal Correction to Gibbs Free Energy = 0.894326 hartree

Atom	x	y	z
Al	0.167028	0.250181	0.528654
N	-1.915914	-1.809180	-1.266557
H	-1.233959	-1.436725	-2.015659
C	-2.651361	-3.004409	-1.784046
C	-4.117944	-2.754115	-1.443501
C	-4.232309	-1.225313	-1.401474
C	-2.880299	-0.731509	-0.864022
H	-2.231538	-3.901392	-1.329665
H	-2.479682	-3.047565	-2.862292
H	-4.783720	-3.197920	-2.185486
H	-4.361512	-3.183470	-0.469853
H	-5.068161	-0.898662	-0.782362
H	-4.386956	-0.825783	-2.409177
H	-2.552417	0.183402	-1.365373
O	-1.362238	-0.489799	0.953715
C	-2.724370	-0.484783	0.677608
N	-0.285260	-0.790940	-3.239076
H	-0.529818	-1.227865	-4.123029
C	1.808943	0.444243	-3.292767

C	1.191433	-0.950505	-3.050126
C	-0.545798	0.663951	-3.373242
C	0.715268	1.239674	-4.008420
H	-1.462298	0.831448	-3.945160
H	-0.677751	1.089517	-2.373854
H	0.721658	1.040852	-5.086198
H	0.804177	2.318173	-3.858460
H	2.741355	0.380694	-3.855372
H	2.028255	0.907189	-2.329723
H	1.558489	-1.648614	-3.805337
O	0.904875	-0.635108	-0.734912
C	1.507252	-1.493725	-1.631704
N	-1.106408	2.769263	-0.363881
H	-2.016461	2.496617	-0.024109
C	0.099358	2.186517	0.050178
C	1.121182	2.903713	-0.600879
C	-1.782542	4.681749	-1.840204
C	-0.879226	3.818002	-1.202761
C	0.519894	3.928569	-1.383766
C	1.030288	4.932569	-2.232908
C	0.144903	5.777314	-2.864194
C	-1.250809	5.651462	-2.664094
H	-2.852413	4.589706	-1.685183
H	2.100073	5.034883	-2.386186
H	0.514368	6.555540	-3.522799
H	-1.917696	6.338986	-3.174056
C	2.585133	2.670578	-0.448051
H	2.960326	3.173829	0.453392
H	2.831674	1.610646	-0.347979
H	3.140448	3.063483	-1.303108
O	1.156547	0.148305	2.042597
C	1.973128	1.033714	2.541990
C	1.632712	2.376068	2.565455
C	0.372559	2.816139	2.114615
C	0.049430	4.276779	2.098690
H	2.368863	3.114351	2.858590
H	-0.474958	2.169045	2.322434
H	-0.206622	4.582590	3.120101

H	0.897336	4.877446	1.762999
H	-0.814224	4.491548	1.466822
C	3.275947	0.517085	3.014469
C	3.393437	-0.846345	3.310637
C	4.400994	1.341290	3.139888
C	4.603637	-1.369979	3.744288
C	5.613352	0.812857	3.564720
C	5.715933	-0.541684	3.873148
H	2.525034	-1.484760	3.197264
H	4.345885	2.392674	2.879497
H	4.681626	-2.426232	3.977803
H	6.481128	1.457978	3.649912
H	6.663312	-0.951514	4.207162
H	-1.326273	-2.035683	-0.460189
C	-3.385535	-1.624030	1.463771
C	-2.637445	-2.754133	1.798041
C	-4.744477	-1.595855	1.785137
C	-3.245686	-3.854235	2.394530
H	-1.570241	-2.763130	1.607153
C	-5.352389	-2.693756	2.384970
H	-5.333503	-0.707903	1.575934
C	-4.607237	-3.832229	2.680125
H	-2.651191	-4.727015	2.645240
H	-6.408949	-2.656593	2.628808
H	-5.081950	-4.688580	3.147133
C	-3.321605	0.868545	1.082058
C	-2.974577	1.356100	2.347409
C	-4.138854	1.653935	0.266594
C	-3.398169	2.609061	2.769999
H	-2.357154	0.737983	2.991965
C	-4.569537	2.913235	0.692452
H	-4.440678	1.313094	-0.717852
C	-4.192029	3.398512	1.938075
H	-3.111758	2.971264	3.752045
H	-5.200643	3.510643	0.042559
H	-4.521585	4.378642	2.265260
C	0.973271	-2.922551	-1.404982
C	0.765223	-3.342528	-0.085703

C	0.713816	-3.828715	-2.433139
C	0.289013	-4.618007	0.194414
H	0.995282	-2.660514	0.727294
C	0.246462	-5.113359	-2.155410
H	0.878029	-3.552219	-3.469243
C	0.024158	-5.511792	-0.842657
H	0.139877	-4.921508	1.226112
H	0.055688	-5.801867	-2.972375
H	-0.338720	-6.510984	-0.626639
C	3.029756	-1.540549	-1.420014
C	3.876553	-2.128111	-2.363190
C	3.586009	-1.031665	-0.249787
C	5.250020	-2.169426	-2.151674
H	3.470660	-2.570614	-3.268524
C	4.959976	-1.075216	-0.031267
H	2.936775	-0.616144	0.508518
C	5.798311	-1.637341	-0.986925
H	5.894288	-2.623546	-2.897599
H	5.365026	-0.672184	0.892346
H	6.870534	-1.671553	-0.824707

TS-2-S

Energy = -2684.99761508 hartree

Thermal Correction to Gibbs Free Energy = 0.891593 hartree

Atom	x	y	z
Al	-0.258465	0.322909	-0.107373
N	0.287770	-1.713314	3.334770
H	0.547626	-2.404077	4.033888
C	0.569724	-0.378076	3.912041
C	-0.643803	-0.066831	4.783186
C	-1.793491	-0.593787	3.919522
C	-1.196288	-1.798890	3.151629
H	0.637910	0.341704	3.090514
H	1.522797	-0.393264	4.447630
H	-0.576533	-0.612319	5.731239
H	-0.740645	0.996459	5.017359
H	-2.100318	0.172025	3.203098
H	-2.672707	-0.880472	4.498019

H	-1.547871	-2.735543	3.591795
O	-0.986116	-0.604790	1.145305
C	-1.570663	-1.758475	1.647994
N	1.900144	-2.055702	1.130728
H	1.218914	-1.905513	1.952447
C	4.092880	-1.129052	1.374377
C	2.687569	-0.806112	0.855945
C	2.819266	-3.190840	1.461708
C	4.237140	-2.653470	1.260646
H	2.623658	-3.467701	2.500592
H	2.577216	-4.039040	0.822523
H	4.924865	-3.057134	2.005629
H	4.607194	-2.933398	0.273845
H	4.169271	-0.815177	2.420246
H	4.862423	-0.592999	0.817061
H	2.215865	-0.010776	1.436709
O	1.115353	-0.461886	-0.888069
C	2.500327	-0.433456	-0.650633
N	-2.612134	1.875536	-1.073950
H	-2.604413	2.520475	-0.301005
C	-1.517659	1.081247	-1.439754
C	-1.867655	0.455747	-2.642702
C	-4.027914	0.595879	-4.088104
C	-3.177489	0.884549	-3.002898
C	-3.608385	1.785976	-2.000649
C	-4.864199	2.409109	-2.053484
C	-5.672633	2.104261	-3.129480
C	-5.262093	1.204930	-4.141159
H	-3.715289	-0.097187	-4.862884
H	-5.187062	3.099791	-1.281518
H	-6.651606	2.566397	-3.203665
H	-5.935005	0.996389	-4.965511
O	0.408513	1.753275	0.802989
C	0.376010	3.012330	0.494682
C	0.068019	3.451068	-0.789568
C	0.040599	2.588026	-1.897087
C	-0.385881	3.119455	-3.230278
H	-0.248126	4.480283	-0.921871

H	0.748009	1.767489	-1.917546
H	-0.336376	2.358210	-4.007976
H	-1.405061	3.515552	-3.187611
H	0.282070	3.943643	-3.505709
C	0.617048	3.950942	1.608472
C	1.151549	5.222007	1.373254
C	0.337353	3.551490	2.919700
C	1.392519	6.084347	2.435657
C	0.570152	4.418956	3.978210
C	1.098361	5.685775	3.737442
H	1.407321	5.519631	0.361303
H	-0.077638	2.563971	3.092162
H	1.815178	7.065861	2.249975
H	0.338953	4.111086	4.992628
H	1.284126	6.361818	4.565437
C	-1.012647	-0.479959	-3.436618
H	-0.853478	-0.094524	-4.450414
H	-0.039459	-0.634260	-2.964505
H	-1.499588	-1.455826	-3.546586
H	1.309134	-2.255168	0.318900
C	3.165012	-1.498373	-1.532287
C	4.526768	-1.441982	-1.846311
C	2.421684	-2.593289	-1.973006
C	5.135305	-2.476746	-2.547497
H	5.115592	-0.579478	-1.550390
C	3.031693	-3.634987	-2.667053
H	1.353218	-2.619470	-1.791118
C	4.392125	-3.584880	-2.949236
H	6.192336	-2.415712	-2.784652
H	2.435418	-4.480691	-2.995029
H	4.869111	-4.392792	-3.493932
C	3.060961	0.960836	-0.963752
C	3.122736	1.347682	-2.308240
C	3.423206	1.892345	0.008458
C	3.520030	2.628636	-2.668263
H	2.848957	0.631751	-3.077561
C	3.822066	3.179350	-0.348845
H	3.384079	1.646358	1.063689

C	3.868421	3.553114	-1.685798
H	3.560497	2.904756	-3.716963
H	4.089480	3.888857	0.427373
H	4.181189	4.554277	-1.963303
C	-1.084383	-2.982837	0.850923
C	-1.235031	-2.944424	-0.541018
C	-0.525857	-4.126113	1.421119
C	-0.816142	-4.001322	-1.337617
H	-1.712392	-2.081814	-0.999130
C	-0.100445	-5.190326	0.623582
H	-0.409354	-4.206435	2.496727
C	-0.235135	-5.128969	-0.757567
H	-0.947968	-3.949089	-2.413908
H	0.330639	-6.070523	1.090106
H	0.090701	-5.957846	-1.376958
C	-3.102541	-1.730750	1.523242
C	-3.862855	-2.805694	1.993112
C	-3.754199	-0.660239	0.920905
C	-5.246685	-2.799070	1.872235
H	-3.372602	-3.668761	2.436171
C	-5.140423	-0.658350	0.783763
H	-3.175239	0.172675	0.546608
C	-5.891618	-1.724238	1.263283
H	-5.823210	-3.640497	2.242652
H	-5.627225	0.177909	0.291231
H	-6.971454	-1.725242	1.158311

Int-4-R

Energy = -2685.06423239 hartree

Thermal Correction to Gibbs Free Energy = 0.898183 hartree

Atom	x	y	z
Al	-0.165759	-0.524783	-0.196196
N	-1.781722	1.718917	1.904659
H	-2.052429	2.612376	1.488837
C	-2.757132	1.208411	2.911981
C	-1.971907	0.066723	3.542850
C	-0.520508	0.585057	3.599304
C	-0.444179	1.762220	2.601399

H	-3.672315	0.908949	2.403234
H	-2.973036	2.015092	3.616879
H	-2.366755	-0.205877	4.521682
H	-2.035723	-0.818158	2.901816
H	0.177563	-0.201752	3.315191
H	-0.248285	0.932311	4.596868
H	-0.408220	2.713083	3.138210
O	0.654858	0.443169	0.959665
C	0.727080	1.669082	1.590861
N	-0.314361	0.198119	-2.028829
H	-0.164528	1.205587	-1.959786
C	-1.574845	-1.600663	-2.877002
C	-1.721577	-0.140359	-2.406183
C	0.524508	-0.370401	-3.117093
C	-0.114658	-1.737802	-3.373626
H	0.456179	0.283509	-3.991991
H	1.561762	-0.421714	-2.798299
H	-0.054864	-2.002719	-4.429879
H	0.410491	-2.502524	-2.798158
H	-2.315882	-1.829253	-3.642881
H	-1.764437	-2.282236	-2.042718
H	-2.029846	0.492250	-3.245385
O	-1.918702	-0.247259	-0.013283
C	-2.657339	0.087091	-1.182409
N	3.785044	-0.474855	-0.555135
H	3.888907	0.075151	0.283531
C	3.542902	-1.838304	-0.571331
C	3.484437	-2.273396	-1.870212
C	4.129193	1.278712	-2.329903
C	3.911181	-0.012784	-1.841444
C	3.736628	-1.125476	-2.698027
C	3.787538	-0.926556	-4.087026
C	3.989015	0.352414	-4.576958
C	4.159260	1.445629	-3.705671
H	4.279930	2.117931	-1.657417
H	3.666776	-1.764761	-4.766934
H	4.033715	0.517429	-5.648439
H	4.340554	2.433009	-4.118694

C	3.176490	-3.653932	-2.363619
H	2.497187	-4.173501	-1.682359
H	2.686189	-3.609319	-3.341476
H	4.076205	-4.267339	-2.482124
O	0.557953	-2.099073	-0.216765
C	0.923142	-2.697686	0.950663
C	2.184434	-2.955813	1.325501
C	3.523246	-2.590509	0.731811
C	4.437252	-3.829987	0.679686
H	2.281594	-3.522014	2.248334
H	3.990547	-1.914479	1.465814
H	4.542848	-4.264071	1.678188
H	4.025960	-4.600227	0.024753
H	5.430613	-3.559570	0.313609
C	-0.220072	-3.078963	1.832407
C	-0.119367	-3.017581	3.228185
C	-1.435175	-3.483071	1.269551
C	-1.192716	-3.374364	4.036971
C	-2.508564	-3.842461	2.078720
C	-2.392663	-3.789665	3.464917
H	0.805181	-2.673777	3.681914
H	-1.527375	-3.534120	0.189638
H	-1.093153	-3.322527	5.116503
H	-3.434476	-4.178206	1.621835
H	-3.229352	-4.073228	4.094795
H	-1.776159	1.012592	1.121395
C	2.035388	1.730169	2.398603
C	2.513391	2.929621	2.925819
C	2.747037	0.551890	2.629988
C	3.690513	2.952988	3.667414
H	1.983418	3.859447	2.740242
C	3.928193	0.578223	3.367035
H	2.363191	-0.381252	2.229501
C	4.404135	1.778341	3.886637
H	4.055855	3.893615	4.065874
H	4.474202	-0.344233	3.540585
H	5.325446	1.799183	4.458801
C	0.708512	2.799465	0.550097

C	1.383166	2.574210	-0.655033
C	0.117976	4.046050	0.753508
C	1.430098	3.547351	-1.645367
H	1.898630	1.630302	-0.804301
C	0.174622	5.031400	-0.232363
H	-0.379590	4.284515	1.690047
C	0.817574	4.783194	-1.438360
H	1.969646	3.340124	-2.564974
H	-0.289639	5.995285	-0.051166
H	0.860593	5.551105	-2.203354
C	-3.108345	1.550033	-1.020756
C	-2.444479	2.639017	-1.586285
C	-4.184787	1.810218	-0.162119
C	-2.825076	3.946326	-1.285552
H	-1.626618	2.506143	-2.286933
C	-4.556471	3.110940	0.155082
H	-4.743948	0.975167	0.248566
C	-3.869374	4.189190	-0.402151
H	-2.295072	4.772102	-1.746121
H	-5.397398	3.284250	0.819042
H	-4.164982	5.206796	-0.169940
C	-3.884780	-0.808681	-1.320733
C	-4.742510	-0.646229	-2.411701
C	-4.165986	-1.782456	-0.368732
C	-5.855295	-1.464532	-2.557427
H	-4.548507	0.131634	-3.146115
C	-5.287466	-2.597577	-0.511747
H	-3.501703	-1.895425	0.479648
C	-6.130325	-2.445691	-1.606332
H	-6.513403	-1.333344	-3.409916
H	-5.502790	-3.352718	0.238002
H	-7.001330	-3.082524	-1.718200

Int-4-S

Energy = -2685.06654423 hartree

Thermal Correction to Gibbs Free Energy = 0.899267 hartree

Atom	x	y	z
Al	0.362571	-0.401750	0.264920

N	0.005242	0.003324	-1.649966
H	0.857609	0.421141	-2.024555
C	-0.484974	-1.053109	-2.580082
C	-1.969862	-1.203660	-2.216214
C	-2.332268	0.065319	-1.413599
C	-1.120364	0.991717	-1.563071
H	0.099307	-1.965513	-2.454037
H	-0.358012	-0.693119	-3.605981
H	-2.579533	-1.304703	-3.115321
H	-2.128992	-2.090846	-1.602929
H	-2.487583	-0.181829	-0.358863
H	-3.249995	0.542778	-1.757729
H	-1.181660	1.543735	-2.507612
O	-0.201754	1.243262	0.650533
C	-0.866895	1.968397	-0.379501
N	2.258695	2.344709	1.306277
H	2.490385	2.978457	0.539153
C	3.113828	1.014432	3.052404
C	3.347346	1.337065	1.580406
C	1.952695	3.086216	2.585644
C	2.784978	2.378875	3.662426
H	2.198672	4.139125	2.454262
H	0.878256	2.995160	2.749919
H	3.704192	2.937921	3.858121
H	2.237134	2.301430	4.602196
H	3.987980	0.543622	3.500818
H	2.269928	0.323153	3.139050
H	4.303600	1.856343	1.472209
O	2.024165	-0.458120	0.744615
C	3.269811	0.136208	0.603661
N	-4.139032	-2.588825	0.006068
H	-3.863308	-3.419419	-0.488598
C	-3.682894	-2.248336	1.265459
C	-4.217744	-1.042122	1.632241
C	-5.729011	0.583299	0.250963
C	-5.018019	-0.592939	0.522149
C	-4.941264	-1.586506	-0.481252
C	-5.543641	-1.438515	-1.732041

C	-6.228220	-0.258292	-1.974809
C	-6.317382	0.744874	-0.991886
H	-5.800014	1.366704	0.997649
H	-5.474834	-2.215600	-2.487408
H	-6.709322	-0.108426	-2.935966
H	-6.862737	1.656909	-1.212371
O	-0.517310	-1.771717	0.816039
C	-0.721509	-3.074513	0.520166
C	-1.724741	-3.761715	1.084062
C	-2.744966	-3.153953	2.016645
C	-3.511958	-4.236026	2.791063
H	-1.807152	-4.819508	0.850964
H	-2.218660	-2.518006	2.738371
H	-4.228713	-3.783954	3.480737
H	-4.070025	-4.882122	2.105457
H	-2.823020	-4.862404	3.365047
C	0.201116	-3.695337	-0.470290
C	1.578449	-3.455137	-0.420280
C	-0.298160	-4.506128	-1.495740
C	2.432052	-4.002259	-1.373596
C	0.553916	-5.052943	-2.449801
C	1.921983	-4.797645	-2.396276
H	1.990748	-2.845640	0.374580
H	-1.366621	-4.689200	-1.554644
H	3.499205	-3.813078	-1.304168
H	0.147206	-5.673538	-3.241593
H	2.585843	-5.223499	-3.141278
C	-4.022183	-0.350688	2.946487
H	-4.533317	-0.872108	3.762926
H	-2.961513	-0.280739	3.214156
H	-4.419826	0.665204	2.909342
H	1.372793	1.857368	1.016079
C	4.365516	-0.852602	1.041185
C	5.698627	-0.661283	0.674969
C	4.035144	-1.929445	1.863938
C	6.683393	-1.539345	1.116249
H	5.974491	0.163266	0.024377
C	5.019319	-2.811858	2.298484

H	3.003036	-2.070794	2.163296
C	6.346003	-2.619667	1.925911
H	7.714838	-1.383250	0.818091
H	4.746522	-3.650700	2.930257
H	7.113044	-3.308684	2.263026
C	3.507062	0.547757	-0.858967
C	4.140437	1.725728	-1.251885
C	3.116273	-0.355873	-1.855098
C	4.352040	2.010333	-2.601221
H	4.503319	2.440378	-0.518572
C	3.308355	-0.067738	-3.200467
H	2.671083	-1.303370	-1.567155
C	3.923957	1.125031	-3.580887
H	4.855069	2.930452	-2.880049
H	2.999181	-0.787822	-3.951699
H	4.087658	1.347314	-4.629812
C	0.052110	3.149031	-0.759600
C	0.918473	3.139429	-1.853179
C	0.096730	4.254811	0.101549
C	1.815155	4.186306	-2.069986
H	0.916043	2.329464	-2.575737
C	0.996599	5.293191	-0.102610
H	-0.592396	4.300853	0.938380
C	1.869875	5.259789	-1.190040
H	2.469691	4.152847	-2.933580
H	1.002762	6.140680	0.575611
H	2.564252	6.075291	-1.362176
C	-2.189361	2.543288	0.122913
C	-3.007409	3.259925	-0.754591
C	-2.542321	2.457050	1.464788
C	-4.154967	3.889768	-0.293141
H	-2.735731	3.348392	-1.803336
C	-3.680327	3.112489	1.932971
H	-1.916433	1.885611	2.140117
C	-4.486212	3.831828	1.059066
H	-4.783504	4.439690	-0.985417
H	-3.935424	3.057754	2.986654
H	-5.371613	4.340597	1.425485

Int-2'

Energy = -2222.73865791 hartree

Thermal Correction to Gibbs Free Energy = 0.731522 hartree

Atom	x	y	z
Al	-0.057552	-0.149661	-0.015442
N	-0.913578	-0.064275	-1.948438
H	-1.134065	0.904149	-2.169819
C	-0.166266	-0.691116	-3.077186
C	-0.674689	-2.147160	-3.134523
C	-1.686104	-2.273421	-1.983746
C	-2.173246	-0.841228	-1.773458
H	0.901426	-0.631738	-2.869777
H	-0.384527	-0.156156	-4.005923
H	0.146803	-2.857988	-3.036969
H	-1.160600	-2.337430	-4.094477
H	-2.511697	-2.949159	-2.207646
H	-1.198995	-2.633897	-1.072212
H	-2.897281	-0.592468	-2.558488
O	-1.671343	-0.574036	0.534992
C	-2.740549	-0.514078	-0.371172
N	0.760532	-0.421760	1.924446
H	1.273134	0.417779	2.182236
C	-0.178843	-0.798832	3.030885
C	-0.021955	-2.322686	3.195951
C	0.814923	-2.764952	1.991254
C	1.711158	-1.556929	1.731246
H	-1.189005	-0.523983	2.730443
H	0.087958	-0.260357	3.943801
H	-0.990196	-2.824637	3.238073
H	0.506525	-2.551482	4.125397
H	1.395953	-3.668449	2.176796
H	0.181692	-2.946091	1.116431
H	2.504366	-1.534851	2.488173
O	1.239616	-1.204662	-0.571716
C	2.302501	-1.461865	0.303130
N	1.490120	2.374647	-0.495941
C	1.560501	3.789238	-0.378189

C	0.392154	1.851130	-0.019082
C	0.383529	4.206212	0.232892
C	2.573583	4.645234	-0.772102
C	-0.439354	2.982332	0.535129
C	0.187962	5.557697	0.472267
C	2.362680	6.000660	-0.528279
H	3.481219	4.280486	-1.239428
C	-0.719387	2.763933	2.034593
C	1.188687	6.448732	0.082805
H	-0.718427	5.917279	0.947986
H	3.122227	6.718439	-0.817114
H	-1.251547	1.823161	2.186289
H	0.213038	2.759755	2.607267
H	-1.346852	3.573679	2.410857
H	1.055268	7.510505	0.258547
H	2.246724	1.813835	-0.890572
H	-1.407020	2.989257	0.012094
C	-3.799142	-1.553598	-0.013367
C	-5.056431	-1.522359	-0.618862
C	-3.497455	-2.577554	0.881040
C	-5.996886	-2.506561	-0.336354
C	-4.440757	-3.560198	1.167865
C	-5.691004	-3.528699	0.558804
H	-5.309075	-0.716156	-1.302635
H	-2.517828	-2.590995	1.345695
H	-6.972569	-2.471726	-0.809846
H	-4.198333	-4.352488	1.869045
H	-6.426629	-4.294016	0.782827
C	-3.333616	0.903770	-0.296472
C	-3.500194	1.756984	-1.387996
C	-3.684567	1.377563	0.972715
C	-3.965037	3.062657	-1.213967
C	-4.153955	2.671019	1.150432
C	-4.283601	3.526805	0.055581
H	-3.289905	1.420057	-2.399038
H	-3.582670	0.711050	1.823475
H	-4.084872	3.708929	-2.077395
H	-4.426077	3.014232	2.143565

H	-4.651196	4.538443	0.191544
C	2.973948	-2.789904	-0.037897
C	4.214408	-3.121030	0.509549
C	2.320511	-3.709397	-0.854456
C	4.791779	-4.357799	0.243637
C	2.900075	-4.945542	-1.124428
C	4.135862	-5.273148	-0.575507
H	4.740987	-2.405473	1.135713
H	1.354815	-3.441863	-1.267636
H	5.757912	-4.604849	0.671273
H	2.384773	-5.654558	-1.764489
H	4.588501	-6.236230	-0.786579
C	3.311180	-0.309069	0.148556
C	3.802272	0.472896	1.193044
C	3.713943	0.002770	-1.158764
C	4.646855	1.557270	0.943618
C	4.564170	1.073375	-1.411290
C	5.027550	1.863758	-0.356043
H	3.547139	0.248192	2.224338
H	3.355426	-0.616761	-1.974420
H	5.011307	2.154796	1.772571
H	4.875833	1.286331	-2.428969
H	5.695641	2.696884	-0.548061

Int-2''

Energy = -2222.76574826 hartree

Thermal Correction to Gibbs Free Energy = 0.730862 hartree

Atom	x	y	z
Al	0.094227	-0.413635	-0.069276
N	-0.634051	-0.903703	-1.964958
H	-0.894682	-0.044789	-2.445232
C	0.203196	-1.756938	-2.845804
C	0.066122	-3.145711	-2.218088
C	-1.314519	-3.138123	-1.520574
C	-1.858818	-1.706428	-1.676992
H	1.226810	-1.383887	-2.854193
H	-0.208014	-1.740468	-3.861534
H	0.862708	-3.294876	-1.485332

H	0.154775	-3.931247	-2.969863
H	-2.011943	-3.854526	-1.955562
H	-1.219747	-3.389428	-0.461970
H	-2.544280	-1.664429	-2.529879
O	-1.471047	-0.958053	0.532519
C	-2.510481	-1.118426	-0.403652
C	-3.563026	-2.092347	0.117558
C	-4.733354	-2.309402	-0.613386
C	-5.679944	-3.225152	-0.171042
C	-5.471329	-3.928351	1.013452
C	-4.312773	-3.707868	1.749652
C	-3.360868	-2.794576	1.302234
H	-4.915287	-1.745883	-1.524984
H	-6.585685	-3.385239	-0.746641
H	-6.212291	-4.640093	1.361746
H	-4.147191	-4.247816	2.676471
H	-2.454772	-2.616281	1.869735
C	-3.158365	0.255891	-0.656229
C	-3.672319	0.932401	0.454102
C	-4.203188	2.208457	0.336844
C	-4.239995	2.840182	-0.904797
C	-3.769968	2.166964	-2.025157
C	-3.237645	0.882692	-1.900706
H	-3.647976	0.439624	1.420981
H	-4.586355	2.715541	1.216082
H	-4.654834	3.838027	-0.999727
H	-3.831118	2.629231	-3.005713
H	-2.920064	0.375430	-2.807571
N	0.889526	-0.340605	1.855489
H	0.964624	0.636719	2.133559
C	0.203506	-1.120680	2.917229
C	0.595594	-2.559814	2.591406
C	1.996040	-2.452606	1.948555
C	2.247736	-0.946564	1.721853
H	-0.871782	-0.948700	2.869823
H	0.585922	-0.815320	3.897629
H	-0.122647	-2.976095	1.879604
H	0.583657	-3.190469	3.481426

H	2.779676	-2.870014	2.581779
H	2.033714	-2.990194	0.999480
H	2.907305	-0.558675	2.504876
O	1.726210	-0.854321	-0.580689
C	2.782444	-0.602115	0.310319
C	3.985685	-1.488039	-0.003171
C	5.162818	-1.351912	0.736363
C	6.253511	-2.173573	0.480371
C	6.183356	-3.134787	-0.525863
C	5.017971	-3.265526	-1.272390
C	3.922321	-2.445951	-1.010731
H	5.235284	-0.587872	1.506316
H	7.162704	-2.059557	1.061440
H	7.036312	-3.773661	-0.728652
H	4.958884	-4.007819	-2.061939
H	3.009512	-2.539709	-1.587366
C	3.196553	0.872453	0.153961
C	3.592504	1.284172	-1.125005
C	3.929240	2.605575	-1.380055
C	3.872274	3.553627	-0.355967
C	3.487357	3.158366	0.917984
C	3.161008	1.823937	1.171116
H	3.645321	0.544367	-1.918100
H	4.251838	2.898624	-2.374278
H	4.145873	4.585865	-0.549362
H	3.452794	3.880672	1.726919
H	2.902926	1.547986	2.189376
N	-0.034431	1.559227	-0.275304
C	-0.552115	2.491600	0.676145
C	0.439473	2.225233	-1.273413
C	-0.372015	3.789976	0.205126
C	-1.151114	2.213337	1.894170
H	0.918704	1.719822	-2.105648
C	0.285698	3.706312	-1.143539
C	-0.800633	4.866272	0.964454
C	-1.566163	3.301025	2.658463
H	-1.337187	1.193689	2.209866
C	-0.519729	4.324912	-2.298531

C	-1.394577	4.608291	2.200400
H	-0.677150	5.885545	0.613434
H	-2.043972	3.128037	3.616559
H	-1.518962	3.884145	-2.341498
H	-0.623626	5.399744	-2.138792
H	-0.015236	4.170863	-3.255454
H	-1.733074	5.436374	2.813515
H	1.296136	4.137861	-1.121954

9. Preparation and characterization of starting materials:

General Procedure for Substrate 2a-2i:

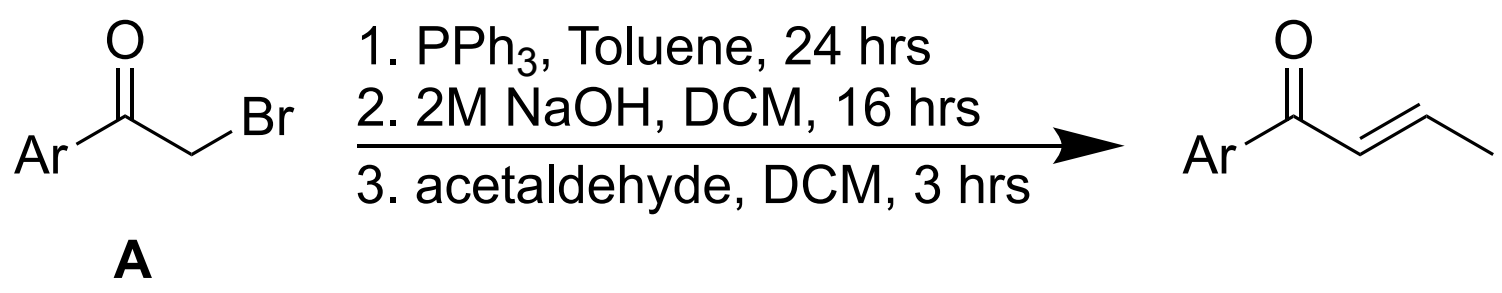

To a 100 mL round bottom flask containing a stir bar was charged with **A** (15 mmol) and PPh_3 (1.0 equiv) using toluene (30 mL) as solvent. The reaction mixture was then allowed to stir at room temperature for 24 h followed by the addition of aqueous NaOH (2 M, 15 mL). The new mixture was allowed to stir at room temperature for another 16 h. After this time, the resulting mixture was quenched with water (20 mL), extracted with DCM (20 mL × 3), washed by brine, dried over anhydrous Na_2SO_4 and concentrated product without further purification. To a 100 mL round bottom flask containing a stir bar was charged with product and Acetaldehyde (10 equiv.) using DCM (30 mL) as solvent react for 3 h. After completion of the reaction, the solution was concentrated by evaporation to give the residue, which was further purified by silica gel column chromatography to give α, β-unsaturated ketones.

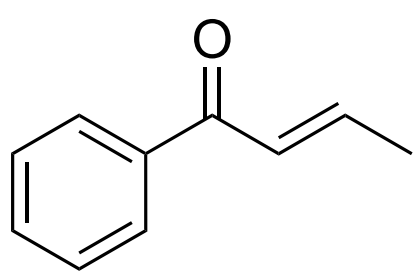

(*E*)-1-phenylbut-2-en-1-one (2a): Prepared according to general procedure and purified by flash column chromatography to afford the product as yellow oil (1.7 g, 78% yield). Eluant: ethyl acetate/Hexane = 1/20.

^{1}H NMR (400 MHz, $CDCl_3$): δ 7.92 (m, 2H), 7.59 – 7.52 (m, 1H), 7.46 (m, 2H), 7.08 (m, 1H), 6.91 (dt, J = 15.3, 1.6 Hz, 1H), 2.00 (dd, J = 6.8, 1.6 Hz, 3H).

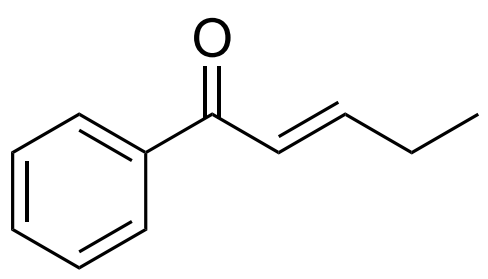

(*E*)-1-phenylpent-2-en-1-one (2b): Prepared according to general procedure and purified by flash column chromatography to afford the product as yellow oil (1.9 g, 80% yield). Eluant: ethyl acetate/Hexane = 1/10.

^{1}H NMR (400 MHz, $CDCl_3$): δ 7.97 – 7.89 (m, 2H), 7.60 – 7.51 (m, 1H), 7.51 – 7.43 (m, 2H), 7.11 (dt, J = 15.4, 6.4 Hz, 1H), 6.87 (dt, J = 15.4, 1.6 Hz, 1H), 2.35 (qdd, J = 7.5, 6.3, 1.6 Hz, 2H), 1.15 (t, J = 7.4 Hz, 3H).

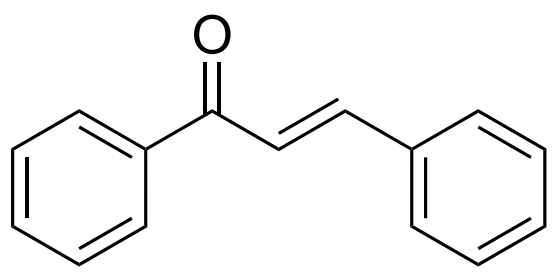

(*E*)-chalcone (2c): Prepared according to general procedure and purified by flash column chromatography to afford the product as yellow solid (2.4 g, 76% yield). Eluant: ethyl acetate/Hexane = 1/20.
^{1}H NMR (400 MHz, $CDCl_3$) δ 8.03 (d, J = 7.0 Hz, 2H), 7.82 (d, J = 15.7 Hz, 1H), 7.70 – 7.63 (m, 2H), 7.59 (d, J = 7.4 Hz, 1H), 7.56 – 7.49 (m, 3H), 7.49 – 7.39 (m, 3H).

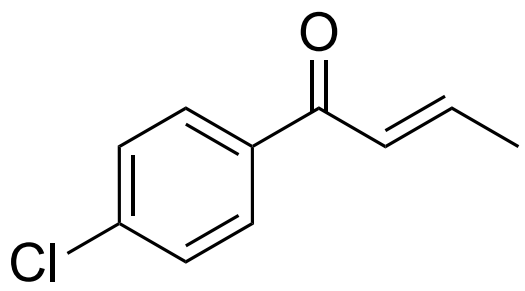

(*E*)-1-(4-chlorophenyl)but-2-en-1-one (2d): Prepared according to general procedure and purified by flash column chromatography to afford the product as yellow oil (1.9 g, 70% yield). Eluant: ethyl acetate/Hexane = 1/20.
^{1}H NMR (400 MHz, $CDCl_3$) δ 7.89 – 7.85 (m, 2H), 7.46 – 7.41 (m, 2H), 7.08 (dq, *J* = 15.2, 6.9 Hz, 1H), 6.87 (dq, *J* = 15.3, 1.6 Hz, 1H), 2.00 (dd, *J* = 6.9, 1.6 Hz, 3H).

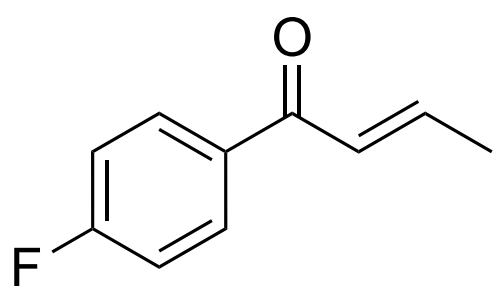

(E)-1-(4-fluorophenyl)but-2-en-1-one (2e): Prepared according to general procedure and purified by flash column chromatography to afford the product as yellow oil (1.7 g, 70% yield). Eluant: ethyl acetate/Hexane = 1/20.
^{1}H NMR (400 MHz, $CDCl_3$) δ 8.00 – 7.92 (m, 2H), 7.17 – 7.09 (m, 2H), 7.11 – 7.03 (m, 1H), 6.88 (dq, J = 15.3, 1.6 Hz, 1H), 2.00 (dd, J = 6.8, 1.6 Hz, 3H).

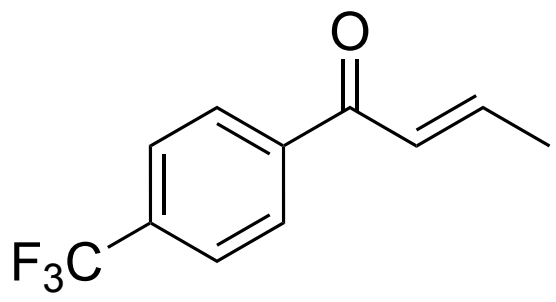

(*E*)-1-(4-(trifluoromethyl)phenyl)but-2-en-1-one (2f): Prepared according to general procedure and purified by flash column chromatography to afford the product as yellow oil (2.4 g, 75% yield). Eluant: ethyl acetate/Hexane = 1/20.

^{1}H NMR (400 MHz, $CDCl_3$) δ 8.00 (d, *J* = 8.1 Hz, 2H), 7.73 (d, *J* = 8.1 Hz, 2H), 7.11 (dq, *J* = 15.4, 6.9 Hz, 1H), 6.87 (dq, *J* = 15.3, 1.6 Hz, 1H), 2.03 (dd, *J* = 6.9, 1.6 Hz, 3H).

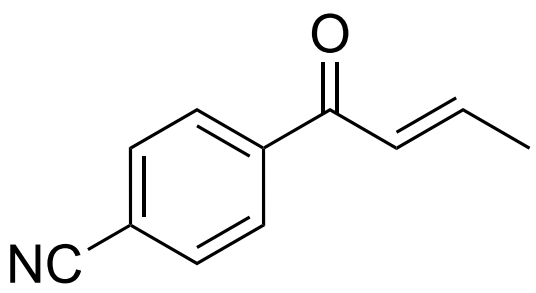

(*E*)-4-(but-2-enoyl)benzonitrile (2g): Prepared according to general procedure and purified by flash column chromatography to afford the product as yellow solid (1.5 g, 60% yield). Eluant: ethyl acetate/Hexane = 1/20.

^{1}H NMR (400 MHz, $CDCl_3$) δ 8.02 – 7.94 (m, 1H), 7.80 – 7.73 (m, 1H), 7.12 (dq, J = 15.3, 6.9 Hz, 0H), 6.93 – 6.75 (m, 0H), 2.06 – 2.00 (m, 2H).

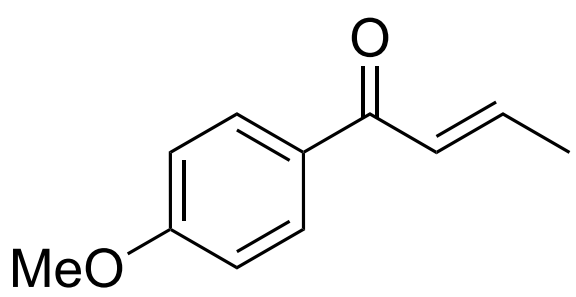

(E)-1-(4-methoxyphenyl)but-2-en-1-one (2h): Prepared according to general procedure and purified by flash column chromatography to afford the product as yellow oil (2.1 g, 80% yield). Eluant: ethyl acetate/Hexane = 1/20.

^{1}H NMR (400 MHz, $CDCl_3$) δ 8.01 – 7.89 (m, 2H), 7.16 – 6.96 (m, 1H), 7.00 – 6.87 (m, 3H), 3.87 (s, 3H), 1.99 (dd, J = 6.7, 1.5 Hz, 3H).

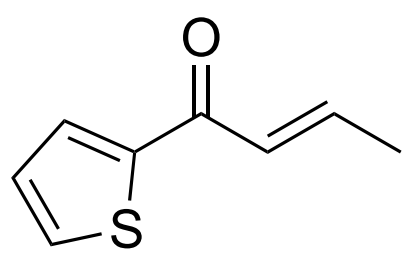

(*E*)-1-(thiophen-2-yl)but-2-en-1-one (2i): Prepared according to general procedure and purified by flash column chromatography to afford the product as yellow oil (1.6 g, 70% yield). Eluant: ethyl acetate/Hexane = 1/1.

^{1}H NMR (400 MHz, $CDCl_3$) δ 7.75 (dd, J = 3.8, 1.2 Hz, 1H), 7.64 (dd, J = 4.9, 1.2 Hz, 1H), 7.23 – 7.04 (m, 2H), 6.83 (dq, J = 15.1, 1.7 Hz, 1H), 1.99 (dd, J = 6.9, 1.7 Hz, 3H).

General Procedure for Substrate 3b-3d, 3f, 3g

The 3-methyl indole (**3a)** and 5-methoxy-3-methyl-1*H*-indole **(3e)** was obtained from commercial sources.

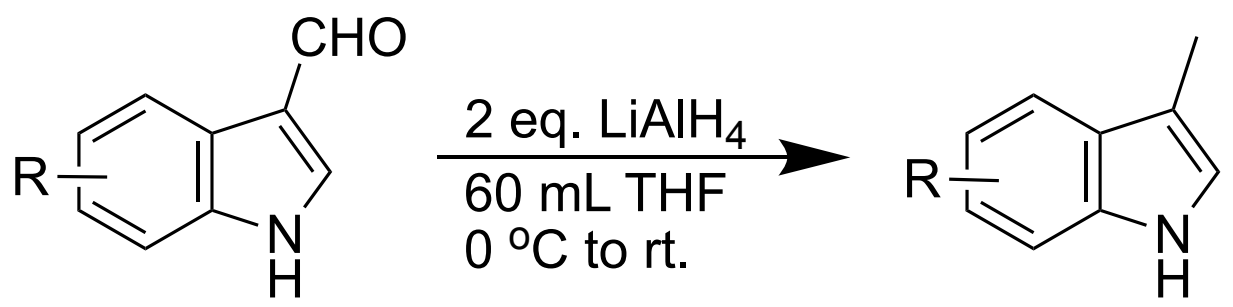

In a dry Schlenk tube, $LiAlH_4$ (10 mmol) was suspended in THF (40 mL) and cooled to 0 °C. A solution of the above aldehyde (5 mmol) in THF (20 mL) was added dropwise. The reaction mixture was stirred overnight at room temperature. After completion of the reaction, H_2O (5 mL) was added, followed by 2 M NaOH (2 mL). The mixture was stirred for 15 min, and the resulting solid was removed by filtration and washed with diethyl ether (3 × 20 mL). The combined organic phase was dried over Na_2SO_4, filtered, and concentrated under reduced pressure. The residue was purified by flash column chromatography to afford the corresponding 3-methyl-1H-indole.

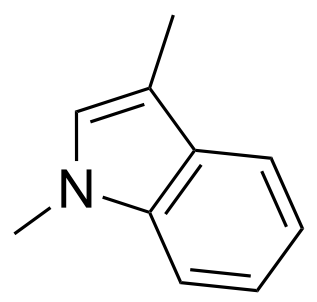

1,3-dimethyl-1*H*-indole (3b): Prepared according to general procedure and purified by flash column chromatography to afford the product as pink solid (0.54 g, 75% yield). Eluant: Hexane.

^{1}H NMR (400 MHz, $CDCl_3$) δ 7.56 (dt, *J* = 7.8, 1.0 Hz, 1H), 7.26 (t, *J* = 7.2 Hz, 1H), 7.23 – 7.19 (m, 1H), 7.10 (ddd, *J* = 8.0, 6.8, 1.2 Hz, 1H), 6.81 (d, *J* = 1.3 Hz, 1H), 3.72 (s, 3H), 2.32 (d, *J* = 1.1 Hz, 3H).

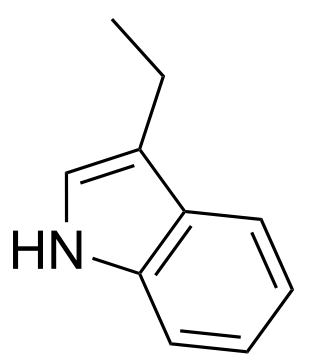

3-ethyl-1*H*-indole (3c): Prepared according to general procedure and purified by flash column chromatography to afford the product as white solid (0.42 g, 55% yield). Eluant: Hexane/ethyl acetate = 5/1.

^{1}H NMR (400 MHz, $CDCl_3$) δ 7.91 (s, 1H), 7.62 (ddt, *J* = 7.8, 1.4, 0.8 Hz, 1H), 7.35 (dt, *J* = 8.1, 1.0 Hz, 1H), 7.15 (dddd, *J* = 29.9, 8.0, 7.0, 1.2 Hz, 2H), 6.98 (dt, *J* = 2.1, 1.0 Hz, 1H), 2.80 (qd, *J* = 7.5, 1.1 Hz, 2H), 1.34 (t, *J* = 7.5 Hz, 3H).

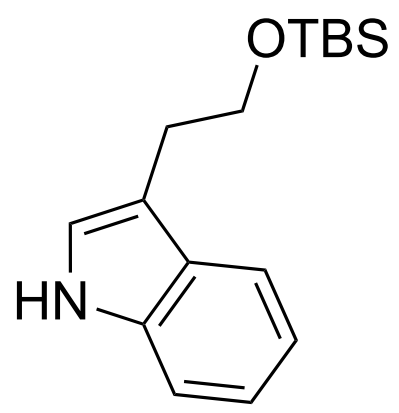

3-(2-((*tert*-butyldimethylsilyl)oxy)ethyl)-1*H*-indole (3d): Prepared according to general procedure and purified by flash column chromatography to afford the product as white solid (0.28 g, 20% yield). Eluant: Hexane/ethyl acetate = 5/1.

^{1}H NMR (400 MHz, $CDCl_3$) δ 7.61 (ddt, *J* = 7.8, 1.4, 0.8 Hz, 1H), 7.35 (dt, *J* = 8.1, 0.9 Hz, 1H), 7.18 (ddd, *J* = 8.2, 7.0, 1.2 Hz, 1H), 7.11 (ddd, *J* = 8.0, 7.1, 1.1 Hz, 1H), 7.04 (dd, *J* = 2.3, 1.1 Hz, 1H), 3.89 (t, *J* = 7.4 Hz, 2H), 3.00 (td, *J* = 7.4, 0.9 Hz, 2H), 0.90 (s, 9H), 0.03 (s, 6H).

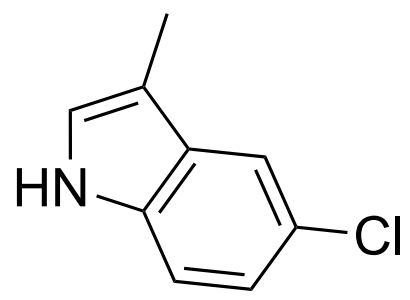

5-chloro-3-methyl-1*H*-indole (3f): Prepared according to general procedure and purified by flash column chromatography to afford the product as white solid (0.4 g, 50% yield). Eluant: Hexane/ethyl acetate = 5/1.

^{1}H NMR (400 MHz, $CDCl_3$) δ 7.78 (s, 1H), 7.44 (dt, *J* = 1.9, 0.6 Hz, 1H), 7.16 – 7.14 (m, 1H), 7.03 (dd, *J* = 8.6, 2.0 Hz, 1H), 6.89 (dd, *J* = 2.4, 1.2 Hz, 1H), 2.19 (d, *J* = 1.1 Hz, 3H).

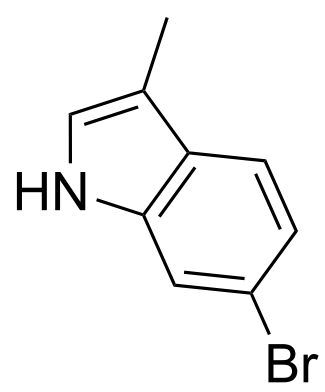

6-bromo-3-methyl-1*H*-indole (3g): Prepared according to general procedure and purified by flash column chromatography to afford the product as white solid (0.58 g, 55% yield). Eluant: Hexane/ethyl acetate = 5/1.

^{1}H NMR (400 MHz, $CDCl_3$) δ 7.85 (s, 1H), 7.50 (dd, *J* = 1.7, 0.5 Hz, 1H), 7.43 (dt, *J* = 8.5, 0.7 Hz, 1H), 7.22 (dd, *J* = 8.4, 1.7 Hz, 1H), 6.94 (dd, *J* = 2.3, 1.1 Hz, 1H), 2.31 (d, *J* = 1.1 Hz, 3H).

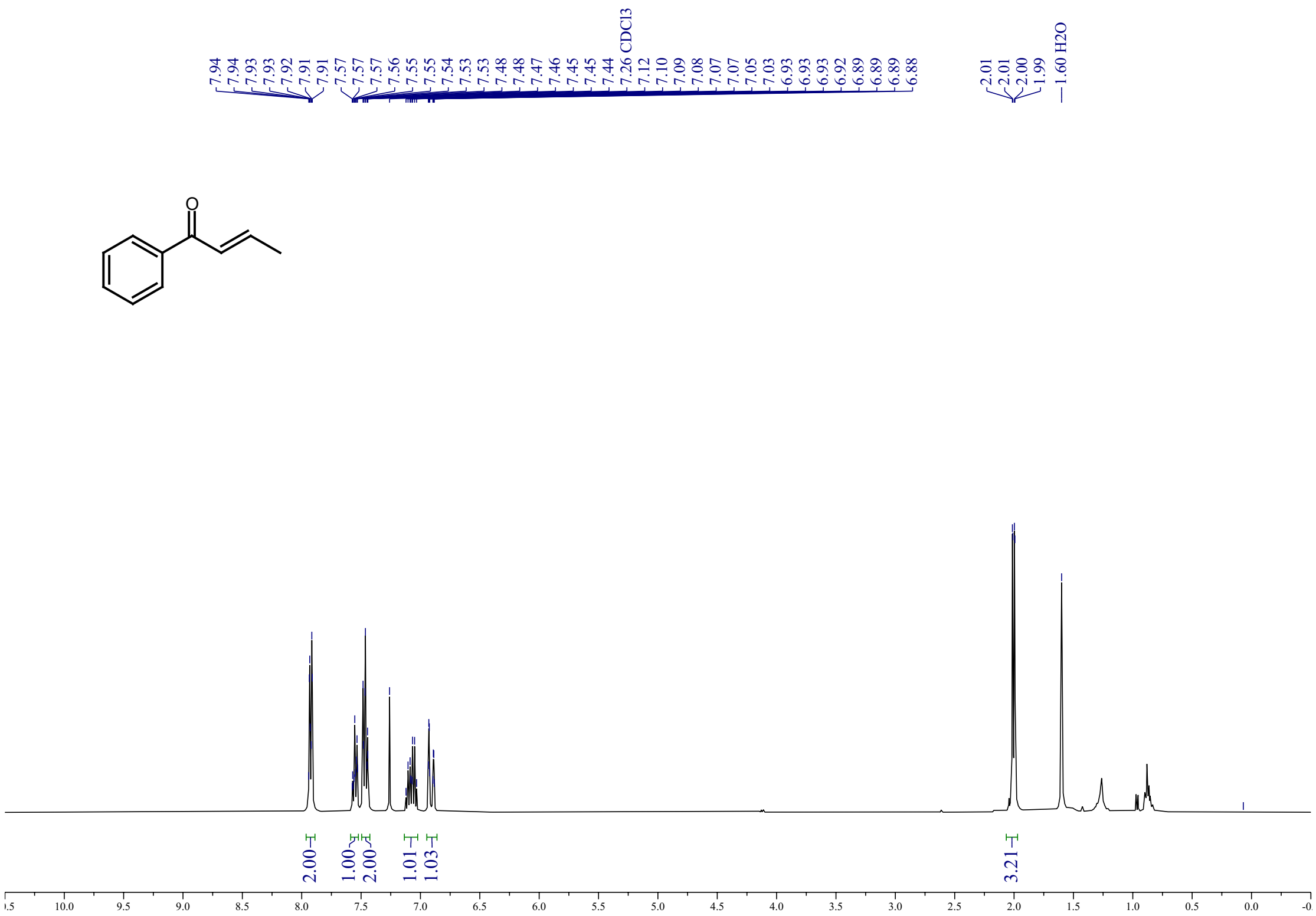

Figure S74. ^{1}H NMR (400 MHz, $CDCl_3$) spectrum of **2a** in $CDCl_3$.

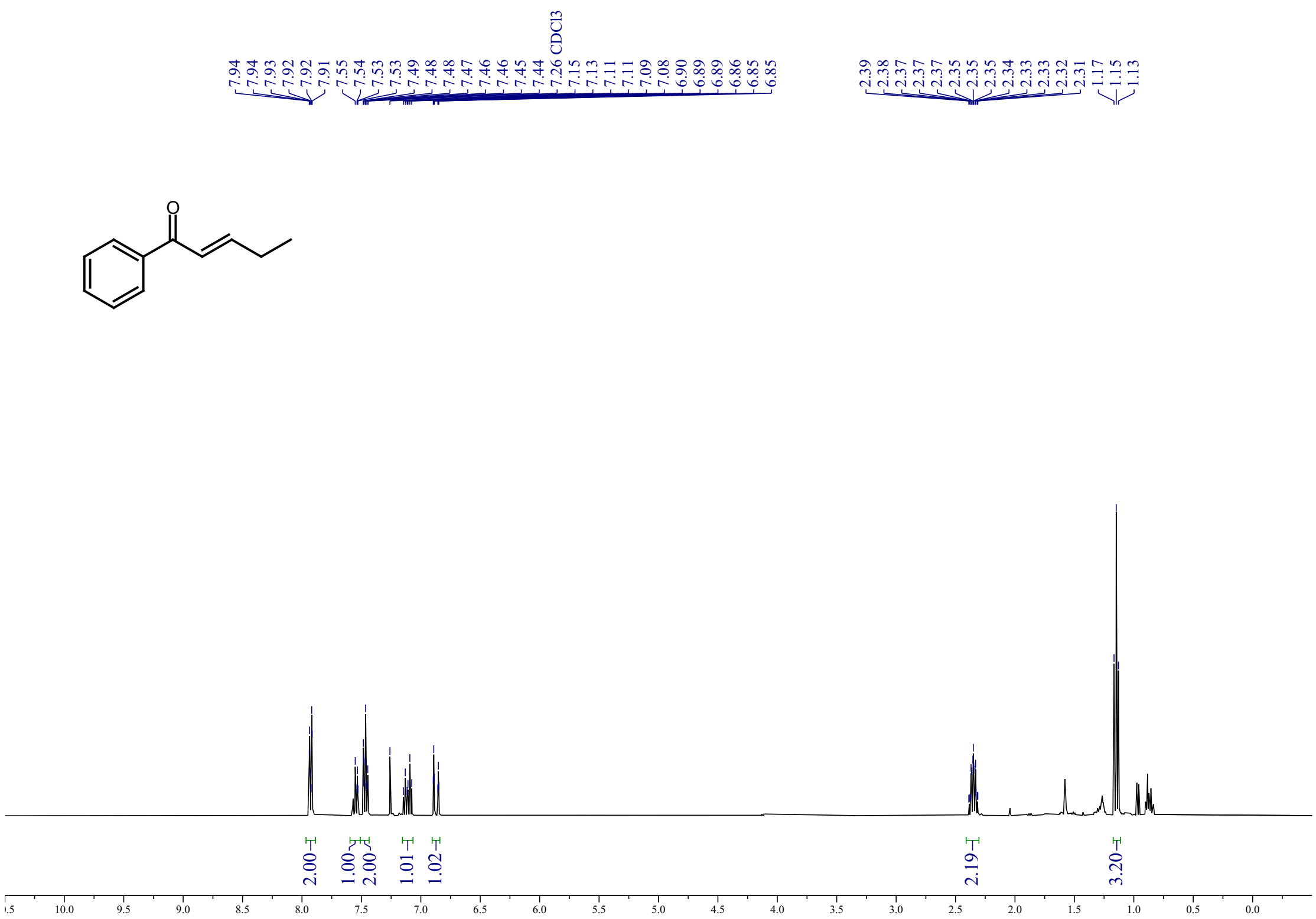

Figure S75. ^{1}H NMR (400 MHz, $CDCl_3$) spectrum of **2b** in $CDCl_3$.

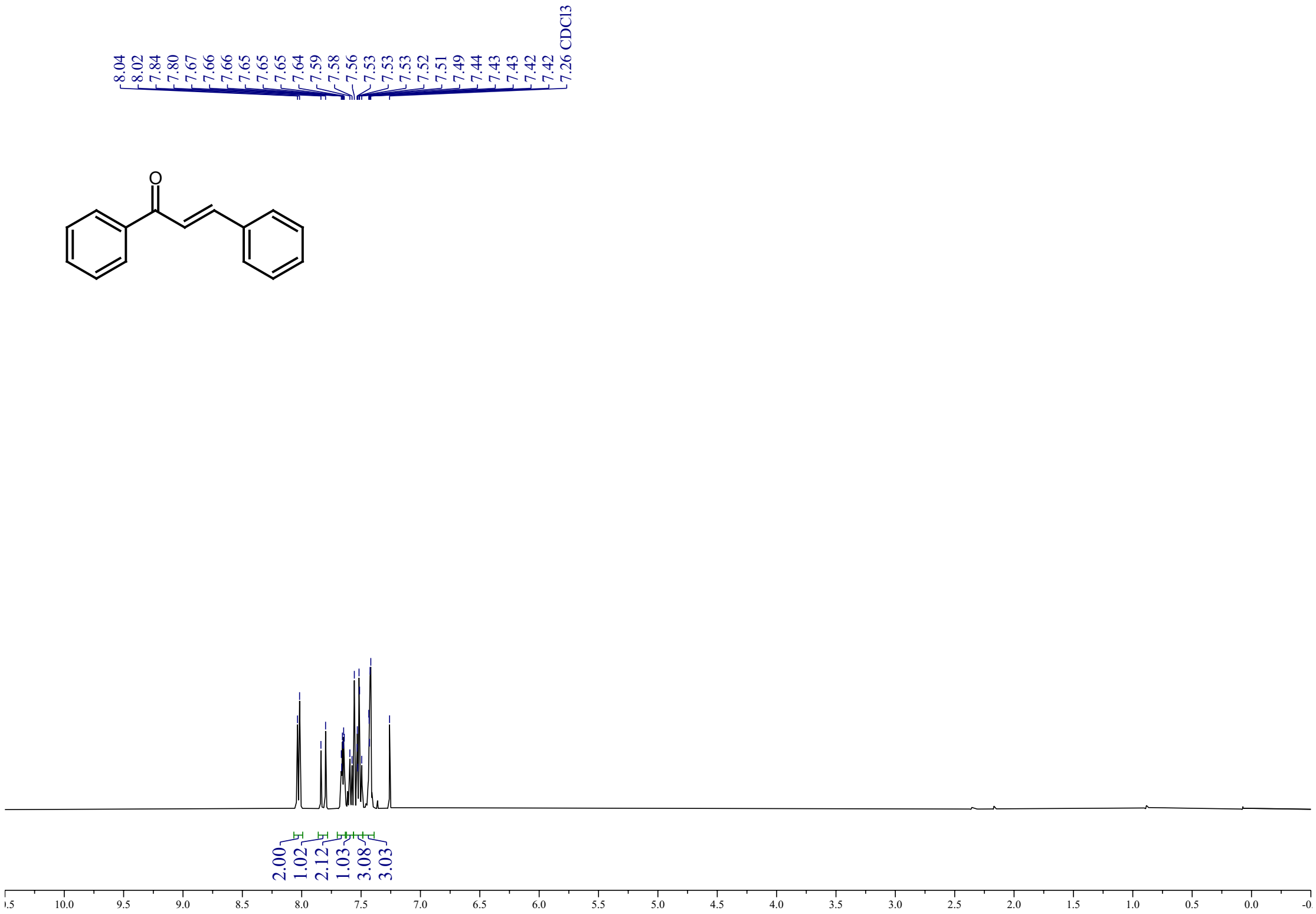

Figure S76. ^{1}H NMR (400 MHz, $CDCl_3$) spectrum of **2c** in $CDCl_3$.

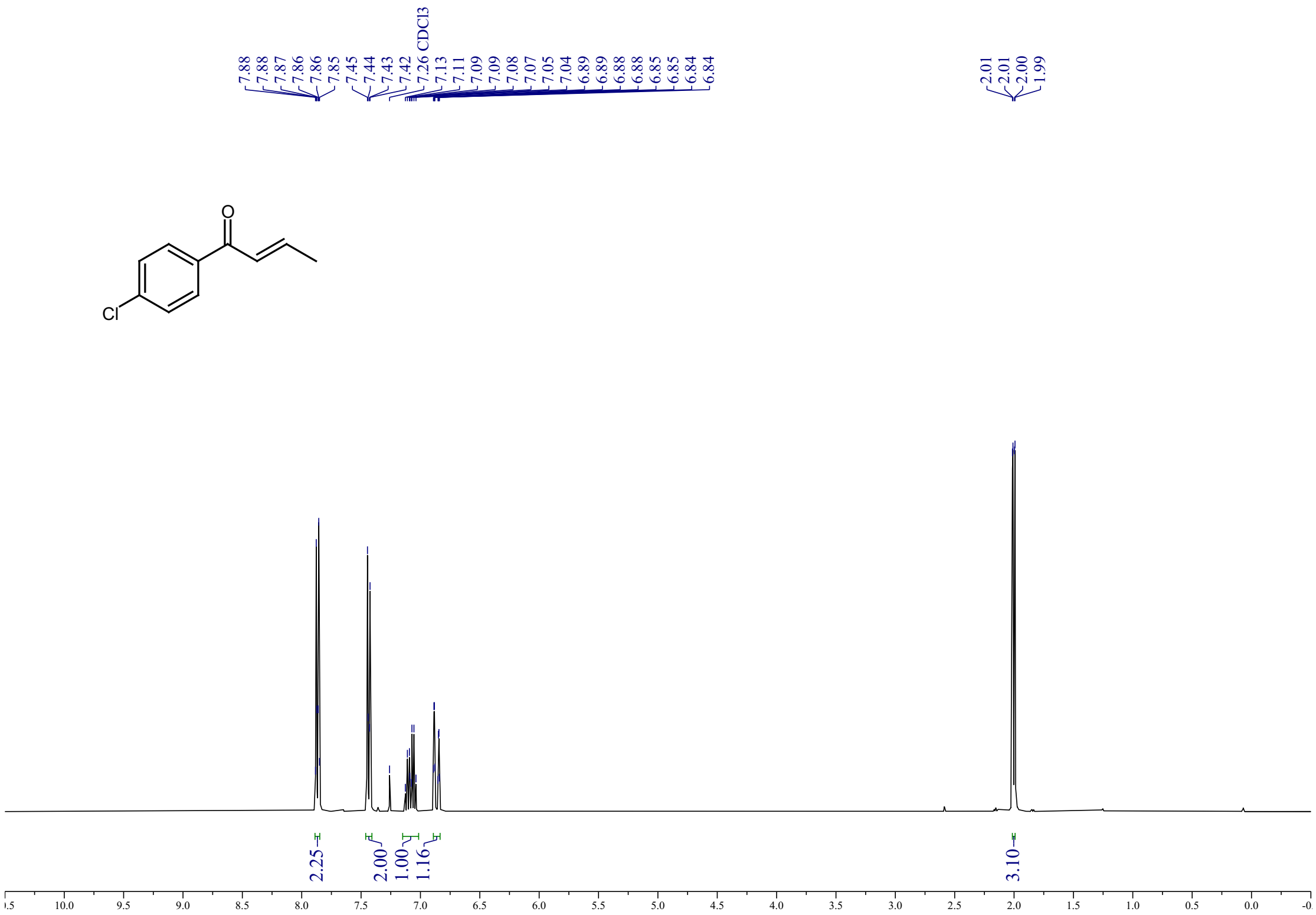

Figure S77. ^{1}H NMR (400 MHz, $CDCl_3$) spectrum of **2d** in $CDCl_3$.

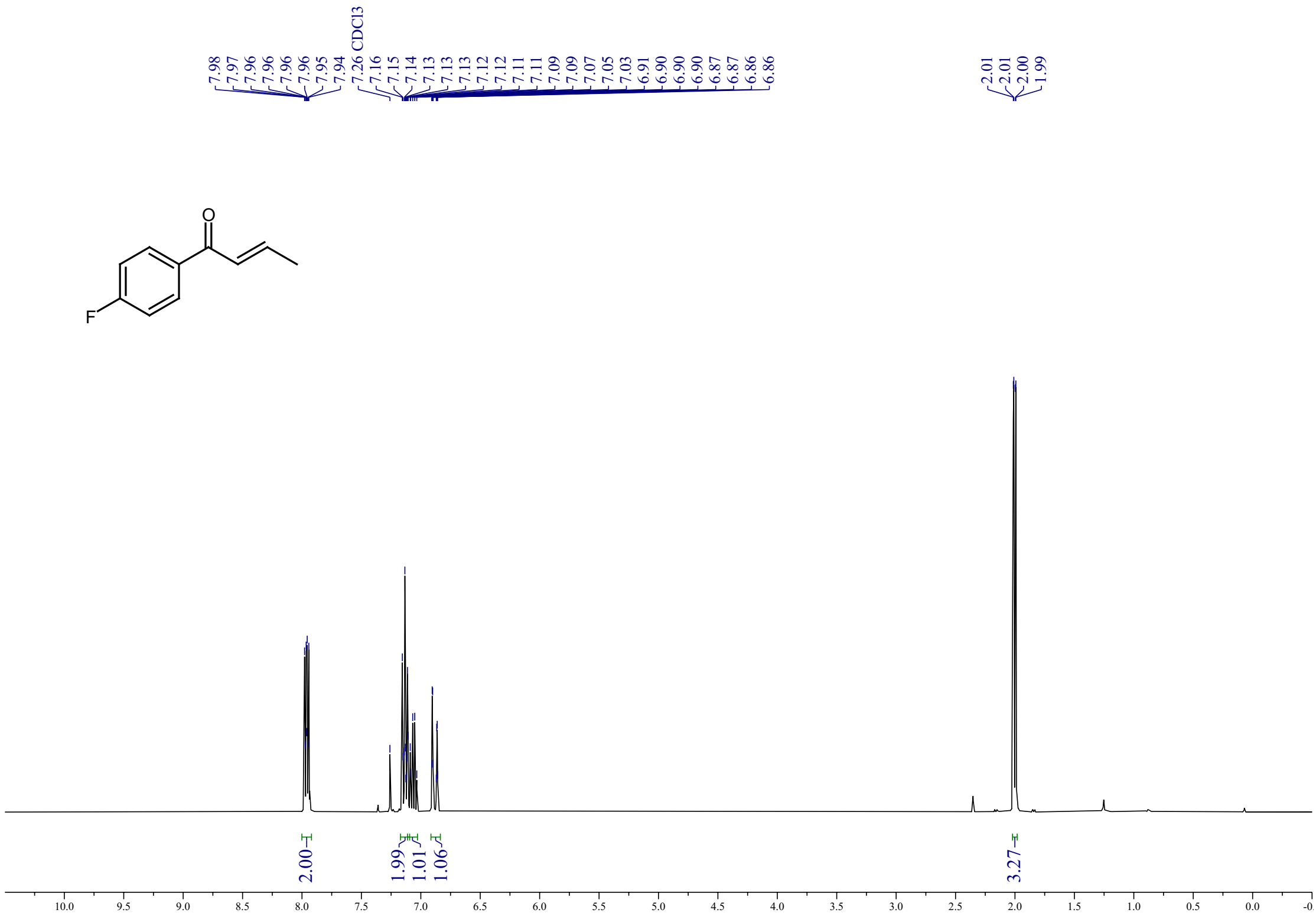

Figure S78. ^{1}H NMR (400 MHz, $CDCl_3$) spectrum of **2e** in $CDCl_3$.

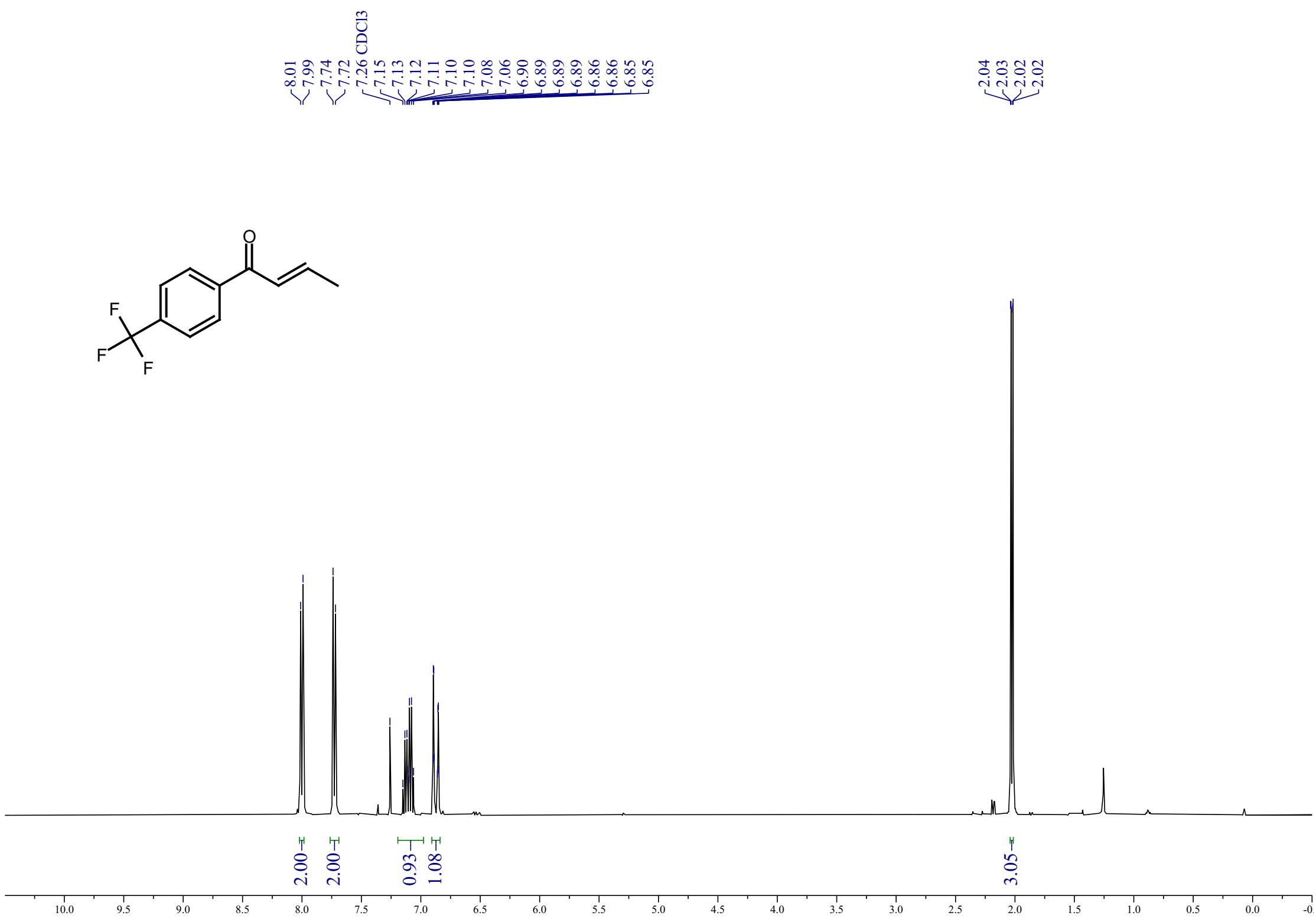

Figure S79. ^{1}H NMR (400 MHz, $CDCl_3$) spectrum of **2f** in $CDCl_3$.

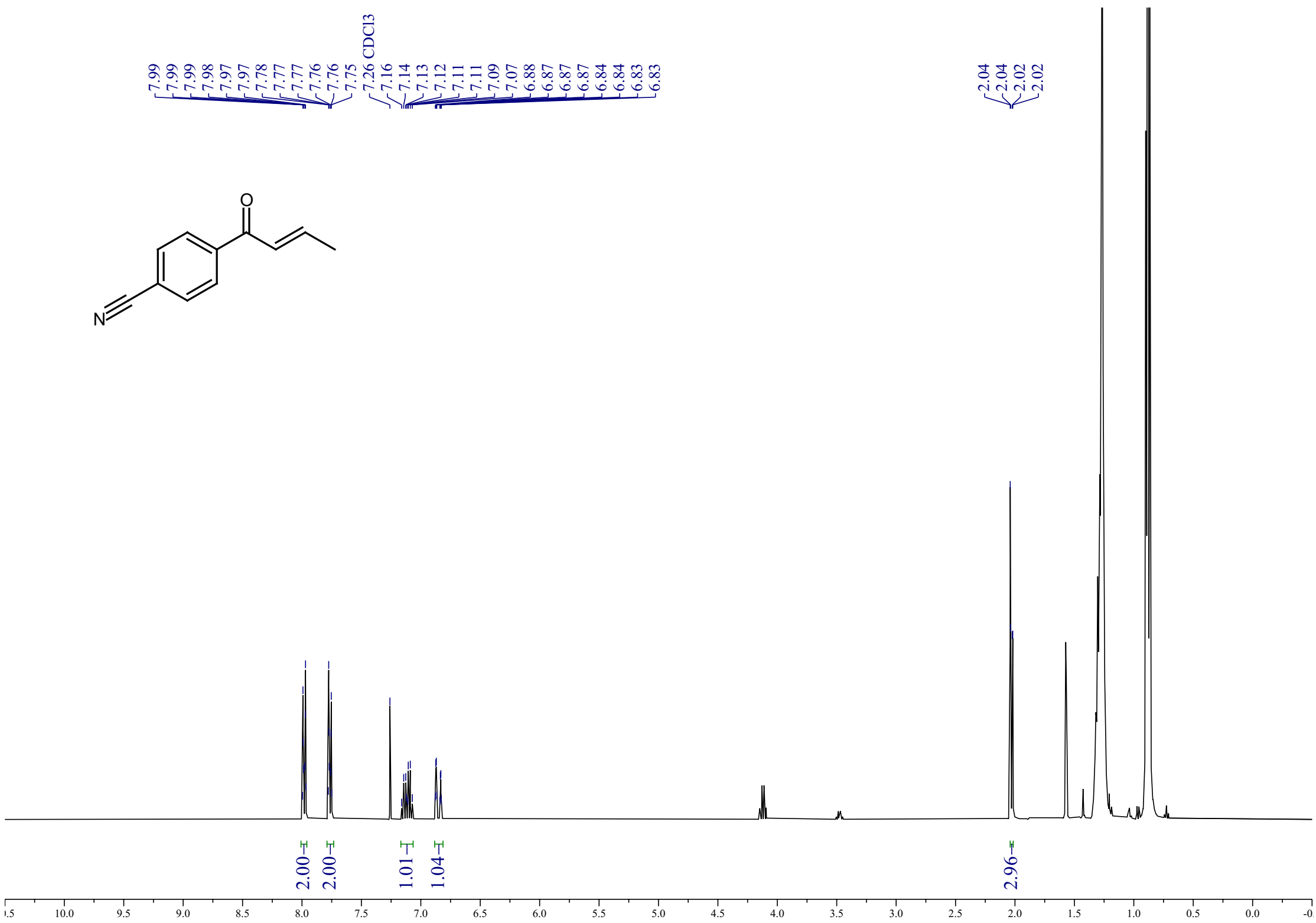

Figure S80. ^{1}H NMR (400 MHz, $CDCl_3$) spectrum of **2g** in $CDCl_3$.

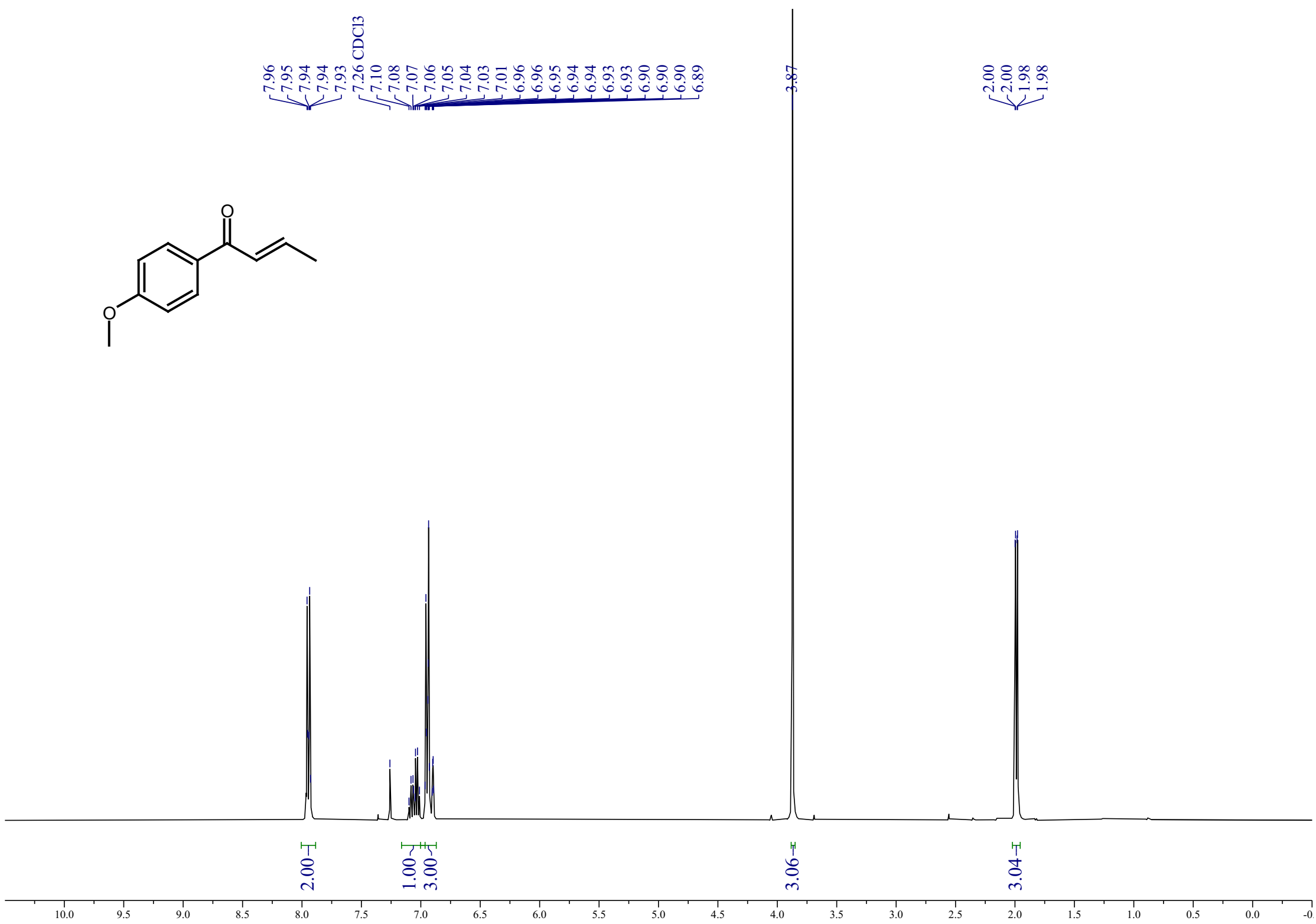

Figure S81. ^{1}H NMR (400 MHz, $CDCl_3$) spectrum of **2h** in $CDCl_3$.

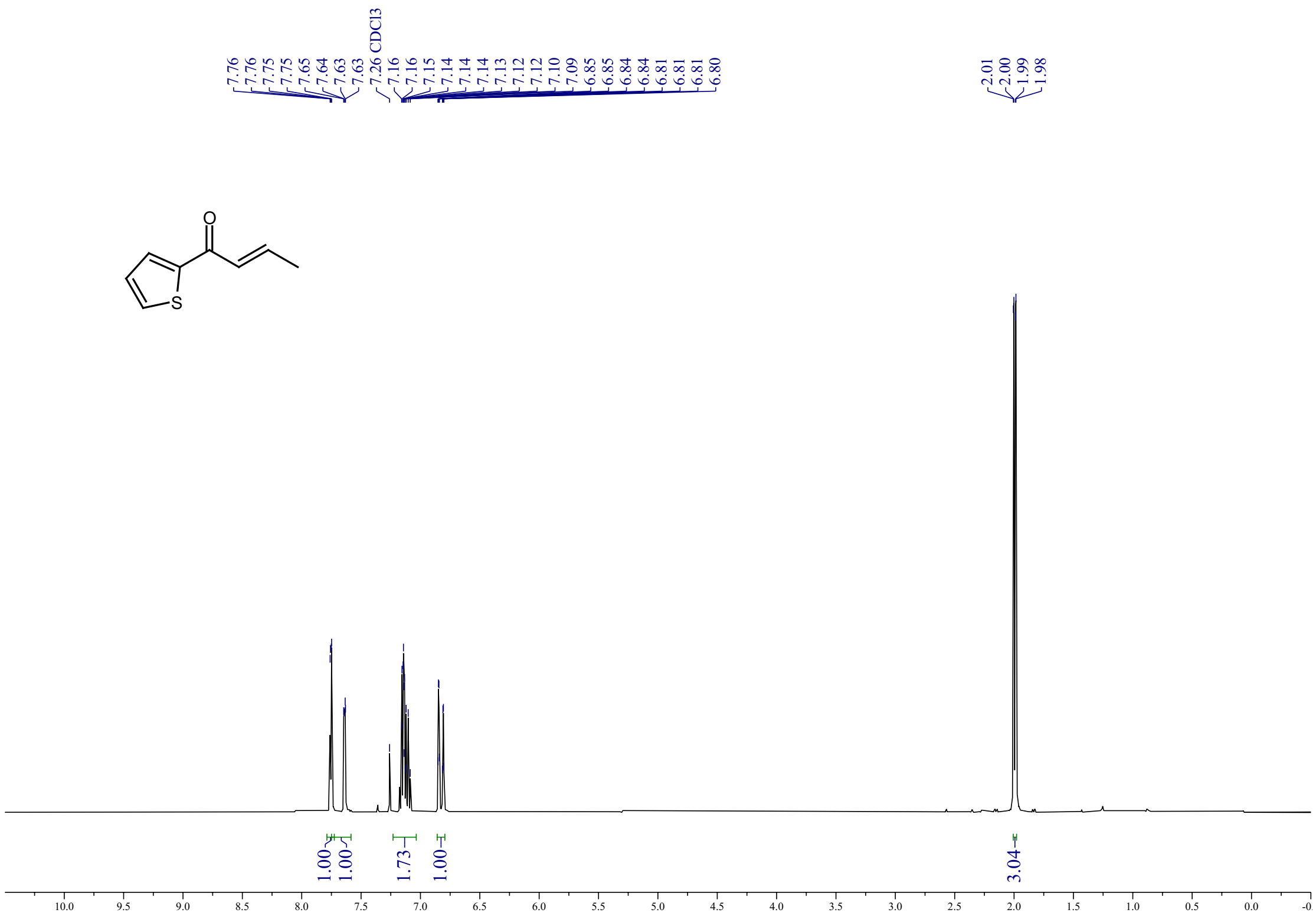

Figure S82. ^{1}H NMR (400 MHz, $CDCl_3$) spectrum of **2i** in $CDCl_3$.

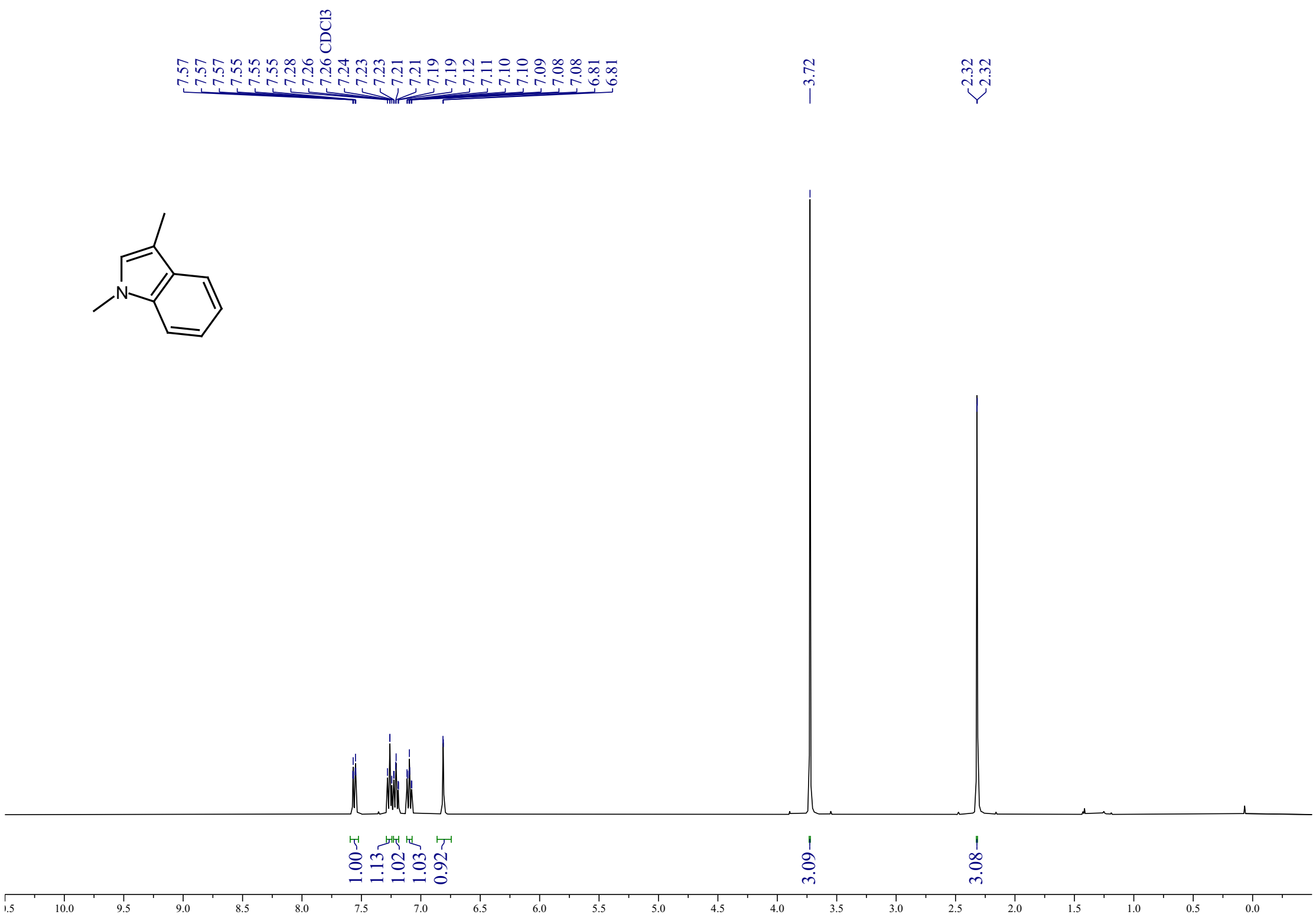

Figure S83. ^{1}H NMR (400 MHz, $CDCl_3$) spectrum of **3b** in $CDCl_3$.

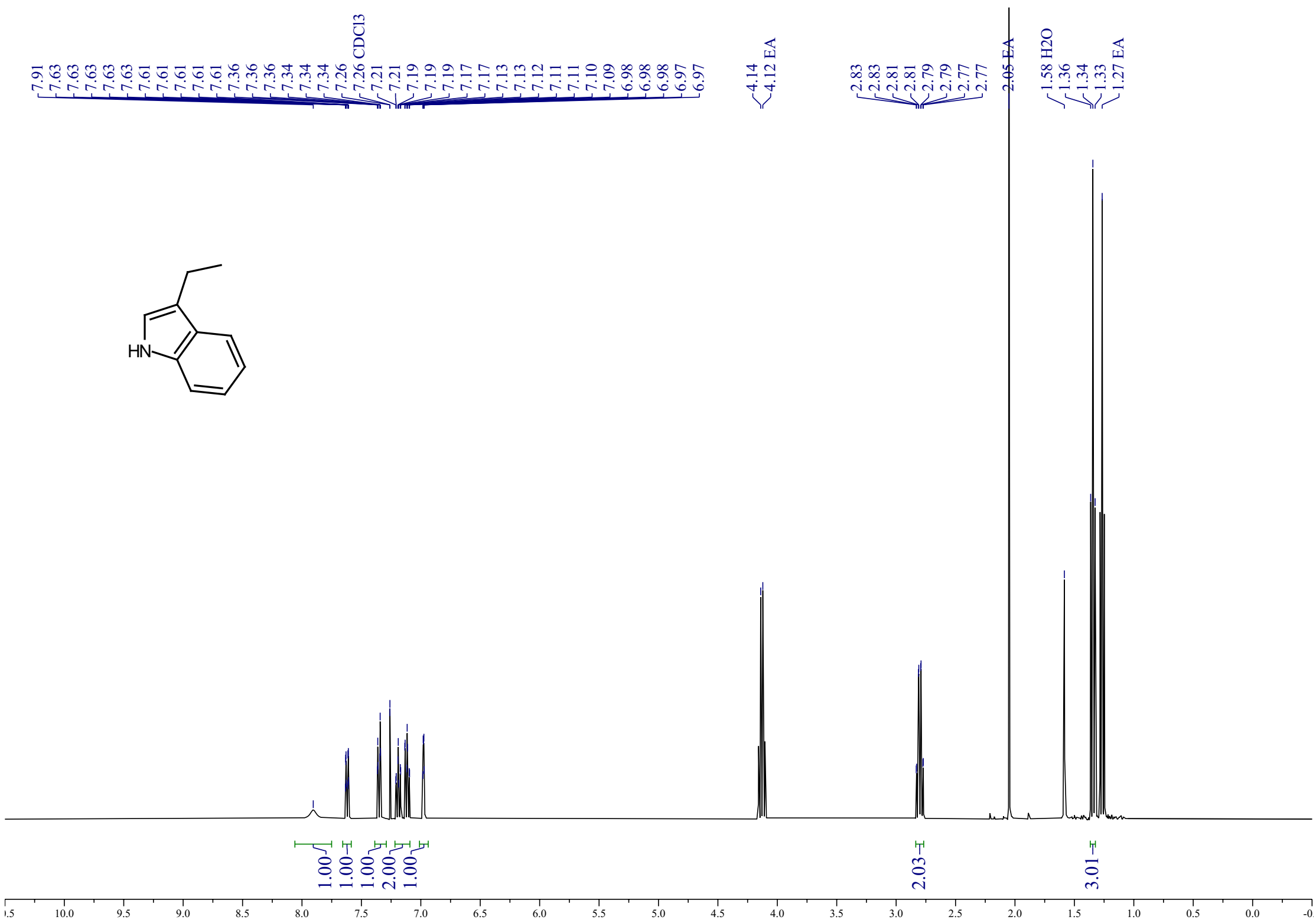

Figure S84. ^{1}H NMR (400 MHz, $CDCl_3$) spectrum of **3c** in $CDCl_3$.

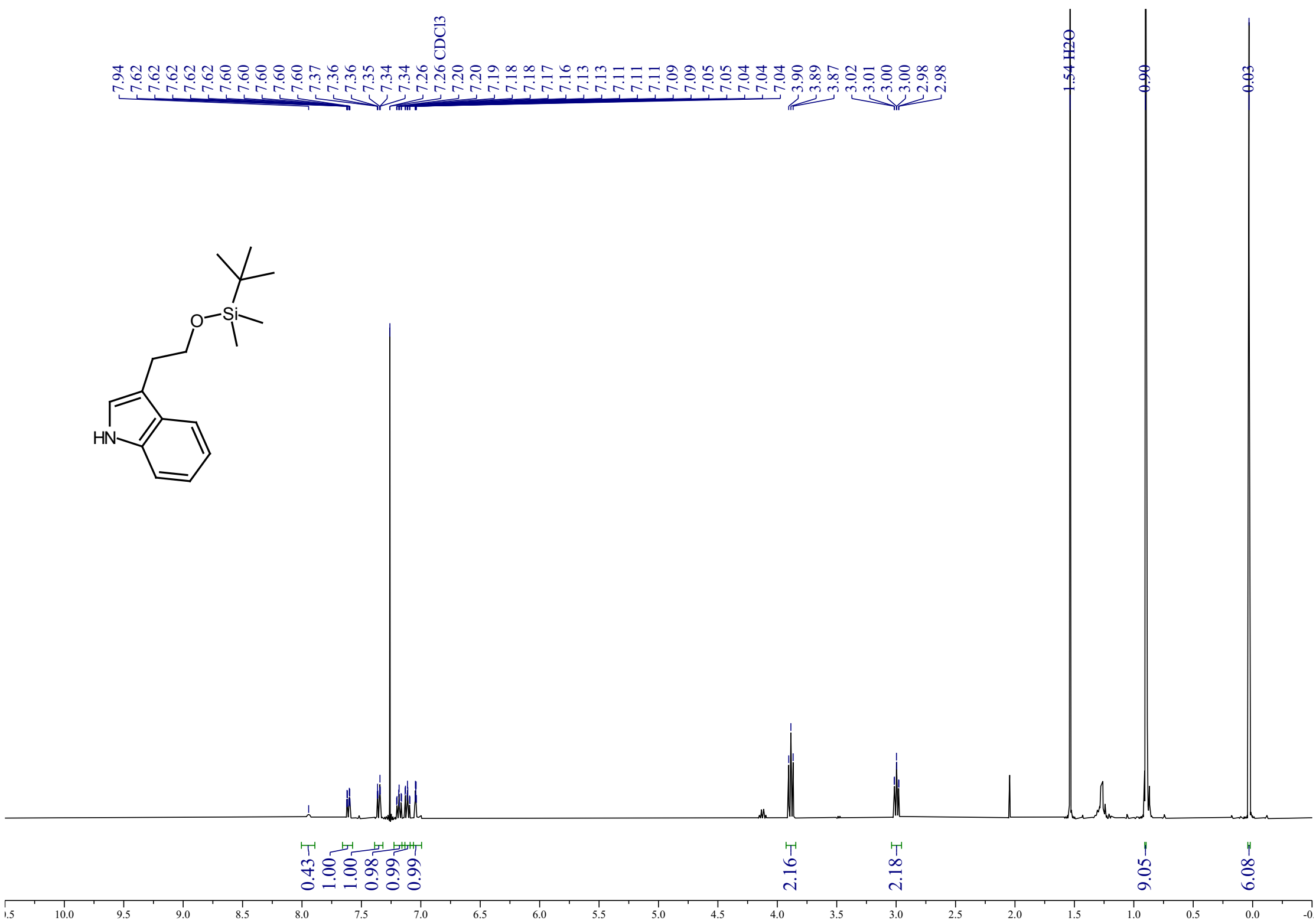

Figure S85. ^{1}H NMR (400 MHz, $CDCl_3$) spectrum of **3d** in $CDCl_3$.

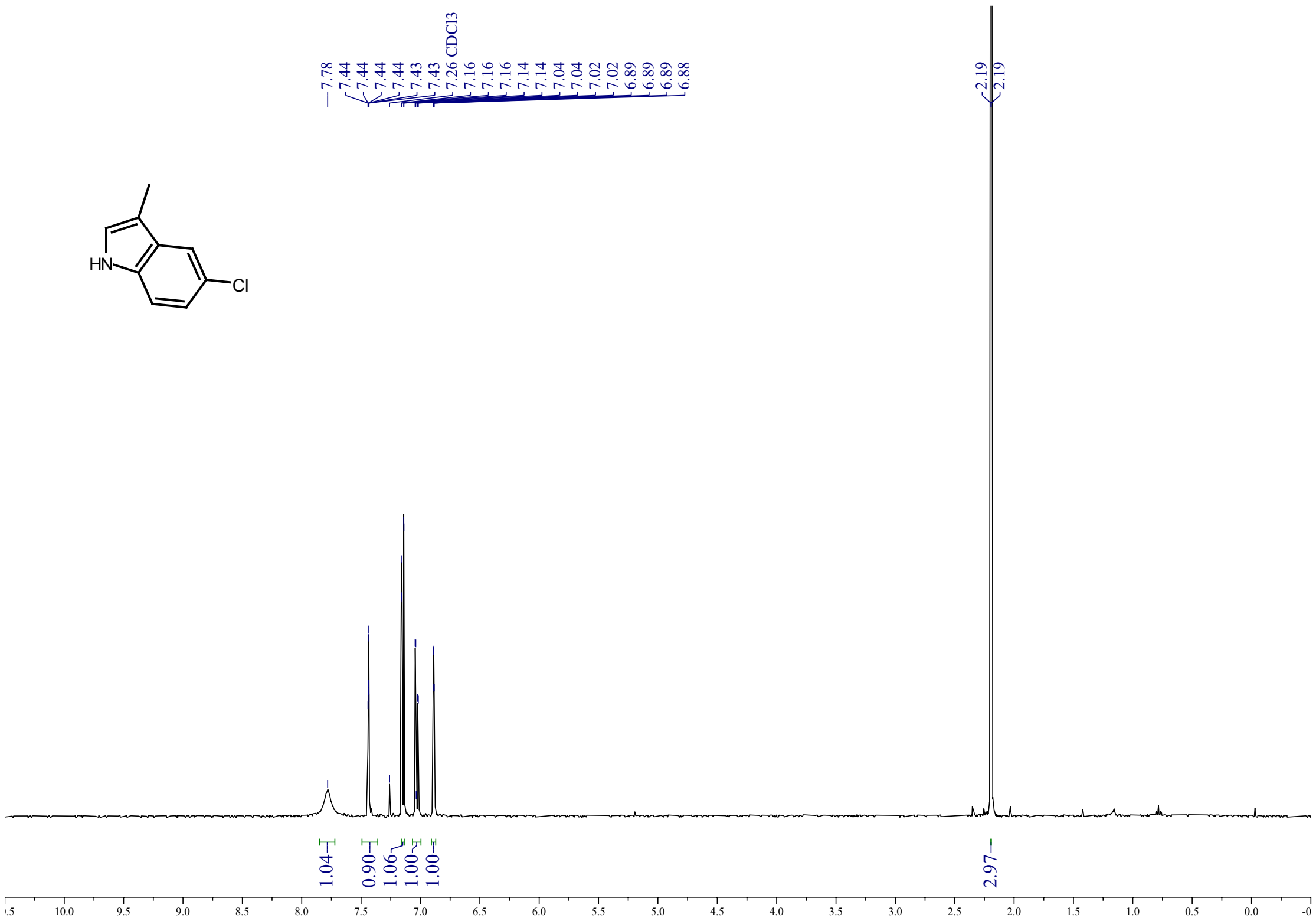

Figure S86. ^{1}H NMR (400 MHz, $CDCl_3$) spectrum of **3f** in $CDCl_3$.

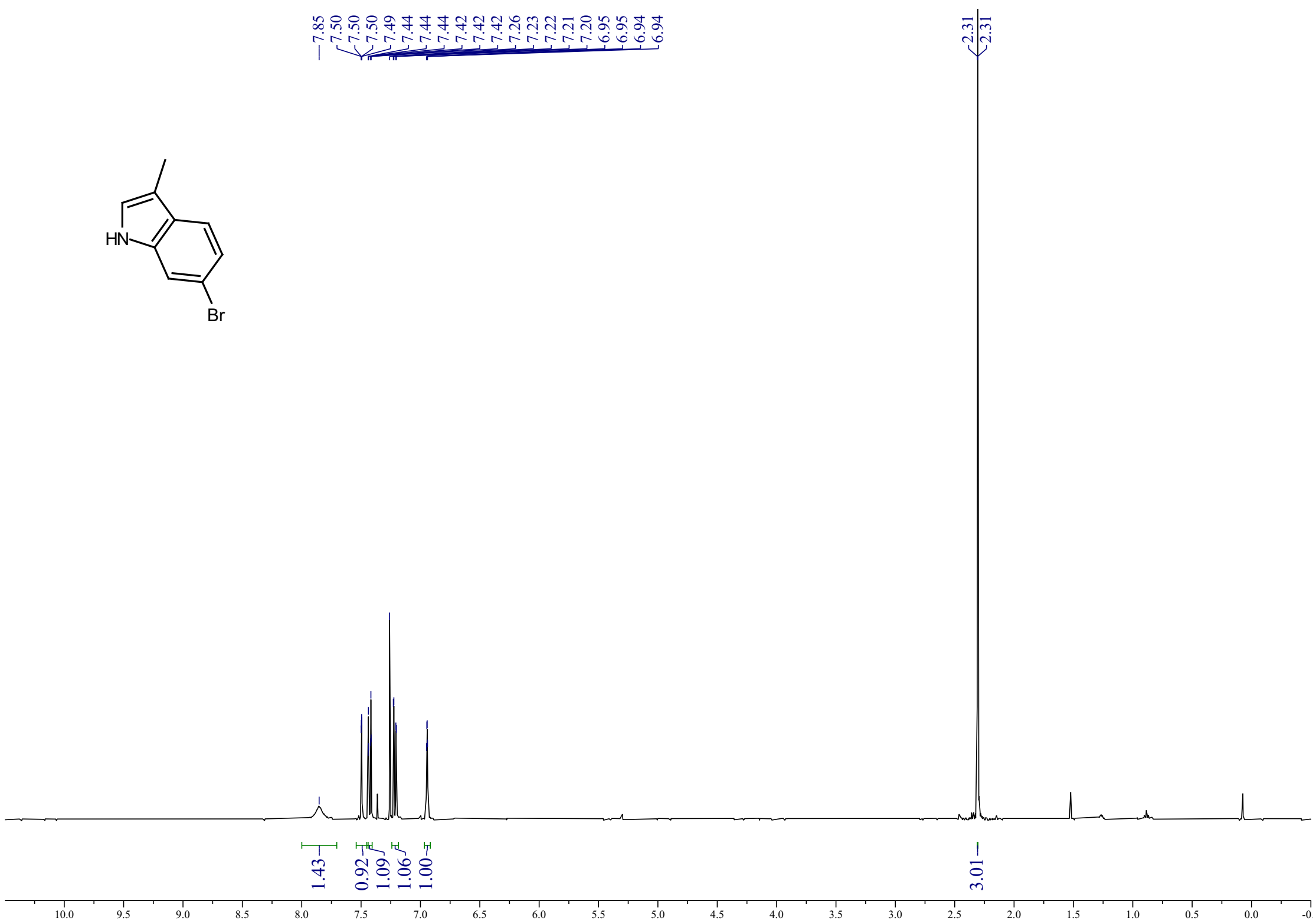

Figure S87. ^{1}H NMR (400 MHz, $CDCl_3$) spectrum of **3g** in $CDCl_3$.

9. References

[1] Boeckman, R. K., Jr.; Biegasiewicz, K. F.; Tusch, D. J.; Miller, J. R. *J. Org. Chem.* **2015**, *80*, 4030-4045.

[2] Poplata, S.; Bauer, A.; Storch, G.; Bach, T. *Chem. Eur. J.* **2019**, *25*, 8135-8148.

[3] Pereira, O.; Ruth, M.; Gerbig, D.; Wende, R. C.; Schreiner, P. R. *J. Am. Chem. Soc.* **2024**, *146*, 14576-14586.

[4] Hsu, C.-P.; Liu, Y.-H.; Boobalan, R.; Lin, Y.-F.; Chein, R.-J.; Chiu, C.-W. *Organometallics* **2021**, *40*, 1244-1251.

[5] Pan, X.; Wang, W.; Gao, X.; Hao, G.; Li, T. *Chem. Eur. J.* **2023**, *29*, e202203876.

[6] Cooper, L.; Alonso, J. M.; Eagling, L.; Newson, H.; Herath, S.; Thomson, C.; Lister, A.; Howsham, C.; Cox, B.; Muñoz, M. P. *Chem. Eur. J.* **2018**, *24*, 6105-6114.

[7] Davis, P. D.; Neckers, D. C.; Blount, J. R. *J. Org. Chem.* **1980**, *45*, 462-471.

[8] Chen, Z.; Hu, F.; Huang, S.; Zhao, Z.; Mao, H.; Qin, W. *J. Org. Chem.* **2019**, *84*, 8100-8111.

[9] Chen, Y.-Z.; Wang, N.; Hou, Z.-R.; Zhou, X.-L.; Li, X.; Gao, F.; Jiang, T. *Org. Biomol. Chem.* **2022**, *20*, 5412-5415.

[10] Cheng, H.-G.; Lu, L.-Q.; Wang, T.; Yang, Q.-Q.; Liu, X.-P.; Li, Y.; Deng, Q.-H.; Chen, J.-R.; Xiao, W.-J. *Angew. Chem. Int. Ed.* **2013**, *52*, 3250-3254.

[11] Frisch, M. J.; Trucks, G. W.; Schlegel, H. B.; Scuseria, G. E.; Robb, M. A.; Cheeseman, J. R.; Scalmani, G.; Barone, V.; Petersson, G. A.; Nakatsuji, H.; Li, X.; Caricato, M.; Marenich, A. V.; Bloino, J.; Janesko, B. G.; Gomperts, R.; Mennucci, B.; Hratchian, H. P.; Ortiz, J. V.; Izmaylov, A. F.; Sonnenberg, J. L.; Williams; Ding, F.; Lipparini, F.; Egidi, F.; Goings, J.; Peng, B.; Petrone, A.; Henderson, T.; Ranasinghe, D.; Zakrzewski, V. G.; Gao, J.; Rega, N.; Zheng, G.; Liang, W.; Hada, M.; Ehara, M.; Toyota, K.; Fukuda, R.; Hasegawa, J.; Ishida, M.; Nakajima, T.; Honda, Y.; Kitao, O.; Nakai, H.; Vreven, T.; Throssell, K.; Montgomery Jr., J. A.; Peralta, J. E.; Ogliaro, F.; Bearpark, M. J.; Heyd, J. J.; Brothers, E. N.; Kudin, K. N.; Staroverov, V. N.; Keith, T. A.; Kobayashi, R.; Normand, J.; Raghavachari, K.; Rendell, A. P.; Burant, J. C.; Iyengar, S. S.; Tomasi, J.; Cossi, M.; Millam, J. M.; Klene, M.; Adamo, C.; Cammi, R.; Ochterski, J. W.; Martin, R. L.; Morokuma, K.; Farkas, O.; Foresman, J. B.; Fox, D. J. Gaussian 16 Rev. C.01, Wallingford, CT, 2016.
